# One-Pot Nucleophilic Organocatalytic Enantioselective [8 + 2] Cycloadditions of Photogenerated Ketenes with Triflate Tropolones

**DOI:** 10.1021/acs.orglett.6c01642

**Published:** 2026-05-28

**Authors:** Aleksandra Murre, Macarena Eugui, Ana C. S. Carvalho, Karl Anker Jørgensen, Mikk Kaasik

**Affiliations:** † Department of Chemistry and Biotechnology, 54561 Tallinn University of Technology, Akadeemia tee 15, 12618 Tallinn, Estonia; ‡ Department of Chemistry, Aarhus University, DK-8000 Aarhus C, Denmark

## Abstract

This work presents diazoketones as ketene precursors under photochemical conditions, in conjunction with an isothiourea catalyst, to enable an enantioselective [8 + 2] cycloaddition with triflate-substituted tropolones affording 7,5-fused lactone scaffolds. The cycloadducts are obtained in up to 99% yield with up to excellent diastereoselectivity (>20:1 d.r.) and enantioselectivity (up to 99% ee). Furthermore, it is demonstrated that the 7,5-fused lactone can undergo diverse attractive transformations.

## Graphical abstract

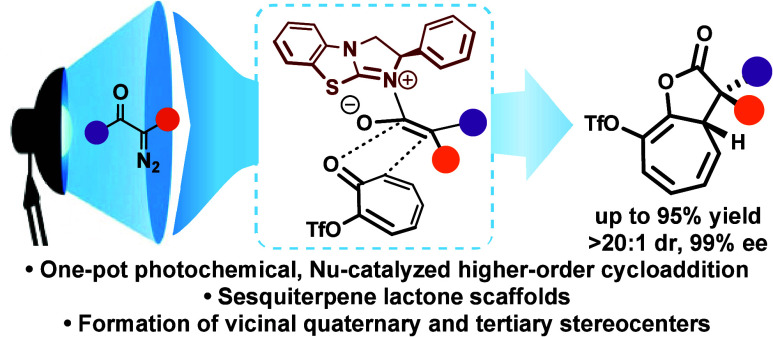

Strategies that facilitate the efficient construction of complex, stereochemically defined frameworks from simple building blocks are essential to modern synthetic chemistry.^1,2^ Cycloadditions are among the most powerful methods for this purpose, enabling rapid formation of multiple bonds and stereocenters in a single step.^3–5^ Despite their well-established reactivity, achieving high levels of selectivity, particularly with reactive intermediates, remains challenging. Enantioselective variants involving reactive intermediates in combination with extended π-systems, such as higher-order cycloadditions (HOCs), have only recently received considerable attention.^6–8^

Enantioselective [8 + 2] cycloadditions can be challenging to control due to the extended π-conjugation of the substrates,^6,9^ which can induce conformational flexibility and competing pericyclic pathways, such as [4 + 2] or [6 + 4] cycloadditions, often via ambimodal transition states.^6,10,11^ These reactions are typically asynchronous or proceed through stepwise mechanisms thereby requiring further constrains on catalytic activation modes that can stabilize zwitterionic intermediates while ensuring effective stereocontrol.^10,12,13^ Furthermore, these challenges are amplified when highly reactive or short-lived intermediates are involved, as their transient nature often compromises both selectivity and efficiency.

Ketenes are among such reactive species that have found application in cycloadditions.^14,15^ Pericàs et al. have reported an enantioselective [8 + 2] cycloaddition of azaheptafulvenes (Scheme 1A) using isothiourea (**ITU**) catalysts to activate ketenes generated from acyl halides.^16^ Song et al. have demonstrated that photogenerated ketenes can be engaged as C1-ammonium enolates under isothiourea catalysis in an enantioselective [4 + 2] cycloaddition (Scheme 1B).^17^ In both cases, enantioselective induction is achieved through nucleophilic catalysis,^18,19^ an approach only sparsely explored in HOCs,^16,20^ and more so for the development of phosphine-mediated allene activation.^21–23^ In this context, the present work explores new opportunities for nucleophilic catalysis in HOCs, which currently mainly rely on aminocatalytic activation of aldehydes.^8^

**Scheme 1:**
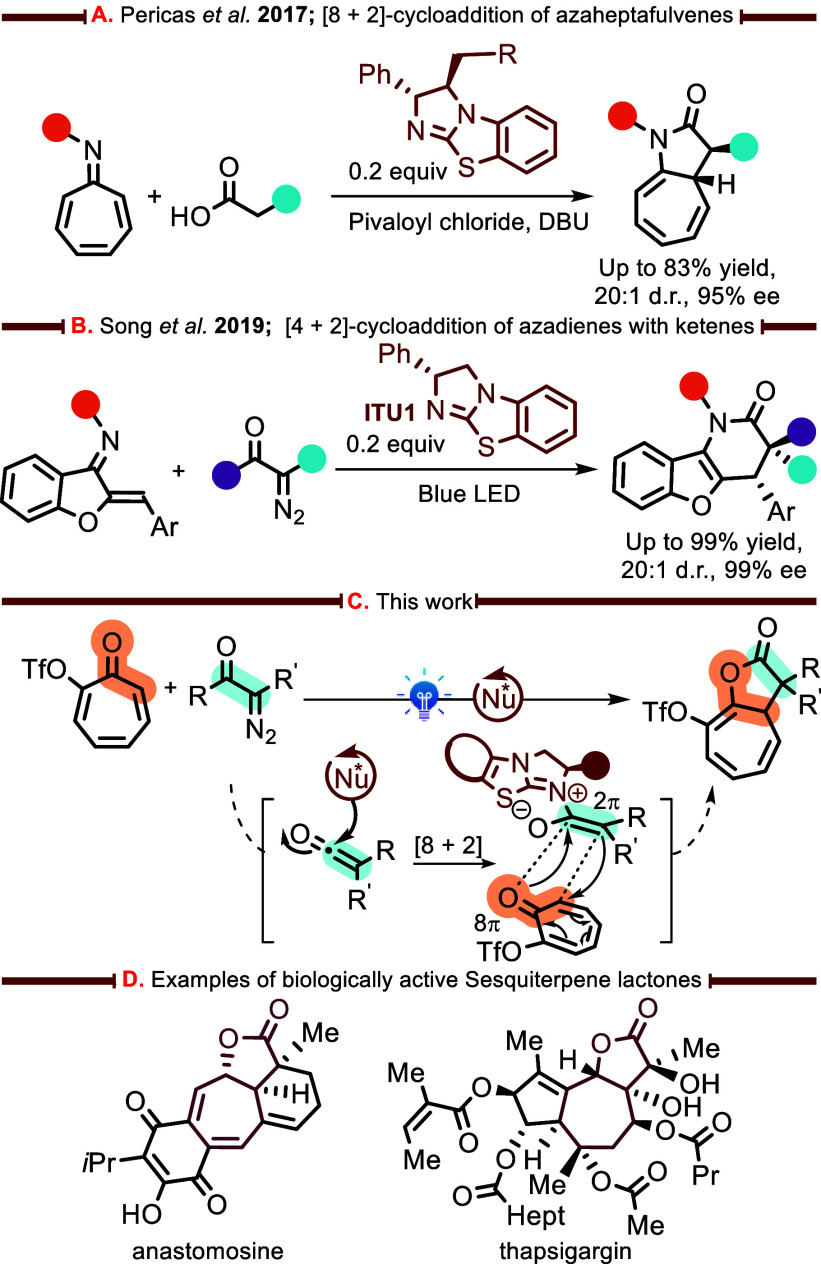
Cycloaddition Reactions Utilizing Ketenes and Bioactive Sesquiterpene Lactones

The photoinduced Wolff rearrangement provides a greener strategy for ketene generation compared to conventional approaches, such as thermal or metal-catalyzed diazo-to-ketene transformations.^24,25^ Given the inherent instability of ketenes, their *in situ* generation and interception improve both operational simplicity and process efficiency.^26,27^

Herein, we explore the enantioselective interception of highly reactive, photochemically generated ketenes in HOCs (Scheme 1C),^28–30^ thereby expanding the enantioselective reactivity of nonbenzenoid aromatic systems such as tropolones.^31–33^ This approach enables rapid access to architecturally complex frameworks from simple precursors. The targeted cycloadduct features a 7,5-fused lactone core, a privileged structural motif present in Sesquiterpene lactones, a class of natural products renowned for their structural diversity and pharmacological relevance (Scheme 1D).^34–36^ We report the synthesis of representatives of this scaffold incorporating an all-carbon quaternary stereocenter, whose formation remains a challenge in synthetic chemistry,^37,38^ and demonstrate the potential for further diversification of the scaffold.

The reaction concept was initially explored using tropone **1a** and diazoketone **2a**. Unfortunately, the application of different nucleophilic organocatalysts afforded either racemic product in low yield or no reaction at all (Scheme 2A, see Supporting Information, ). It was envisioned that a more electrophilic tropone species would better match the nucleophilic C1-ammonium enolate, thus we turned our attention to triflate tropolone **1b**. Gratifyingly, with **ITU1** the desired product formed in an enantioenriched manner (Scheme 2B). Interestingly, with the change of the tropone species, a change occurred in the favored diastereoisomer from *syn* for **4a** to *anti* for **3a**.

**Scheme 2:**
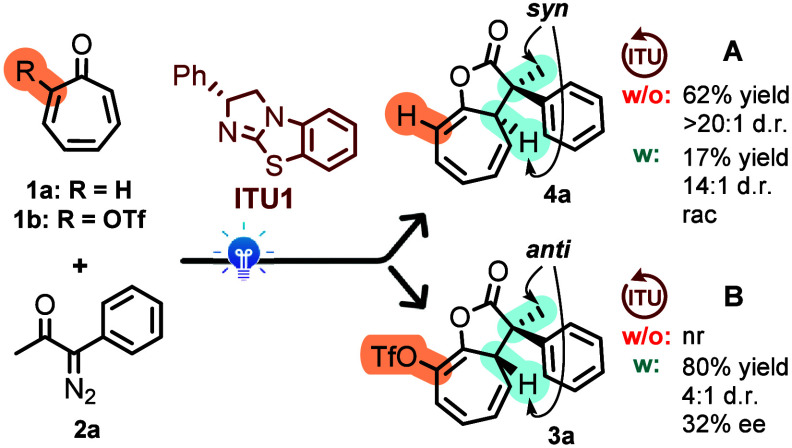
Initial Experiments with (A) Tropone 1a and (B) Triflate Tropolone 1b

Initial screening revealed poor reproducibility due to the high reactivity of intermediates and the strong dependence on reaction conditions (see Supporting Information, ). Trace moisture and air led to a significant drop in conversion and enantioselectivity, necessitating strictly anhydrous, oxygen-free conditions. No reaction was observed in the absence of light. Triflate tropolone **1b** and diazoketone **2a** are incompatible with one another, which is more pronounced in the absence of solvent and results in the decomposition of **2a** during setup. Acid additives diminished both yield and selectivity, whereas variations in setup (e.g., Schlenk-tube position, LED distance, stirring rate) had no measurable impact.

For all the optimization studies, a full one-pot procedure was employed, in which [8 + 2] cycloadduct **3a** was reduced to **4a** for enantiomeric excess determination (Table 1). After extensive initial screening of various catalysts, isothiourea catalysts were identified as the most promising (see Supporting Information, ). Among them, isothiourea **ITU1** was selected, as it demonstrated the best combination of both yield and selectivity (Table 1, entries 5–7, see also Supporting Information, ). Further optimization of solvents revealed that CPME and toluene provide comparable performance (entries 1–4). However, CPME enabled full conversion at lower temperature with encouraging enantiomeric excess (78% ee, entry 5). The reaction was sensitive to catalyst loading, with 20 mol % identified as optimal (entries 5,8, see also Supporting Information, ). The use of drying agents to ensure an even more moisture-free environment was found to be unnecessary (entry 9). It was found that the yield and diastereo- and enantioselectivities were all lower when the ketene was preformed in a separate reaction, indicating that it is unstable under the applied conditions (entry 10).^17^ Screening of other variables confirmed CPME at −40 °C as the optimal conditions (see Supporting Information, ).

**Table 1:**
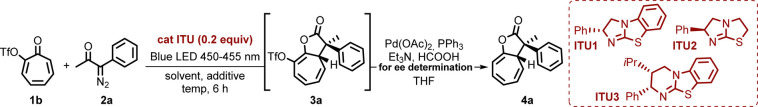
Optimization of the Reaction Conditions^a^

					NMR quantity			ee (**4a**), %	
Entry	solvent	temp, °C	cat. ITU	additives	**1b**, %	**3a**, %	d.r.	maj	min
1	Toluene	0	**ITU1**	–	16	72	4.4:1	71	47
2^b^	DCM	0	**ITU1**	–	53	37	2.1:1	30	17
3^b^	EtOAc	0	**ITU1**	–	30	49	4.4:1	60	20
4	CPME	0	**ITU1**	–	13	78	4.2:1	75	40
5	CPME	–40	**ITU1**	–	<3	99	4:1	78	53
6	CPME	–40	**ITU2**	–	12	60	0.9:1	–26	–54
7	CPME	–40	**ITU3**	–	5	76	0.7:1	–10	10
8	CPME	–40	**ITU1** (0.1 equiv)	–	25	66	3.4:1	71	47
9^b^	CPME	–40	**ITU1**	3Å MS	11	18	3.5:1	77	31
10^b^	CPME	0	**ITU1**	Preprepared ketene	86	14	2.5:1	30	16

a Reaction conditions: 0.05 mmol scale, **2a** (1.2 equiv), cat **ITU** (0.2 equiv), solvent (0.5 mL). The major enantiomer of the major diastereomer is shown in the scheme. The NMR quantity and diastereomeric ratio were determined by ^19^F NMR with 1,4-(CF_3_)_2_C_6_H_4_ as an internal standard. ee was determined after reduction of **3a** to **4a** by HPLC analysis on a chiral stationary phase.

b For 16 h.

Additional experiments were conducted to investigate the temperature dependence of both ketene formation and the overall cascade reaction (see Supporting Information, Figures S3–S4. In the absence of **1b** and **ITU1**, the ketene NMR yield peaked within 3 h but did not exceed 50%, followed by a steady decrease in its concentration. In contrast, complete conversion to **3** was achieved in the cascade reaction within 4 h at −40 °C, further high-lighting the reactivity of the ketene intermediate and the advantage of the one-pot approach.

Substrate scope studies revealed a generally good tolerance to structural variations. Replacement of the methyl group (Scheme 3, **3a**) in the diazoketone with an ethyl group was well tolerated, affording **3b** with comparable results. Diazoketones **2c**–**f** bearing halogen substituents at various positions on the phenyl ring also reacted smoothly with tropolone derivative **1b**, yielding the corresponding cycloheptatriene-fused γ-lactones **3c**–**f**. Notably, bulkier cyclohexane-based diazoketones **2g**–**j** were found to be suitable substrates for this transformation. These not only enabled the formation of structurally intriguing spiro-cycloheptatriene-fused γ-lactones **3g**–**i** but also significantly enhanced enantioselectivity with up to 99% ee. In addition, diastereoselectivity was either markedly enhanced, or the resulting diastereomers were readily separable by chromatography, allowing the isolation of single diastereoisomers with excellent enantiomeric excess (**3g**,**i**).

**Scheme 3:**
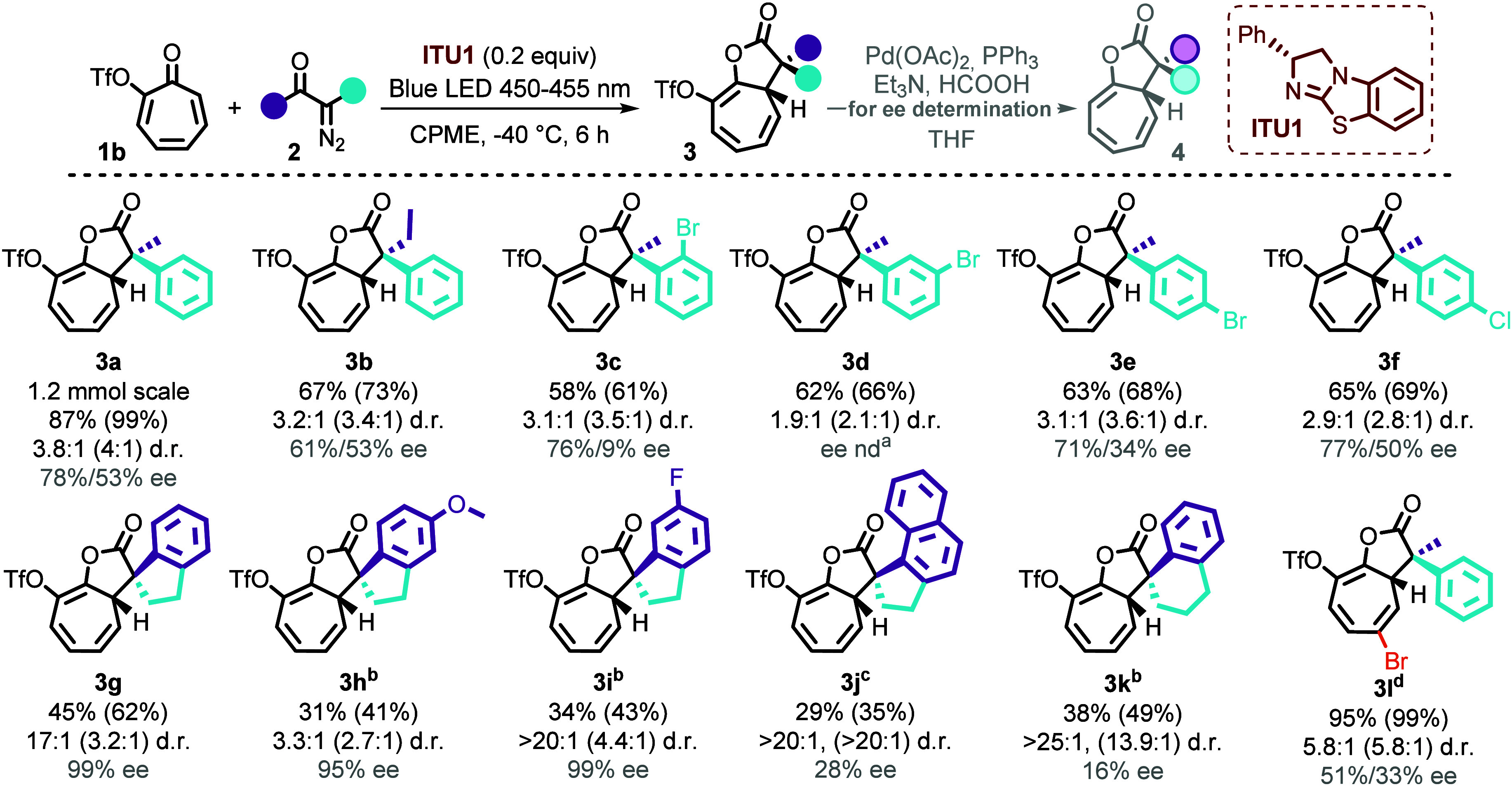
Scope of the Developed Reaction

Reaction conditions: 0.1 mmol scale, **2** (1.2 equiv), **ITU1** (0.2 equiv), CPME (1 mL), −40 °C. The major enantiomer of the major diastereomer is the one depicted. Information in the brackets refers to the NMR yield and the diastereomeric ratio, determined by ^19^F NMR with 1,4-(CF_3_)_2_C_6_H_4_ as an internal standard. The ee was determined after reduction of **3a** to **4a** by HPLC analysis on a chiral stationary phase.
a nd – not determined.
b For 16 h.
c For 24 h at −20 °C.
d
**1c** instead of **1b**, for 3 h.

Cycloheptane-based diazoketones were also viable substrates; however, enantioselectivity of the corresponding product was reduced, most likely due to increased seven-membered ring flexibility (**3k**). Addition of a bromo-substituent into the triflate tropolone was tolerated in terms of yield and led to a slightly improved diastereoselectivity at the expense of enantioselectivity (**3l**). Overall, the reaction is efficient with triflate tropolones and mixed alkyl–aryl diazoketones; with limitations outlined in Supporting Information, Scheme S2.

The structures of **4j** and **4k** were confirmed by single-crystal X-ray crystallography (see Supporting Information). The absolute configurations of the formed stereogenic centers in all products were assigned as 3*R*,3a*S* by analogy to the X-ray structure of compound **8** (Scheme 4, see also Supporting Information). Importantly, the robustness of the methodology was confirmed by a 1.2 mmol scale-up, which delivered full conversion of **1b** to **3a** within 6 h without loss of yield or enantioselectivity.

**Scheme 4:**
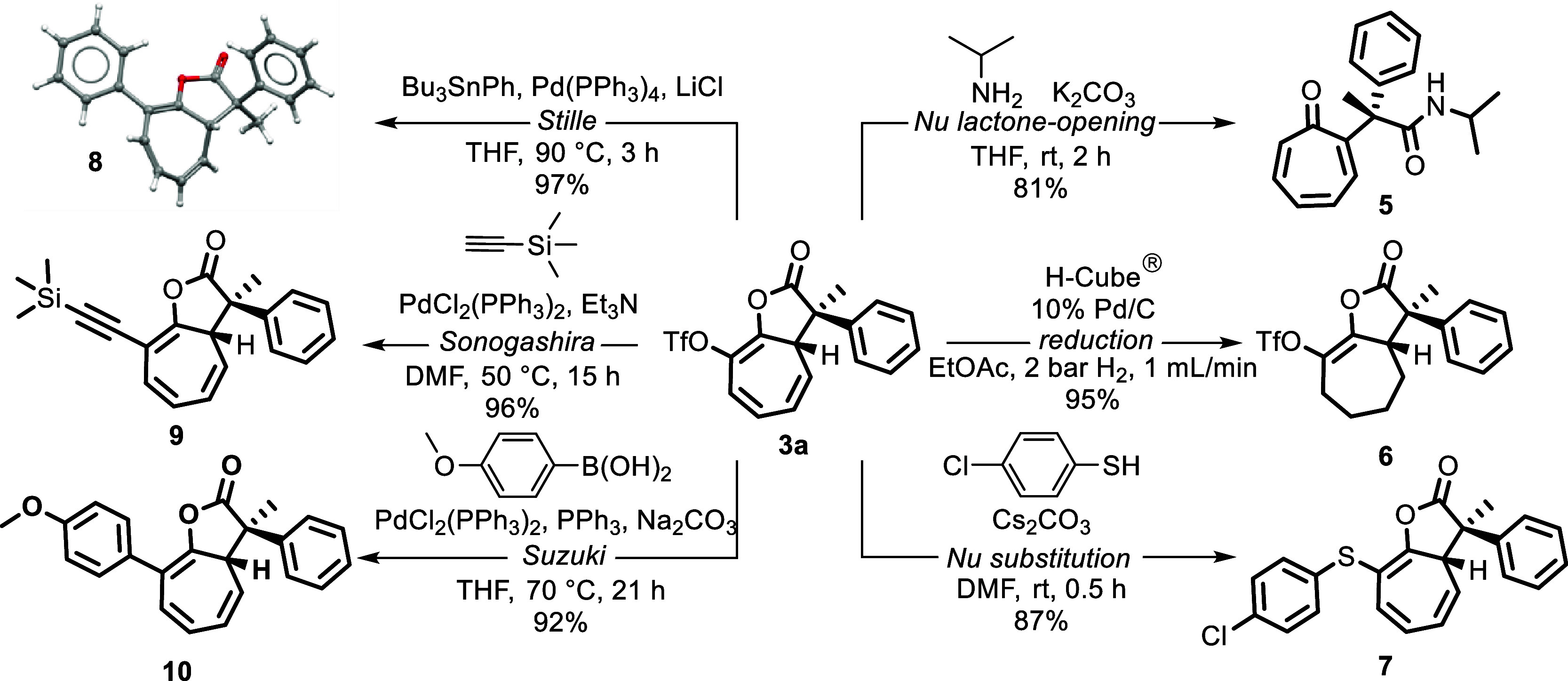
Transformations of **3a**

Following the scope studies, the synthetic utility of the obtained scaffolds was further demonstrated. The lactone core was opened with isopropylamine to give tropone **5** in 81% yield (Scheme 4). Given the prevalence of lactone motifs in bioactive compounds, compound **3a** served as a platform for orthogonal derivatizations, allowing selective functional group manipulation and access to diverse analogues. Catalytic hydrogenation over palladium enabled selective reduction of two of the three double bonds within the cycloheptatriene core, providing product **6** with 95% yield. Furthermore, the triflate group could be selectively engaged in a variety of transformations. Nucleophilic substitution of the triflate group gave thioether **7** in 87% yield. The triflate underwent Stille, Sonogashira, and Suzuki cross-couplings to afford **8**–**10** in 97%, 96%, and 92% yield, respectively. These transformations underscore the broad functionalization potential of the cycloaddition products **3**, while also demonstrating the strategic value of the triflate group–initially introduced as a necessary yet seemingly superfluous substituent for enabling the reaction.

In summary, photochemically generated ketenes act as effective 2π partners that engage tropolone derivatives in an unprecedented higher-order cycloaddition cascade. The one-pot process enables *in situ* generation and interception of the highly reactive ketene intermediate, thereby establishing a powerful platform for the development of new cascade reactions. Notably, the triflate group is not only essential for the cycloaddition but also serves as a versatile handle for orthogonal derivatization. Overall, this strategy and the synthetic flexibility of the resulting products provide access from simple precursors to architecturally complex and sterically demanding scaffolds related to pharmacologically relevant sesquiterpene lactones.

## Supplementary Material





## Data Availability

The data underlying this study are available in the published article and its .

## Notes

Optimization data, reproducibility study, experimental procedures, characterization of new compounds, copies of NMR and HPLC data, X-ray crystallographic data (PDF)
Video of diazoketone **2a** decomposition (MP4)
