# Supplementary material for: One-Pot Nucleophilic Organocatalytic Enantioselective [8 + 2] Cycloadditions of Photogenerated Ketenes with Triflate Tropolones
Source: Org Lett. 2026 May 28;28(23):7310–4. doi: 10.1021/acs.orglett.6c01642 (PMC13270640; doi:10.1021/acs.orglett.6c01642)
Supplement: Supplementary file 1 [file ol6c01642_si_001.pdf]

One-Pot Nucleophilic Organocatalytic Enantioselective [8+2] Cycloadditions of Photogenerated Ketenes with Triflate Tropolones

SUPPORTING INFORMATION

Aleksandra Murre,† Macarena Eugui,‡ Ana C. S. Carvalho,†
Karl Anker Jørgensen,‡ Mikk Kaasik,*†‡
†Tallinn University of Technology, Estonia
‡ Aarhus University, Denmark
E-mail: mikk.kaasik@taltech.ee

Contents

1. General information 2
2. Photochemical Setup 3
3. Optimization of the reaction with both photochemical setups 6
Initial screening – setup A 6
Additional screening – setup B 8
4. Reproducibility studies 11
5. Ketene formation correlation with reaction kinetics 12
6. Additional scope examples and limitations 14
7. Experimental part 15
Synthesis of the starting materials 15
General procedure for the photochemical enantioselective [8+2]-cycloadditions 20
Late-stage modifications 30
8. NMR 34
^{1}H and ^{13}C NMR spectra of newly synthesized diazoketones **2** 34
^{1}H and ^{13}C NMR spectra of tropolone derivative **1e** 36
^{1}H and ^{13}C NMR spectra of products 3 and derivatizations products **5**-**10** 38
9. HPLC 77
HPLC chromatograms of racemic and asymmetric products **4** 77
HPLC chromatograms of derivatization product **8** 83
10. SCXRD analysis 86
11. Specifications and datasheets for the UV-LED lamps 91
12. References 94

1. General information

^{1}H and ^{13}C NMR spectra were recorded on a Bruker Avance III instrument at 400 MHz for ^{1}H, 100.6 MHz for ^{13}C and 376 MHz for ^{19}F. Chemical shifts (d) are reported in ppm relative to residual solvent signal $CDCl_3$ (7.26 ppm, 77.16 ppm) or C_6D_6 (7.16 ppm, 128.06 ppm). NMR data was processed using the MestReNova 14.2.1 software packages. All peak assignments are confirmed by 2D experiments (^{1}H-^{1}H COSY, ^{1}H-^{13}C HSQC, ^{1}H-^{13}C HMBC). In ^{13}C NMR, 2 C in brackets refers to either two chemically equivalent or two overlapping unique carbon signals. Known products were characterized by comparing to the corresponding ^{1}H NMR and ^{13}C NMR from literature. The names of all products were generated using the PerkinElmer ChemDraw Professional v.23.1.1.3 software package. HRMS measurements were performed on Agilent 6540 UHD Accurate-Mass Q-TOF LC/MS system (Agilent Technologies, Santa Clara, CA, US) equipped with AJS-ESI source. Precoated silica gel plates (Supelco Sigma-Aldrich™ 60 F_{254}) were used for TLC analysis. The enantiomeric excess (% ee) of the products was determined by Agilent Technologies 1200 series chromatograph using appropriate chiral columns (see below). Flash column chromatography was performed on a Biotage® Isolera Prime with silica gel VWR (40–63 µm). Crystallographic analysis was performed on a Rigaku XtaLAB Synergy-S diffractometer, equipped with a PhotonJet-S CuKα (λ = 1.54184 Å) radiation source, and HyPix-Arc 100° detector. SPS grade THF, Et_2O, DMF and DCM were used to conduct the reactions. All other purchased solvents and chemicals were used as received. NMR yields were determined from ^{19}F spectra by addition of 0.5 equiv of an internal standard (1,4-bis(trifluoromethyl)benzene) to the crude reaction mixture. After the indicated reaction time (depending on the starting materials), an aliquot (40 µL) of the crude reaction mixture was taken out for ^{1}H and ^{19}F NMR analysis. NMR yields were calculated from the ratio of the corresponding integrals, corrected for the number of nuclei contributing to each signal. Racemic samples of compounds were prepared following the general procedures described below using **(±)-ITU1** (obtained by mixing the two pure enantiomers together). Therefore, a deviation from an expected 50:50 ratio of enantiomers in the HPLC is observed.

Caution! *Ultraviolet light is damaging to biological tissues. Caution is required when working with the LED lamp and corresponding protective eyewear must be used.*

Caution! *Diazo compounds can be highly unstable and potentially explosive, especially when exposed to heat or shock. Proper protective equipment must be worn, and all handling should be carried out with care under controlled conditions.*

2. Photochemical Setup

Setup A

The photochemical setup is shown in **Figure S1**. The photochemical experiments were conducted using LED lamps bought from www.led-tech.de (Cree XT-E Royal blue UV-LED lamp, with a manufacturer measured dominant wavelength range 457-460 nm; outtakes of the datasheets of the lamps are attached at the end of the SI).

The photoreactor (**Figure S1**) consists of the following units:

A) Photoreactor, with mirrors inside and two fans on the side walls

B) Light source, which consists of the three LED lamps

C) Controller

D) Magnetic stirrer.

Mirrors were installed to distribute irradiation to aim both at the bottom and the side of the reaction vials, whilst temperature control was managed with fans removing heat from the photoreactor (at the corresponding controller setting with cooling, t = 23 °C inside the photoreactor).

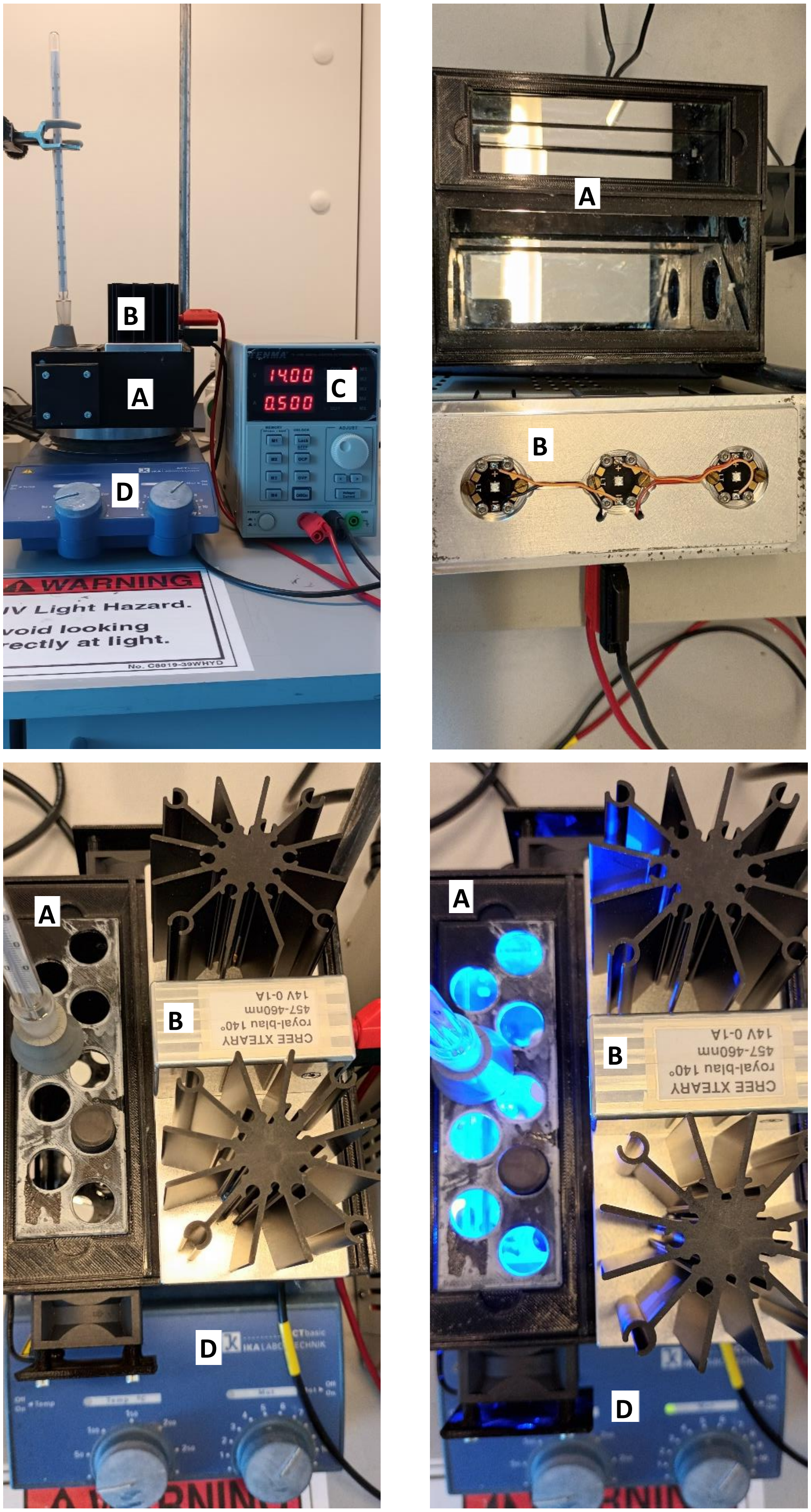

Figure S1. Photochemical setup with the photoreactor

Setup B

The photochemical setup is shown in **Figure S2**. A Blue LED lamp (ABI 23W Tuna Blue LED Bulb Coral Reef Optimized Spectrum 11-Band PAR38, with a manufacturer measured dominant wavelength range 453 nm;) was placed above a cooling bath at a height of 30 cm from the coolant. The reaction vessels were placed in the bath at an angle and inserted 1-1.5 cm into the bath (this ensured that if a layer of ice formed onto the vessel it was not covering the solvent in the vessel). The temperature of the reaction mixture was regulated with a chiller. The lamp was on during the indicated time.

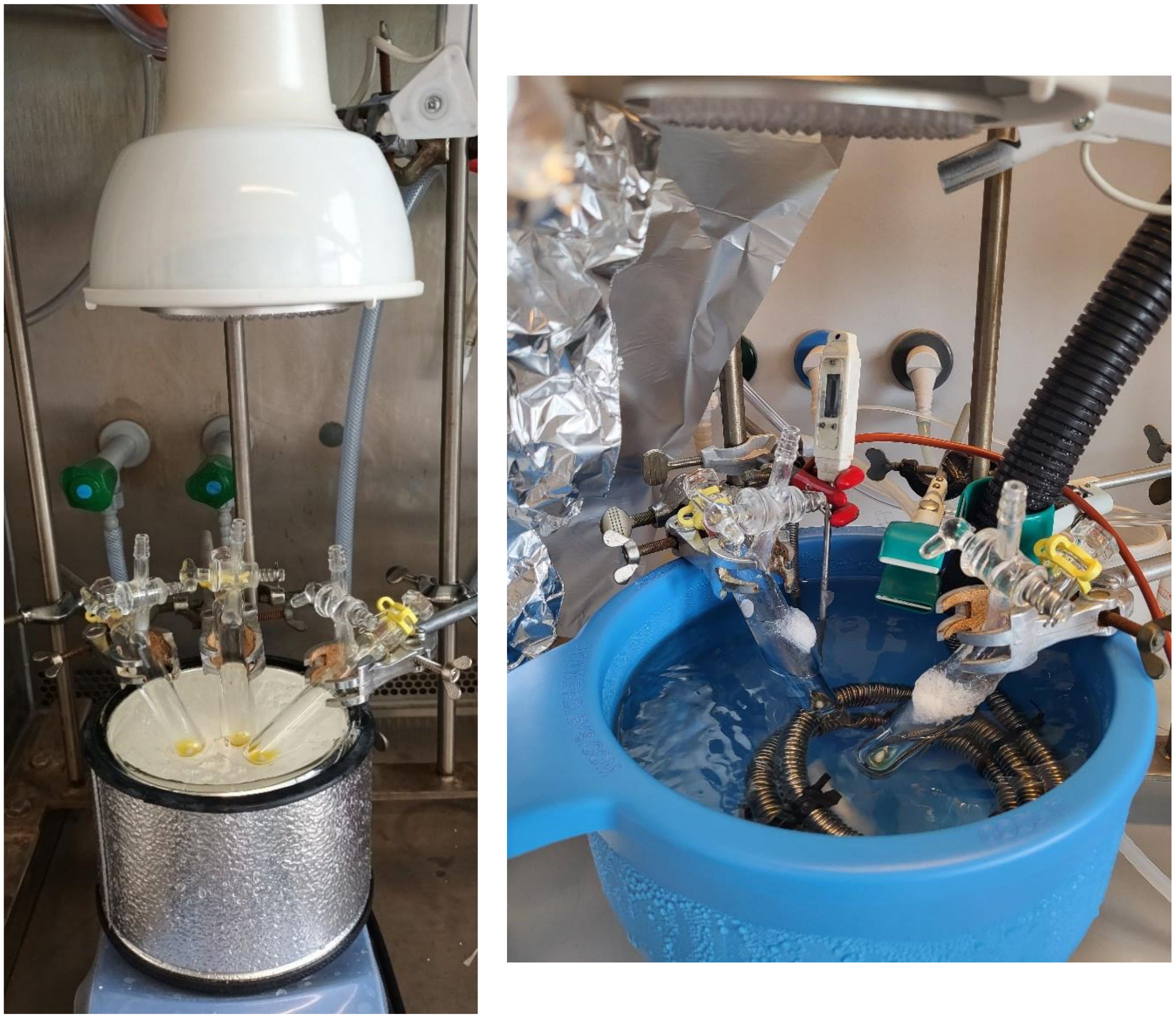

Figure S2. Setup of the photochemical reaction

3. Optimization of the reaction with both photochemical setups

Initial screening – setup A

Initial screening using tropone **1a** showed that product **4a** was formed under light irradiation without added catalyst. However, with the addition of possible nucleophilic catalysts represented in **Scheme S1**, no product was formed, except in the case of **ITU1** and NHC catalyst, which provided product **4a**, however in a racemic manner.

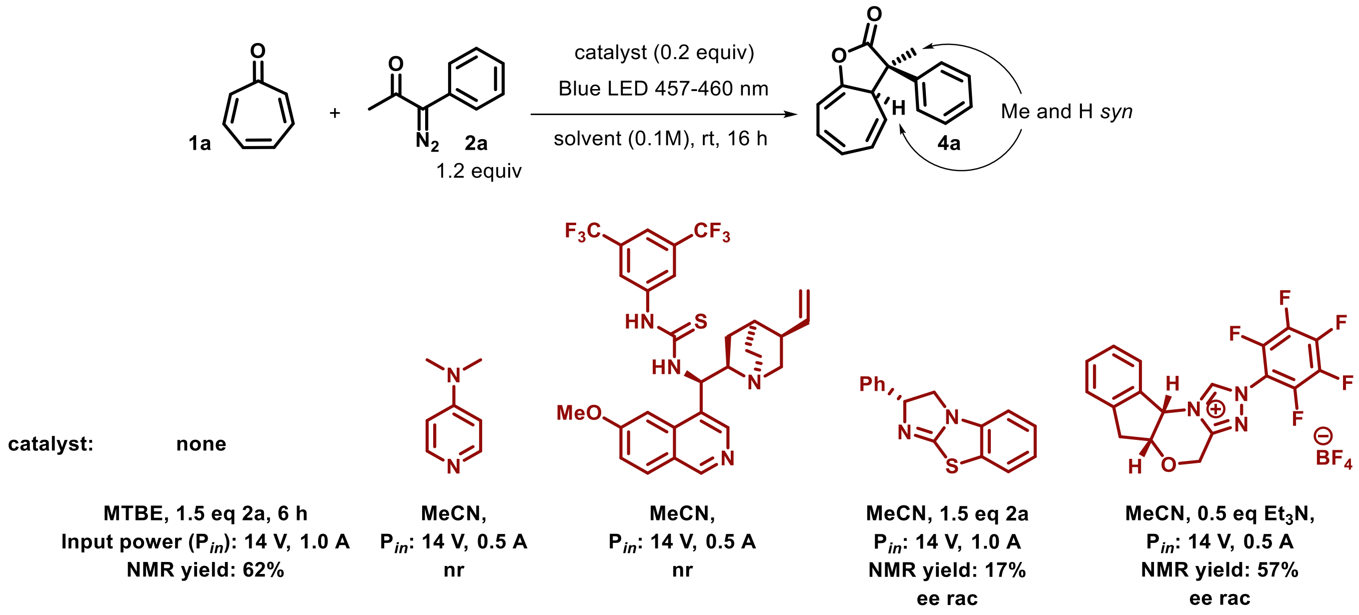

Scheme S1. Initial screening with tropone

In the case of triflate tropolone **1b**, no product was formed in the absence of a catalyst under light irradiation. The amines tested were largely inactive; only DMAP exhibited a reasonable level of reactivity, whereas its chiral analogue **C** showed low reactivity. Isothioureas proved to be promising catalysts, affording high conversions and up to moderate enantioselectivity, with catalyst **ITU1** providing the best result in terms of both enantio- and diastereoselectivity. Phosphine catalysts also showed some turnover; however, they gave overall lower conversions and selectivities compared to isothioureas (**Table S1**). Although **3a** is isolatable, we were unable to find conditions to determine its enantiomeric excess using UPCC or HPLC analysis due to its instability during the analysis. Therefore, in all cases ee was measured for **4a**.

Table S1. Initial screening with tropolone **1b**

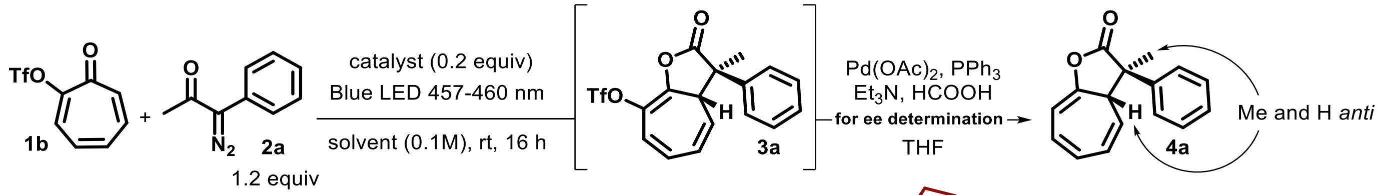

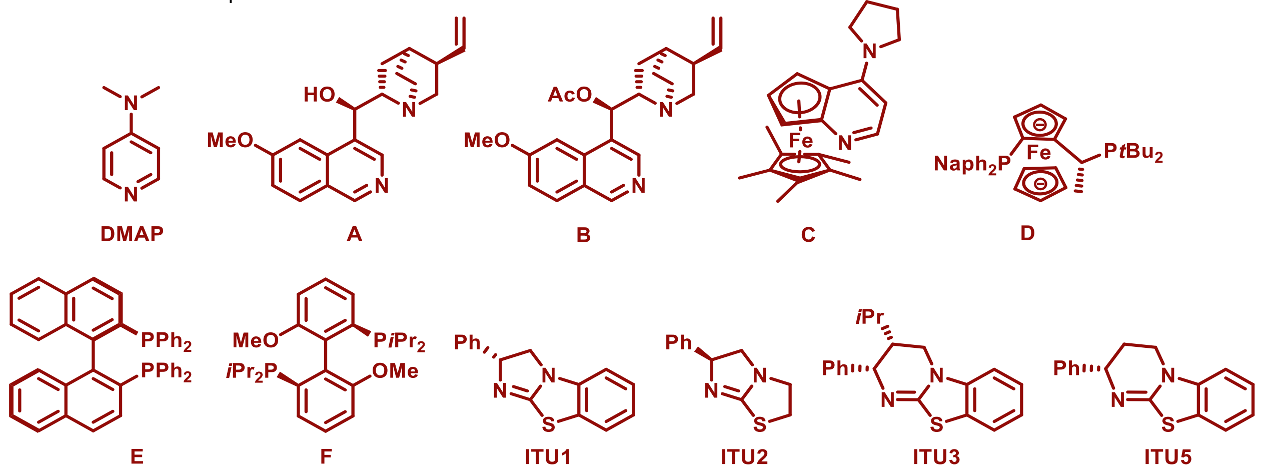

Nr.	Catalyst	Conditions	Input power (P_{in})	NMR quantity, % 1b	3a	d.r.	ee (4a), % Maj	Min
1	-	MeCN	V = 14 V, I = 1.0 A			nr		
2	**DMAP**	MeCN	V = 14 V, I = 1.0 A	21	47	3.3:1	-	
3	**ITU1**	MeCN	V = 14 V, I = 0.5 A	29	61	2.2:1	23	2
4	**A**	MeCN	V = 14 V, I = 0.5 A			nr		
5	**B**	MeCN	V = 14 V, I = 0.5 A			nr		
6	**C**	MeCN	V = 14 V, I = 0.1 A	66	7	2.4:1	nd	
7	**D**	THF	V = 14 V, I = 0.1 A	53	30	2.3:1	-8	9
8	**E**	THF	V = 14 V, I = 0.1 A	27	67	2.9:1	rac	10
9	**F**	THF	V = 14 V, I = 0.1 A	44	37	2.1:1	rac	rac
10	**ITU1**	MeCN	V = 14 V, I = 0.1 A	25	60	2.3:1	30	10
11	**ITU2**	MeCN	V = 14 V, I = 0.5 A	15	55	1.7:1	rac	12
12	**ITU3**	MeCN	V = 14 V, I = 0.1 A	8	80	1.2:1	18	19
13	**ITU5**	MeCN	V = 14 V, I = 0.1 A	13	75	1.4:1	8	20

The initial screening experiments were conducted in vials that were flushed with argon prior to the reaction. However, several inconsistencies in the results were observed occasionally. Control experiments revealed that the reaction is highly sensitive to air, with the NMR yield decreasing from 60% under an argon atmosphere to 15% under air (**Table S2**, entry 2). Additionally, the reaction is inhibited by the presence of acids or acidic additives, as demonstrated in entries 3-6 in **Table S2**. Water was also found to be detrimental, leading to reduced yield and enantiomeric excess (compare entries 1 and 6, **Table S2**). These key findings were considered when planning and setting up reactions using setup B.

Table S2. Control experiments with tropolone **1b**

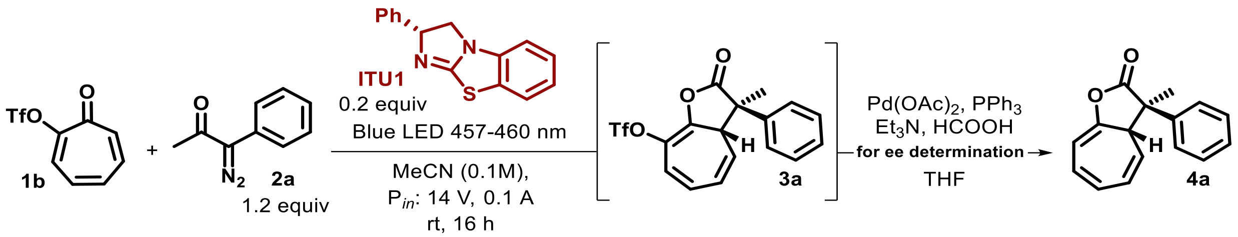

Nr.	Deviation from the standard conditions	NMR quantity, %		ee (**4a**), %	
		**1b**	**3a**	maj	min
1	-	25	60	30	10
2	Under air atmosphere	70	15	18	19
3	With added AcOH (20 mol%)	nr			
4	With added silicagel	61	27	nd	
5	With added crushed 3Å MS	45	46	nd	
6	MeCN containing 167 ppm of H_2O	35	52	19	rac

Additional screening – setup B

Upon deployment of setup B it was observed that the reaction performed significantly worse at rt compared to the initially explored conditions. This was partly due to the differences in the light sources (differences in dominant wavelengths and input power). Most notably, with setup B we were unable to maintain the reaction temperature at rt due to the heating effect of the lamp. Therefore, 0 °C was chosen as a starting point for the optimization experiments with setup B. Considering the differences in the experimental setups used, several catalysts were additionally rescreened with setup B, which still indicated **ITU1** as the most efficient catalyst for the studied reaction (**Table S3**, A). Further optimization experiments were conducted in the presence of **ITU1**.

Initial solvent screening at 0 °C with 0.2 equiv of catalyst revealed a pronounced solvent effect on both reaction efficiency and enantioselectivity. Polar solvents such as acetonitrile, acetone, and THF resulted in low yields of **3a** and poor enantioinduction, indicating that highly polar media are detrimental to effective asymmetric control in this transformation. Halogenated solvents showed moderate reactivity but did not significantly improve the enantioselectivity of the major diastereomer. In contrast, nonpolar solvents proved substantially more effective. Ethers and aromatic solvents afforded remarkably higher yields of **3a** together with improved enantiomeric excess. Among these, CPME and toluene consistently provided the best overall performance and were therefore selected for further optimization. Notably, reactions conducted in hexane delivered quite poor conversion to **3a**, however with very good enantioselectivity, underscoring the role of low-polarity environments in stabilizing the enantioselective transition state.

In the case of toluene, temperature was found to have only a minor influence on rate and stereochemical outcome of the reaction. However, in the case of CPME, reactions showed near-complete consumption of **1b** at lower temperatures albeit with no significant influence on the ee of the major diastereomer. In CPME, an optimal balance between yield and enantioselectivity was observed at −40 °C. The improved conversion at lower temperature is likely due to reduced instability of the substrate, intermediate, and/or product (see **Figures S3**, **S4**). Cooling therefore allows the desired reaction pathway to dominate by minimizing these unwanted processes.

Considering the sensitivity of ketene-based transformations to moisture, the effect of additives was next evaluated with particular emphasis on water control. Surprisingly, the addition of common drying agents (such as pre-dried Na_2SO_4 and $CaCl_2$), as well as activated molecular sieves, did not improve the reaction outcome in toluene and, in the case of CPME, even led to diminished reactivity without affecting the enantioselectivity. Moreover, reactions conducted in the presence of molecular sieves proved to be poorly reproducible. Most likely, mechanical degradation of the sieves during stirring led to acidification of the reaction environment to a different extent each time, thereby resulting in inconsistent and irreproducible outcomes.

To rule out the possible interference of the two sequential steps, control experiments employing pre-prepared ketene were conducted. Reactions in both toluene and CPME resulted in diminished conversion to product. In addition, in the case of CPME, the enantioselectivity was substantially reduced compared to reaction using *in situ* ketene generation. This finding indicates that *in situ* ketene formation is essential for achieving both high yield and effective asymmetric induction, as the gradual generation of the unstable ketene ensures its immediate participation in the cycloaddition step, thereby suppressing ketene degradation.

Overall, the optimal conditions for the asymmetric formation of **3a** were identified as reaction conducted in CPME, at reduced temperature (−40 °C), using 0.2 equiv of catalyst. These parameters provide the most favorable compromise between yield of **3a** and enantioselectivity of its major diastereomer and were therefore selected for subsequent investigations (**Table S3**, B).

Table S3. Additional optimization with setup B

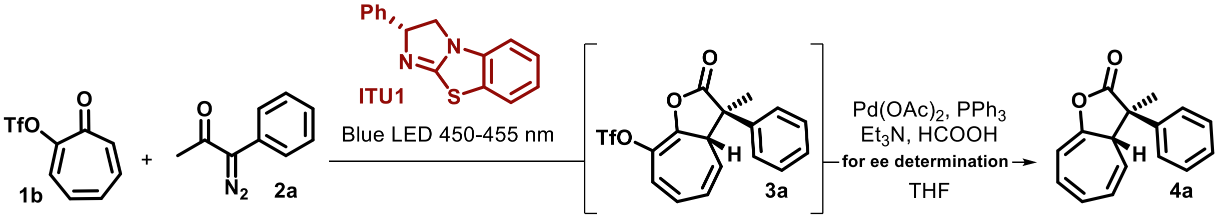

A: Catalyst screening

Reaction conditions: 0.05 mmol scale, **2a** (1.2 equiv), catalyst (0.2 equiv), CPME (0.1M), -40 °C, 6 h

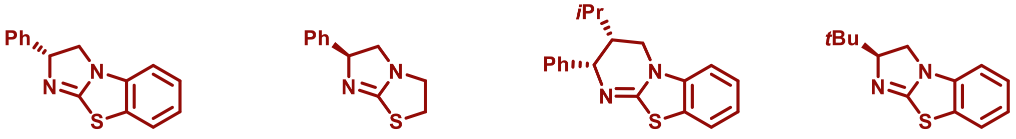

ITU1	ITU2	ITU3	ITU4
NMR yield: 99%	NMR yield: 60%	NMR yield: 76%	NMR yield: 62%
4:1 d.r.	0.9:1 d.r.	0.7:1 d.r.	1.1:1 d.r.
78%/53% ee	-26%/-54% ee	-10%/10% ee	-66%/-60% ee

B: Additional optimization

Reaction conditions: 0.05 mmol scale, **2a** (1.2 equiv), solvent (0.1M), additives, temperature, 6 h

Nr.	Solvent	Temp, °C	**ITU1**, equiv	Additives	NMR quantity, % **1b**	NMR quantity, % **3a**	d.r.	ee (**4a**), % maj	ee (**4a**), % min
1	MeCN	rt	0.2	-	69	12	3:1	nd	nd
2	Toluene	0	0.2	-	16	72	4.4:1	75	47
3	Et_2O	0	0.2	-	15	72	4.5:1	67	34
4	EtOAc	0	0.2	-	30	49	4.4:1	60	20
5	DCM	0	0.2	-	53	37	2.1:1	30	17
6	$CHCl_3$	0	0.2	-	79	10	4:1	47	35
7	THF	0	0.2	-	17	56	2.5:1	13	13
8	Acetone	0	0.2	-	37	39	3.3:1	37	8
9	MTBE	0	0.2	-	16	77	4.4:1	42	27
10	CPME	0	0.2	-	13	78	4.2:1	75	40
11	Hexane	-40	0.2	-	75	15	2.8:1	74	59
12	Hex:CPME	-40	0.2	-	15	63	3.8:1	75	55
13	Hex:Tol	-40	0.2	-	60	13	1.6:1	21	10
14	Toluene	-20	0.2	-	24	64	4.3:1	71	41
15	Toluene	-40	0.2	-	20	62	4.1:1	74	51
16	Toluene	0	0.1	-	38	54	4.4:1	62	27
17	Toluene	-20	0.1	-	35	54	4.4:1	61	49
18	Toluene	-40	0.1	-	55	32	3.6:1	66	55
19	Toluene	0	0.2	2 equiv of **2a***	2	87	4.1:1	68	36
20	Toluene	0	0.2	$CaCl_2$	20	71	4.5:1	74	45
21	Toluene	0	0.2	Na_2SO_4	18	73	4.2:1	78	53
22	Toluene	0	0.2	3Å MS	17	69	4.3:1	79	49
23	Toluene	0	0.2	4Å MS	22	64	4.3:1	77	48
24	Toluene	-20	0.2	3Å MS	13	71	4.5:1	79	51
25	Toluene	-40	0.2	3Å MS	13	74	3.9:1	81	50
26	CPME	-20	0.2	-	5	73	4.2:1	80	45
27	CPME	-40	0.2	-	<3	99	4:1	78	53
28	CPME	0	0.1	-	42	46	3.8:1	63	44
29	CPME	-40	0.1	-	25	66	3.4:1	71	47
30	CPME	-40	0.4	-	15	60	4.5:1	60	26
31	CPME	0	0.2	2 equiv of **2a***	1	84	4.1:1	59	35
32	CPME	0	0.2	$CaCl_2$	20	71	4.5:1	74	45
33	CPME	0	0.2	Na_2SO_4	18	73	4.2:1	78	46
34	CPME	0	0.2	3Å MS	19	54	4.4:1	76	52
35	CPME	-20	0.2	3Å MS	9	66	4.5:1	82	42
36	CPME	-40	0.2	3Å MS	11	18	3.5:1	77	31
37	Toluene	0	0.2	Pre-prepared ketene	nd	24	3.8:1	74	60
38	CPME	0	0.2	Pre-prepared ketene	86	14	2.5:1	30	16

* Both variations, addition of 2 equiv of diazo compound **2a** at once or sequential addition of 1 equiv at the beginning and another equiv after 6 h of reaction, provided the same result.

4. Reproducibility studies

As noted earlier, the reaction was found to be highly unpredictable when not set up thoroughly. Therefore, a broader range of experimental parameters were systematically examined again with setup B in Schlenk reactors, in order to distinguish between inconsequential variables and those significantly influencing the reaction outcome (**Table S4**).

Notably, several factors were found not to affect either the yield or enantioselectivity of product **3a**. These included the overall reaction concentration, the distance between the LED light source and the reaction vessel (within a 65 cm distance), the immersion depth of the reaction flask within the cooling bath (either ice or acetone), the batch of starting materials or catalyst and the stirring rate (except reactions conducted using molecular sieves). Furthermore, while varying these parameters no changes in the reaction outcome were detected, indicating that they are not responsible for the observed inconsistency.

In contrast, a number of parameters were identified as critical for reproducible reaction performance. Thorough dryness of the entire reaction setup proved essential, with trace moisture leading to diminished or unpredictable reactivity. The presence of dissolved oxygen in the solvent was also detrimental, and degassing was required to achieve consistent results. In contrast, attempts to improve reproducibility through additional solvent degassing via freeze-pump-thaw cycles did not result in any measurable benefit. This observation may be attributed to the sensitivity of the reaction to the order of reagent addition. Control experiments involving variations in the addition sequence revealed undesired reactivity between the triflate tropolone **1b** and the diazo compound **2a** (especially under neat conditions), which led to the decomposition of **2a** during reaction setup (see video). In addition, the LED source had a pronounced impact on the outcome, as evidenced by the change from setup A to setup B. Interestingly, periodically switching the LED source off and on to slow down ketene formation and thus reduce the accumulation of excess ketene in solution only capable to participate in undesired side reactions, resulted in an even poorer outcome. Unexpectedly, the material of the Schlenk stopcock was found to be decisive: reactions conducted in vessels equipped with glass stopcocks proceeded reliably, whereas those using PTFE stopcocks consistently failed, even when performed under an argon atmosphere maintained with an argon-filled balloon.

Table S4. Control experiments evaluating procedural and setup parameters

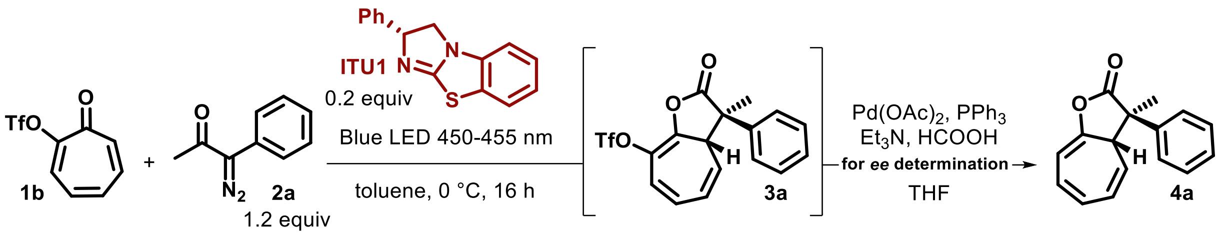

Nr.	Deviation from the standard conditions	NMR yield (**3a**), %	ee (**4a**), % maj	ee (**4a**), % min
1	-	72	75	47
2	Position of the Schlenk:<br>Height from the LED source (up to 65 cm)<br>Depth into cooling bath (up to 3 cm)	72	72	42
3	Step-by-step, with isolation of **3a**	69	73	47
4	Without solvent degassing	34	46	33
5	Freeze-pump-thaw	61	41	22
6	Vial, argon	75	74	44
7	Vial, air	25	nd	nd
8	CPME, -40 °C, 6 h	99	78	50
9	CPME, -40 °C, 6 h, LED source on-off	52	62	48
10	CPME, -40 °C, 6 h, no light and in the dark	0	-	-

Diastereomeric ratio was not significantly affected by changes in the reaction conditions.

Taken together, the identification of these critical parameters enabled the establishment of a robust and reproducible protocol, while also rationalizing the inconsistencies observed during early optimization studies.

5. Ketene formation correlation with reaction kinetics

To gain more detailed insight into the reaction, the rate of ketene formation was investigated. The two most successful solvents for this reaction, toluene (**Figure S3**, A) and CPME (**Figure S3**, B), were selected. In both solvents, reactions were conducted at room temperature, −20 °C, and −40 °C. All experiments were performed twice and in two parallels to minimize experimental errors: (1) as five separate reactions (setup as larger batch, divided into five vessels), each quenched at a specific reaction time followed by the addition of an internal standard; and (2) as a single larger batch containing the internal standard from the beginning.

In both solvents, ketene formation proceeded more rapidly and favorably at lower temperatures, leading to higher conversions. However, in all cases the maximum theoretical NMR yield was not reached, with the highest observed yield being approximately 50% after 3 h. Thereafter, gradual ketene depletion was observed, ultimately resulting in complete degradation after 9 h.

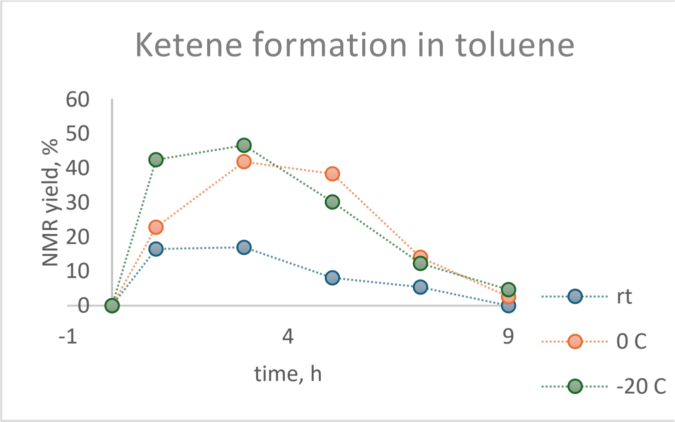

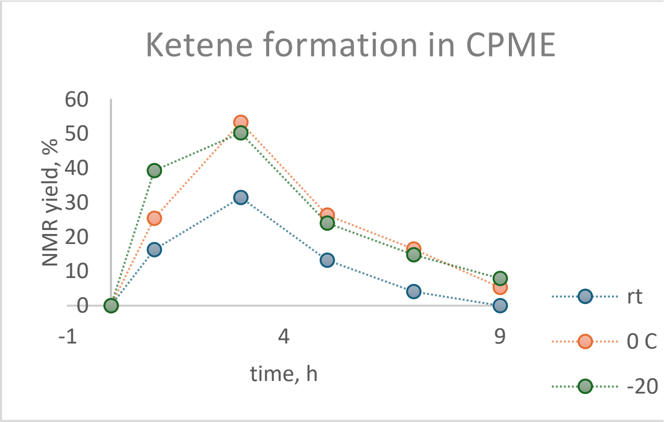

Figure S3. Ketene formation in toluene (A) and CPME (B) at rt, 0 °C and -20 °C

Subsequently, the profile of the one-pot reaction under optimal conditions was monitored (**Figure S4**). Full conversion was achieved already within 3 h. After this point, gradual decomposition of the product was observed upon further exposure to the reaction medium. This finding indicates that (a) ketene formation is faster than previously thought, and its reactivity in side reactions is correspondingly high (b) precise determination of the optimal reaction time is crucial for achieving the best possible outcome.

As optimal reaction time may vary significantly between substrates, this observation suggests that improved outcomes may be achieved through substrate-specific reoptimization of the reaction conditions. In addition, technical aspects of the photochemical setup could be explored further, *e.g.* varying the photon flux to influence the speed of ketene formation depending on the diazoketone absorbance wavelength. Nevertheless, it is important to note that this cascade process involves multiple bond cleavages and formations, enabling the construction of a structurally complex chiral product from simple starting materials under operationally straightforward conditions.

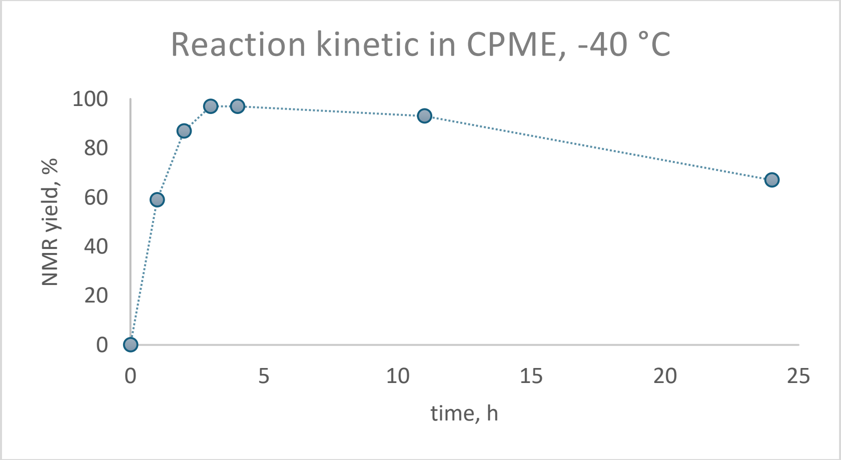

Figure S4. NMR yield profile of product **3a** over time

6. Additional scope examples and limitations

Cycloaddition product can also be obtained from *p*-methoxy-substituted diazo compound (**Scheme S2**, **3m**). However, due to the instability of the corresponding diazo compound, the reaction afforded a messy mixture with low enantioselectivity. The diazo compound bearing *p*-nitro substitution on phenyl ring appeared to be poorly soluble in the solvents optimal for the reaction (**Scheme S2**, **3n**). Screening of different temperatures did not improve the situation, which ultimately led to very low conversion. For **3o**, the lower conversion can be the result of the electron donating effect of the isopropyl group rather than its steric hindrance (**Scheme S2**, **3o**).

A range of additional diazo ketones were also evaluated. However, these substrates exhibited lower or no reactivity under the reaction conditions. Diazoketones bearing methyl or hydrogen substitution (**2v** and **2w**), rather than aromatic groups, exhibited very limited reactivity, likely due to their reduced stability. Steric effects also appeared to play a significant role, which may account for the suppressed reactivity of **2q**. In the case of compound **2r**, excitation was not achieved under the applied irradiation wavelength. Heteroaromatic substitution likewise proved incompatible, as evidenced by compound **2t**, where the additional nitrogen atom in the pyridine ring likely competes with the **ITU1** catalyst. Furthermore, the five-membered cyclic diazo compound **2p** did not undergo reaction, possibly because formation of the corresponding four-membered ring product is too strained to occur.

Several additional tropolone derivatives were also investigated. Among these, the limited reactivity of the nitroso-substituted analogue **1e** can be attributed to its poor solubility. Neither elevated temperature nor the use of alternative solvents led to any improvement in the outcome. Compounds **1f** and **1g** were found to be insufficiently reactive and therefore did not undergo the reaction.

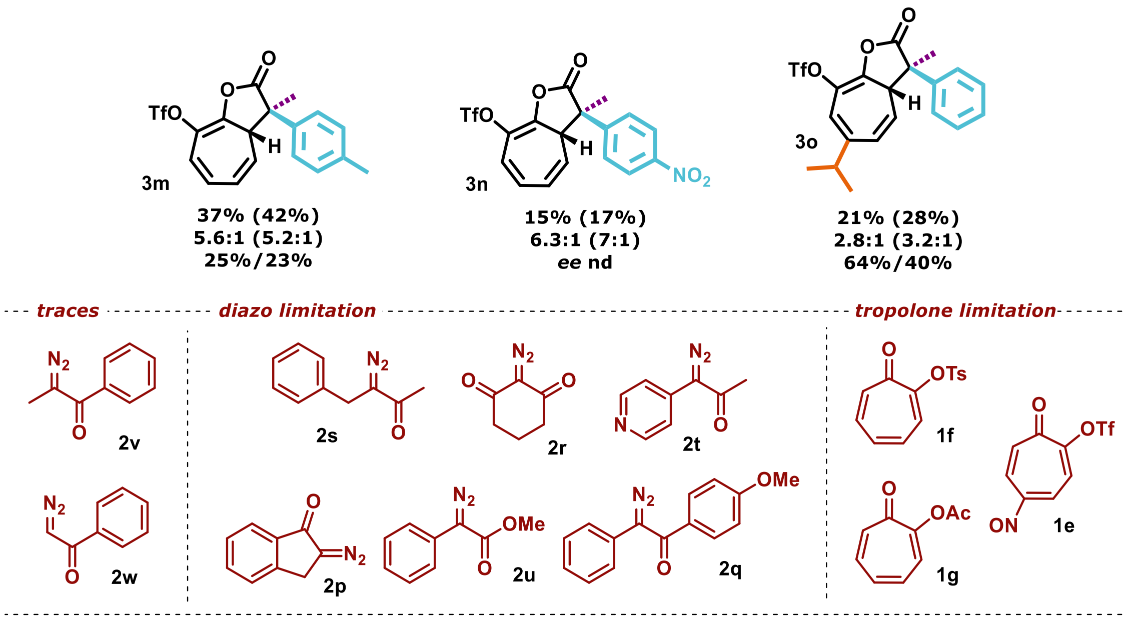

Scheme S2. Additional scope examples and limitations

7. Experimental part

Synthesis of the starting materials

Synthesis of tropolone derivatives **1**

Synthesis of tropolone derivatives **1** was performed according to the published chemical procedures.

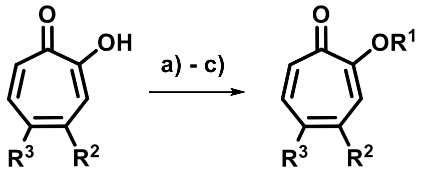

a) Tropolone (1 equiv), Tf_2O (1.5 equiv), pyridine (1.2 equiv), DCM (0.25 M), 0 °C, 1.5 h.[1]

1b[2]: R^1 = Tf, R^2/R^3 = H – white crystals (3.2 g, 54% yield)

^{1}H NMR (400 MHz, $CDCl_3$) δ 7.38 (dd, *J* = 10.4, 1.0 Hz, 1H), 7.36 (dd, *J* = 5.2, 0.9 Hz, 1H), 7.36 (dd, *J* = 5.1, 1.0 Hz, 1H), 7.22 (dddd, *J* = 10.1, 5.1, 4.4, 0.8 Hz, 1H), 7.04 (ddt, *J* = 11.0, 9.4, 0.9 Hz, 1H). **^{13}C NMR** (101 MHz, $CDCl_3$) δ 178.3, 156.4, 141.3, 137.3, 136.2, 130.5, 128.3, 118.8 (q, *J* = 320.3 Hz). **^{19}F NMR** (376 MHz, $CDCl_3$) δ -74.3.

1c*: R^1 = Tf, R^2 = H, R^3 = Br – brown solid (76 mg, 76% yield)

^{1}H NMR (400 MHz, $CDCl_3$) δ 7.55 (dd, *J* = 13.0, 2.0 Hz, 1H), 7.42 (dd, *J* = 10.3, 2.0 Hz, 1H), 7.13 (d, *J* = 13.0 Hz, 1H), 7.12 (d, *J* = 10.3 Hz, 1H). **^{13}C NMR** (101 MHz, $CDCl_3$) δ 177.4, 155.2, 141.6, 140.1, 133.0, 132.1, 127.3, 118.7 (q, *J* = 320.5 Hz).

1d[3]: R^1 = Tf, R^2 = *i*Pr, R^3 = H – yellow oil (428 mg, 72% yield, 1:1 inseparable mixture of regioisomers)

^{1}H NMR (400 MHz, C_6D_6) δ 7.27 – 7.21 (m, 5H), 7.13 (ddd, *J* = 11.4, 1.6, 0.8 Hz, 1H), 7.07 (ddd, *J* = 8.4, 2.3, 1.4 Hz, 1H), 6.97 (dd, J = 11.4, 9.5 Hz, 1H), 2.86 (h, *J* = 6.8 Hz, 1H), 2.84 (h, *J* = 6.8 Hz, 1H), 1.27 (d, *J* = 6.9 Hz, 6H), 1.26 (d, *J* = 6.8 Hz, 1H). **^{13}C NMR** (101 MHz, C_6D_6) δ 178.0, 177.7, 158.7, 155.93, 155.88, 152.3, 139.0, 138.2, 137.87, 137.85, 131.9, 129.9, 129.3, 127.6, 118.8 (q, *J* = 320.2 Hz), 118.8 (q, *J* = 320.2 Hz), 38.8, 38.2, 22.9, 22.8.

1e: R^1 = Tf, R^2 = H, R^3 = NO

Note! Compound **1e** is highly sensitive to both acids and moisture. Although the synthesis can be performed in DCM, chlorinated solvents should be avoided in all subsequent manipulations. Therefore, purification of **1e** was carried out using a modified procedure.

Upon completion of the reaction, water was added and the layers were separated. The aqueous phase was extracted with EtOAc (3 × 15 mL). The combined organic extracts were concentrated under reduced pressure and the residue was rapidly purified by flash column chromatography (3%–10% EtOAc in PE). The resulting brown solid was triturated with hexane and after stirring for 5 min, the solvent was removed. This washing step was repeated twice more to afford the corresponding tropolone derivative **1e** (340 mg, 36% yield) as a dark orange amorphous solid.

^{1}H NMR (400 MHz, C_6D_6) δ 6.03 (dd, *J* = 12.9, 1.6 Hz, 1H), 5.76 – 5.68 (m, 2H), 5.54 (d, *J* = 12.9 Hz, 1H). **^{13}C NMR** (101 MHz, C_6D_6) δ 184.8, 183.9, 158.5, 135.0, 133.7, 131.7, 120.7, 119.1 (q, *J* = 320.3 Hz). ^{19}F NMR (376 MHz, C_6D_6) δ -71.40.

b) Tropolone (1 equiv), TosCl (1.2 equiv), Et_3N (1.2 equiv), DCM (0.3 M), 0 °C to rt, 21 h.[4]

1f: R^1 = Tos, R^2/R^3 = H – white solid (697 mg, quantitative yield)

^{1}H NMR (400 MHz, $CDCl_3$) δ 7.95 – 7.89 (m, 2H), 7.44 (dd, *J* = 9.3, 0.9 Hz, 1H), 7.37 – 7.31 (m, 2H), 7.20 (ddd, *J* = 12.3, 7.8, 1.2 Hz, 1H), 7.13 (dt, *J* = 12.2, 1.1 Hz, 1H), 7.08 (ddt, *J* = 10.9, 7.8, 1.1 Hz, 1H), 6.97 (ddt, *J* = 10.8, 10.2, 1.0 Hz, 1H), 2.44 (s, 3H). **^{13}C NMR** (101 MHz, $CDCl_3$) δ 179.5, 155.3, 145.6, 141.4, 136.5, 134.7, 133.5, 130.9, 130.1, 129.7 (2C), 128.7 (2C), 21.9.

c) Tropolone (1 equiv), AcCl (1.2 equiv), pyridine (1.2 equiv), DCM (0.2 M), rt, 1 h.[5]

1g: R^1 = Ac, R^2/R^3 = H – white solid (143 mg, 93% yield)

^{1}H NMR (400 MHz, $CDCl_3$) δ 7.24 – 7.19 (m, 2H), 7.17 – 7.10 (m, 2H), 7.09 – 7.03 (m, 1H), 2.35 (s, 3H). **^{13}C NMR** (101 MHz, $CDCl_3$) δ 168.3, 134.1 (b), 133.6, 20.8. Due to a rapid acyl shift, some carbons cannot be observed in the ^{13}C NMR spectrum.[5]

* Preparation of the precursor to compound **1c** was carried out according to the published synthetic scheme:[6]

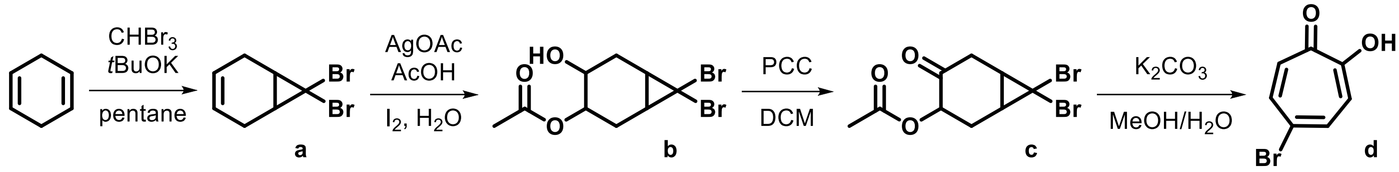

a: ^{1}H NMR (400 MHz, $CDCl_3$) δ 5.51 (bs, 2H), 2.55 – 2.40 (m, 2H), 2.16 – 2.05 (m, 2H), 1.96 – 1.87 (m, 2H).

b: ^{1}H NMR (400 MHz, $CDCl_3$) δ 4.80 (td, *J* = 6.4, 3.3 Hz, 1H), 3.80 (td, *J* = 6.7, 3.2 Hz, 1H), 2.50 – 2.32 (m, 2H), 2.07 (s, 3H), 1.96 – 1.80 (m, 4H), 1.74 (ddd, *J* = 15.3, 7.3, 3.8 Hz, 1H).

c: ^{1}H NMR (400 MHz, $CDCl_3$) δ 5.14 (ddd, *J* = 13.1, 7.5, 0.8 Hz, 1H), 2.91 – 2.72 (m, 3H), 2.33 – 2.24 (m, 1H), 2.22 – 2.15 (m, 1H), 2.15 (s, 3H), 2.04 (ddd, *J* = 14.2, 13.1, 4.6 Hz, 1H).

d: ^{1}H NMR (400 MHz, $CDCl_3$) δ 7.68 (d, *J* = 1.5 Hz, 1H), 7.65 (d, *J* = 1.6 Hz, 1H), 7.12 (d, *J* = 1.5 Hz, 1H), 7.10 (d, *J* = 1.6 Hz, 1H). **^{13}C NMR** (101 MHz, $CDCl_3$) δ 171.4 (2C), 140.6 (2C), 123.4 (2C), 123.1.

Synthesis of diazoketones **2**

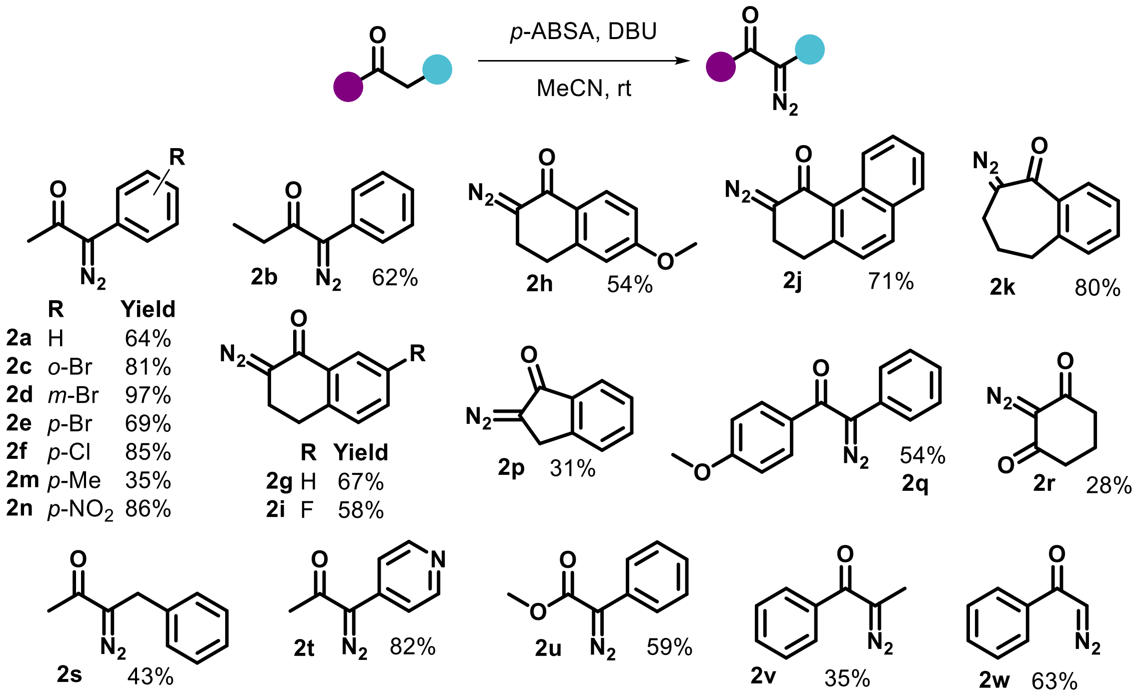

Synthesis of diazoketones **2** was performed according to the published chemical procedure.[7]

To a solution of corresponding ketone (1 equiv, 0.5 M) and *p*-ABSA (1.2 equiv) in dry MeCN (stored under Ar with 4Å MS) was added DBU (1.3 equiv) dropwise at 0 °C. Then the mixture was stirred at rt until full conversion was observed on TLC (typically 2 h). The reaction mixture was then concentrated under reduced pressure and purified directly by flash column chromatography (5%-20% EtOAc in PE) to afford the corresponding diazo compound **2**.

Note! For all diazo compounds **2**, the carbon associated with the diazo group was not observed in the ^{13}C spectrum.

2a: yellow crystals. **^{1}H NMR** (400 MHz, $CDCl_3$) δ 7.53 – 7.46 (m, 2H), 7.45 – 7.37 (m, 2H), 7.29 – 7.22 (m, 1H), 2.37 (s, 3H). **^{13}C NMR** (101 MHz, $CDCl_3$) δ 190.1, 129.2 (2C), 127.1, 126 (2C), 125.7, 27.1.

2b: yellow solid. **^{1}H NMR** (400 MHz, $CDCl_3$) δ 7.56 – 7.49 (m, 2H), 7.46 – 7.38 (m, 2H), 7.29 – 7.22 (m, 1H), 2.63 (q, J = 7.4 Hz, 2H), 1.21 (t, J = 7.4 Hz, 3H). **^{13}C NMR** (101 MHz, $CDCl_3$) δ 193.7, 129.2 (2C), 127, 126 (2C), 125.8, 32.6, 8.7.

2c: yellow solid. **^{1}H NMR** (400 MHz, $CDCl_3$) δ 7.56 – 7.48 (m, 2H), 7.43 – 7.35 (m, 2H), 2.38 (s, 3H).

2d: yellow solid. **^{1}H NMR** (400 MHz, $CDCl_3$) δ 7.70 (t, J = 1.8 Hz, 1H), 7.43 (dt, J = 8.0, 1.4 Hz, 1H), 7.37 (ddd, J = 8.0, 1.9, 1.1 Hz, 1H), 7.26 (t, J = 8.0 Hz, 1H), 2.38 (s, 3H). **^{13}C NMR** (101 MHz, $CDCl_3$) δ 189.5, 130.5, 130, 128.3, 127.9, 124, 123.2, 27.2.

2e: yellow solid. **^{1}H NMR** (400 MHz, $CDCl_3$) δ 7.56 – 7.48 (m, 2H), 7.42 – 7.36 (m, 2H), 2.38 (s, 3H). **^{13}C NMR** (101 MHz, $CDCl_3$) δ 189.7, 132.3 (2C), 127.1 (2C), 124.7, 120.7, 27.1.

2f: yellow solid. **^{1}H NMR** (400 MHz, $CDCl_3$) δ 7.50 – 7.42 (m, 2H), 7.41 – 7.34 (m, 2H), 2.38 (s, 3H). **^{13}C NMR** (101 MHz, $CDCl_3$) δ 189.6, 132.8, 129.4 (2C), 126.9 (2C), 124.1, 27.1.

2g: yellow solid. **^{1}H NMR** (400 MHz, $CDCl_3$) δ 8.01 (dd, *J* = 7.7, 1.5 Hz, 1H), 7.43 (td, *J* = 7.5, 1.5 Hz, 1H), 7.35 (td, *J* = 7.5, 1.3 Hz, 1H), 7.21 (ddd, *J* = 7.5, 1.5, 0.8 Hz, 1H), 3.07 – 3.01 (m, 2H), 3.01 – 2.96 (m, 2H). **^{13}C NMR** (101 MHz, $CDCl_3$) δ 183.6, 140.1, 133.3, 132.6, 128.2, 127.2, 126.0, 27.9, 20.8.

2h: red solid. **^{1}H NMR** (400 MHz, $CDCl_3$) δ 7.96 (d, *J* = 8.7 Hz, 1H), 6.84 (dd, *J* = 8.7, 2.6 Hz, 1H), 6.67 (d, *J* = 2.5 Hz, 1H), 3.83 (s, 3H), 3.03 – 2.92 (m, 4H). **^{13}C NMR** (101 MHz, $CDCl_3$) δ 183.1, 163, 142.6, 128.4, 126.8, 113.2, 112.7, 55.5, 28.3, 21.

2i: yellow solid. **^{1}H NMR** (400 MHz, $CDCl_3$) δ 7.68 (dd, *J* = 9.1, 2.8 Hz, 1H), 7.19 (dd, *J* = 8.4, 5.2 Hz, 1H), 7.12 (td, *J* = 8.2, 2.8 Hz, 1H), 3.03 – 2.96 (m, 4H). **^{13}C NMR** (101 MHz, $CDCl_3$) δ 182.6 (d, *J* = 2.2 Hz), 162.1 (d, *J* = 246.4 Hz), 135.8 (d, *J* = 3.1 Hz), 135.1 (d, *J* = 6.9 Hz), 130 (d, *J* = 7.3 Hz), 119.5 (d, *J* = 22.0 Hz), 112.7 (d, *J* = 22.8 Hz), 27.3, 21.

2j: Compound was isolated after recrystallization from a pentane/EtOAc solvent system in 71% yield as a yellow solid. **^{1}H NMR** (400 MHz, $CDCl_3$) δ 9.40 (dd, *J* = 8.9, 1.2 Hz, 1H), 7.90 (d, *J* = 8.3 Hz, 1H), 7.81 (dd, *J* = 8.1, 1.4 Hz, 1H), 7.61 (ddd, *J* = 8.6, 6.8, 1.5 Hz, 1H), 7.49 (ddd, *J* = 8.1, 6.8, 1.2 Hz, 1H), 7.29 (d, *J* = 8.3 Hz, 1H), 3.17 (t, *J* = 6.8 Hz, 2H), 2.98 (t, *J* = 6.8 Hz, 2H). **^{13}C NMR** (101 MHz, $CDCl_3$) δ 185.4, 141.8, 133.5, 133.3, 130.9, 128.6, 128.4, 128.2, 126.8, 126.6, 126.1, 30, 20.7. ^{19}F NMR (376 MHz, $CDCl_3$) δ -114.52 (td, *J* = 8.6, 5.1 Hz).

2k: yellow solid. **^{1}H NMR** (400 MHz, $CDCl_3$) δ 7.63 (dd, *J* = 7.5, 1.5 Hz, 1H), 7.40 (td, *J* = 7.5, 1.6 Hz, 1H), 7.33 (td, *J* = 7.5, 1.3 Hz, 1H), 7.16 (dd, *J* = 7.5, 1.7 Hz, 1H), 2.86 (t, *J* = 6.9 Hz, 2H), 2.45 (t, *J* = 6.9 Hz, 2H), 2.08 (p, *J* = 6.9 Hz, 2H). **^{13}C NMR** (101 MHz, $CDCl_3$) δ 192.5, 138.9, 138.9, 131.9, 129.3, 127.5, 127.2, 31.4, 28.5, 22.3.

2m: yellow amorphous solid. **^{1}H NMR** (400 MHz, $CDCl_3$) δ 7.36 (d, *J* = 8.0 Hz, 2H), 7.22 (d, *J* = 8.1 Hz, 2H), 2.36 (s, 3H), 2.34 (s, 3H). **^{13}C NMR** (101 MHz, $CDCl_3$) δ 206.9, 129.9 (2C), 129.8, 126.3 (2C), 123.7, 27, 21.2.

2n: yellow solid. **^{1}H NMR** (400 MHz, $CDCl_3$) δ 8.29 – 8.19 (m, 2H), 7.77 – 7.70 (m, 2H), 2.46 (s, 3H). **^{13}C NMR** (101 MHz, $CDCl_3$) δ 188.7, 145.7, 133.4, 124.5 (2C), 124.3 (2C), 27.3.

2p: yellow solid. **^{1}H NMR** (400 MHz, $CDCl_3$) δ 7.81 (dd, *J* = 8.0, 1.3 Hz, 1H), 7.59 (td, *J* = 7.4, 1.3 Hz, 1H), 7.48 – 7.40 (m, 2H), 4.09 (s, 2H). **^{13}C NMR** (101 MHz, $CDCl_3$) δ 188.7, 143.5, 137.7, 133.4, 128.1, 125.6, 123.1, 28.9.

2q: orange solid. **^{1}H NMR** (400 MHz, $CDCl_3$) δ 7.64 – 7.58 (m, 2H), 7.47 – 7.36 (m, 4H), 7.30 – 7.21 (m, 1H), 6.93 – 6.87 (m, 2H), 3.85 (s, 3H). **^{13}C NMR** (101 MHz, $CDCl_3$) δ 187.5, 162.6, 130.6, 130.2 (2C), 129.2 (2C), 127, 126.7, 126.3 (2C), 113.8 (2C), 55.6.

2r: dark yellow solid. **^{1}H NMR** (400 MHz, $CDCl_3$) δ 2.61 – 2.54 (m, 4H), 2.05 (tt, *J* = 7.2, 5.7 Hz, 2H). **^{13}C NMR** (101 MHz, $CDCl_3$) δ 190.5 (2C), 37 (2C), 18.8.

2s: yellow solid. **^{1}H NMR** (400 MHz, $CDCl_3$) δ 7.36 – 7.29 (m, 2H), 7.28 – 7.24 (m, 1H), 7.23 – 7.17 (m, 2H), 3.65 (s, 2H), 2.27 (s, 3H). **^{13}C NMR** (101 MHz, $CDCl_3$) δ 190.6, 137.0, 129 (2C), 128.7 (2C), 127.3, 28.7, 25.6.

2t: Compound was isolated in 78% yield as a yellow solid. **^{1}H NMR** (400 MHz, $CDCl_3$) δ 8.57 (d, *J* = 1.7 Hz, 1H), 8.55 (d, *J* = 1.7 Hz, 1H), 7.49 (d, *J* = 1.7 Hz, 1H), 7.48 (d, *J* = 1.7 Hz, 1H), 2.43 (s, 3H). **^{13}C NMR** (101 MHz, $CDCl_3$) δ 188.8, 149.9 (2C), 135.6, 118.4 (2C), 27.5.

2u: dark orange oil. **^{1}H NMR** (400 MHz, $CDCl_3$) δ 7.51 – 7.46 (m, 2H), 7.42 – 7.36 (m, 2H), 7.22 – 7.15 (m, 1H), 3.87 (s, 3H). **^{13}C NMR** (101 MHz, $CDCl_3$) δ 165.8, 129.1 (2C), 126, 125.6, 124.1 (2C), 52.1.

2v: yellow solid. **^{1}H NMR** (400 MHz, $CDCl_3$) δ 7.60 – 7.55 (m, 2H), 7.51 – 7.46 (m, 1H), 7.45 – 7.39 (m, 2H), 2.15 (s, 3H). **^{13}C NMR** (101 MHz, $CDCl_3$) δ 190.3, 137.8, 131.5, 128.7 (2C), 127.3 (2C), 9.7.

2w: yellow solid. **^{1}H NMR** (400 MHz, $CDCl_3$) δ 7.84 – 7.70 (m, 2H), 7.58 – 7.51 (m, 1H), 7.50 – 7.39 (m, 2H), 5.90 (s, 1H). **^{13}C NMR** (101 MHz, $CDCl_3$) δ 186.6, 137.1, 132.8, 128.54 (2C), 128.47 (2C).

General procedure for the photochemical enantioselective [8+2]-cycloadditions

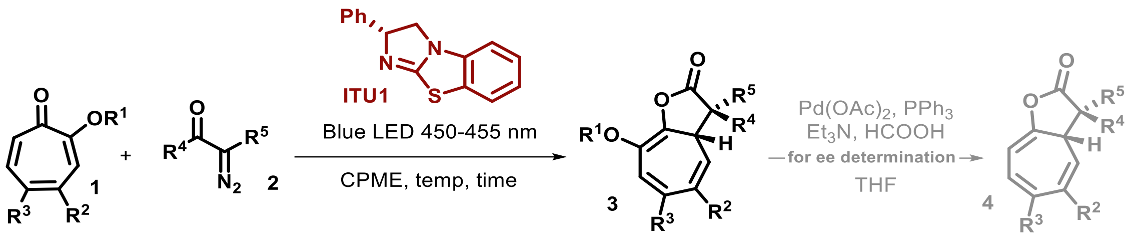

Step 1: A flame-dried Schlenk reactor was charged with tropolone derivative **1** (0.1 mmol, 1 equiv, 0.1 M) and catalyst **ITU1** (0.02 mmol, 0.2 equiv) under an argon atmosphere. Subsequently, 1 mL of degassed cyclopentyl methyl ether (CPME) (stored over 3Å molecular sieves under Ar atmosphere) was added using an argon-flushed syringe, ensuring the system remained under an inert atmosphere. The addition of the diazo compound **2** (0.12 mmol, 1.2 equiv) followed under inert conditions. The Schlenk joint was greased under an argon atmosphere to ensure an airtight seal. The reaction mixture was then placed in a cooling bath at corresponding temperature (using a chiller), and the reaction was initiated by irradiating with blue LED light (450–455 nm). The reaction proceeded under these conditions for 3-24 h, with continuous cooling and light exposure.

After the specified time, the presence of an overpressure of argon was verified by releasing gas from the Schlenk reactor. If no gas release was observed, the reaction result was excluded from consideration, as the maintenance of air-free conditions could not be confirmed. Subsequently, 4 µL of internal standard was added, and 40 µL of the reaction mixture was taken for NMR analysis. The remaining reaction mixture was transferred to a vial, and the solvent was removed using a rotary evaporator at 30 °C. The residue was purified by flash column chromatography (5%-35% DCM in PE) to afford product **3**.

Note! For all optimization experiments, products **3** were not isolated. After NMR analysis of the crude mixture, the remaining reaction mixture was transferred to a vial. Following solvent evaporation, Step 2 was performed directly.

Note! For all products **3**, the carbon associated with the triflate group was not observed in the ^{13}C spectrum.

Step 2 (applicable for each compound tested on a 0.1 mmol scale): 1.6 mL of a pre-prepared Pd solution was then added under an argon atmosphere. The Pd solution was prepared by combining $Pd(OAc)_2$ (4.7 mg, 0.21 equiv) catalyst and PPh_3 (11.6 mg, 0.42 equiv) in a flask under an argon atmosphere, followed by the addition of THF (1.8 mL), Et_3N (176 µL, 12.6 equiv), and HCOOH (39.6 µL, 10.5 equiv), resulting in a homogeneous yellow solution. The reaction mixture was stirred at 60 °C for 1 h, concentrated, and purified by flash column chromatography (5%-35% DCM in PE) to afford product **4** suitable for the HPLC analysis.

Isolation, identification and analysis of the model compound (3*R*,3a*S*)-3-methyl-2-oxo-3-phenyl-3,3a-dihydro-2*H*-cyclohepta[*b*]furan-8-yl trifluoromethanesulfonate (3a) and corresponding derivative 4a

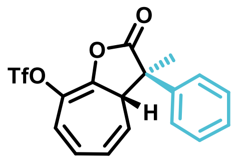

Synthesized on a 1.2 mmol scale from tropolone derivative **1b** (305 mg) and diazo compound **2a** (231 mg) using **ITU** (60.6 mg) in CPME (12 mL), following general procedure Step 1 at −40 °C for 6 h. The desired product **3a** was isolated as a colorless oil (404 mg, 87% yield, 3.8:1 d.r.).

HRMS (ESI$^+$) m/z: $[M + Na]^+$ Calcd. for $C_{17}H_{13}F_3O_5SNa$ 409.0328; Found: 409.0328.

Major diastereoisomer: **^{1}H NMR** (400 MHz, $CDCl_3$) δ 7.51 – 7.28 (m, 5H), 6.45 (dd, *J* = 11.9, 5.9 Hz, 1H), 6.34 (d, *J* = 11.9 Hz, 1H), 6.27 (ddd, *J* = 10.1, 5.9, 2.2 Hz, 1H), 5.56 (ddd, *J* = 10.1, 3.9, 0.9 Hz, 1H), 3.43 (dd, *J* = 3.9, 2.2 Hz, 1H), 1.88 (s, 3H). **^{13}C NMR** (101 MHz, $CDCl_3$) δ 175.3, 141.7, 137.3, 129.5 (2C), 128.8, 128.3, 127.8, 125.8, 125.3 (2C), 123.0, 121.2, 49.9, 46.9, 21.5. ^{19}F NMR (376 MHz, $CDCl_3$) δ -73.66.

Minor diastereoisomer: **^{1}H NMR** (400 MHz, $CDCl_3$) δ 7.51 – 7.28 (m, 5H), 6.45 (dd, *J* = 11.9, 5.7 Hz, 1H), 6.40 - 6.36 (m, 1H), 5.98 (ddd, *J* = 10.0, 5.7, 2.1 Hz, 1H), 4.57 (ddd, *J* = 10.0, 4.0, 0.9 Hz, 1H), 3.13 (dd, *J* = 4.0, 2.1 Hz, 1H), 1.87 (s, 3H). **^{13}C NMR** (101 MHz, $CDCl_3$) δ 175.4, 136.8, 136.2, 129.4 (2C), 128.4, 127.3 (2C), 126.5, 125.6, 123.4, 123.0, 122.6, 50.7, 47.0, 26.9. ^{19}F NMR (376 MHz, $CDCl_3$) δ -73.65.

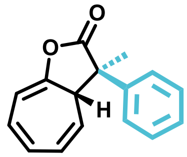

Synthesized from **3a** by following the general procedure Step 2 using 42 mL of Pd solution, pre-prepared using $Pd(OAc)_2$ (49.3 mg, 0.21 equiv), PPh_3 (115.2 mg, 0.42 equiv), Et_3N (1.84 mL, 12.6 equiv), HCOOH (414 µl, 10.5 equiv) and THF (47 mL). The desired product **4a** was isolated as a colorless oil (192 mg, 77% yield, 3.8:1 d.r.).

HRMS (ESI$^+$) m/z: $[M + Na]^+$ Calcd. for $C_{16}H_{14}O_2Na$ 261.0886; Found: 261.0888.

Major diastereoisomer: **^{1}H NMR** (400 MHz, $CDCl_3$) δ 7.37 – 7.33 (m, 4H), 7.31 – 7.27 (m, 1H), 6.40 – 6.30 (m, 2H), 6.26 – 6.18 (m, 1H), 6.05 – 5.99 (m, 1H), 5.40 (dd, *J* = 10.0, 3.9 Hz, 1H), 3.31 (dt, *J* = 3.9, 2.0 Hz, 1H), 1.84 (s, 3H). **^{13}C NMR** (101 MHz, $CDCl_3$) δ 178, 144.5, 143.0, 129.2 (2C), 127.8, 127.74, 127.65, 127.45, 125.4 (2C), 118.2, 101.9, 50.3, 49.2, 21.7.

HPLC 78% ee [Chiralcel AD-H column, hexane/*i*PrOH 98:2, flow rate 1 mL/min, 25 °C, λ = 280 nm; t_R (major) = 11.4 min and t_R (minor) = 10.4 min].

Minor diastereoisomer: **^{1}H NMR** (400 MHz, $CDCl_3$) δ 7.45 – 7.39 (m, 2H), 7.31 – 7.27 (m, 3H), 6.46 – 6.40 (m, 1H), 6.40 – 6.35 (m, 1H), 6.09 – 6.04 (m, 1H), 5.93 (ddd, *J* = 9.9, 5.8, 2.0 Hz, 1H), 4.39 (dd, *J* = 9.9, 4.1 Hz, 1H), 2.99 (dt, *J* = 3.9, 1.9 Hz, 1H), 1.84 (s, 3H). **^{13}C NMR** (101 MHz, $CDCl_3$) δ 178.5, 143.4, 138.0, 129.1 (2C), 127.9, 127.5, 127.49, 127.3 (2C), 126.4, 120.3, 101.7, 51.2, 49.6, 27.3.

HPLC 53% ee [Chiralcel AD-H column, hexane/*i*PrOH 98:2, flow rate 1 mL/min, 25 °C, λ = 280 nm; t_R (major) = 7.2 min and t_R (minor) = 8.2 min].

Isolation, identification and analysis of the (3*R*,3a*S*)-3-ethyl-2-oxo-3-phenyl-3,3a-dihydro-2*H*-cyclohepta[*b*]furan-8-yl trifluoromethanesulfonate (3b) and corresponding derivative 4b

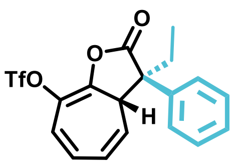

Synthesized from tropolone derivative **1b** (25.4 mg) and diazo compound **2b** (20.9 mg) by following the general procedure Step 1 at -40 °C within 6 h. The desired product **3b** was isolated as a colorless oil (26.8 mg, 67% yield, 3.2:1 d.r.).

HRMS (ESI$^+$) m/z: $[M + Na]^+$ Calcd. for $C_{18}H_{15}F_3O_5SNa$ 423.0484; Found: 423.0492.

Major diastereoisomer: **^{1}H NMR** (400 MHz, C_6D_6) δ 7.12 – 7.08 (m, 2H), 7.06 – 7.03 (m, 1H), 7.02 – 6.97 (m, 2H), 5.88 (d, *J* = 11.8 Hz, 1H), 5.74 (dd, *J* = 11.9, 6.0 Hz, 1H), 5.62 (ddd, *J* = 10.3, 6.1, 2.3 Hz, 1H), 4.88 (ddd, *J* = 10.3, 3.6, 0.9 Hz, 1H), 3.15 (dd, *J* = 3.5, 2.3 Hz, 1H), 1.73 (q, *J* = 7.3 Hz, 2H), 0.73 (t, *J* = 7.4 Hz, 3H). **^{13}C NMR** (101 MHz, C_6D_6) δ 173.2, 140.3, 139.5, 136.5, 129.23 (2C), 127.98, 127.56, 127.53, 126.1 (2C), 123.2, 120.9, 53.8, 45.4, 29.5, 9.0. ^{19}F NMR (376 MHz, $CDCl_3$) δ -73.64.

Minor diastereoisomer: **^{1}H NMR** (400 MHz, C_6D_6) δ 7.06 – 7.03 (m, 1H), 7.02 – 6.97 (m, 4H), 6.15 (d, *J* = 11.7 Hz, 1H), 5.96 – 5.88 (m, 1H), 5.42 (ddd, *J* = 9.7, 5.8, 2.0 Hz, 1H), 4.27 (ddd, *J* = 9.7, 4.5, 0.9 Hz, 1H), 2.44 (dd, *J* = 4.5, 2.0 Hz, 1H), 1.74 – 1.63 (m, 1H), 1.53 (q, *J* = 7.2 Hz, 1H), 0.66 (t, *J* = 7.4 Hz, 3H). **^{13}C NMR** (101 MHz, C_6D_6) δ 173.5, 141.5, 140.4, 135.3, 129.19, 129.16 (2C), 127.94 (2C), 125.9, 125.7, 123.0, 122.7, 54.9, 45.8, 34.8, 8.6. ^{19}F NMR (376 MHz, $CDCl_3$) δ -73.69.

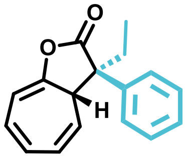

Synthesized from **3b** by following the general procedure Step 2. The desired product **4b** was isolated as a colorless oil (10 mg, 66% yield, 2.9:1 d.r.).

HRMS (ESI$^+$) m/z: $[M + Na]^+$ Calcd. for $C_{17}H_{16}O_2Na$ 275.1043; Found: 275.1047.

Major diastereoisomer: **^{1}H NMR** (400 MHz, $CDCl_3$) δ 7.44 – 7.27 (m, 5H), 6.39 – 6.29 (m, 2H), 6.27 – 6.19 (m, 1H), 5.95 – 5.91 (m, 1H), 5.45 (dd, *J* = 10.0, 3.9 Hz, 1H), 3.49 (dt, *J* = 3.9, 2.0 Hz, 1H), 2.35 (dq, *J* = 14.6, 7.3 Hz, 1H), 2.23 (dq, *J* = 14.6, 7.4 Hz, 1H), 1.00 (t, *J* = 7.4 Hz, 3H). **^{13}C NMR** (101 MHz, $CDCl_3$) δ 176.7, 145.3, 140.8, 129.1 (2C), 127.9, 127.77, 127.74 (2C), 126.2 (2C), 118.1, 101.3, 54.2, 47.6, 28.8, 9.3.

HPLC 61% ee [Chiralcel AD-H column, hexane/*i*PrOH 99:1, flow rate 1 mL/min, 25 °C, λ = 280 nm; t_R (major) = 13.2 min and t_R (minor) = 11.0 min].

Minor diastereoisomer: **^{1}H NMR** (400 MHz, $CDCl_3$) δ 7.44 – 7.27 (m, 5H), 6.50 (dd, *J* = 11.1, 6.2 Hz, 1H), 6.46 – 6.38 (m, 1H), 6.06 – 5.99 (m, 1H), 6.00 – 5.95 (m, 1H), 4.43 (dd, *J* = 9.6, 4.5 Hz, 1H), 2.88 – 2.77 (m, 1H), 2.28 - 2.19 (m, 1H), 2.19 - 2.13 (m, 1H), 1.02 - 0.96 (m, 3H). **^{13}C NMR** (101 MHz, $CDCl_3$) δ 176.8, 142.7, 137.5, 129 (2C), 127.79, 127.66, 127.4, 127.1 (2C), 126.0, 119.9, 100.6, 55.5, 48.3, 35.1, 9.0.

HPLC 53% ee [Chiralcel AD-H column, hexane/*i*PrOH 99:1, flow rate 1 mL/min, 25 °C, λ = 280 nm; t_R (major) = 6.9 min and t_R (minor) = 7.7 min].

(3*R*,3a*S*)-3-(2-Bromophenyl)-3-methyl-2-oxo-3,3a-dihydro-2*H*-cyclohepta[*b*]furan-8-yl trifluoromethanesulfonate (3c)

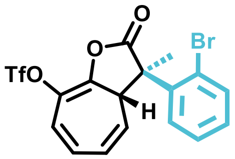

Synthesized from tropolone derivative **1b** (25.4 mg) and diazo compound **2c** (28.7 mg) by following the general procedure Step 1 at -40 °C within 6 h. The desired product **3c** was isolated as a yellow oil (27 mg, 58% yield, 3.1:1 d.r.).

HRMS (ESI$^+$) m/z: $[M + Na]^+$ Calcd. for $C_{17}H_{12}BrF_3O_5SNa$ 486.9433; Found: 486.9428.

Major diastereoisomer: **^{1}H NMR** (400 MHz, C_6D_6) δ 7.10 – 7.06 (m, 2H), 6.65 – 6.58 (m, 2H), 5.92 (d, *J* = 11.9 Hz, 1H), 5.77 (dd, *J* = 11.9, 6.1 Hz, 1H), 5.60 (ddd, *J* = 10.3, 6.1, 2.2 Hz, 1H), 4.68 (ddd, *J* = 10.3, 3.7, 0.9 Hz, 1H), 2.86 (dd, *J* = 3.7, 2.2 Hz, 1H), 1.13 (s, 3H). **^{13}C NMR** (101 MHz, C_6D_6) δ 174.0, 140.5, 138.0, 132.43 (2C), 127.9, 127.6, 127.3 (2C), 126.1, 122.84, 122.77, 120.8, 49.3, 46.4, 21.1. ^{19}F NMR (376 MHz, C_6D_6) δ -73.97.

HPLC of **4c** 76% ee [Chiralcel AD-H column, hexane/*i*PrOH 99:1, flow rate 1 mL/min, 25 °C, λ = 280 nm; t_R (major) = 22.6 min and t_R (minor) = 20.6 min].

Minor diastereoisomer: **^{1}H NMR** (400 MHz, C_6D_6) δ 7.14 – 7.11 (m, 2H), 6.65 – 6.58 (m, 2H), 6.04 (d, *J* = 11.8 Hz, 1H), 5.83 (dd, *J* = 11.8, 5.9 Hz, 1H), 5.40 (ddd, *J* = 10.0, 5.9, 2.1 Hz, 1H), 4.08 (ddd, *J* = 10.0, 4.0, 0.9 Hz, 1H), 2.43 (dd, *J* = 4.0, 2.1 Hz, 1H), 1.07 (s, 3H). **^{13}C NMR** (101 MHz, C_6D_6) δ 174.2, 136.0, 132.9, 132.37 (2C), 129.2 (2C), 128.9, 126.5, 125.7, 122.6, 122.5, 122.3, 49.9, 46.6, 26.2. ^{19}F NMR (376 MHz, C_6D_6) δ -73.97.

HPLC of **4c** 9% ee [Chiralcel AD-H column, hexane/*i*PrOH 99:1, flow rate 1 mL/min, 25 °C, λ = 280 nm; t_R (major) = 14.2 min and t_R (minor) = 11.0 min].

(3*R*,3a*S*)-3-(3-Bromophenyl)-3-methyl-2-oxo-3,3a-dihydro-2*H*-cyclohepta[*b*]furan-8-yl trifluoromethanesulfonate (3d)

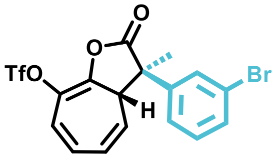

Synthesized from tropolone derivative **1b** (25.4 mg) and diazo compound **2d** (28.7 mg) by following the general procedure Step 1 at -40 °C within 6 h. The desired product **3d** was isolated as a yellow oil (28.8 mg, 62% yield, 1.9:1 d.r.).

HRMS (ESI$^+$) m/z: $[M + Na]^+$ Calcd. for $C_{17}H_{12}BrF_3O_5SNa$ 486.9433; Found: 486.9427.

Major diastereoisomer: **^{1}H NMR** (400 MHz, C_6D_6) δ 7.30 (t, *J* = 1.9 Hz, 1H), 7.13 – 7.06 (m, 1H), 6.87 – 6.81 (m, 1H), 6.60 (t, *J* = 8.0 Hz, 1H), 5.87 (d, *J* = 11.9 Hz, 1H), 5.73 (dd, *J* = 11.9, 6.1 Hz, 1H), 5.56 (ddd, *J* = 10.3, 6.1, 2.3 Hz, 1H), 4.62 (ddd, *J* = 10.3, 3.6, 0.9 Hz, 1H), 2.87 (dd, *J* = 3.6, 2.3 Hz, 1H), 1.08 (s, 3H). **^{13}C NMR** (101 MHz, C_6D_6) δ 173.8, 143.7, 138, 131.4, 130.9, 128.7, 127.9, 127.7, 126.5, 124.4, 123.5, 122.7, 120.6, 49.4, 46.2, 21.2. ^{19}F NMR (376 MHz, C_6D_6) δ -73.97.

Minor diastereoisomer: **^{1}H NMR** (400 MHz, C_6D_6) δ 7.35 (t, *J* = 1.9 Hz, 1H), 7.13 – 7.06 (m, 1H), 6.87 – 6.81 (m, 1H), 6.62 (t, *J* = 7.9 Hz, 1H), 6.02 (d, *J* = 11.8 Hz, 1H), 5.81 (dd, *J* = 11.8, 5.9 Hz, 1H), 5.35 (ddd, *J* = 10.0, 5.9, 2.1 Hz, 1H), 4.09 (ddd, *J* = 10.0, 4.1, 0.9 Hz, 1H), 2.39 (dd, *J* = 4.1, 2.1 Hz, 1H), 1.02 (s, 3H).**^{13}C NMR** (101 MHz, C_6D_6) δ 174.1, 139.4, 136.0, 131.5, 130.8, 130.3, 129.0, 126.4, 126.2, 125.7, 123.6, 122.65, 122.55, 50.0, 46.6, 26.2. ^{19}F NMR (376 MHz, C_6D_6) δ -73.94.

(3*R*,3a*S*)-3-(4-Bromophenyl)-3-methyl-2-oxo-3,3a-dihydro-2*H*-cyclohepta[*b*]furan-8-yl trifluoromethanesulfonate (3e)

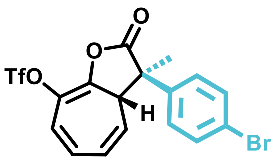

Synthesized from tropolone derivative **1b** (25.4 mg) and diazo compound **2e** (28.7 mg) by following the general procedure Step 1 at -40 °C within 6 h. The desired product **3e** was isolated as a yellow oil (29.4 mg, 63% yield, 3.1:1 d.r.).

HRMS (ESI$^+$) m/z: $[M + Na]^+$ Calcd. for $C_{17}H_{12}BrF_3O_5SNa$ 486.9433; Found: 486.9426.

Major diastereoisomer: **^{1}H NMR** (400 MHz, C_6D_6) δ 7.11 – 7.06 (m, 2H), 6.67 – 6.57 (m, 2H), 5.92 (d, *J* = 11.9 Hz, 1H), 5.76 (dd, *J* = 11.9, 6.1 Hz, 1H), 5.60 (ddd, *J* = 10.3, 6.0, 2.2 Hz, 1H), 4.67 (ddd, *J* = 10.3, 3.7, 0.9 Hz, 1H), 2.86 (dd, *J* = 3.7, 2.2 Hz, 1H), 1.12 (s, 3H). **^{13}C NMR** (101 MHz, C_6D_6) δ 173.99, 140.5, 138.0, 132.4 (2C), 127.9, 127.6, 127.3 (2C), 126.5, 122.9, 122.3, 120.76, 49.3, 46.4, 21.1. ^{19}F NMR (376 MHz, C_6D_6) δ -73.97.

HPLC of **4e** 71% ee [Chiralcel AD-H column, hexane/*i*PrOH 99:1, flow rate 1 mL/min, 25 °C, λ = 280 nm; t_R (major) = 9.8 min and t_R (minor) = 14.6 min].

Minor diastereoisomer: **^{1}H NMR** (400 MHz, C_6D_6) δ 7.14 – 7.11 (m, 2H), 6.67 – 6.57 (m, 2H), 6.04 (d, *J* = 11.8 Hz, 1H), 5.83 (dd, *J* = 11.8, 5.9 Hz, 1H), 5.40 (ddd, *J* = 10.0, 5.9, 2.1 Hz, 1H), 4.07 (ddd, *J* = 10.0, 4.0, 0.9 Hz, 1H), 2.43 (dd, *J* = 4.0, 2.1 Hz, 1H), 1.06 (s, 3H).**^{13}C NMR** (101 MHz, C_6D_6) δ 174.04, 136.25, 135.96, 132.4 (2C), 129.2 (2C), 128.9, 126.1, 125.7, 122.8, 122.6, 122.5, 49.9, 46.6, 26.2. ^{19}F NMR (376 MHz, C_6D_6) δ -73.94.

HPLC of **4e** 34% ee [Chiralcel AD-H column, hexane/*i*PrOH 99:1, flow rate 1 mL/min, 25 °C, λ = 280 nm; t_R (major) = 23.3 min and t_R (minor) = 17.4 min].

(3*R*,3a*S*)-3-(4-Chlorophenyl)-3-methyl-2-oxo-3,3a-dihydro-2*H*-cyclohepta[b]furan-8-yl trifluoromethanesulfonate (3f)

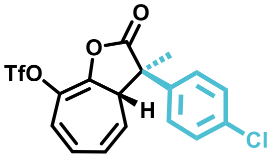

Synthesized from tropolone derivative **1b** (25.4 mg) and diazo compound **2f** (23.4 mg) by following the general procedure Step 1 at -40 °C within 6 h. The desired product **3f** was isolated as a light yellow oil (27.4 mg, 65% yield, 2.9:1 d.r.).

HRMS (ESI$^+$) m/z: $[M + Na]^+$ Calcd. for $C_{17}H_{12}ClF_3O_5SNa$ 442.9938; Found: 442.9928.

Major diastereoisomer: **^{1}H NMR** (400 MHz, C_6D_6) δ 6.99 – 6.91 (m, 2H), 6.73 – 6.65 (m, 2H), 5.93 (d, *J* = 11.9 Hz, 1H), 5.78 (dd, *J* = 11.9, 6.0 Hz, 1H), 5.61 (ddd, *J* = 10.2, 6.0, 2.2 Hz, 1H), 4.69 (ddd, *J* = 10.3, 3.6, 1.0 Hz, 1H), 2.88 (dd, *J* = 3.7, 2.2 Hz, 1H), 1.14 (s, 3H). **^{13}C NMR** (101 MHz, C_6D_6) δ 174.1, 140.0, 138.0, 134.2, 129.45 (2C), 128.2, 127.6, 127 (2C), 126.1, 122.81, 120.8, 49.2, 46.5, 21.2. ^{19}F NMR (376 MHz, C_6D_6) δ -73.97.

HPLC of **4f** 77% ee [Chiralcel AD-H column, hexane/*i*PrOH 99:1, flow rate 1 mL/min, 25 °C, λ = 280 nm; t_R (major) = 20.3 min and t_R (minor) = 18.3 min].

Minor diastereoisomer: **^{1}H NMR** (400 MHz, C_6D_6) δ 6.99 – 6.91 (m, 2H), 6.73 – 6.65 (m, 2H), 6.05 (d, *J* = 11.8 Hz, 1H), 5.84 (dd, *J* = 11.8, 5.9 Hz, 1H), 5.41 (ddd, *J* = 10.0, 5.9, 2.1 Hz, 1H), 4.09 (ddd, *J* = 10.0, 4.0, 0.9 Hz, 1H), 2.45 (dd, *J* = 4.0, 2.1 Hz, 1H), 1.09 (s, 3H). **^{13}C NMR** (101 MHz, C_6D_6) δ 174.3, 136.3, 135.5, 134.3,

129.39 (2C), 128.91, 128.9 (2C), 126.5, 125.7, 122.8, 122.6, 49.9, 46.7, 26.2. ^{19}F NMR (376 MHz, C_6D_6) δ -73.94.

HPLC of **4f** 50% ee [Chiralcel AD-H column, hexane/*i*PrOH 99:1, flow rate 1 mL/min, 25 °C, λ = 280 nm; t_R (major) = 10.2 min and t_R (minor) = 13 min].

(3*R*,3a*S*)-2-Oxo-2',3'-dihydro-2*H*,3a*H*-spiro[cyclohepta[*b*]furan-3,1'-inden]-8-yl trifluoromethanesulfonate (3g)

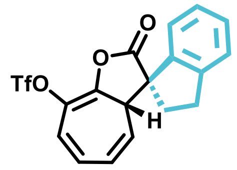

Synthesized from tropolone derivative **1b** (25.4 mg) and diazo compound **2g** (20.7 mg) by following the general procedure Step 1 at -40 °C within 6 h. The desired product **3g** was isolated as a colorless oil (18 mg, 45% yield, 17:1 d.r.).

HRMS (ESI$^+$) m/z: [M + Na]$^+$ Calcd. for $C_{18}H_{13}F_3O_5SNa$ 421.0328; Found: 421.0318.

Major diastereoisomer: **^{1}H NMR** (400 MHz, C_6D_6) δ 6.98 – 6.84 (m, 4H), 6.14 (d, *J* = 11.7 Hz, 1H), 5.92 (dd, *J* = 11.7, 5.8 Hz, 1H), 5.63 (ddd, *J* = 9.7, 5.8, 2.1 Hz, 1H), 4.61 (ddd, *J* = 9.7, 4.5, 0.9 Hz, 1H), 3.07 (dt, *J* = 16.1, 8.2 Hz, 1H), 2.60 (dd, *J* = 4.5, 2.1 Hz, 1H), 2.47 (ddd, *J* = 15.9, 8.9, 3.6 Hz, 1H), 2.17 (ddd, *J* = 13.1, 8.2, 3.6 Hz, 1H), 1.81 (dt, *J* = 13.1, 8.6 Hz, 1H). **^{13}C NMR** (101 MHz, C_6D_6) δ 174.4, 143.6, 143.2, 136.1, 129.1, 129.0, 127.9, 127.0, 125.3, 124.8, 123.0, 122.0, 121.5, 56.4, 45.5, 31.7, 31.2. ^{19}F NMR (376 MHz, C_6D_6) δ -74.01.

HPLC of **4g** 99% ee [Chiralcel AS-H column, hexane/*i*PrOH 98:2, flow rate 1 mL/min, 25 °C, λ = 280 nm; t_R (major) = 12.6 min and t_R (minor) = 17.4 min].

Minor diastereoisomer: **^{1}H NMR** (400 MHz, C_6D_6) δ 6.98 – 6.84 (m, 4H), 5.95 – 5.87 (m, 1H), 5.75 – 5.65 (m, 1H), 5.50 – 5.26 (m, 1H), 4.44 – 4.24 (m, 1H), 2.82 (t, *J* = 2.7 Hz, 1H), 2.72 – 2.62 (m, 1H), 2.42 – 2.24 (m, 2H), 1.45 – 1.07 (m, 1H). **^{13}C NMR** could not be provided due to the low amount of the minor diastereomer formed during the reaction. ^{19}F NMR (376 MHz, C_6D_6) δ -73.94.

(3*R*,3a*S*)-5'-Methoxy-2-oxo-2',3'-dihydro-2*H*,3a*H*-spiro[cyclohepta[*b*]furan-3,1'-inden]-8-yl trifluoromethanesulfonate (3h)

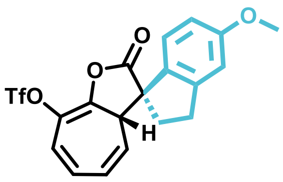

Synthesized from tropolone derivative **1b** (25.4 mg) and diazo compound **2h** (24.3 mg) by following the general procedure Step 1 at -40 °C within 16 h. The desired product **3h** was isolated as a light yellow oil (13.3 mg, 31% yield, 3.3:1 d.r.).

HRMS (ESI$^+$) m/z: [M + Na]$^+$ Calcd. for $C_{19}H_{15}F_3O_6SNa$ 451.0434; Found: 451.0426.

Major diastereoisomer: **^{1}H NMR** (400 MHz, C_6D_6) δ 6.83 (d, *J* = 8.4 Hz, 1H), 6.57 (dd, *J* = 2.6, 1.2 Hz, 1H), 6.51 (dd, *J* = 8.5, 2.5 Hz, 1H), 6.16 (d, *J* = 11.7 Hz, 1H), 5.94 (dd, *J* = 11.7, 5.8 Hz, 1H), 5.65 (ddd, *J* = 9.7, 5.8, 2.0 Hz, 1H), 4.66 (ddd, *J* = 9.7, 4.5, 0.9 Hz, 1H), 3.26 (s, 3H), 3.07 (dt, *J* = 16.3, 8.3 Hz, 1H), 2.65 (dd, *J* = 4.6, 2.1 Hz, 1H), 2.52 – 2.35 (m, 1H), 2.21 (ddd, *J* = 13.1, 8.1, 3.4 Hz, 1H), 1.87 (dt, *J* = 13.1, 8.7 Hz, 1H). **^{13}C NMR** (101 MHz, C_6D_6) δ 174.7, 161.0, 145.0, 136.2, 135.6, 129.0, 127.0, 124.9, 122.9, 122.7, 121.6, 113.8, 110.9, 55.7, 55.01, 45.6, 32.1, 31.3. ^{19}F NMR (376 MHz, C_6D_6) δ -74.02.

HPLC of **4h** 95% ee [Chiralcel AD-H column, hexane/*i*PrOH 95:5, flow rate 1 mL/min, 25 °C, λ = 254 nm; t_R (major) = 13.9 min and t_R (minor) = 11.5 min].

Minor diastereoisomer: **^{1}H NMR** (400 MHz, C_6D_6) δ 6.88 (d, *J* = 8.4 Hz, 1H), 6.6 – 6.55 (m, 1H), 6.46 (dd, *J* = 8.5, 2.5 Hz, 1H), 5.92 (d, *J* = 12.0 Hz, 1H), 5.69 (dd, *J* = 12.0, 6.2 Hz, 1H), 5.45 (ddd, *J* = 10.5, 6.2, 2.4 Hz, 1H), 4.41 (ddd, *J* = 10.5, 3.0, 0.9 Hz, 1H), 3.24 (s, 3H), 2.86 (t, *J* = 2.7 Hz, 1H), 2.71 – 2.58 (m, 1H), 2.52 – 2.35 (m, 2H), 1.51 – 1.38 (m, 1H). **^{13}C NMR** (101 MHz, C_6D_6) δ 174.1, 161.0, 146.5, 140.6, 131.2, 127.6, 127.2, 126.5, 126.1, 123.3, 122.6, 113.3, 111.2, 57.5, 54.97, 44.1, 37.3, 31.2. ^{19}F NMR (376 MHz, C_6D_6) δ -73.93.

(3*R*,3a*S*)-6'-Fluoro-2-oxo-2',3'-dihydro-2*H*,3a*H*-spiro[cyclohepta[*b*]furan-3,1'-inden]-8-yl trifluoromethanesulfonate (3i)

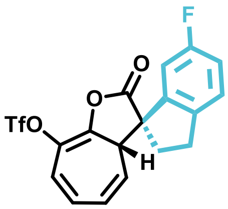

Synthesized from tropolone derivative **1b** (25.4 mg) and diazo compound **2i** (22.8 mg) by following the general procedure Step 1 at -40 °C within 16 h. The desired product **3i** was isolated as a light yellow oil (14.2 mg, 34% yield, only major d.r.).

HRMS (ESI$^+$) m/z: [M + Na]$^+$ Calcd. for $C_{18}H_{12}F_4O_5SNa$ 439.0234; Found: 439.0227.

^{1}H NMR (400 MHz, C_6D_6) δ 6.68 – 6.59 (m, 3H), 6.07 (d, *J* = 11.7 Hz, 1H), 5.88 (dd, *J* = 11.8, 5.8 Hz, 1H), 5.61 (ddd, *J* = 9.7, 5.8, 2.0 Hz, 1H), 4.55 (ddd, *J* = 9.8, 4.3, 0.9 Hz, 1H), 2.91 (ddd, *J* = 16.2, 8.2, 1.9 Hz, 1H), 2.52 (dd, *J* = 4.4, 2.1 Hz, 1H), 2.32 (dddd, *J* = 15.7, 8.9, 3.5, 1.4 Hz, 1H), 2.10 (ddd, *J* = 13.1, 8.1, 3.4 Hz, 1H), 1.78 (dt, *J* = 13.2, 8.6 Hz, 1H). **^{13}C NMR** (101 MHz, C_6D_6) δ 173.7, 135.9, 128.9, 127.2, 126.5, 126.4, 125.0, 123.0, 121.1, 116.4, 116.1, 109.6, 109.4, 56.6, 45.0, 32.3, 30.4. ^{19}F NMR (376 MHz, C_6D_6) δ -74.00, -113.84 – -113.91 (m).

HPLC of **4i** 99% ee [Chiralcel AD-H column, hexane/*i*PrOH 98:2, flow rate 1 mL/min, 25 °C, λ = 280 nm; t_R (major) = 8.7 min and t_R (minor) = 9.7 min].

(3*R*,3a*S*)-2-Oxo-2',3'-dihydro-2*H*,3a*H*-spiro[cyclohepta[*b*]furan-3,1'-cyclopenta[a]naphthalen]-8-yl trifluoromethanesulfonate (3j)

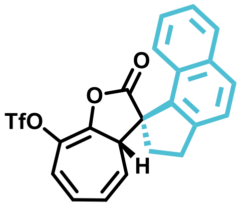

Synthesized from tropolone derivative **1b** (25.4 mg) and diazo compound **2j** (26.7 mg) by following the general procedure Step 1 at -20 °C within 24 h. The desired product **3j** was isolated as a colorless oil (13 mg, 29% yield, only major d.r.).

HRMS (ESI$^+$) m/z: [M + Na]$^+$ Calcd. for $C_{22}H_{15}F_3O_5SNa$ 471.0484; Found: 471.0477.

^{1}H NMR (400 MHz, C_6D_6) δ 7.58 (td, *J* = 8.9, 1.2 Hz, 2H), 7.46 (d, *J* = 8.4 Hz, 1H), 7.21 (ddd, *J* = 8.4, 6.9, 1.4 Hz, 1H), 7.09 (ddd, *J* = 8.1, 6.9, 1.1 Hz, 1H), 6.98 (d, *J* = 8.4 Hz, 1H), 5.91 (d, *J* = 11.9 Hz, 1H), 5.63 (ddd, *J* = 11.9, 6.2, 1.2 Hz, 1H), 5.52 (dddd, *J* = 9.5, 6.2, 2.3, 1.0 Hz, 1H), 4.78 (dd, *J* = 10.4, 3.3 Hz, 1H), 3.53 (t, *J* = 2.8 Hz, 1H), 2.70 – 2.55 (m, 2H), 2.37 (dt, *J* = 13.6, 9.0 Hz, 1H), 2.24 (ddd, *J* = 13.6, 7.1, 3.0 Hz, 1H). **^{13}C NMR** (101 MHz, C_6D_6) δ 175.3, 142.6, 139.5, 137.2, 134.1, 130.4, 129.8, 128.7, 128.18, 128.12, 127.9, 126.6, 125.8, 122.9, 122.6, 122.3, 121.7, 57.5, 45.0, 34.3, 32.5. ^{19}F NMR (376 MHz, C_6D_6) δ -73.98.

HPLC of **4j** 28% ee [Chiralcel AD-H column, hexane/*i*PrOH 99:1, flow rate 1 mL/min, 25 °C, λ = 280 nm; t_R (major) = 22.8 min and t_R (minor) = 15.7 min].

(3*R*,3a*S*)-2-Oxo-3',4'-dihydro-2*H*,2'*H*,3a*H*-spiro[cyclohepta[*b*]furan-3,1'-naphthalen]-8-yl trifluoromethanesulfonate (3k)

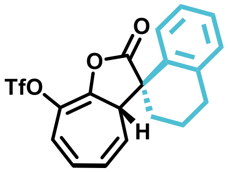

Synthesized from tropolone derivative **1b** (25.4 mg) and diazo compound **2k** (22.4 mg) by following the general procedure Step 1 at -40 °C within 16 h. The desired product **3k** was isolated as a colorless oil (17.4 mg, 42% yield, 14.3:1 d.r.). Second purification afforded product **3k** as single major diastereomer (15.7 mg, 38% yield).

HRMS (ESI^+) m/z: $[M + Na]^+$ Calcd. for $C_{19}H_{15}F_3O_5SNa$ 435.0484; Found: 435.0476.

Major diastereoisomer: **^{1}H NMR** (400 MHz, C_6D_6) δ 7.11 – 6.99 (m, 1H), 6.92 – 6.84 (m, 2H), 6.77 – 6.68 (m, 1H), 6.07 (d, *J* = 11.7 Hz, 1H), 5.87 (dd, *J* = 11.7, 5.8 Hz, 1H), 5.64 (ddd, *J* = 9.8, 5.8, 2.0 Hz, 1H), 4.78 (ddd, *J* = 9.9, 4.6, 0.9 Hz, 1H), 2.61 (dd, *J* = 4.6, 2.0 Hz, 1H), 2.40 (ddd, *J* = 15.2, 9.2, 5.5 Hz, 1H), 2.27 (dt, *J* = 16.8, 5.3 Hz, 1H), 1.94 (ddd, *J* = 13.7, 10.9, 2.4 Hz, 1H), 1.76 – 1.59 (m, 2H), 1.28 – 1.18 (m, 1H). **^{13}C NMR** (101 MHz, C_6D_6) δ 175.3, 137.5, 136.4, 135.9, 130.2, 129.0, 127.94, 127.88, 126.8, 125.7, 125.2, 122.7, 120.8, 48.7, 48.0, 28.8, 28.3, 19.1. ^{19}F NMR (376 MHz, C_6D_6) δ -74.00.

HPLC of **4k** 16% ee [Chiralcel OD-H column, hexane/*i*PrOH 99:1, flow rate 1 mL/min, 25 °C, λ = 280 nm; t_R (major) = 6.9 min and t_R (minor) = 8 min].

Minor diastereoisomer: **^{1}H NMR** (400 MHz, C_6D_6) δ 7.11 – 6.99 (m, 1H), 6.92 – 6.84 (m, 1H), 6.84 – 6.75 (m, 2H), 6.01 (d, *J* = 11.9 Hz, 1H), 5.78 (dd, *J* = 11.9, 6.0 Hz, 1H), 5.39 (ddd, *J* = 10.3, 6.0, 2.2 Hz, 1H), 4.37 (ddd, *J* = 10.2, 3.5, 0.9 Hz, 1H), 2.74 (t, *J* = 2.9 Hz, 1H), 2.47 – 2.35 (m, 1H), 2.30 – 2.23 (m, 1H), 2.00 – 1.88 (m, 1H), 1.76 – 1.59 (m, 2H), 1.31 – 1.24 (m, 1H). **^{13}C NMR** could not be provided due to the low amount of the minor diastereomer formed during the reaction. ^{19}F NMR (376 MHz, C_6D_6) δ -73.91.

(3*R*,3a*S*)-5-Bromo-3-methyl-2-oxo-3-phenyl-3,3a-dihydro-2*H*-cyclohepta[*b*]furan-8-yl trifluoromethanesulfonate (3l)

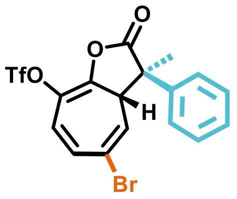

Synthesized from tropolone derivative **1c** (33.3 mg) and diazo compound **2a** (19.2 mg) by following the general procedure Step 1 at -40 °C within 3 h. The desired product **3l** was isolated as a light yellow oil (44.2 mg, 95% yield, 5.8:1 d.r.).

HRMS (ESI^+) m/z: $[M + Na]^+$ Calcd. for $C_{17}H_{12}BrF_3O_5SNa$ 486.9433; Found: 486.9435.

Major diastereoisomer: **^{1}H NMR** (400 MHz, C_6D_6) δ 6.97 – 6.90 (m, 3H), 6.89 – 6.84 (m, 2H), 5.92 (dd, *J* = 12.1, 0.9 Hz, 1H), 5.66 (d, *J* = 12.1 Hz, 1H), 5.24 (d, *J* = 4.4 Hz, 1H), 2.87 (d, *J* = 4.4 Hz, 1H), 1.04 (s, 3H). **^{13}C NMR** (101 MHz, C_6D_6) δ 173.91, 141.6, 139.5, 130.0, 129.45 (2C), 128.23, 127.1, 125.3 (2C), 124.0, 122.2, 119.4, 49.5, 47.65, 20.8. ^{19}F NMR (376 MHz, C_6D_6) δ -73.89.

HPLC of **4l** 51% ee [Chiralcel LUX column, hexane/*i*PrOH 98:2, flow rate 1 mL/min, 25 °C, λ = 280 nm; t_R (major) = 12.5 min and t_R (minor) = 11.4 min].

Minor diastereoisomer: **^{1}H NMR** (400 MHz, C_6D_6) δ 6.97 – 6.90 (m, 3H), 6.89 – 6.84 (m, 2H), 5.99 (dd, *J* = 12.0, 1.0 Hz, 1H), 5.79 (d, *J* = 12.0 Hz, 1H), 4.62 (d, *J* = 4.2 Hz, 1H), 2.54 (d, *J* = 4.4 Hz, 1H), 1.11 (s, 3H). **^{13}C NMR** (101 MHz, C_6D_6) δ 173.96, 138.9, 136.2, 130.4, 129.41 (2C), 128.5, 124.49, 124.41 (2C), 124.1, 123.8, 118.3, 50.3, 47.72, 25.7. ^{19}F NMR (376 MHz, C_6D_6) δ -73.85.

HPLC of **4l** 33% ee [Chiralcel LUX column, hexane/*i*PrOH 98:2, flow rate 1 mL/min, 25 °C, λ = 280 nm; t_R (major) = 10.3 min and t_R (minor) = 9.6 min].

(3*R*,3a*S*)-3-Methyl-2-oxo-3-(p-tolyl)-3,3a-dihydro-2*H*-cyclohepta[*b*]furan-8-yl trifluoromethanesulfonate (3m)

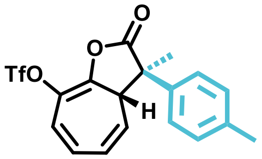

Synthesized from tropolone derivative **1b** (25.4 mg) and diazo compound **2m** (20.9 mg) by following the general procedure Step 1 at -20 °C within 24 h. The desired product **3m** was isolated as a light yellow oil (14.8 mg, 37% yield, 5.6:1 d.r.).

HRMS (ESI$^+$) m/z: [M + Na]$^+$ Calcd. for $C_{18}H_{15}F_3O_5SNa$ 423.0484; Found: 423.0478.

Major diastereoisomer: **^{1}H NMR** (400 MHz, $CDCl_3$) δ 7.25 – 7.16 (m, 4H), 6.44 (dd, *J* = 11.9, 5.9 Hz, 1H), 6.33 (d, *J* = 11.9 Hz, 1H), 6.25 (ddd, *J* = 10.1, 5.9, 2.2 Hz, 1H), 5.54 (ddd, *J* = 10.1, 3.9, 0.9 Hz, 1H), 3.40 (dd, *J* = 3.9, 2.2 Hz, 1H), 2.33 (s, 3H), 1.85 (s, 3H). **^{13}C NMR** (101 MHz, $CDCl_3$) δ 175.4, 138.8, 138.2, 130.1 (2C), 130.0, 128.8, 127.7, 126.5, 125.2 (2C), 123.0, 121.4, 49.5, 47.0, 21.4, 21.08. ^{19}F NMR (376 MHz, C_6D_6) δ -73.67.

HPLC of **4m** 23% ee [Chiralcel AD-H column, hexane/*i*PrOH 98:2, flow rate 1 mL/min, 25 °C, λ = 280 nm; t_R (major) = 18.0 min and t_R (minor) = 15.9 min].

Minor diastereoisomer: **^{1}H NMR** (400 MHz, $CDCl_3$) δ 7.25 – 7.16 (m, 4H), 6.40 – 6.32 (m, 1H), 5.97 (ddd, *J* = 10.0, 5.7, 2.1 Hz, 1H),4.59 (ddd, *J* = 10.0, 3.9, 0.9 Hz, 1H), 3.12 (dd, *J* = 4.0, 2.2 Hz, 1H), 2.38 (s, 3H), 1.85 (s, 3H). **^{13}C NMR** (101 MHz, $CDCl_3$) δ 175.4, 138.3, 137.4, 129.7, 129.2, 127.2 (2C), 126.6, 125.7 (2C), 123.5, 122.6, 120.3, 50.4, 47.04, 26.7, 21.18.

HPLC of **4m** 25% ee [Chiralcel AD-H column, hexane/*i*PrOH 98:2, flow rate 1 mL/min, 25 °C, λ = 280 nm; t_R (major) = 8.3 min and t_R (minor) = 10.9 min].

(3*R*,3a*S*)-3-methyl-3-(4-Nitrophenyl)-2-oxo-3,3a-dihydro-2*H*-cyclohepta[*b*]furan-8-yl trifluoromethanesulfonate (3n)

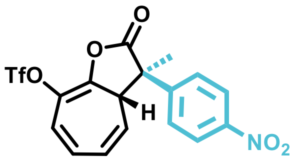

Synthesized from tropolone derivative **1b** (25.4 mg) and diazo compound **2n** (24.6 mg) by following the general procedure Step 1 at 0 °C within 24 h. The desired product **3n** was isolated as a dark orange oil (6.6 mg, 15% yield, 6.3:1 d.r.).

HRMS (ESI$^+$) m/z: [M + Na]$^+$ Calcd. for $C_{17}H_{12}F_3NO_7SNa$ 454.0179; Found: 454.0170.

Major diastereoisomer: **^{1}H NMR** (400 MHz, C_6D_6) δ 7.64 – 7.58 (m, 2H), 6.66 – 6.61 (m, 2H), 5.94 (d, *J* = 11.9 Hz, 1H), 5.79 (dd, *J* = 11.9, 6.1 Hz, 1H), 5.62 (ddd, *J* = 10.3, 6.1, 2.2 Hz, 1H), 4.64 (ddd, *J* = 10.3, 3.7, 0.9 Hz, 1H), 2.79 (dd, *J* = 3.6, 2.2 Hz, 1H), 1.07 (s, 3H). **^{13}C NMR** (101 MHz, C_6D_6) δ 173.4, 147.4, 137.6, 128.17, 128.0, 126.3 (2C), 126.2, 124.2 (2C), 122.9, 122.1, 120.3, 49.6, 46.1, 21.2. ^{19}F NMR (376 MHz, C_6D_6) δ -73.94.

Minor diastereoisomer: **^{1}H NMR** (400 MHz, C_6D_6) δ 7.69 – 7.64 (m, 2H), 6.71 – 6.66 (m, 2H), 6.06 (d, *J* = 11.8 Hz, 1H), 5.86 (dd, *J* = 11.8, 5.9 Hz, 1H), 5.44 (ddd, *J* = 9.9, 5.9, 2.1 Hz, 1H), 3.90 (ddd, *J* = 9.9, 4.1, 0.9 Hz, 1H), 2.38 (dd, *J* = 4.2, 2.1 Hz, 1H), 1.01 (s, 3H). **^{13}C NMR** (101 MHz, C_6D_6) δ 173.6, 147.6, 143.2, 129.1, 128.19 (2C), 127.4, 126.7, 124.0 (2C), 122.8, 120.8, 120.2, 50.1, 46.5, 26.5. ^{19}F NMR (376 MHz, C_6D_6) δ -73.91.

(3*R*,3a*S*)-5-Isopropyl-3-methyl-2-oxo-3-phenyl-3,3a-dihydro-2*H*-cyclohepta[b]furan-8-yl trifluoromethanesulfonate (3o)

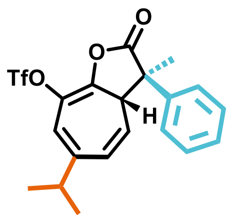

Synthesized from tropolone derivative **1d** (29.6 mg) and diazo compound **2a** (19.2 mg) by following the general procedure Step 1 at -20 °C within 24h. The desired product **3o** was isolated as a colorless oil (9.1 mg, 21% yield, 2.8:1 d.r.).

HRMS (ESI^{+}) m/z: $[M + Na]^{+}$ Calcd. for $C_{20}H_{19}F_3O_5SNa$ 451.0798; Found: 451.0785.

Major diastereoisomer: **^{1}H NMR** (400 MHz, C_6D_6) δ 7.07 – 6.93 (m, 5H), 5.95 (s, 1H), 5.72 (dd, *J* = 10.4, 2.2 Hz, 1H), 4.97 (dd, *J* = 10.4, 4.0 Hz, 1H), 3.00 (ddd, *J* = 4.0, 2.2, 0.8 Hz, 1H), 2.06 (dt, *J* = 13.1, 6.7 Hz, 1H), 1.34 (s, 3H), 0.81 (dd, *J* = 6.9, 3.2 Hz, 6H). **^{13}C NMR** (101 MHz, C_6D_6) δ 174.8, 148.7, 142.4, 136.6, 129.4 (2C), 128.7, 128.1, 125.72, 125.4 (2C), 121.5, 117.2, 49.8, 46.83, 35.9, 22.79, 21.6, 21.0. ^{19}F NMR (376 MHz, C_6D_6) δ -73.92.

HPLC of **4o** 64% ee [Chiralcel OJ-H column, hexane/*i*PrOH 97:3, flow rate 1 mL/min, 25 °C, λ = 280 nm; t_R (major) = 9.2 min and t_R (minor) = 11.4 min].

Minor diastereoisomer: **^{1}H NMR** (400 MHz, C_6D_6) δ 7.07 – 6.93 (m, 5H), 6.08 (s, 1H), 5.39 (dd, *J* = 10.3, 2.1 Hz, 1H), 4.35 (dd, *J* = 10.3, 4.1 Hz, 1H), 2.63 (ddd, *J* = 4.1, 2.1, 0.8 Hz, 1H), 2.12 – 2.01 (m, 1H), 1.23 (s, 3H), 0.84 – 0.77 (m, 6H). **^{13}C NMR** (101 MHz, C_6D_6) δ 175.0, 149.4, 143.3, 137.4, 129.2 (2C), 127.5, 127.4, 126.9, 125.75 (2C), 123.7, 116.9, 50.6, 46.79, 36.0, 26.0, 22.83, 21.7. ^{19}F NMR (376 MHz, C_6D_6) δ -73.89.

HPLC of **4o** 40% ee [Chiralcel OJ-H column, hexane/*i*PrOH 97:3, flow rate 1 mL/min, 25 °C, λ = 280 nm; t_R (major) = 9.8 min and t_R (minor) = 14.4 min].

Late-stage modifications

(*R*)-*N*-Isopropyl-2-(7-oxocyclohepta-1,3,5-trien-1-yl)-2-phenylpropanamide (5)

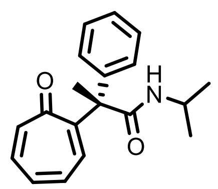

To the suspension of **3a** (25 mg, 0.065 mmol, 1 equiv) and K_2CO_3 (8.9 mg, 0.065 mmol, 0.1 equiv) in THF (324 µl, 0.2 M), isopropyl amine (8.3 µl, 0.097 mmol, 1.5 equiv) was added under Ar atmosphere. The reaction mixture was stirred for 2 h at rt. After complete conversion (monitored by TLC), the reaction mixture was then concentrated under reduced pressure and purified directly by flash column chromatography (5%-70% Et_2O in PE) to afford the product **5** as a yellow viscous oil in 81% yield (15.4 mg).

HRMS (ESI^+) m/z: $[M + Na]^+$ Calcd. for $C_{19}H_{21}NO_2Na$ 318.1465; Found: 318.1476.

^{1}H NMR (400 MHz, $CDCl_3$) δ 7.37 – 7.28 (m, 4H), 7.28 – 7.22 (m, 1H), 7.16 – 7.06 (m, 2H), 7.01 – 6.88 (m, 3H), 6.49 (d, *J* = 7.6 Hz, 1H), 4.07 (dp, *J* = 7.8, 6.5 Hz, 1H), 1.90 (s, 3H), 1.19 (d, *J* = 6.5 Hz, 3H), 1.03 (d, *J* = 6.5 Hz, 3H). **^{13}C NMR** (101 MHz, $CDCl_3$) δ 188.1, 172.8, 156.9, 144.1, 140.3, 135.6, 134.5, 133.5, 133.2, 128.6 (2C), 127.2 (2C), 127.1, 57.8, 41.9, 26.7, 22.6, 22.5.

HPLC of **5** 68% ee [Chiralcel OD-H column, hexane/*i*PrOH 93:7, flow rate 1 mL/min, 25 °C, λ = 230 nm; t_R (major) = 14.3 min and t_R (minor) = 16.3 min].

(3*R*,3a*S*)-3-Methyl-2-oxo-3-phenyl-3,3a,4,5,6,7-hexahydro-2*H*-cyclohepta[*b*]furan-8-yl trifluoromethanesulfonate (6)

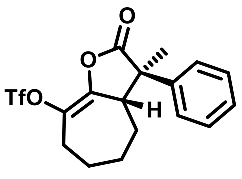

The reduction of a solution of **3a** (25 mg, 0.065 mmol, 1 equiv) in EtOAc (6.5 mL, 0.01 M) was performed in an H-Cube flow reactor at rt using a 10% Pd/C cartridge at a flow rate of 1 mL/min under 2 bar of H_2. Complete conversion (monitored by TLC) was achieved within one cycle. The resulting reaction mixture was then concentrated under reduced pressure and purified directly by flash column chromatography (10%-50% DCM in PE) to afford the product **6** as a colorless oil (24 mg, 95% yield, 3.7:1 d.r.)

HRMS (ESI^+) m/z: $[M + Na]^+$ Calcd. for $C_{17}H_{17}F_3O_5SNa$ 413.0641; Found: 413.0646.

Major diastereoisomer: **^{1}H NMR** (400 MHz, $CDCl_3$) δ 7.43 – 7.31 (m, 5H), 3.25 (dq, *J* = 11.9, 2.6 Hz, 1H), 2.59 – 2.54 (m, 2H), 1.98 – 1.9 (m, 1H), 1.96 – 1.81 (m, 2H), 1.73 – 1.63 (m, 2H), 1.59 (s, 3H), 1.44 – 1.35 (m, 1H). **^{13}C NMR** (101 MHz, $CDCl_3$) δ 175.6, 145.8, 139.9, 131.26, 129.0 (2C), 128.0, 126.4 (2C), 51.35, 48.8, 30.0, 26.3, 25.05, 24.9, 18.7. ^{19}F NMR (376 MHz, $CDCl_3$) δ -74.28.

HPLC of **8** 71% ee [Lux 3u Amylose-2 column, hexane/*i*PrOH 9:1, flow rate 1 mL/min, 25 °C, λ = 210 nm; t_R (major) = 14.0 min and t_R (minor) = 11.0 min].

Minor diastereoisomer: **^{1}H NMR** (400 MHz, $CDCl_3$) δ 7.43 – 7.31 (m, 3H), 7.19 – 7.15 (m, 2H), 3.04 (dq, *J* = 12.1, 2.8 Hz, 1H), 2.54 – 2.47 (m, 2H), 1.84 – 1.81 (m, 2H), 1.75 (s, 3H), 1.51 – 1.45 (m, 1H), 1.5 – 1.38 (m, 2H), 0.89 – 0.61 (m, 1H). **^{13}C NMR** (101 MHz, $CDCl_3$) δ 175.7, 146.1, 136.9, 131.30, 128.9 (2C), 128.1, 126.3 (2C), 51.37, 48.6, 30.3, 27.4, 26.7, 24.96, 22.1. ^{19}F NMR (376 MHz, $CDCl_3$) δ -74.32.

HPLC of **8** 56% ee [Lux 3u Amylose-2 column, hexane/*i*PrOH 9:1, flow rate 1 mL/min, 25 °C, λ = 210 nm; t_R (major) = 10.0 min and t_R (minor) = 9.3 min].

(3*R*,3a*S*)-8-((4-Chlorophenyl)thio)-3-methyl-3-phenyl-3,3a-dihydro-2*H*-cyclohepta[*b*]furan-2-one (7)

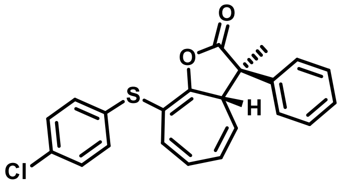

To the solution of **3a** (25 mg, 0.065 mmol, 1 equiv) and Cs_2CO_3 (31.6 mg, 0.097 mmol, 1.5 equiv) in DMF (431 µl, 0.15 M), *p*-chlorothiophenol (12.2 mg, 0.084 mmol, 1.3 equiv) was added under Ar atmosphere. The reaction mixture was stirred for 0.5 h at rt. After complete conversion (monitored by TLC), the reaction mixture was directly purified by flash column chromatography (5%-25% DCM in PE) to afford the product **7** as a yellow viscous oil in 87% yield (21.4 mg, 4.7:1 d.r.).

HRMS (ESI^+) m/z: $[M + Na]^+$ Calcd. for $C_{22}H_{17}ClO_2SNa$ 403.0530; Found: 403.0533.

Major diastereoisomer: **^{1}H NMR** (400 MHz, $CDCl_3$) δ 7.39 – 7.34 (m, 2H), 7.34 – 7.30 (m, 1H), 7.30 – 7.26 (m, 2H), 7.20 – 7.15 (m, 2H), 7.05 – 6.93 (m, 2H), 6.53 – 6.38 (m, 2H), 6.36 – 6.26 (m, 1H), 5.59 (dd, *J* = 9.6, 4.6 Hz, 1H), 3.12 (dd, *J* = 4.6, 2.0 Hz, 1H), 1.89 (s, 3H). **^{13}C NMR** (101 MHz, $CDCl_3$) δ 177.0, 145.6, 143.0, 135.2, 133.0, 132.02, 129.7 (2C), 129.38 (2C), 129.09 (2C), 128.9, 128.03, 127.6, 125.1 (2C), 120.3, 104.4, 50.6, 49.8, 21.3.

Minor diastereoisomer: **^{1}H NMR** (400 MHz, $CDCl_3$) δ 7.47 – 7.41 (m, 4H), 7.39 – 7.34 (m, 1H), 7.20 – 7.15 (m, 2H), 7.05 – 6.93 (m, 2H), 6.53 – 6.38 (m, 2H), 6.03 (dddd, *J* = 9.6, 5.0, 2.0, 0.8 Hz, 1H), 4.64 (dd, *J* = 9.6, 4.6 Hz, 1H), 2.91 (dd, *J* = 4.6, 2.0 Hz, 1H), 1.86 (s, 3H). **^{13}C NMR** (101 MHz, $CDCl_3$) δ 176.6, 145.1, 140.5, 135.4, 132.7, 131.95, 129.45 (2C), 129.24, 129.16 (2C), 129.09 (2C), 128.02, 127.5 (2C), 126.4, 122.4, 104.1, 51.3, 50.3, 29.0.

(3*R*,3a*S*)-3-Methyl-3,8-diphenyl-3,3a-dihydro-2*H*-cyclohepta[*b*]furan-2-one (8)

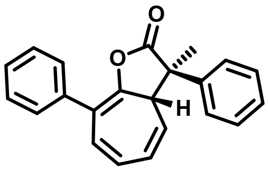

To the solution of **3a** (25 mg, 0.065 mmol, 1 equiv), $Pd(PPh_3)_4$ (3.7 mg, 0.003 mmol, 0.05 equiv) and LiCl (8.2 mg, 0.194 mmol, 3 equiv) in THF (647 µl, 0.1 M), tributylphenyltin (23 µl, 0.071 mmol, 1.1 equiv) was added under Ar atmosphere. The reaction mixture was stirred for 3 h at 90 °C using a heating block. After complete conversion (monitored by TLC), 1 M aq. $NaHCO_3$ (2 mL) was added, followed by extraction with DCM (2 x 2 mL). The combined organic phases were then concentrated under reduced pressure and purified by flash column chromatography (5%-25% DCM in PE) to afford the product **8** as a yellow viscous oil in 97% yield (19.8 mg, 3.8:1 d.r.).

HRMS (ESI^+) m/z: $[M + Na]^+$ Calcd. for $C_{22}H_{18}O_2Na$ 337.1199; Found: 337.1207.

Major diastereoisomer: **^{1}H NMR** (400 MHz, C_6D_6) δ 7.47 – 7.27 (m, 10H), 6.65 (d, *J* = 11.4 Hz, 1H), 6.56 (dd, *J* = 11.3, 5.5 Hz, 1H), 6.30 (ddd, *J* = 9.6, 5.5, 2.0 Hz, 1H), 5.58 (ddd, *J* = 9.5, 4.7, 0.8 Hz, 1H), 3.17 (dd, *J* = 4.7, 2.0 Hz, 1H), 1.90 (s, 3H). **^{13}C NMR** (101 MHz, $CDCl_3$) δ 178.0, 143.5, 138.6, 136.3, 131.7, 129.23 (2C), 129.16 (2C), 128.27 (2C), 128.1, 127.73, 127.29, 127.0, 125.1 (2C), 120.0, 114.55, 49.9, 49.5, 21.1.

HPLC of **8** 77% ee [Chiralcel AD-H column, hexane/*i*PrOH 97:3, flow rate 1 mL/min, 25 °C, λ = 280 nm; t_R (major) = 9.7 min and t_R (minor) = 6.9 min].

Minor diastereoisomer: **^{1}H NMR** (400 MHz, C_6D_6) δ 7.47 – 7.27 (m, 10H), 6.7 (d, *J* = 11.3 Hz, 1H), 6.56 (dd, *J* = 11.3, 5.5 Hz, 1H), 6.01 (ddd, *J* = 9.5, 5.5, 1.9 Hz, 1H), 4.62 (dd, *J* = 9.5, 4.8 Hz, 1H), 2.94 (dd, *J* = 4.8, 2.0 Hz, 1H), 1.93 (s, 3H). **^{13}C NMR** (101 MHz, $CDCl_3$) δ 177.9, 138.0, 137.8, 136.2, 131.3, 129.2 (2C), 128.9 (2C), 128.4, 128.3, 127.68, 127.5 (2C), 127.3 (2C), 125.7, 122.1, 114.57, 50.6, 50.1, 29.

HPLC of **8** 54% ee [Chiralcel AD-H column, hexane/*i*PrOH 97:3, flow rate 1 mL/min, 25 °C, λ = 280 nm; t_R (major) = 6.4 min and t_R (minor) = 7.9 min].

(3*R*,3a*S*)-3-Methyl-3-phenyl-8-((trimethylsilyl)ethynyl)-3,3a-dihydro-2*H*-cyclohepta[*b*]furan-2-one (9)

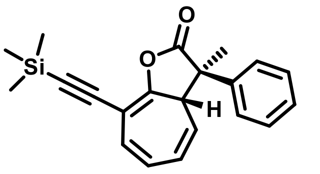

To the solution of **3a** (25 mg, 0.065 mmol, 1 equiv), trimethylsilylacetylene (31.8 mg, 0.324 mmol, 5 equiv) and Et_3N (45.1 µl, 0.324 mmol, 5 equiv) in DMF (431 µl, 0.15 M), $PdCl_2(PPh_3)_2$ (2.3 mg, 0.004 mmol, 0.05 equiv) was added under Ar atmosphere. The reaction mixture was stirred for 15 h at 50 °C using a heating block. After complete conversion (monitored by TLC), water was added (1 mL), followed by extraction with Et_2O (4 x 2 mL). The combined organic phases were then concentrated under reduced pressure and purified by flash column chromatography (5%-20% DCM in PE) to afford the product **9** as a yellow viscous oil in 96% yield (20.8 mg, 3.5:1 d.r.).

HRMS (ESI$^+$) m/z: $[M + Na]^+$ Calcd. for $C_{21}H_{22}O_2SiNa$ 357.1281; Found: 357.1292.

Major diastereoisomer: **^{1}H NMR** (400 MHz, C_6D_6) δ 7.07 – 6.96 (m, 3H), 6.91 – 6.87 (m, 2H), 6.43 (d, *J* = 11.3 Hz, 1H), 5.96 (dd, *J* = 11.3, 5.8 Hz, 1H), 5.75 (ddd, *J* = 9.8, 5.8, 2.1 Hz, 1H), 4.87 (ddd, *J* = 9.8, 4.2, 0.9 Hz, 1H), 3.02 (dd, *J* = 4.3, 2.1 Hz, 1H), 1.35 (s, 3H), 0.21 (s, 9H). **^{13}C NMR** (101 MHz, C_6D_6) δ 176.6, 149.4, 143.3, 130.5, 129.3 (2C), 127.66, 127.3, 127.1, 125.3 (2C), 119.6, 99.71, 99.35, 98.3, 50.1, 49.8, 21.2, 0.04 (3C).

Minor diastereoisomer: **^{1}H NMR** (400 MHz, C_6D_6) δ 7.07 – 6.96 (m, 2H), 6.91 – 6.87 (m, 3H), 6.56 (d, *J* = 11.4 Hz, 1H), 6.00 (dd, *J* = 11.3, 5.7 Hz, 1H), 5.50 (ddd, *J* = 9.7, 5.7, 2.0 Hz, 1H), 4.28 (dd, *J* = 9.7, 4.3 Hz, 1H), 2.60 (dd, *J* = 4.4, 2.0 Hz, 1H), 1.19 (s, 3H), 0.24 (s, 9H). **^{13}C NMR** (101 MHz, C_6D_6) δ 176.5, 148.5, 138.2, 130.2, 129.0 (2C), 127.75, 127.65 (2C), 127.4, 126.2, 121.6, 99.76, 99.42, 98.2, 50.8, 50.0, 27.3, 0.07 (3C).

(3*R*,3a*S*)-8-(4-Methoxyphenyl)-3-methyl-3-phenyl-3,3a-dihydro-2*H*-cyclohepta[*b*]furan-2-one (10)

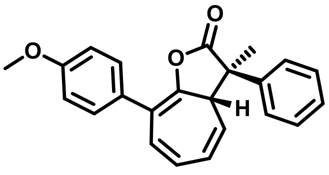

To the solution of **3a** (25 mg, 0.065 mmol, 1 equiv) in THF (324 µl, 0.2 M), (4-methoxyphenyl)boronic acid (10.8 mg, 0.071 mmol, 1.1 equiv), $PdCl_2(PPh_3)_2$ (2.3 mg, 0.003 mmol, 0.05 equiv), PPh_3 (1.7 mg, 0.006 mmol, 0.1 equiv) and Na_2CO_3 (10.3 mg, 0.097 mmol, 1.5 equiv) were added under Ar atmosphere. The reaction mixture was stirred for 21 h at 70 °C using a heating block. After complete conversion (monitored by TLC), the resulting reaction mixture was then concentrated under reduced pressure and

purified directly by flash column chromatography (5%-20% EtOAc in PE) to afford the product **10** as a yellow viscous oil in 92% yield (20.6 mg, 3.8:1 d.r.).

HRMS (ESI^+) m/z: $[M + Na]^+$ Calcd. for $C_{23}H_{20}O_3Na$ 367.1305; Found: 367.1309

Major diastereoisomer: **^{1}H NMR** (400 MHz, C_6D_6) δ 7.45 – 7.28 (m, 7H), 6.95 – 6.88 (m, 2H), 6.62 (d, *J* = 11.3 Hz, 1H), 6.55 (dd, *J* = 11.3, 5.4 Hz, 1H), 6.29 (ddd, *J* = 9.5, 5.4, 2.0 Hz, 1H), 5.58 (dd, *J* = 9.5, 4.7 Hz, 1H), 3.82 (s, 3H), 3.15 (dd, *J* = 4.7, 2.0 Hz, 1H), 1.89 (s, 3H). **^{13}C NMR** (101 MHz, C_6D_6) δ 178.2, 158.87, 143.7, 138.1, 132.0, 130.45, 129.3, 128.44, 128.16, 127.81, 127.0, 125.2, 120.3, 113.82, 113.79 (2C), 55.42, 50.0, 49.6, 21.2.

HPLC of **10** 71% ee [Chiralcel AD-H column, hexane/*i*PrOH 95:5, flow rate 1 mL/min, 25 °C, λ = 254 nm; t_R (major) = 14.1 min and t_R (minor) = 8.7 min].

Minor diastereoisomer: **^{1}H NMR** (400 MHz, C_6D_6) δ 7.45 – 7.28 (m, 7H), 6.95 – 6.88 (m, 2H), 6.67 (d, *J* = 11.3 Hz, 1H), 6.55 (dd, *J* = 11.3, 5.4 Hz, 1H), 6.00 (ddd, *J* = 9.5, 5.4, 1.9 Hz, 1H), 4.62 (dd, *J* = 9.4, 4.8 Hz, 1H), 3.84 (s, 3H), 2.93 (dd, *J* = 4.8, 1.9 Hz, 1H), 1.93 (s, 3H). **^{13}C NMR** (101 MHz, $CDCl_3$) δ 178.1, 158.89, 138.1, 137.3, 131.6, 130.49, 129.0, 128.83, 128.8, 127.75, 127.6, 125.77, 122.3, 114.21, 114.19 (2C), 55.44, 50.8, 50.2, 29.1.

Carbon signals for 3 aromatic carbon atoms (in the range between 127–129.3 ppm) cannot be assigned as compound contains two many aromatic signals in addition to mixture of diastereoisomers.

HPLC of **10** 43% ee [Chiralcel AD-H column, hexane/*i*PrOH 95:5, flow rate 1 mL/min, 25 °C, λ = 254 nm; t_R (major) = 7.7 min and t_R (minor) = 10.6 min].

8. NMR

^{1}H and ^{13}C NMR spectra of newly synthesized diazoketones 2

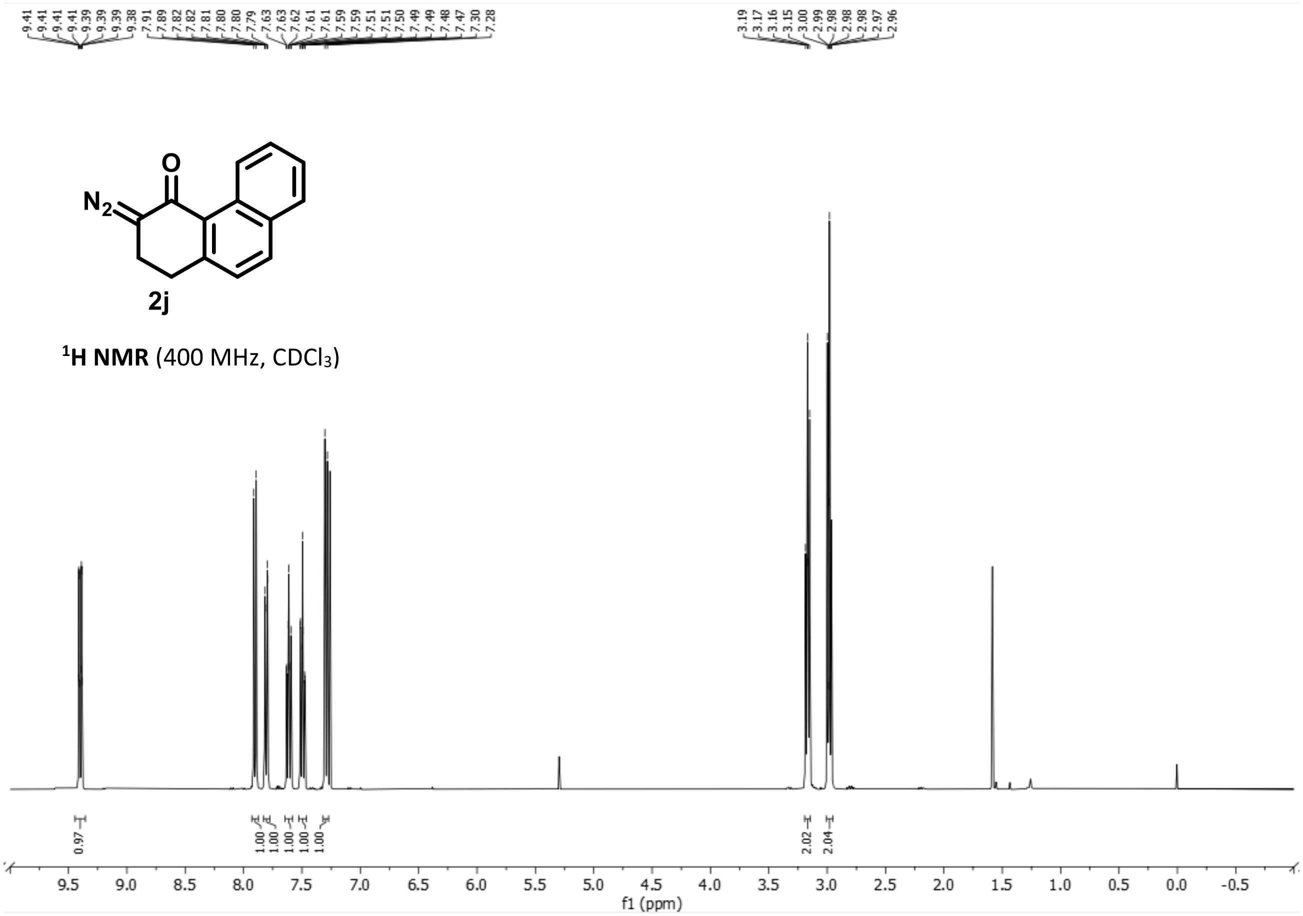

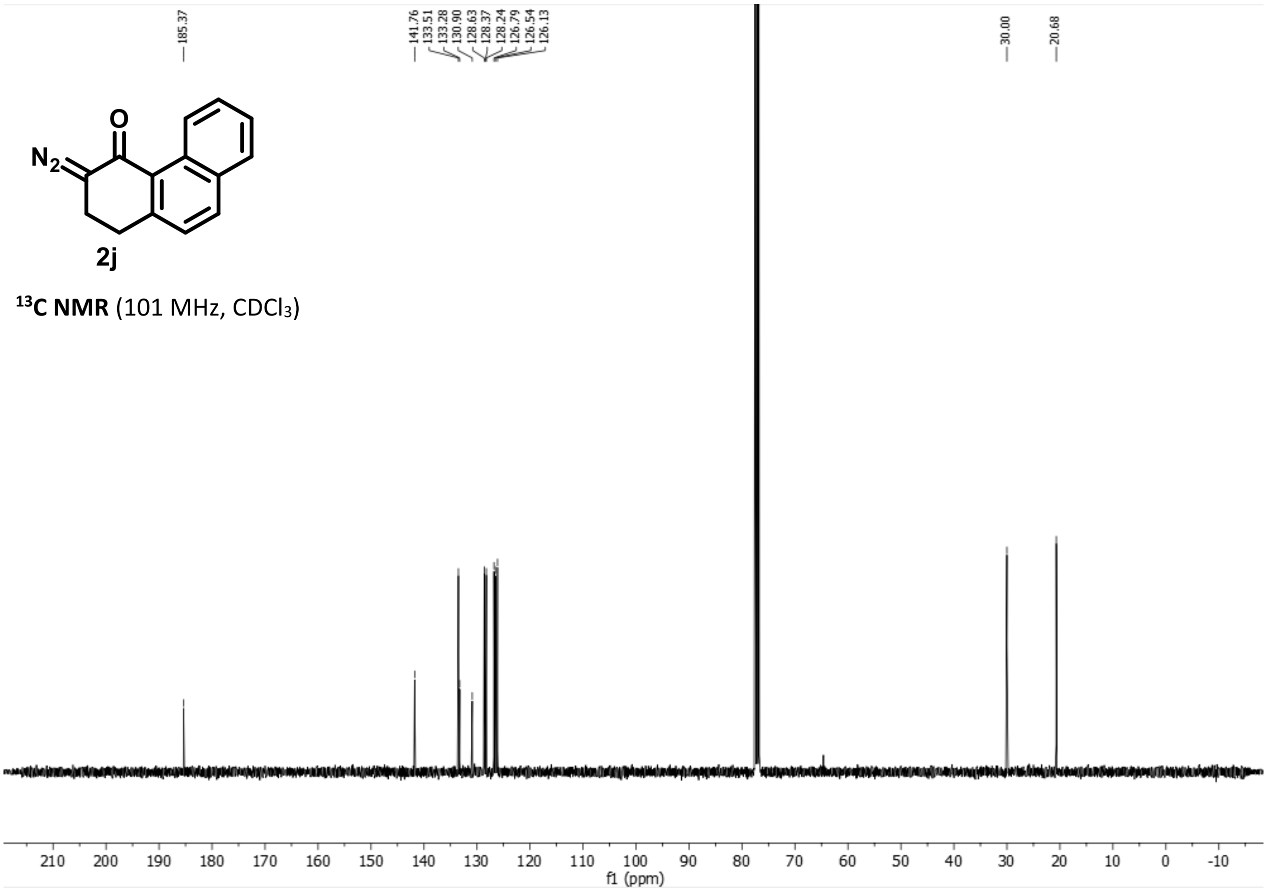

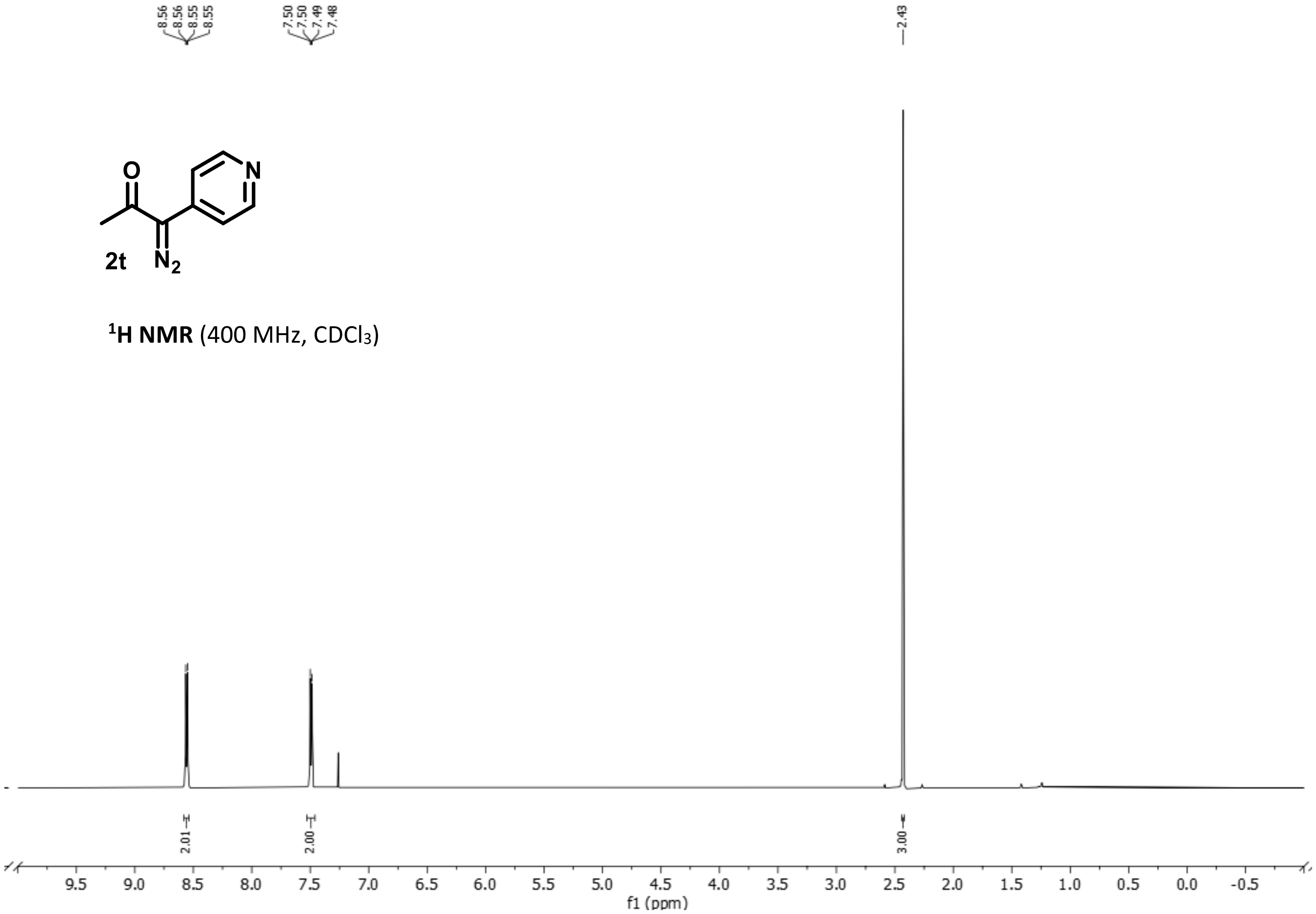

^{1}H NMR (400 MHz, $CDCl_3$)

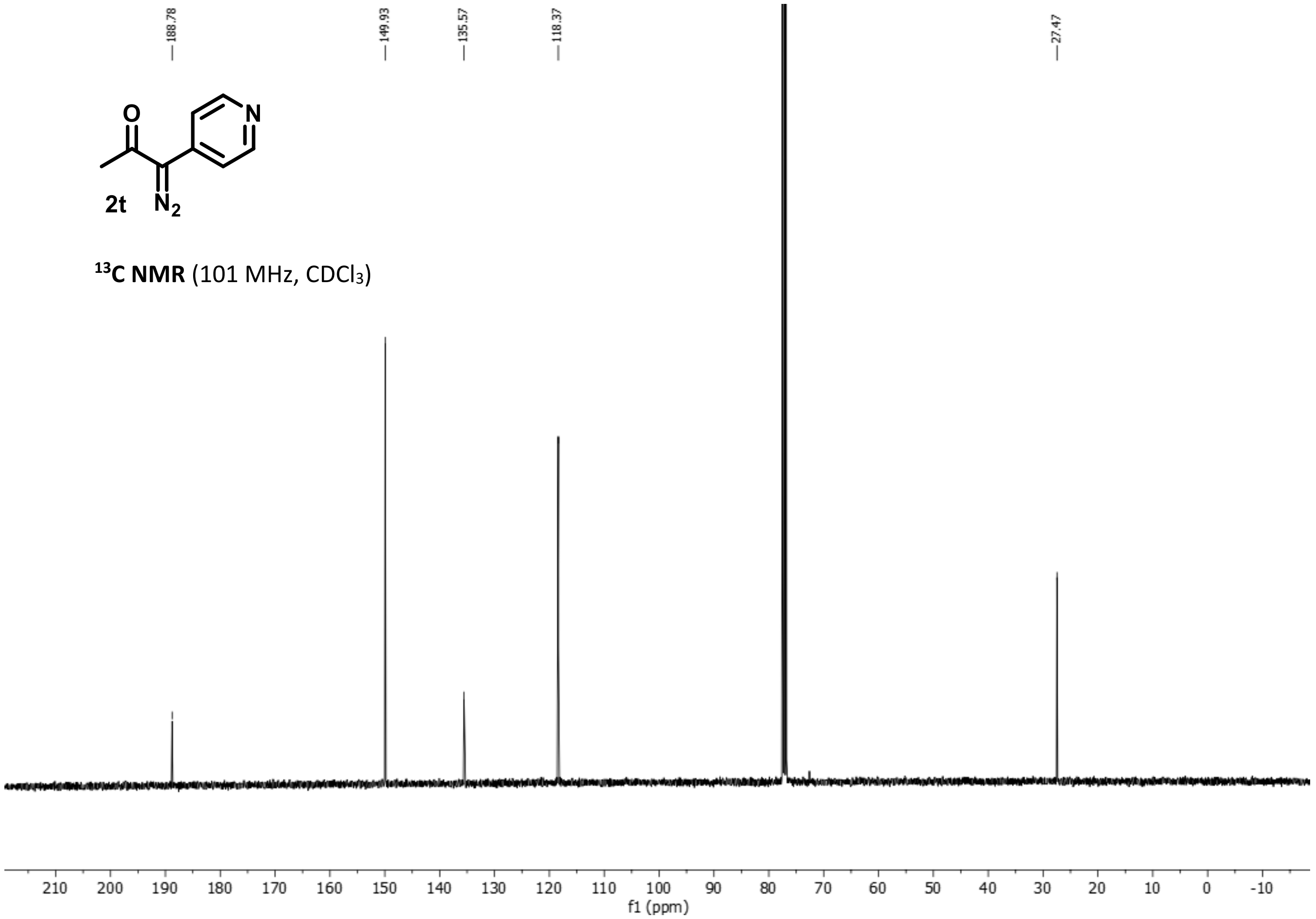

^{13}C NMR (101 MHz, $CDCl_3$)

^{1}H and ^{13}C NMR spectra of tropolone derivative 1e

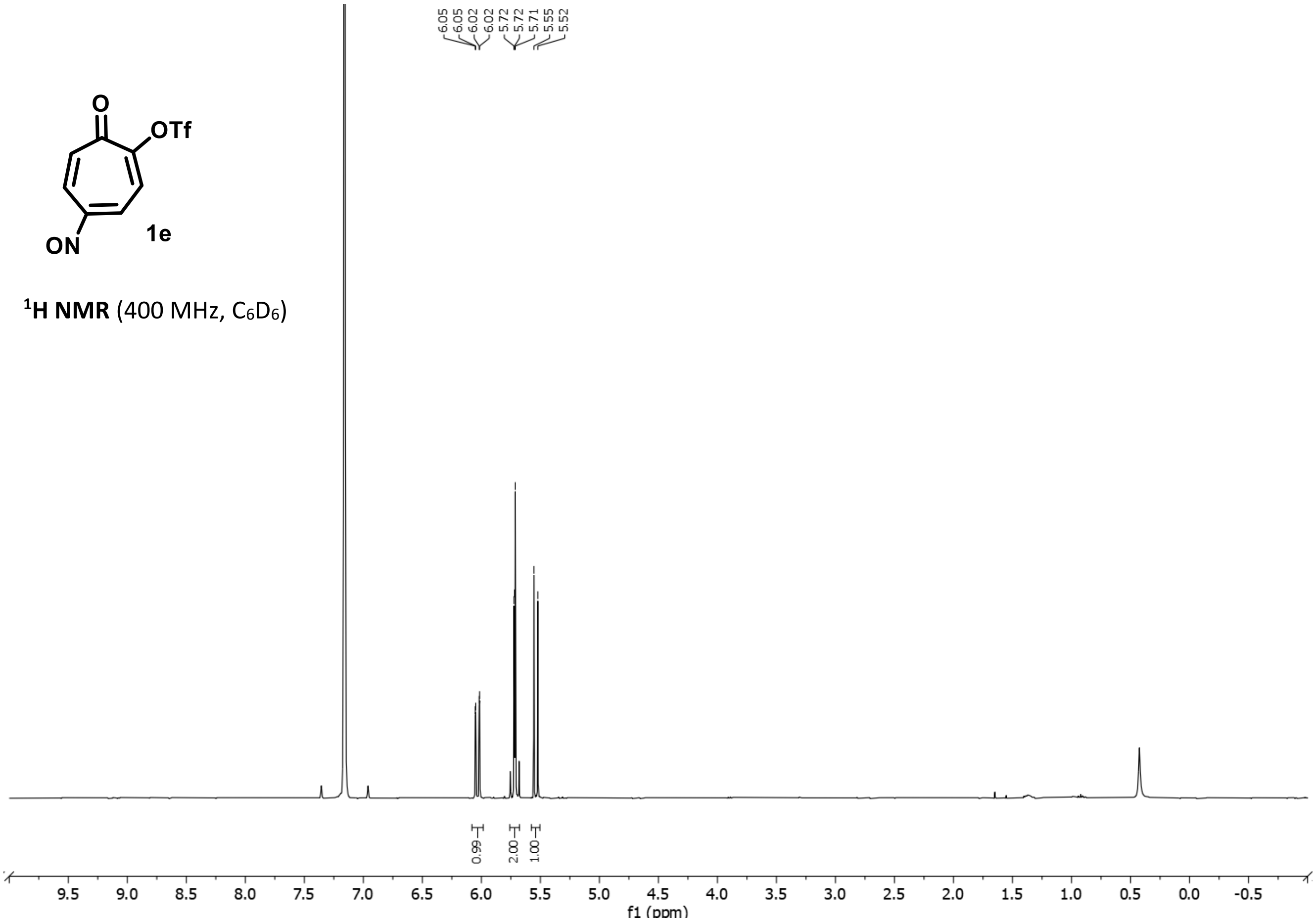

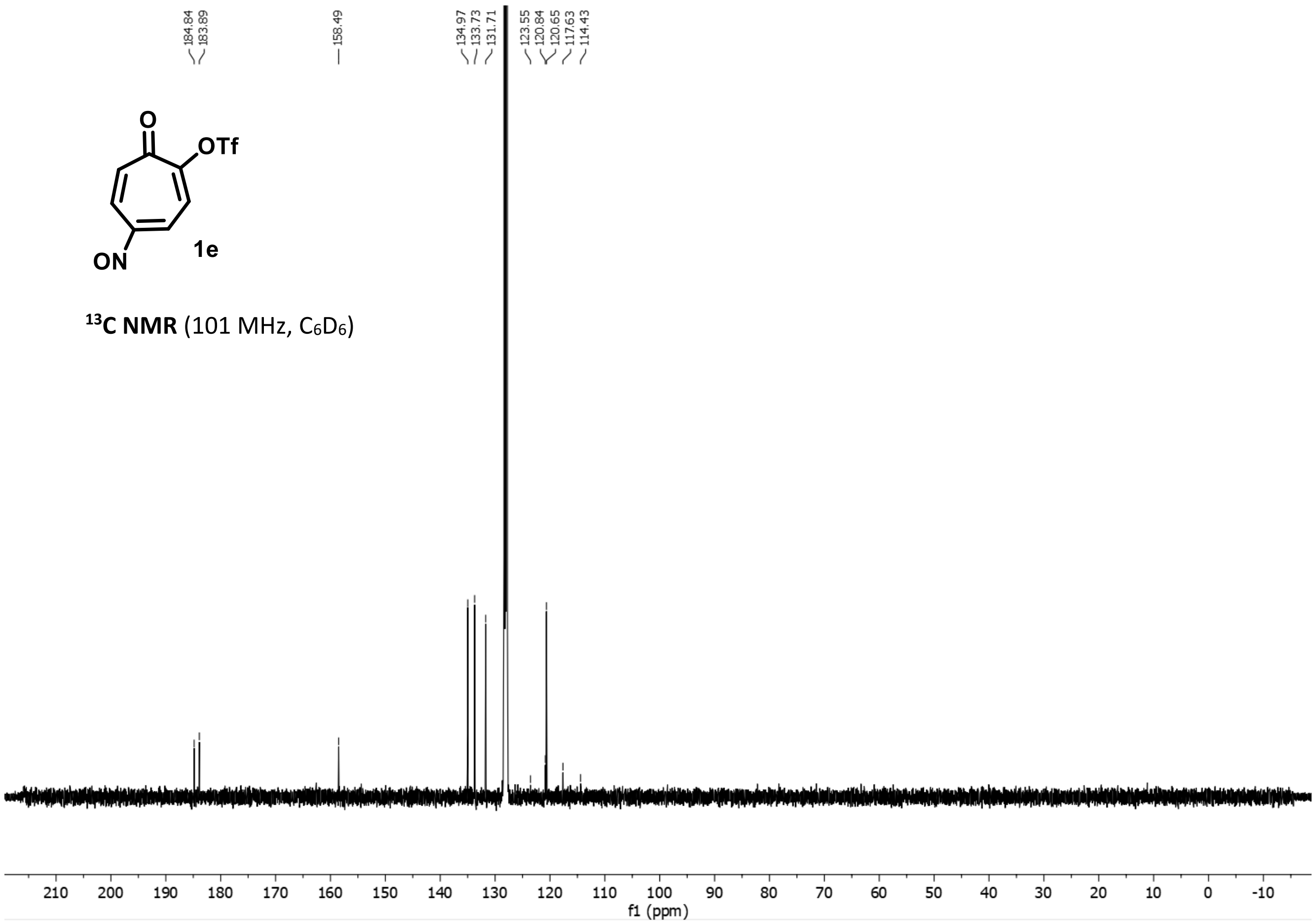

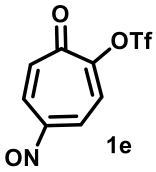

1e

^{19}F NMR (376 MHz, C_6D_6)

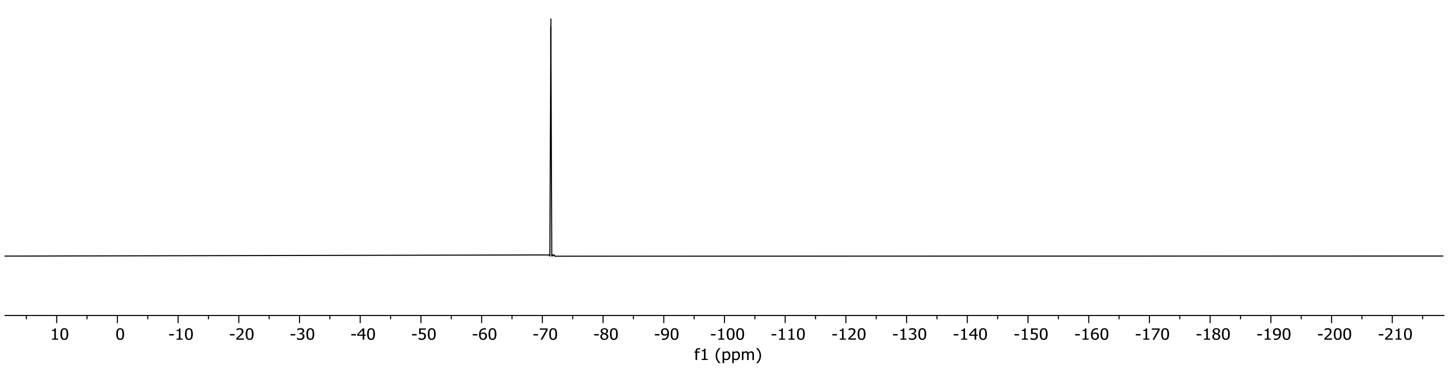

^{1}H and ^{13}C NMR spectra of products 3 and derivatizations products 5-10

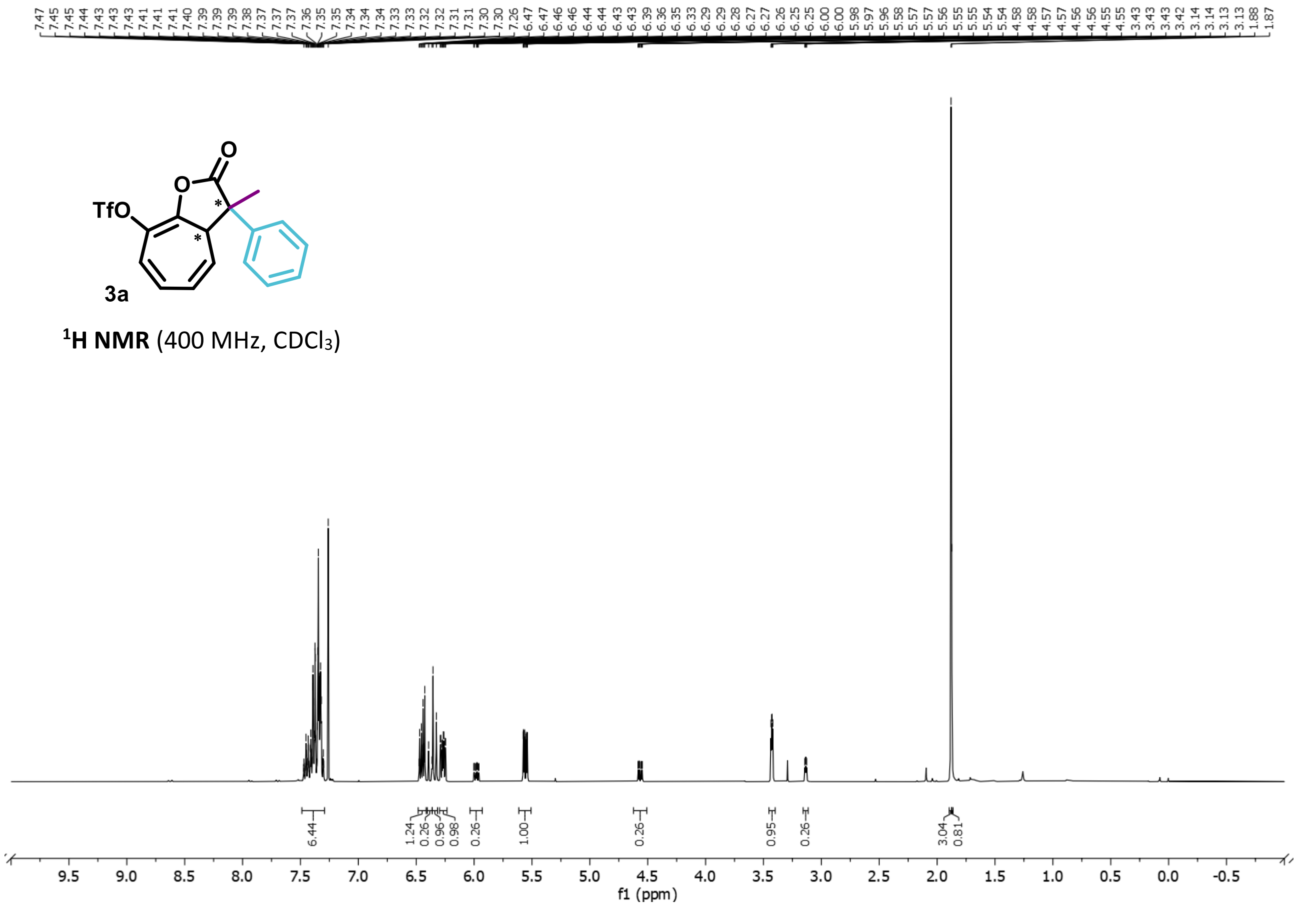

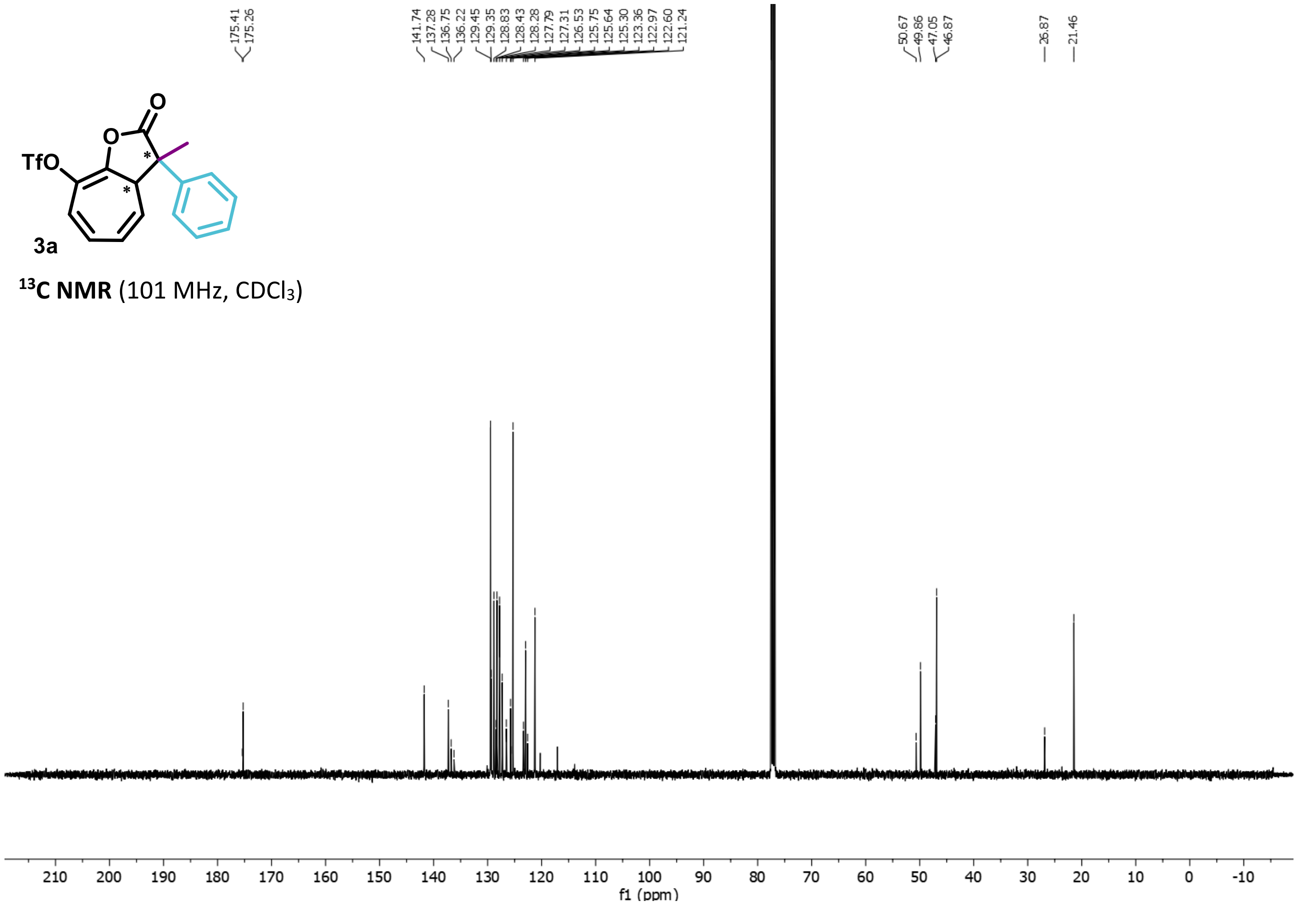

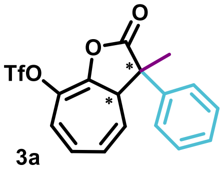

3a

^{19}F NMR (376 MHz, $CDCl_3$)

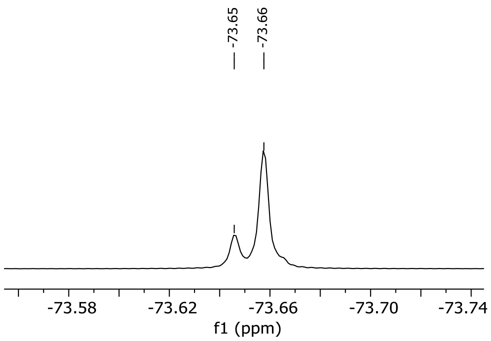

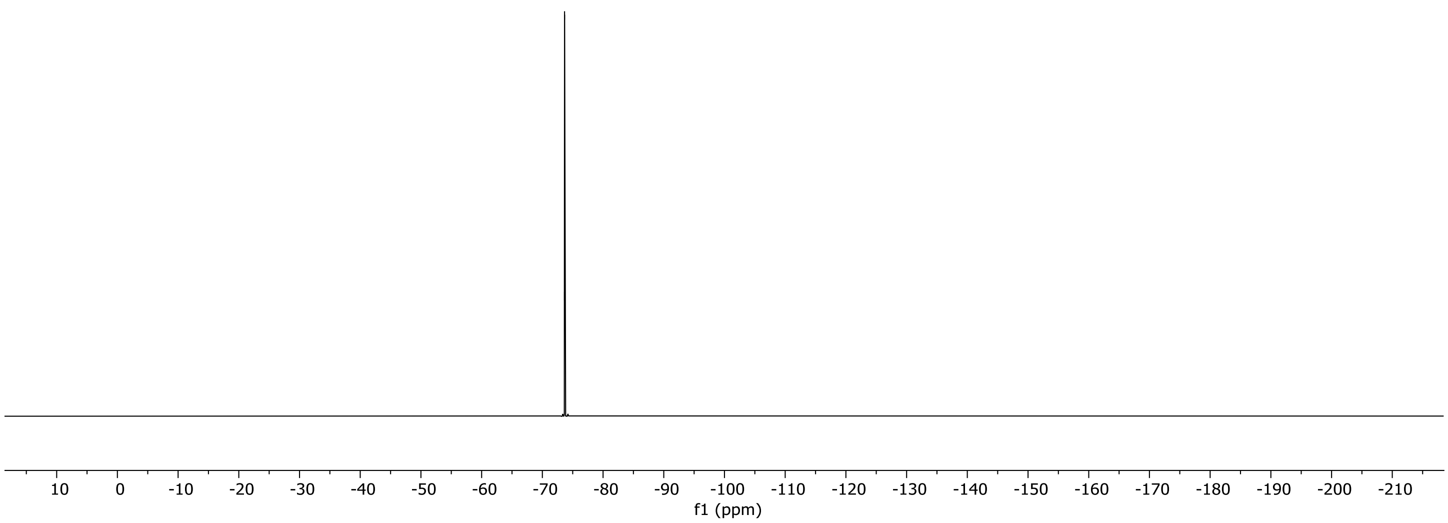

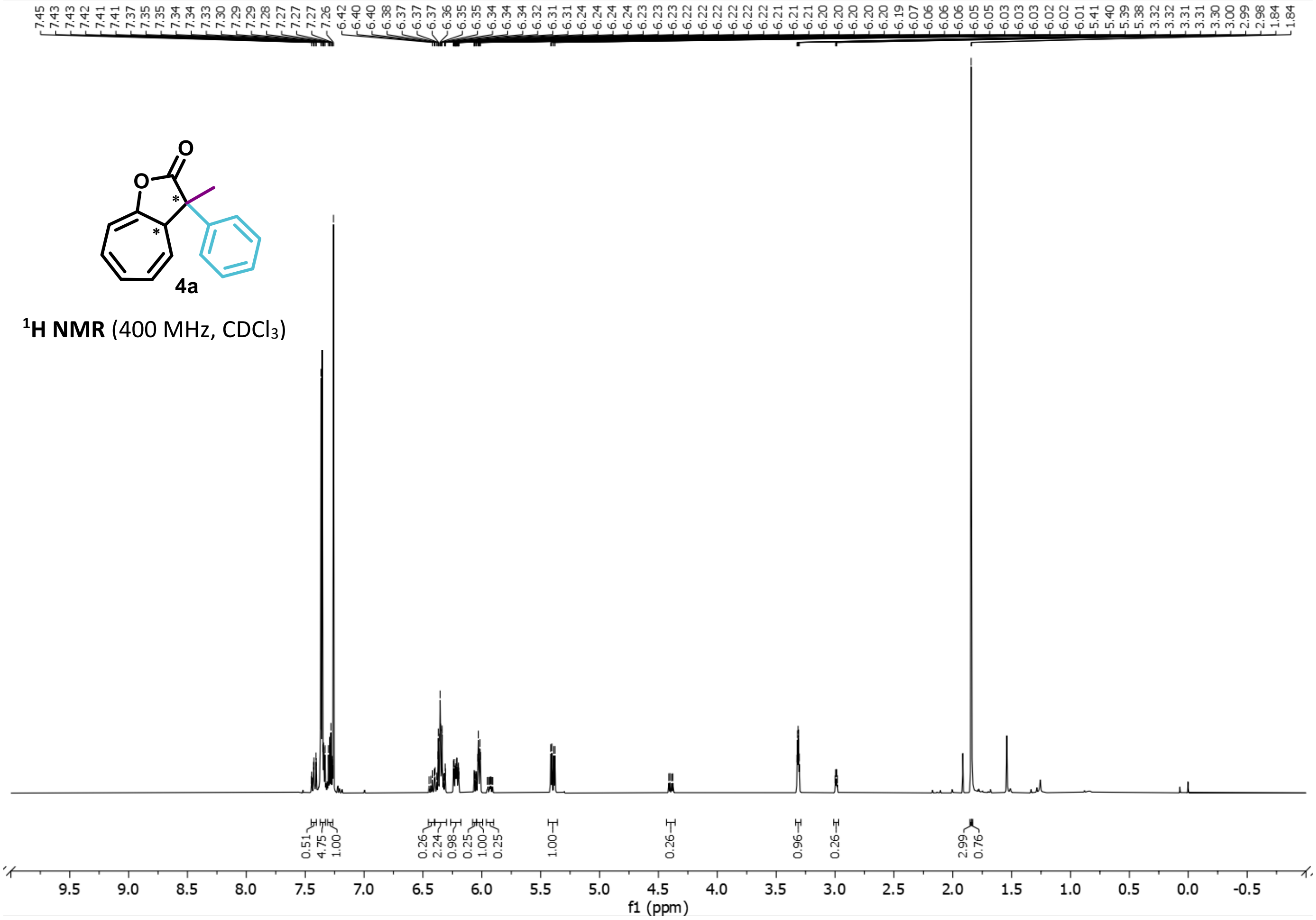
4a
1H NMR (400 MHz, CDCl3)
f1 (ppm)

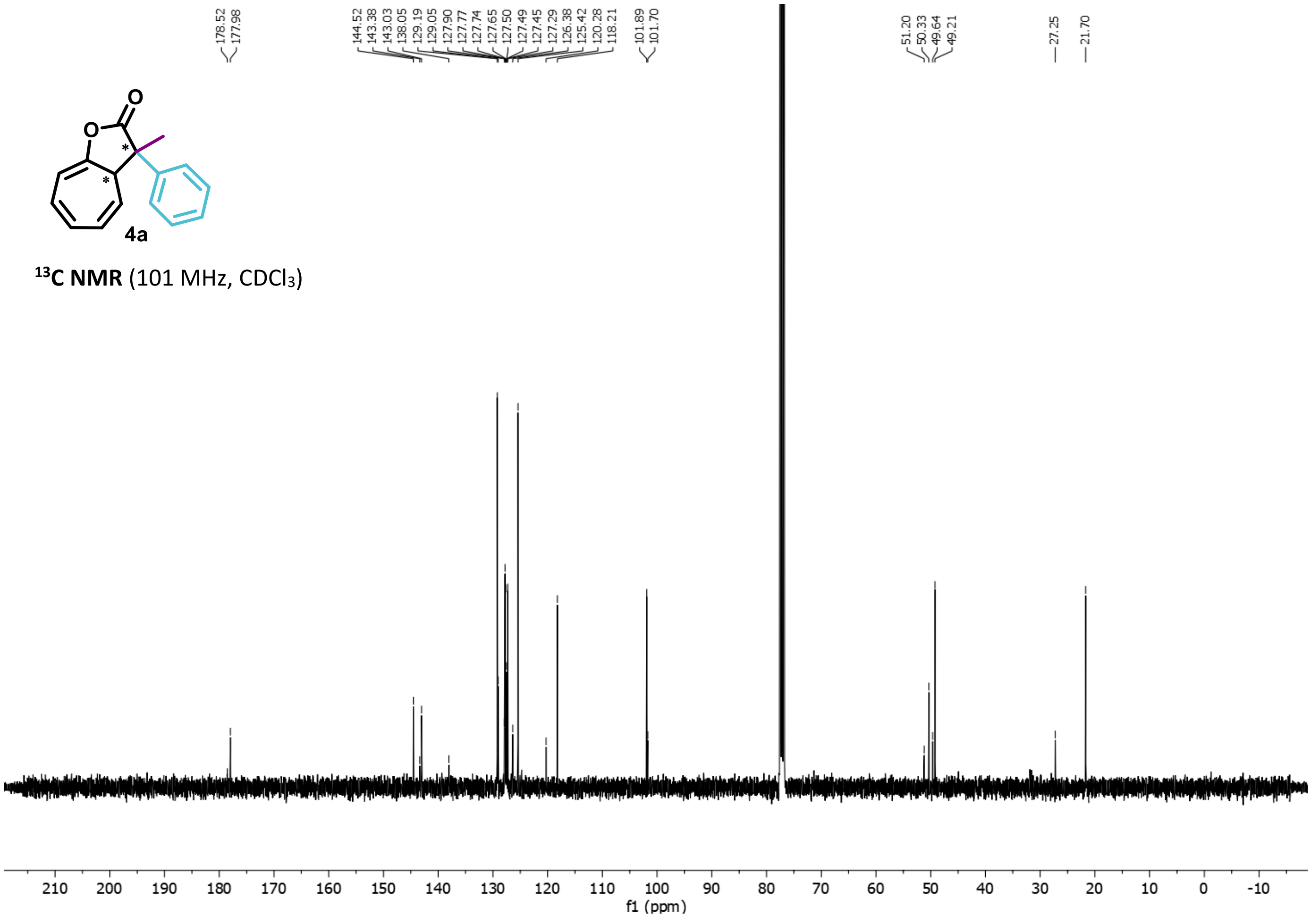
4a
13C NMR (101 MHz, CDCl3)
f1 (ppm)

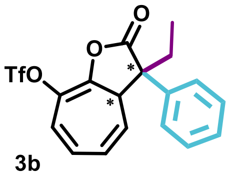

3b

^{1}H NMR (400 MHz, C_6D_6)

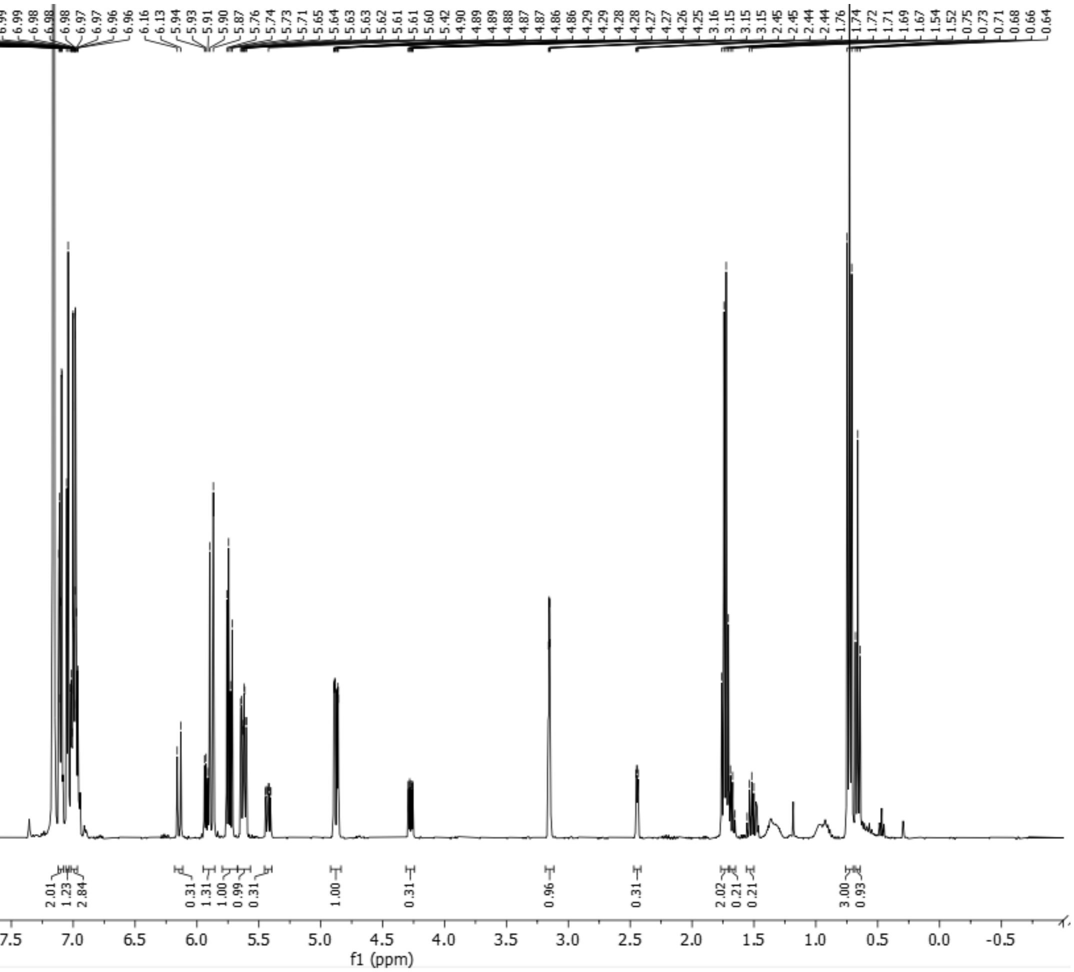

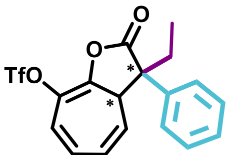

3b

^{13}C NMR (101 MHz, C_6D_6)

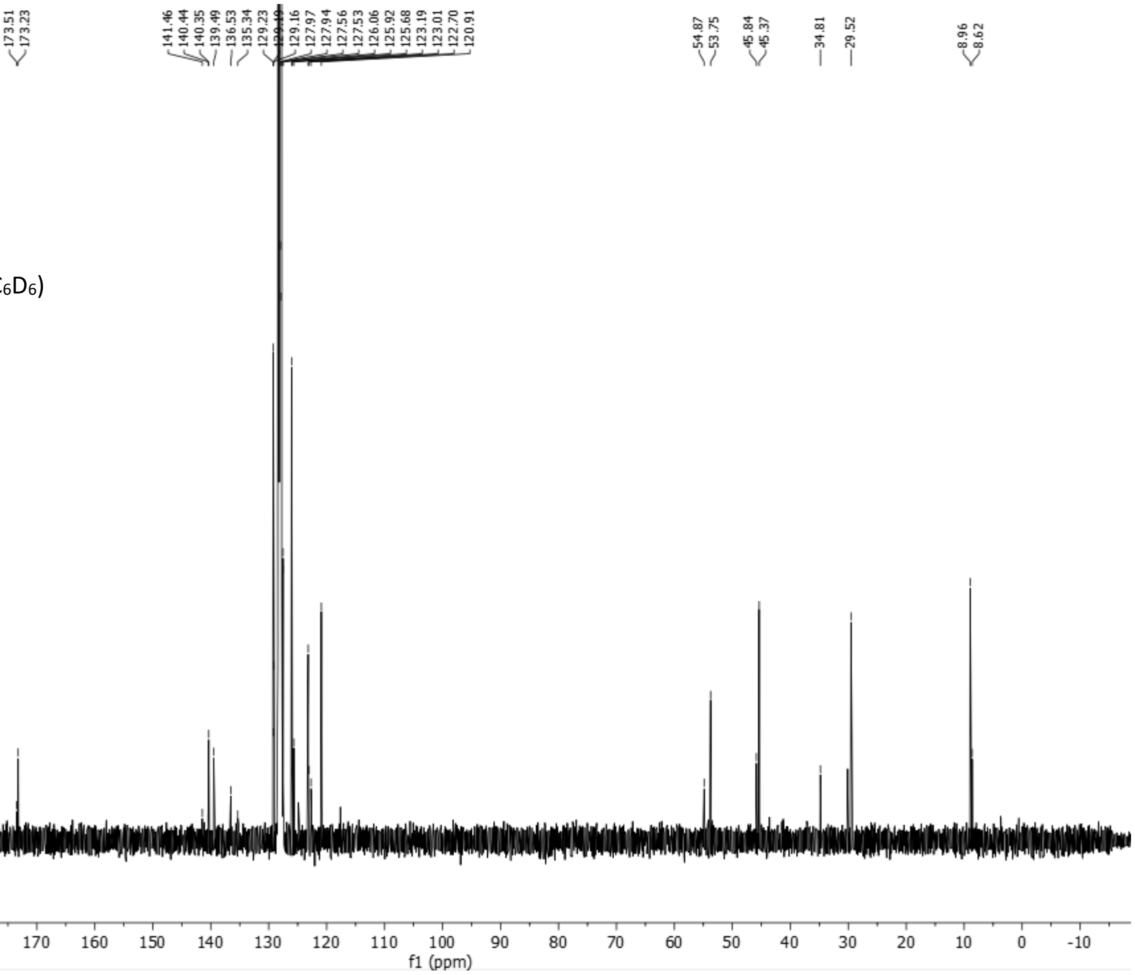

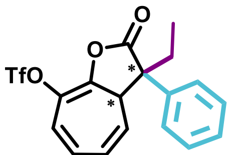

3b

^{19}F NMR (376 MHz, C_6D_6)

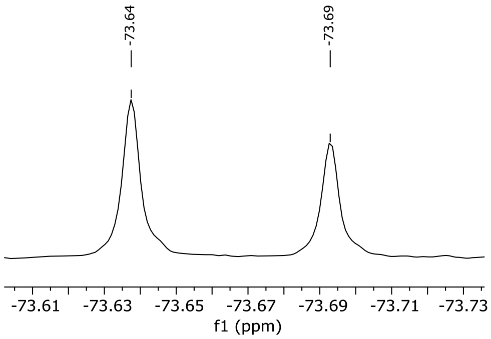

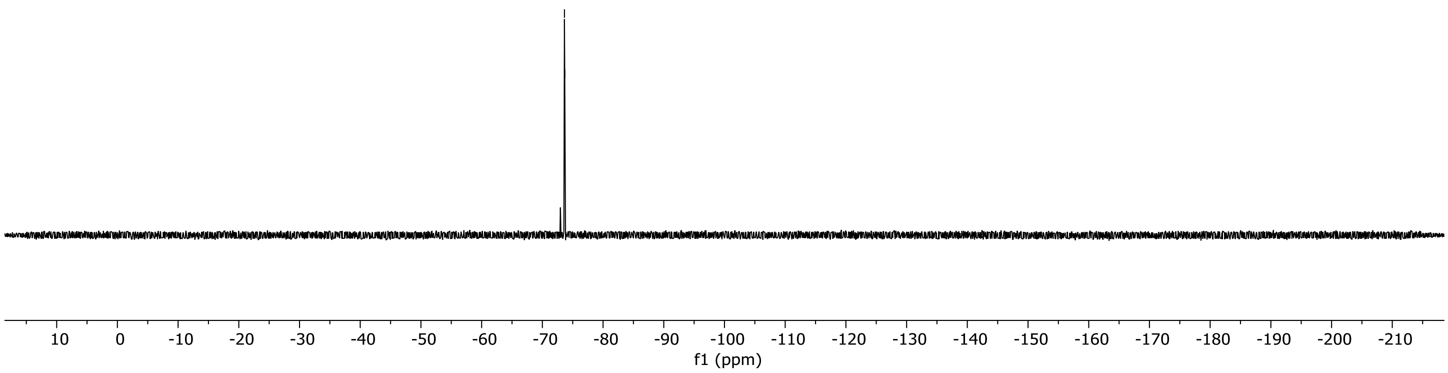

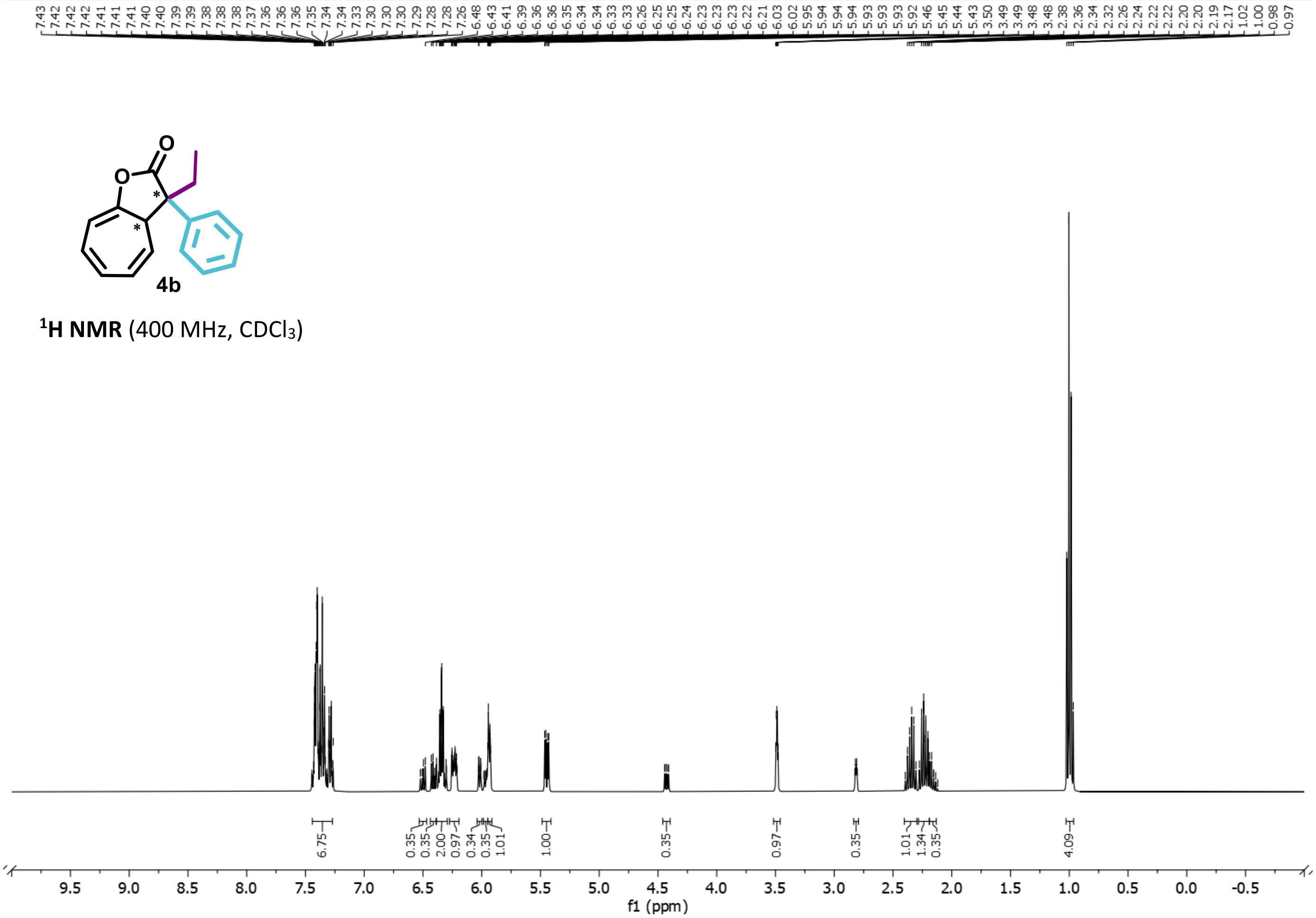

O
4b
1H NMR (400 MHz, CDCl3)
f1 (ppm)

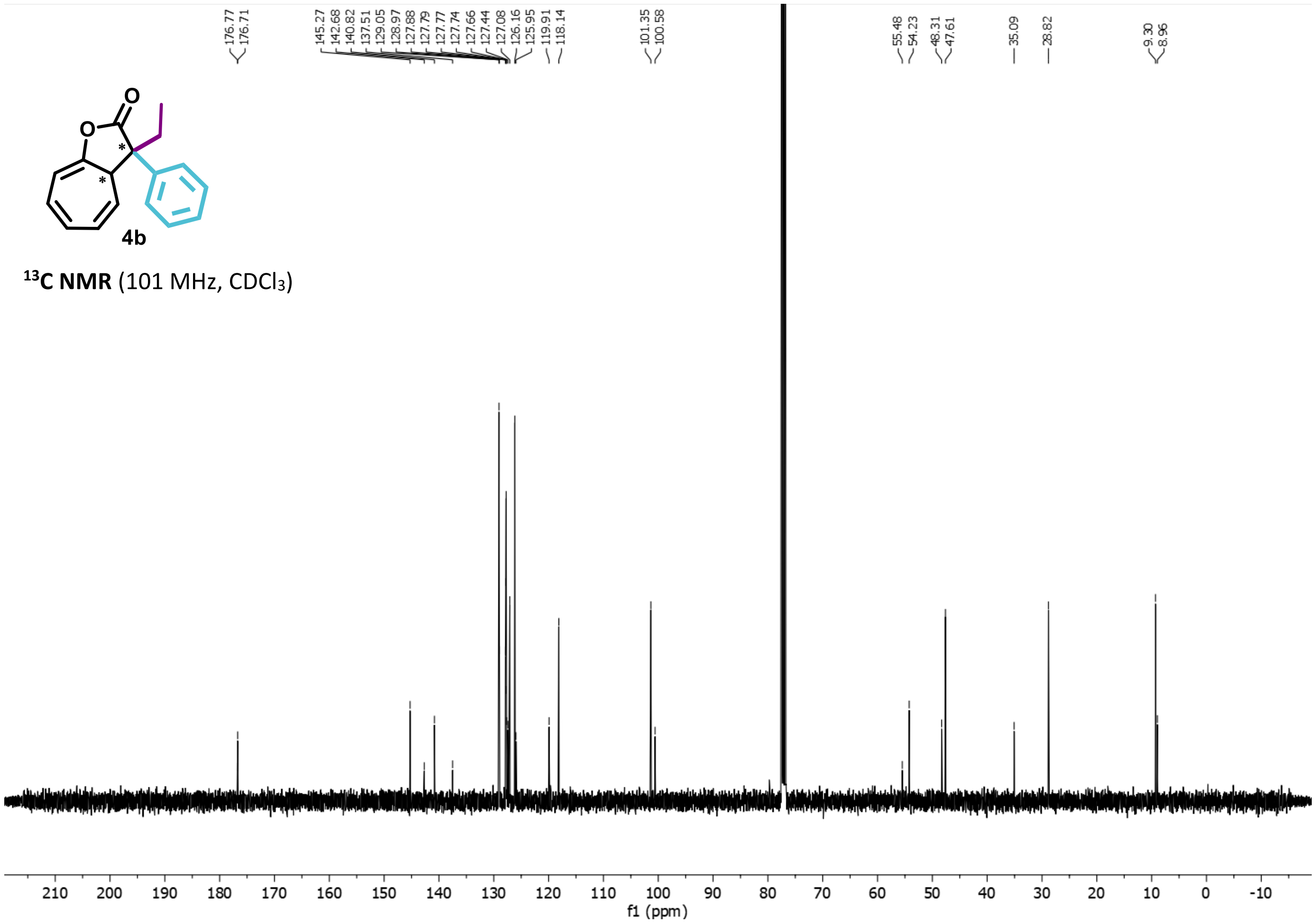

O
4b
13C NMR (101 MHz, CDCl3)
f1 (ppm)

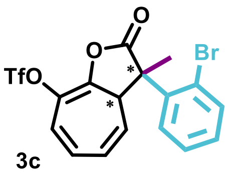

3c

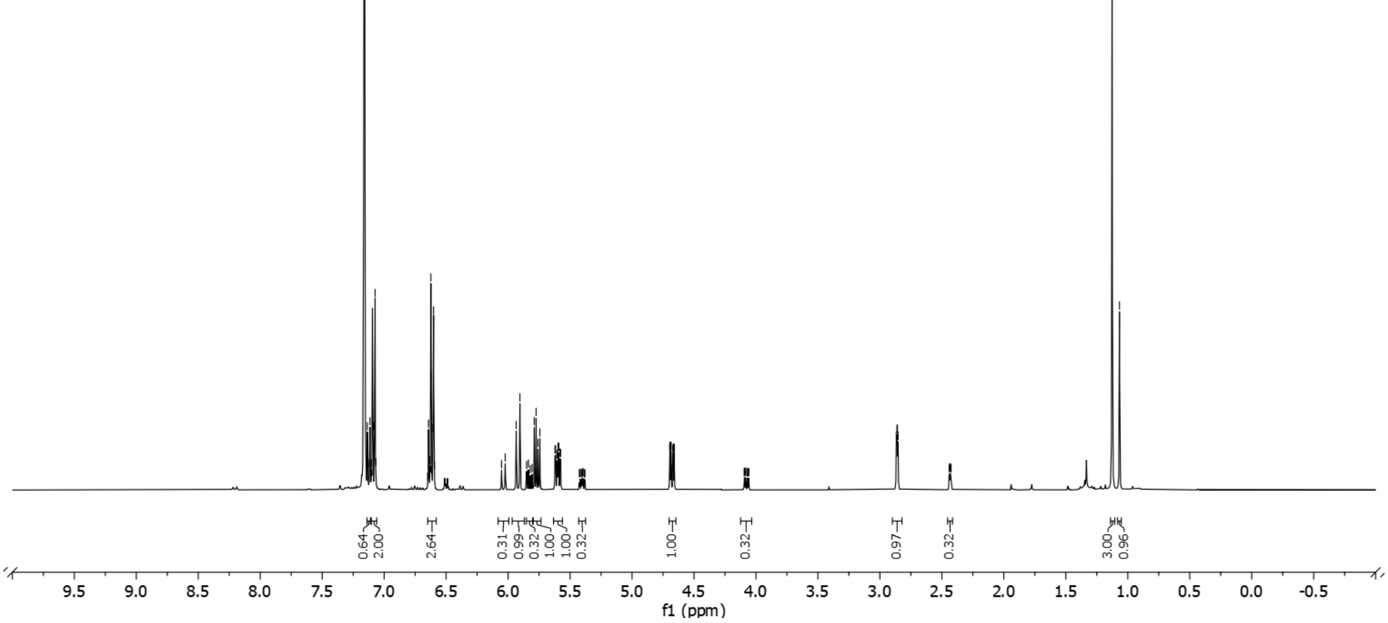

^{1}H NMR (400 MHz, C_6D_6)

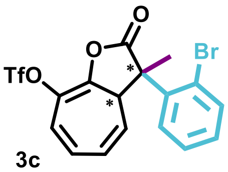

3c

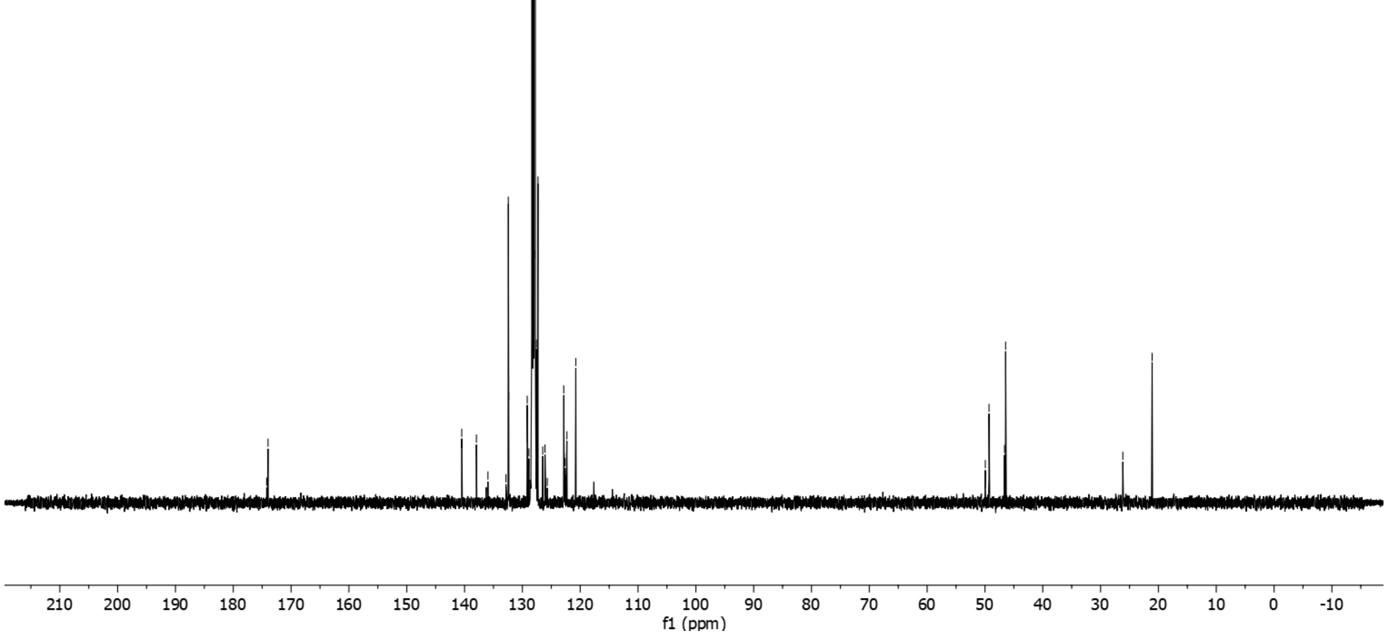

^{13}C NMR (101 MHz, C_6D_6)

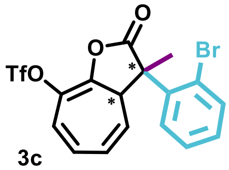

3c

^{19}F NMR (376 MHz, C_6D_6)

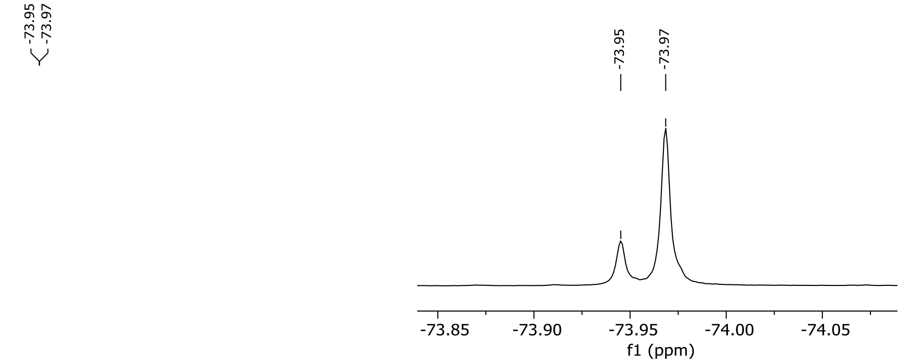

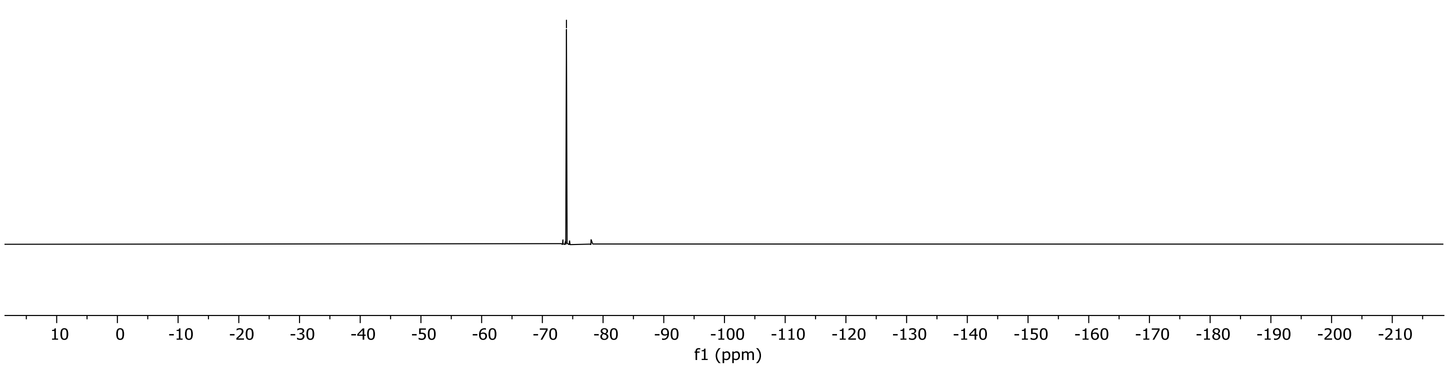

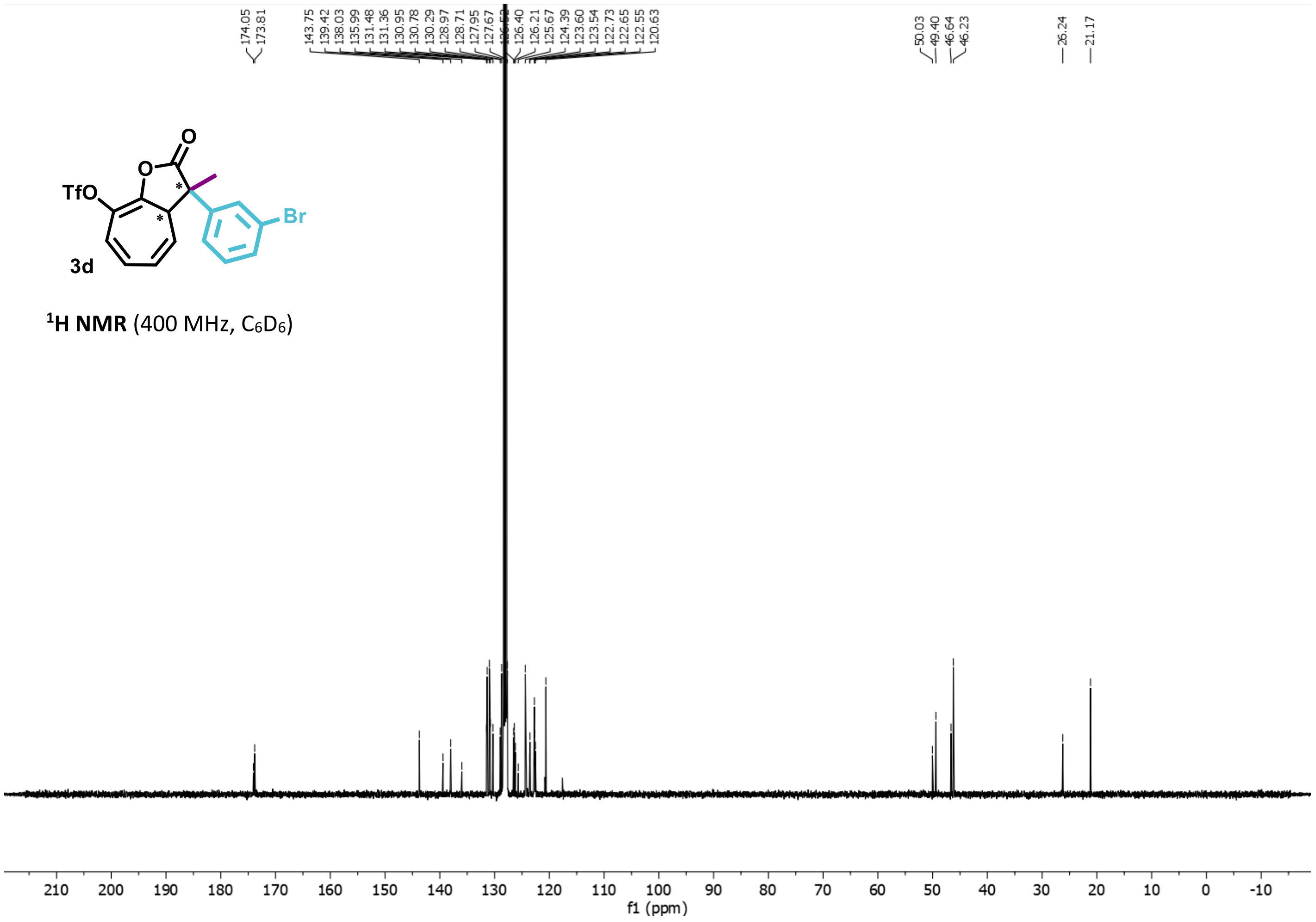

^{1}H NMR (400 MHz, C_6D_6)

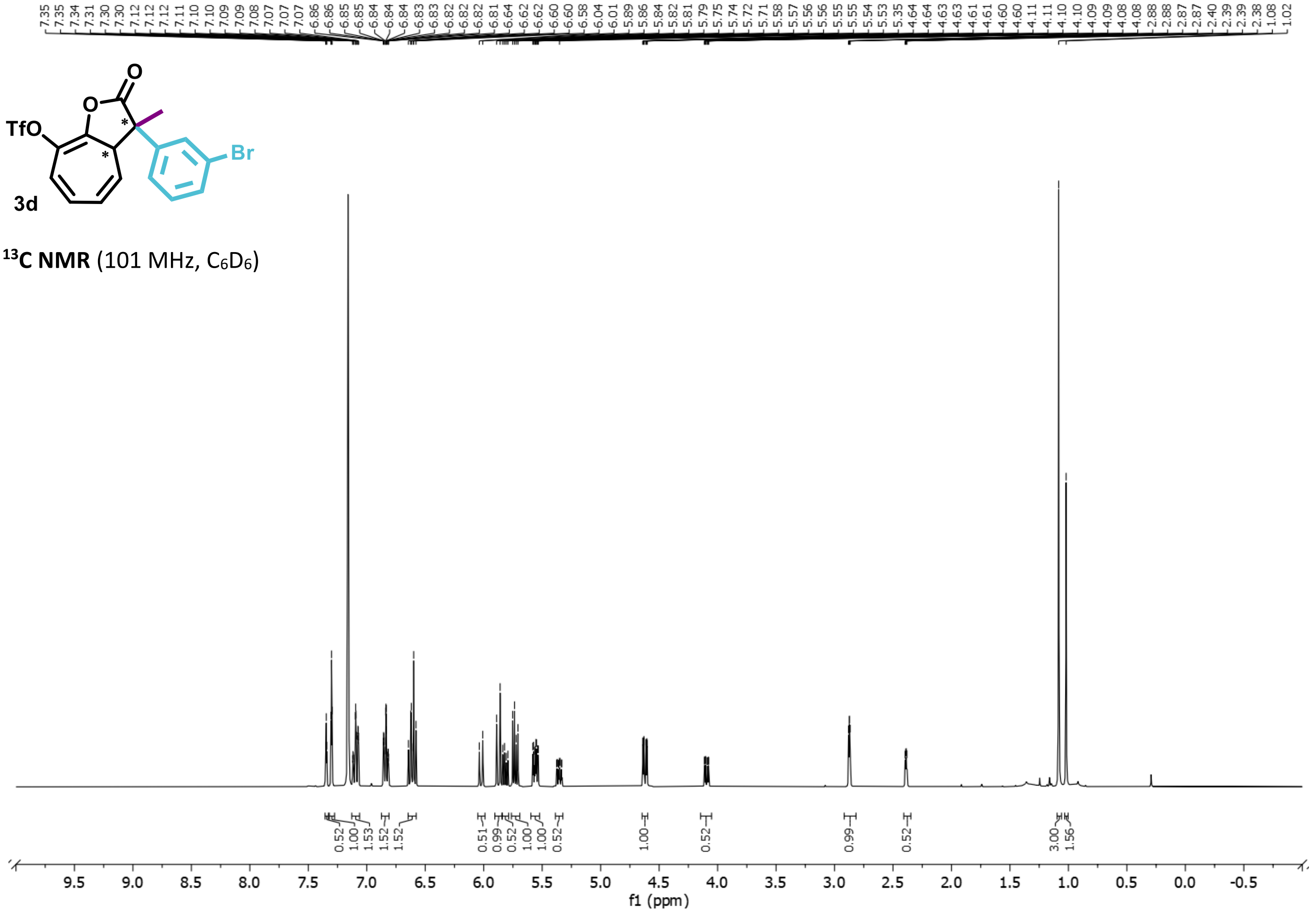

^{13}C NMR (101 MHz, C_6D_6)

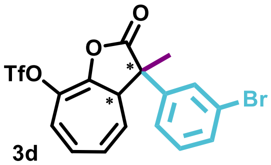

3d

^{19}F NMR (376 MHz, C_6D_6)

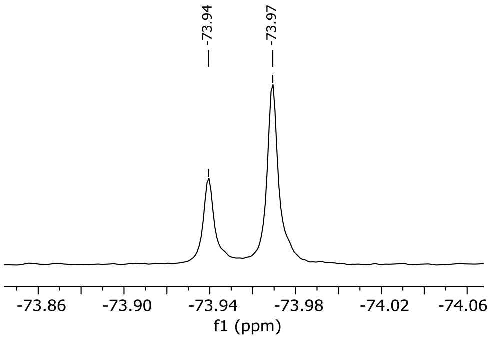

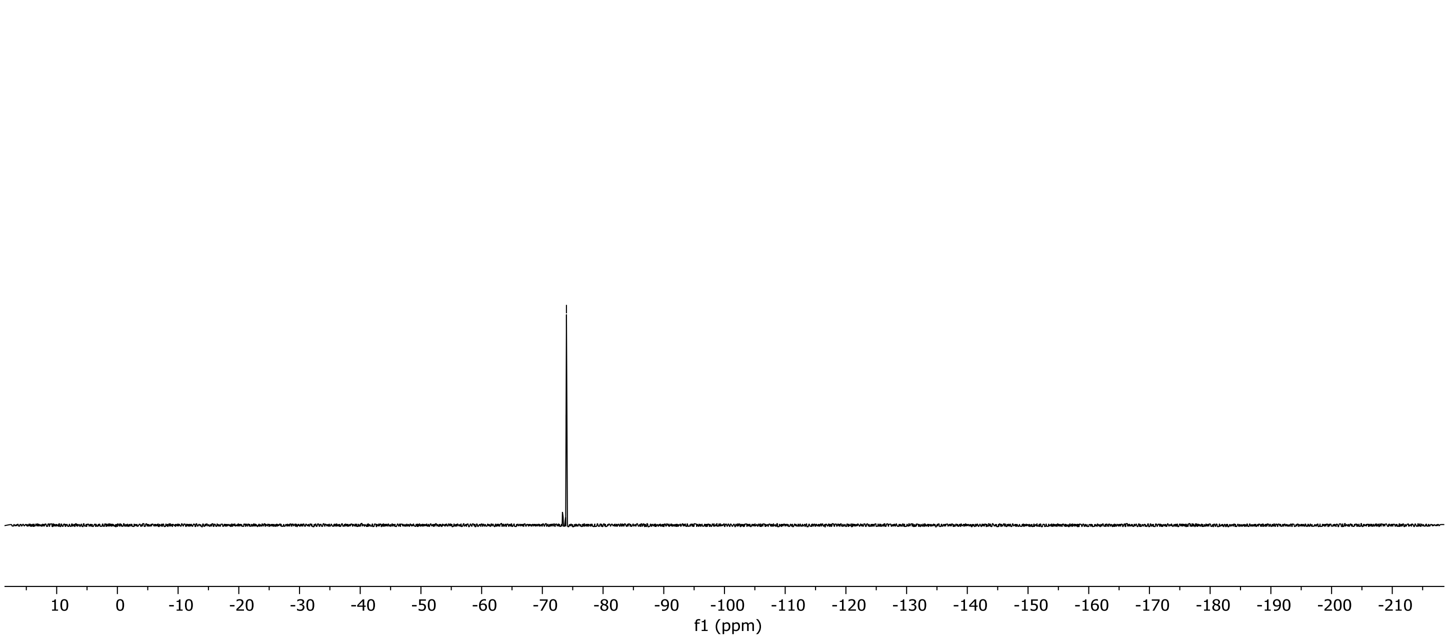

3e

^{1}H NMR (400 MHz, C_6D_6)

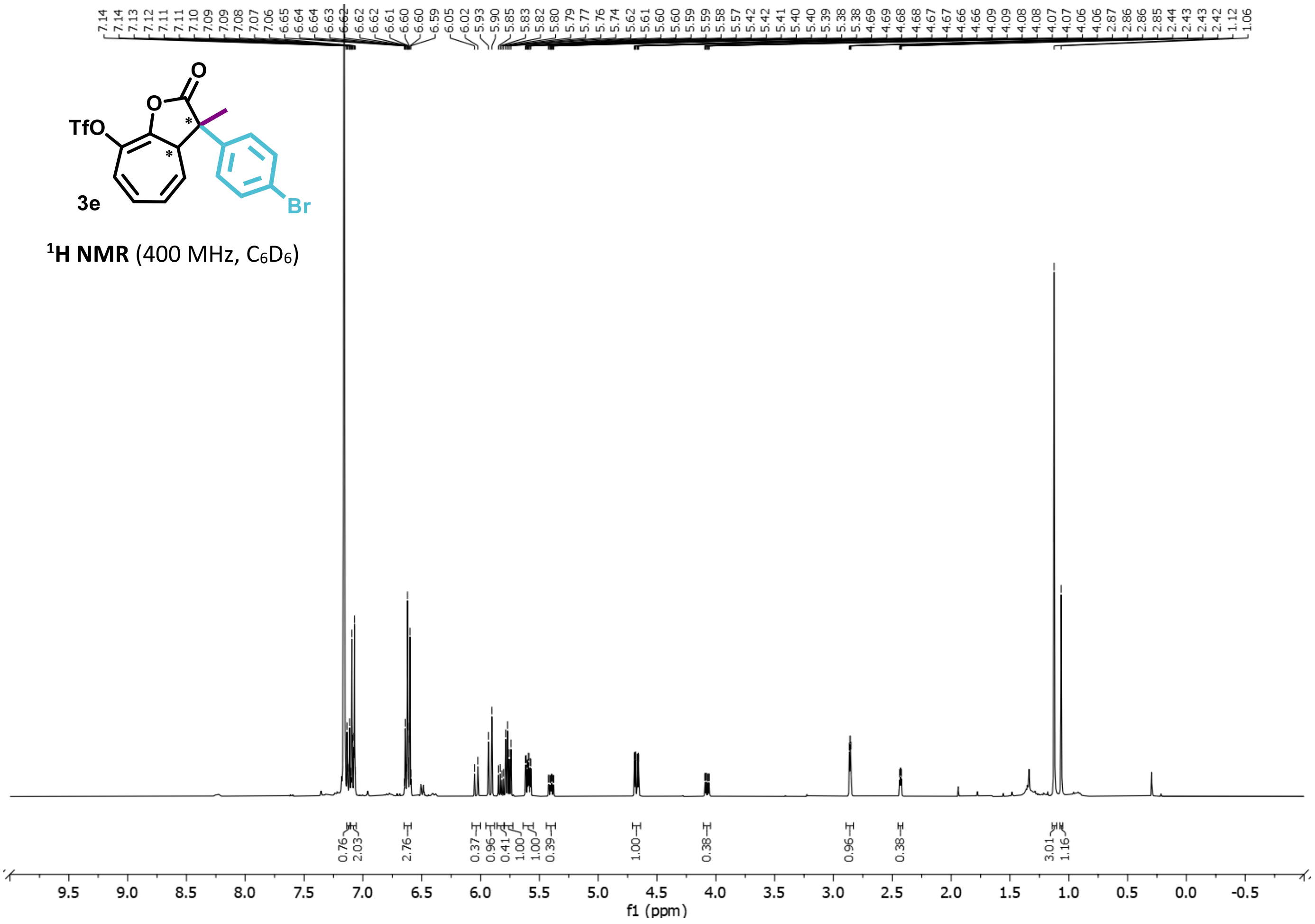

3e

^{13}C NMR (101 MHz, C_6D_6)

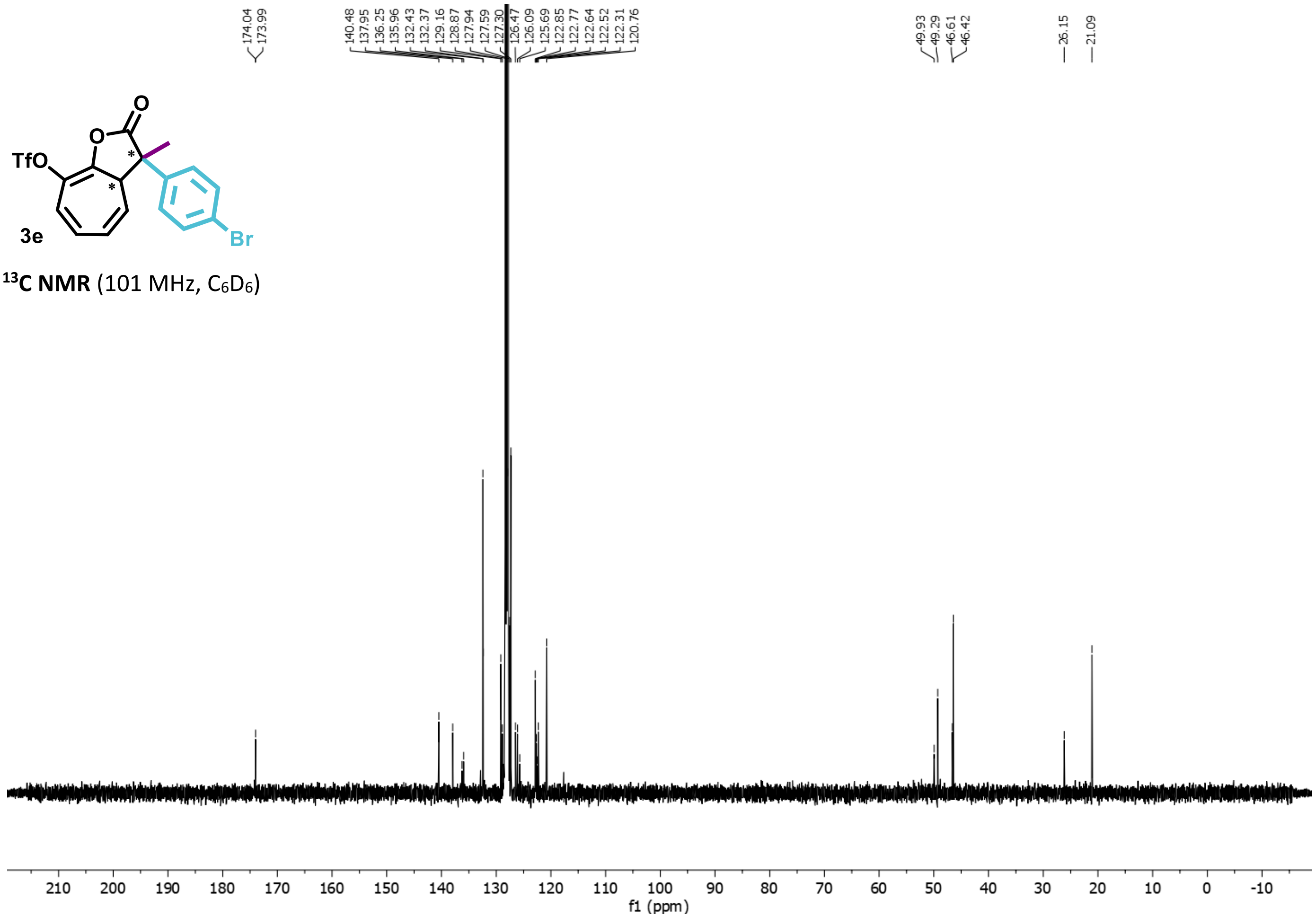

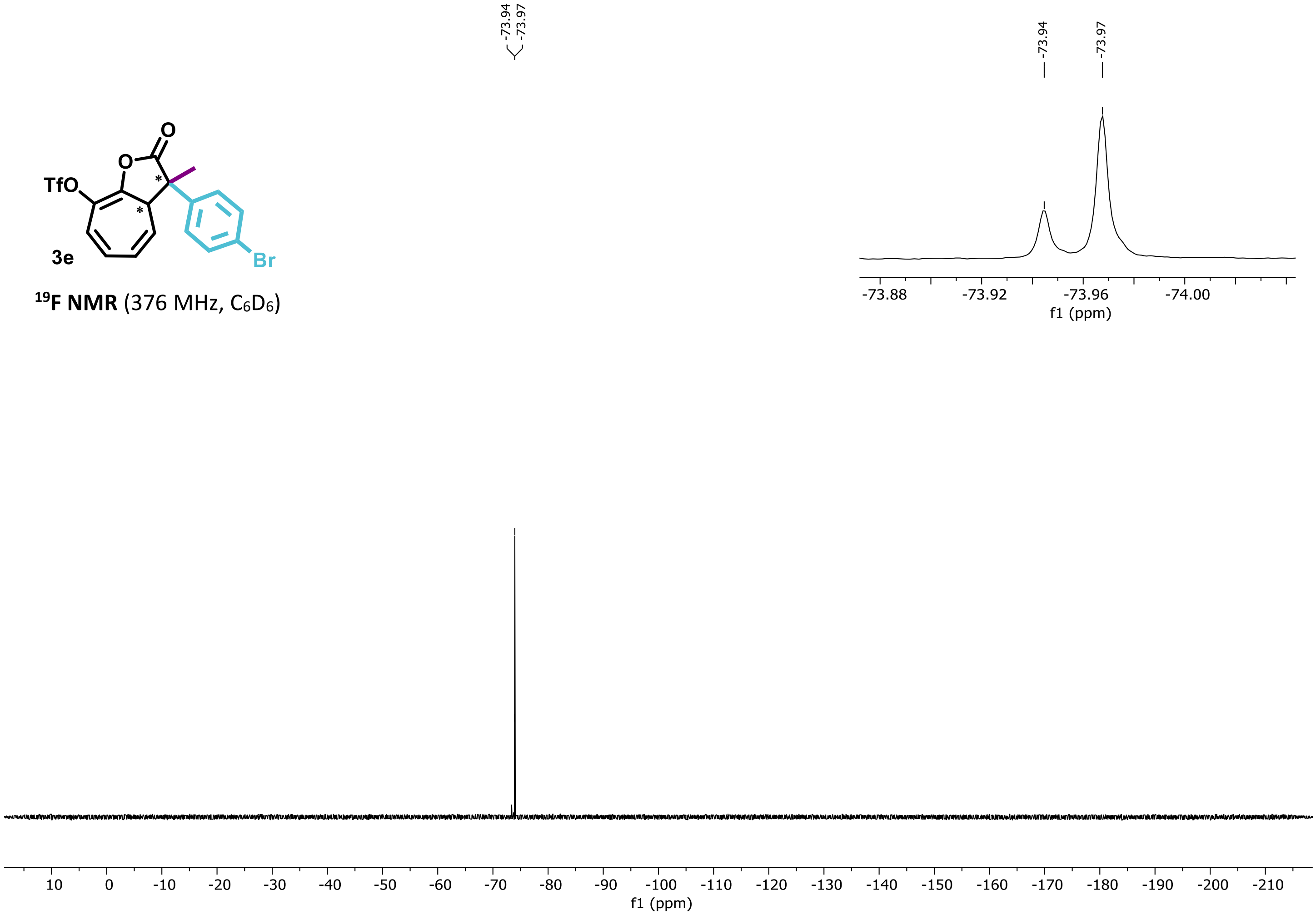

^{19}F NMR (376 MHz, C_6D_6)

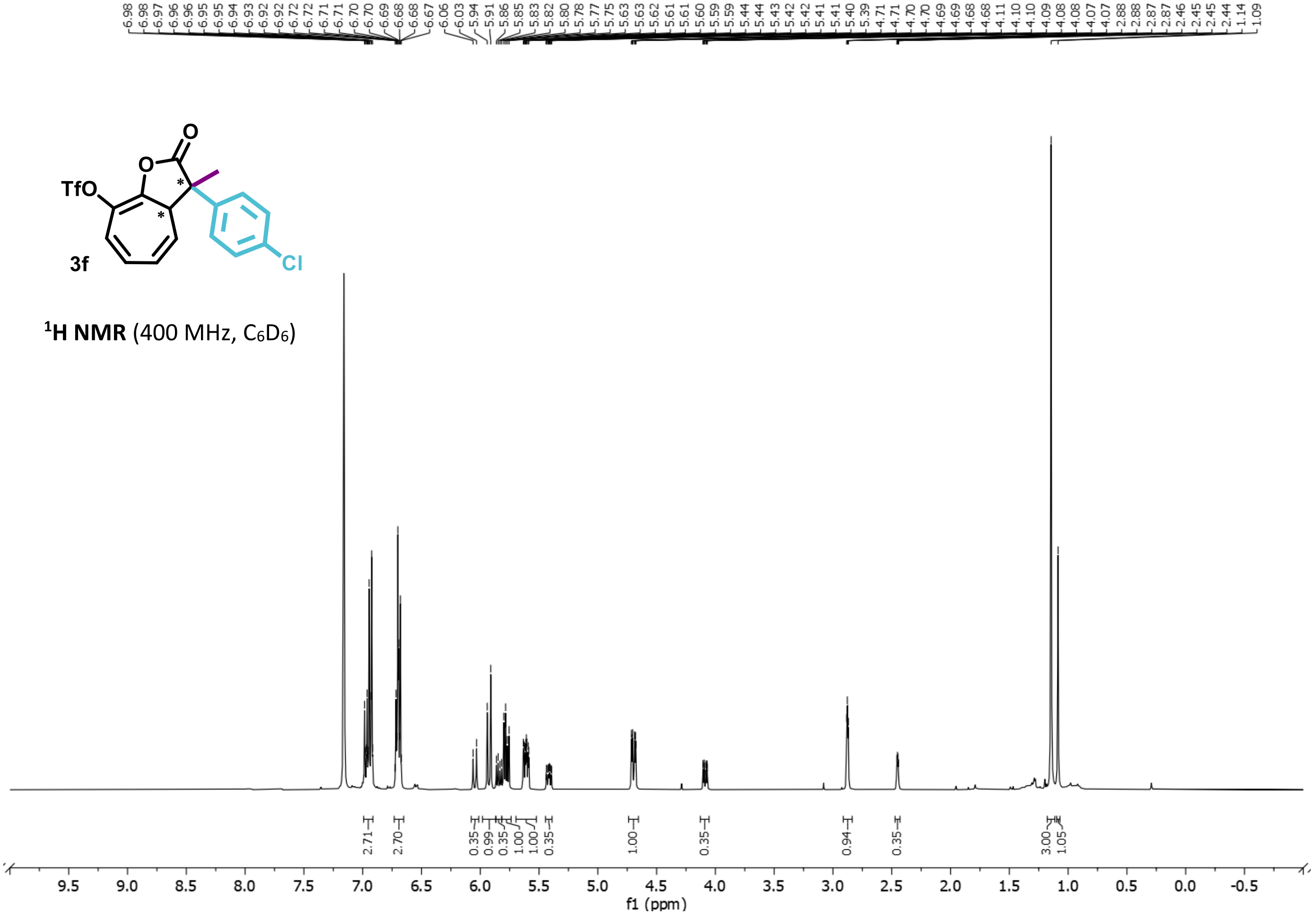
TfO
O
O
3f
Cl
¹H NMR (400 MHz, C₆D₆)
6.98 6.98 6.97 6.96 6.96 6.95 6.94 6.93 6.92 6.92 6.72 6.72 6.71 6.71 6.70 6.70 6.69 6.68 6.68 6.67 6.06 6.03 5.94 5.91 5.86 5.85 5.83 5.82 5.80 5.78 5.77 5.75 5.63 5.63 5.62 5.61 5.61 5.60 5.59 5.59 5.44 5.44 5.43 5.42 5.41 5.41 5.40 5.39 4.71 4.71 4.70 4.70 4.69 4.69 4.68 4.68 4.11 4.10 4.10 4.09 4.08 4.08 4.07 4.07 2.88 2.88 2.87 2.87 2.46 2.45 2.45 2.44 1.14 1.09
2.71 2.70 0.35 0.99 0.35 1.00 1.00 0.35 1.00 0.35 0.94 0.35 3.00 1.05
f1 (ppm)

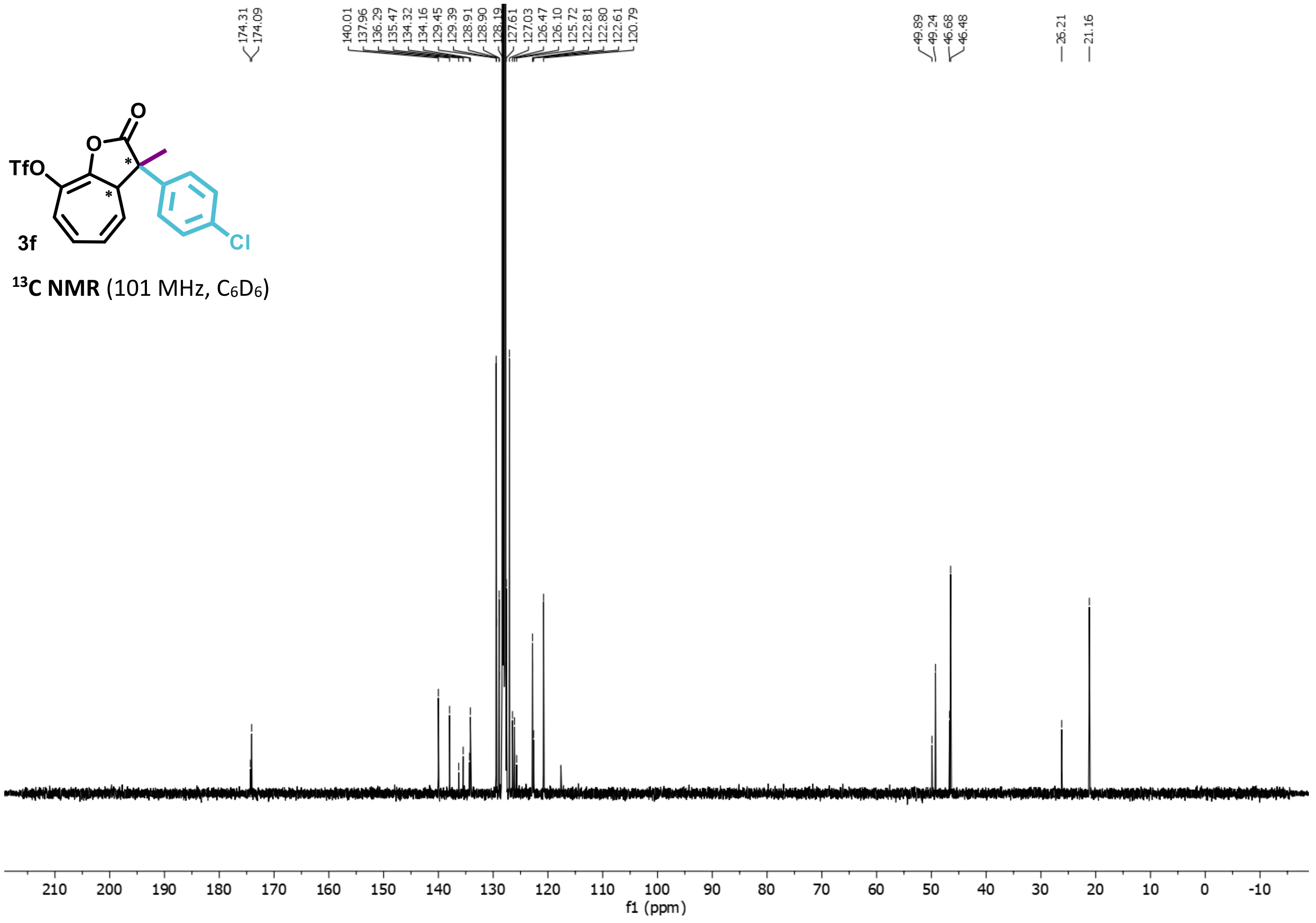
TfO
O
O
3f
Cl
¹³C NMR (101 MHz, C₆D₆)
174.31 174.09 140.01 137.96 136.29 135.47 134.32 134.16 129.45 129.39 128.91 128.90 128.19 127.61 127.03 126.47 126.10 125.72 122.81 122.80 122.61 120.79 49.89 49.24 46.68 46.48 26.21 21.16
f1 (ppm)

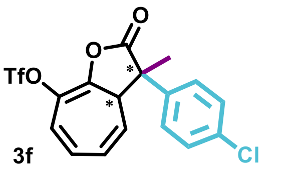

3f

^{19}F NMR (376 MHz, C_6D_6)

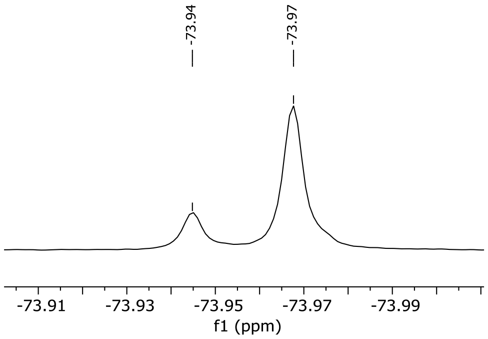

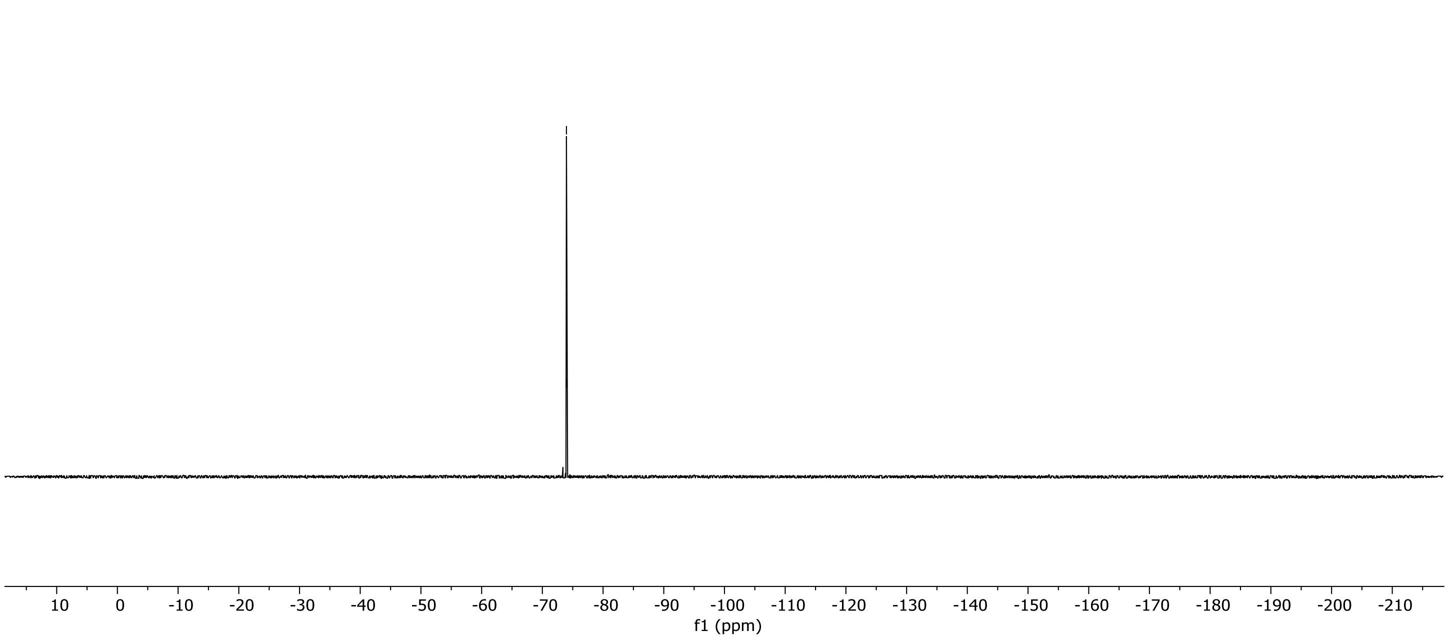

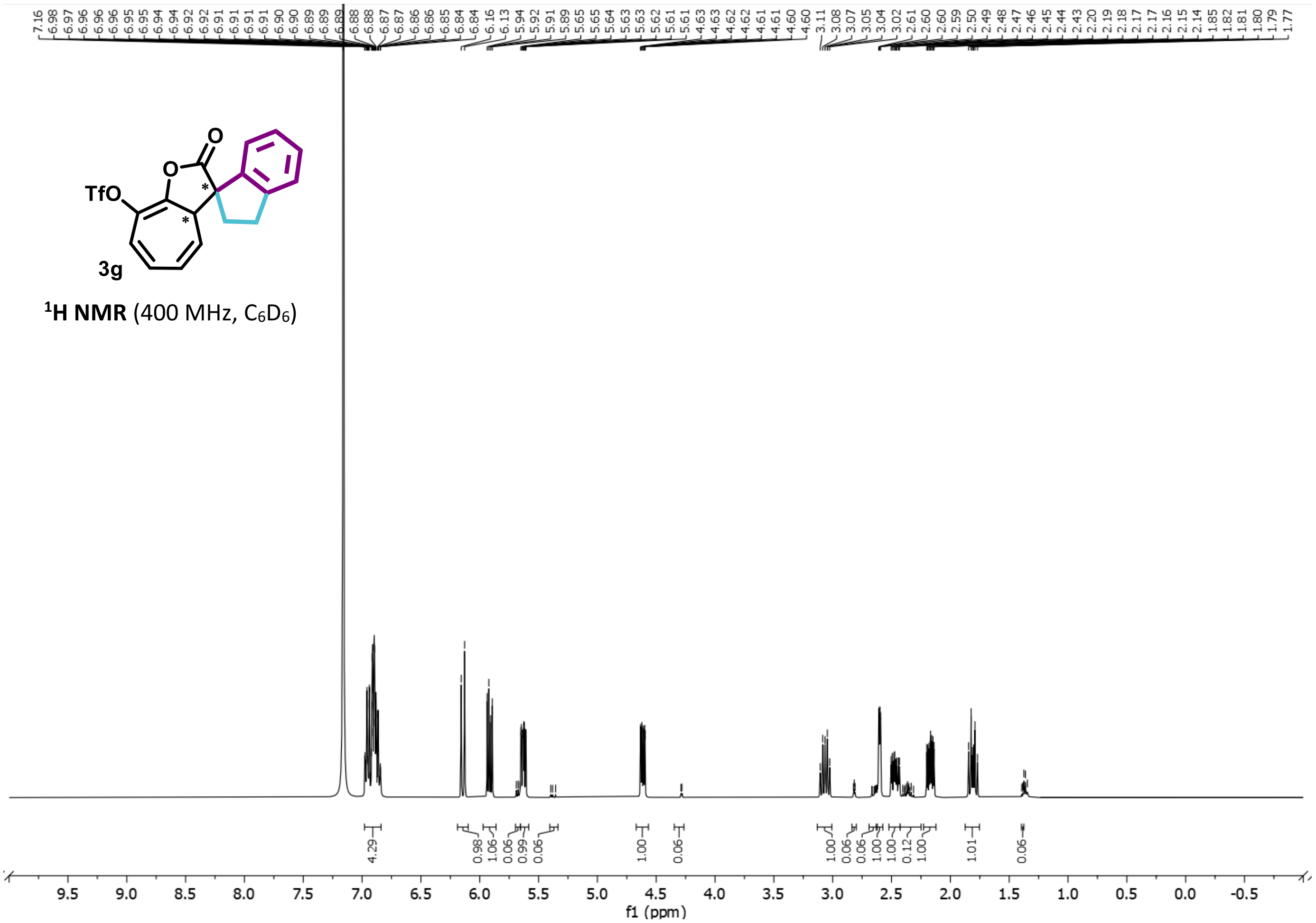
TfO
O
O
3g
¹H NMR (400 MHz, C₆D₆)
f1 (ppm)

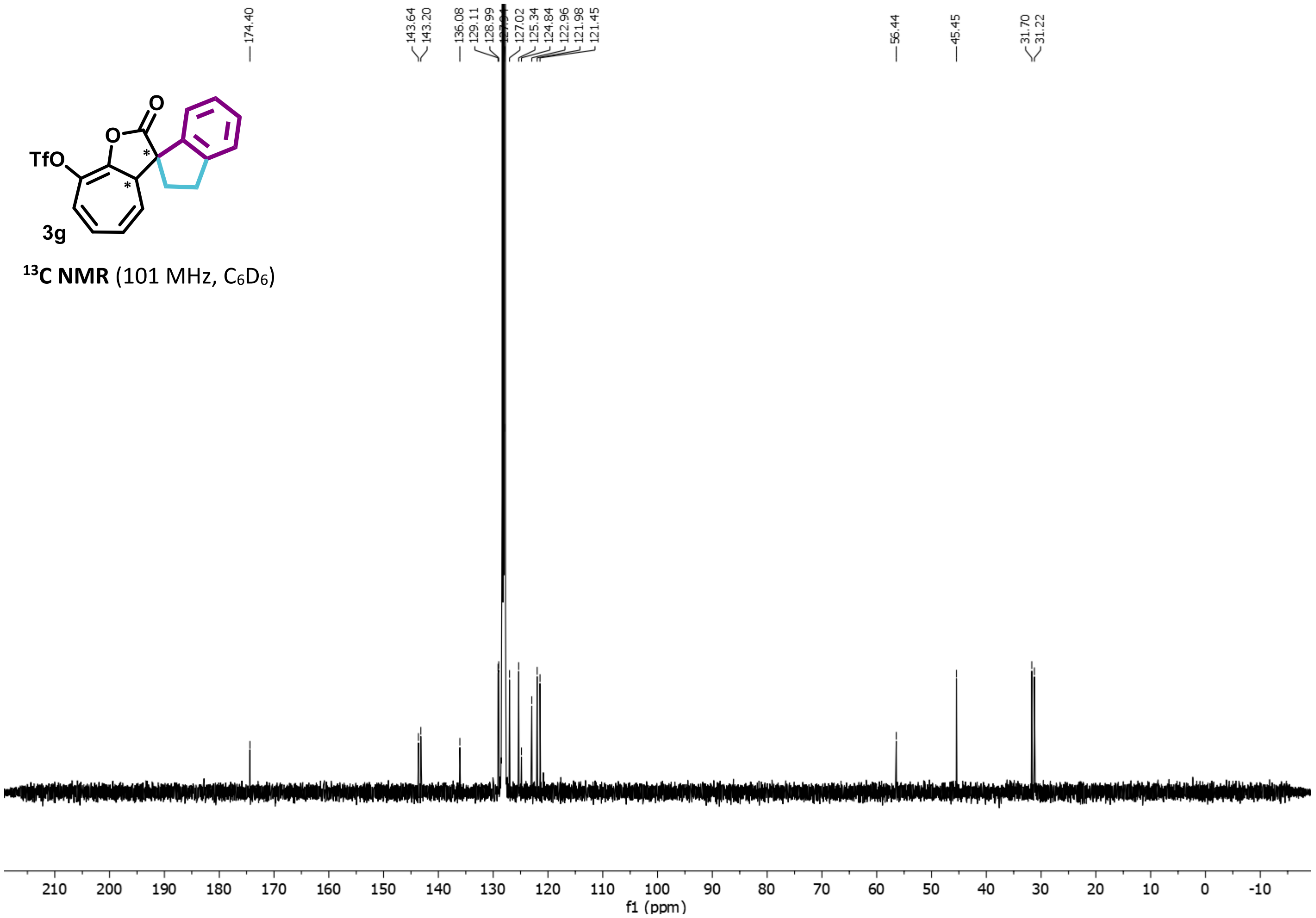
TfO
O
O
3g
¹³C NMR (101 MHz, C₆D₆)
f1 (ppm)

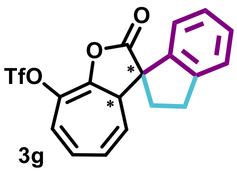

3g

^{19}F NMR (376 MHz, C_6D_6)

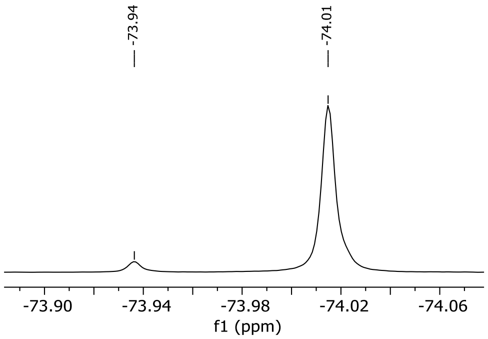

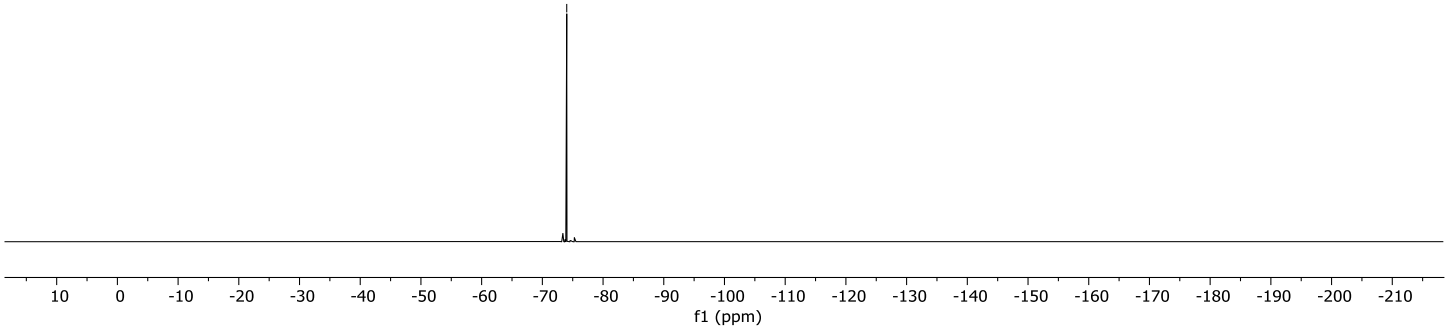

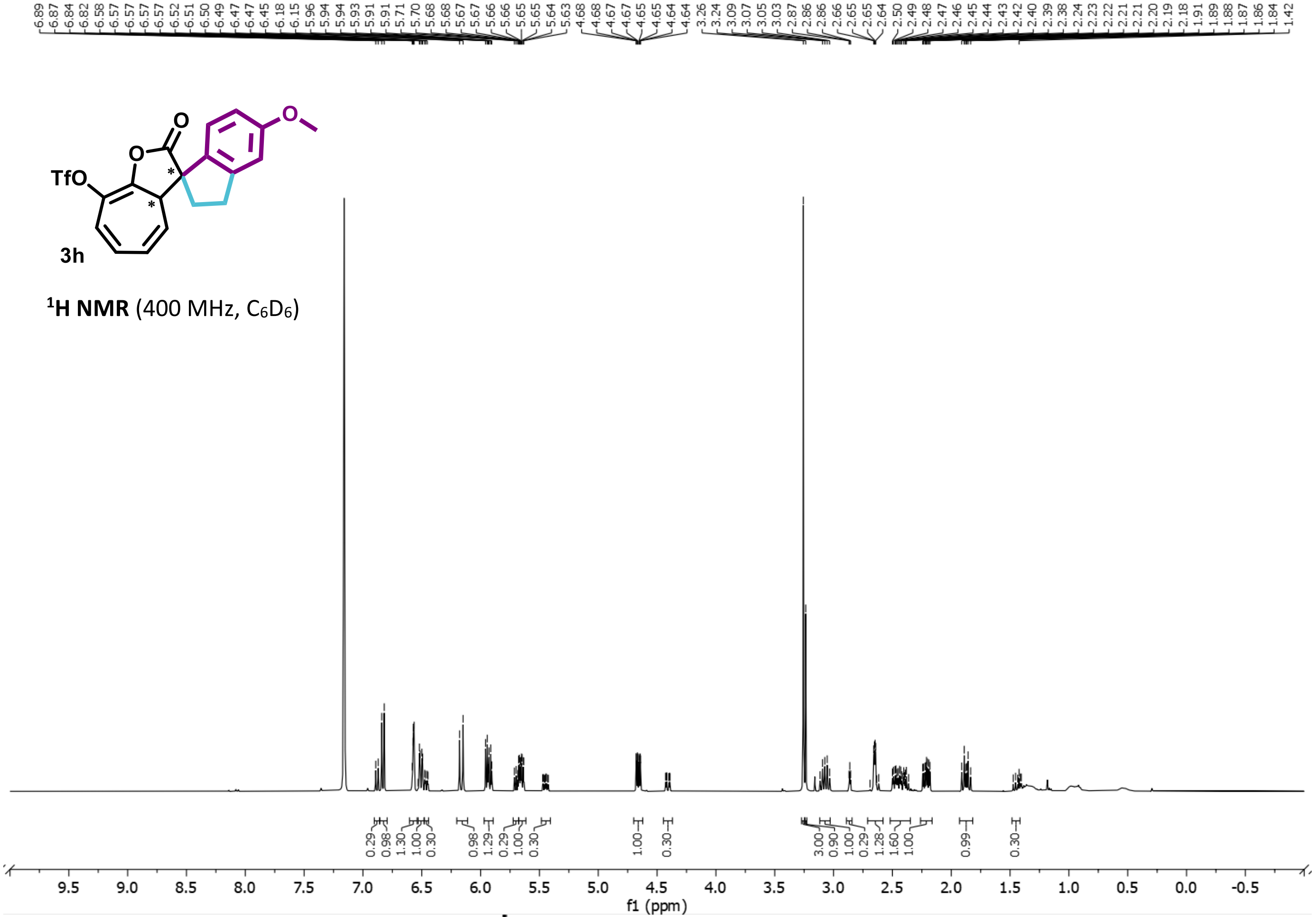
6.89 6.87 6.84 6.82 6.58 6.57 6.57 6.57 6.52 6.51 6.50 6.49 6.47 6.47 6.45 6.18 6.15 5.96 5.94 5.94 5.93 5.91 5.91 5.71 5.70 5.68 5.68 5.67 5.67 5.66 5.66 5.65 5.65 5.64 5.63 4.68 4.68 4.67 4.65 4.65 4.64 4.64 3.26 3.24 3.09 3.07 3.05 3.03 2.87 2.86 2.66 2.65 2.65 2.64 2.50 2.49 2.48 2.47 2.46 2.45 2.44 2.43 2.42 2.40 2.39 2.38 2.24 2.23 2.22 2.21 2.20 2.19 2.18 1.91 1.89 1.88 1.87 1.86 1.84 1.42
TfO
O
O
3h
^{1}H NMR (400 MHz, C_6D_6)
0.29 0.98 1.30 1.00 0.30 0.98 1.29 0.29 1.00 0.30 1.00 0.30 3.00 0.90 1.00 0.29 1.28 1.60 1.00 0.99 0.30
9.5 9.0 8.5 8.0 7.5 7.0 6.5 6.0 5.5 5.0 4.5 4.0 3.5 3.0 2.5 2.0 1.5 1.0 0.5 0.0 -0.5
f1 (ppm)

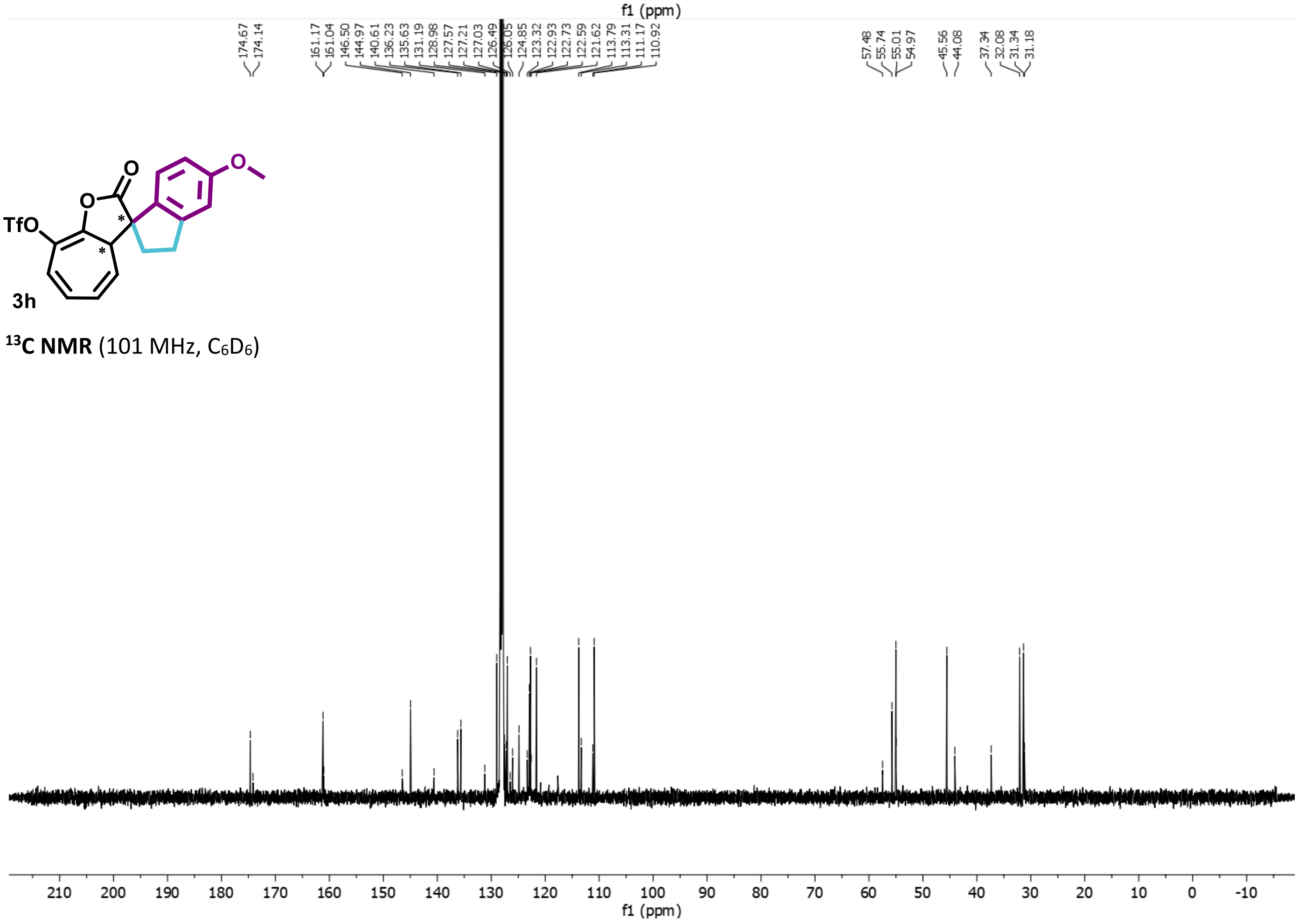
174.67 174.14 161.17 161.04 146.50 144.97 140.61 136.23 135.63 131.19 128.98 127.57 127.21 127.03 126.49 126.05 124.85 123.32 122.93 122.73 122.59 121.62 113.79 113.31 111.17 110.92 57.48 55.74 55.01 54.97 45.56 44.08 37.34 32.08 31.34 31.18
TfO
O
O
3h
^{13}C NMR (101 MHz, C_6D_6)
210 200 190 180 170 160 150 140 130 120 110 100 90 80 70 60 50 40 30 20 10 0 -10
f1 (ppm)

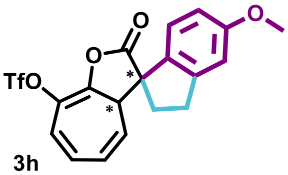

^{19}F NMR (376 MHz, C_6D_6)

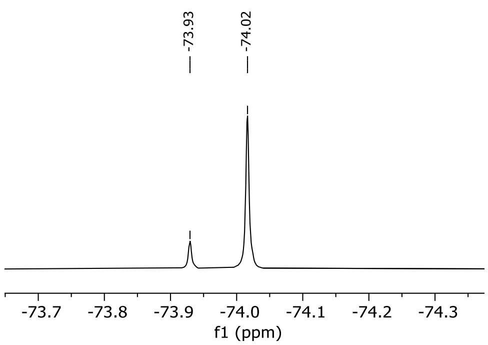

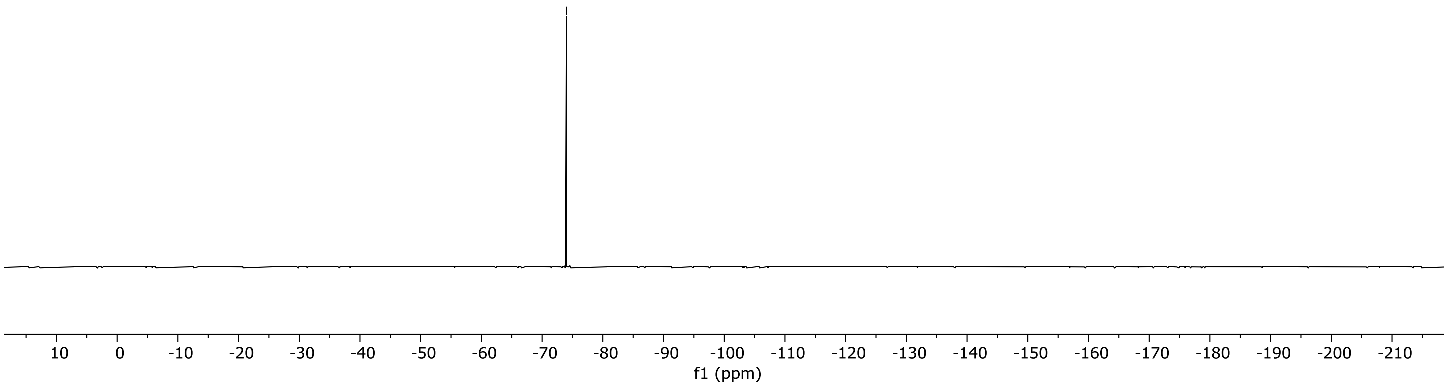

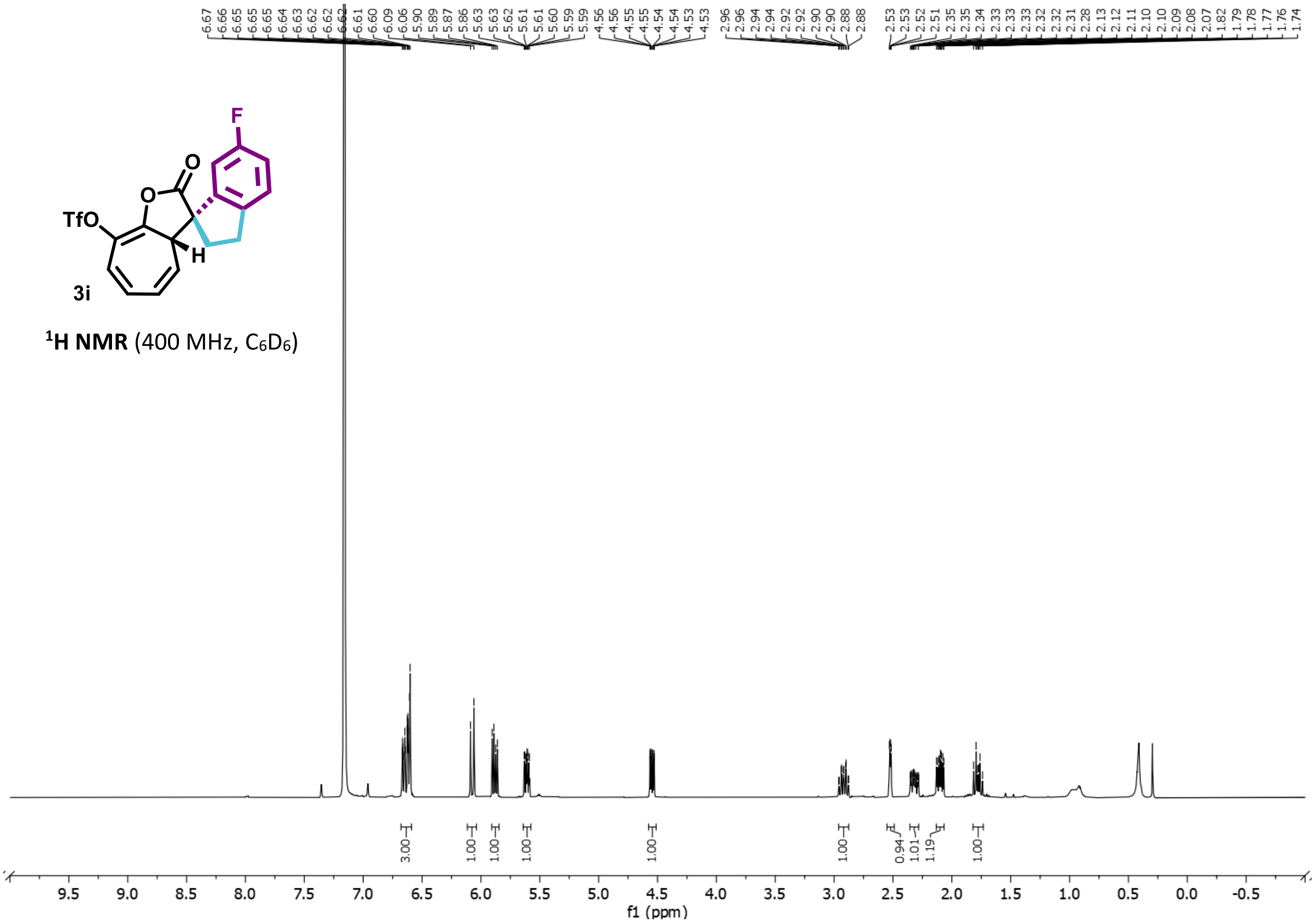
6.67 6.66 6.65 6.65 6.64 6.63 6.62 6.62 6.62 6.61 6.60 6.09 6.06 5.90 5.89 5.87 5.86 5.63 5.63 5.62 5.61 5.61 5.60 5.59 5.59 4.56 4.56 4.55 4.55 4.54 4.54 4.53 4.53 2.96 2.96 2.94 2.94 2.92 2.92 2.90 2.90 2.88 2.88 2.53 2.53 2.52 2.51 2.35 2.35 2.34 2.33 2.33 2.33 2.32 2.32 2.31 2.28 2.13 2.12 2.11 2.10 2.10 2.09 2.08 2.07 1.82 1.79 1.78 1.77 1.76 1.74
F
O
O
TfO
H
3i
1H NMR (400 MHz, C6D6)
3.00 1.00 1.00 1.00 1.00 1.00 0.94 1.01 1.19 1.00
f1 (ppm)

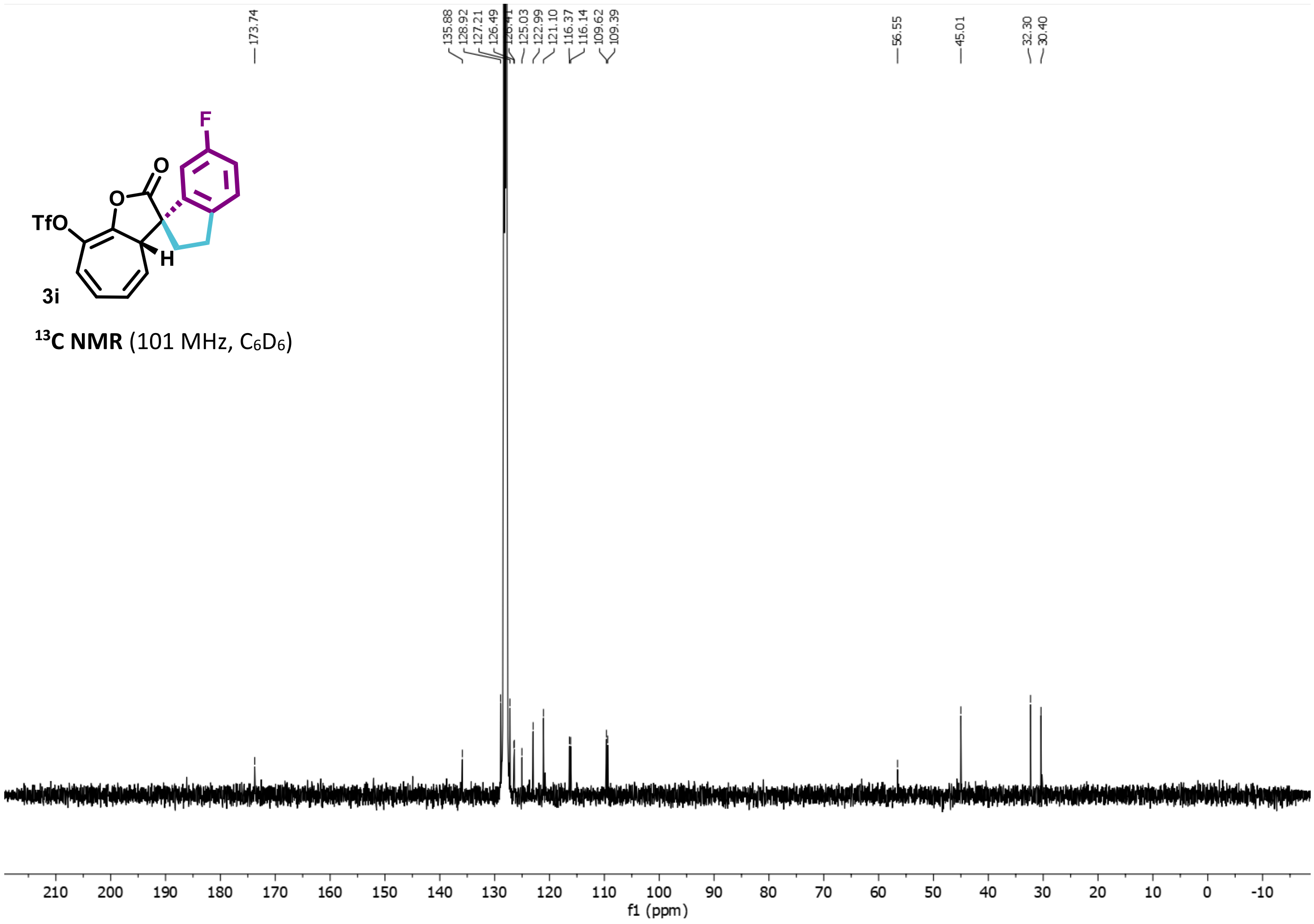
173.74
135.88 128.92 127.21 126.49 126.41 125.03 122.99 121.10 116.37 116.14 109.62 109.39
56.55
45.01
32.30 30.40
F
O
O
TfO
H
3i
13C NMR (101 MHz, C6D6)
f1 (ppm)

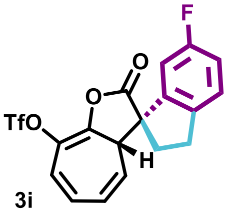

3i

^{19}F NMR (376 MHz, C_6D_6)

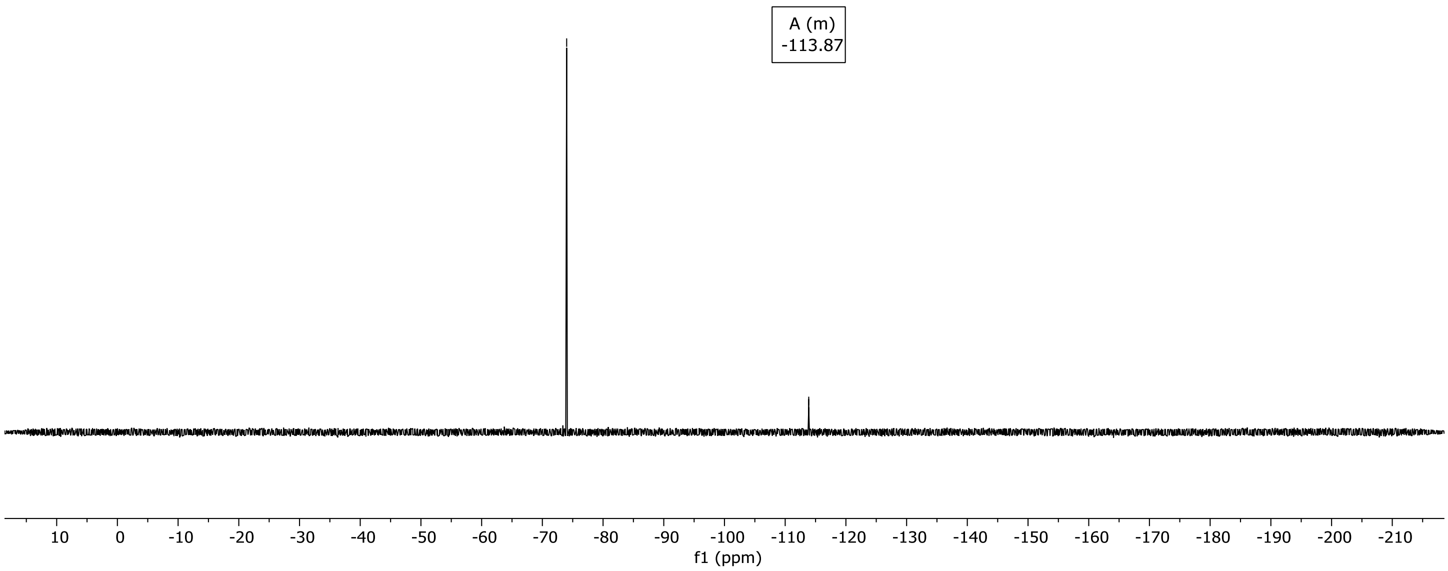

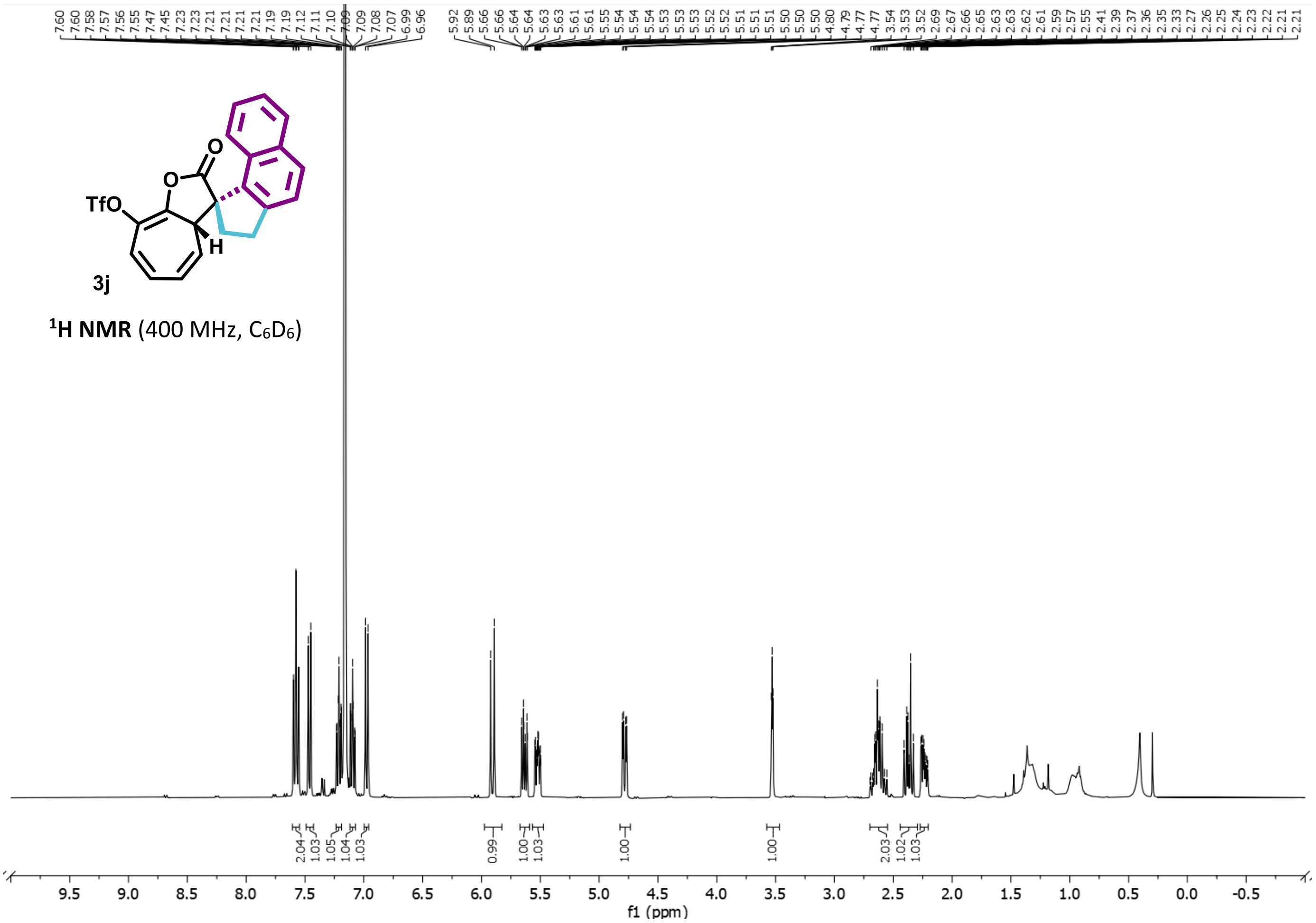
7.60 7.60 7.58 7.57 7.56 7.55 7.47 7.45 7.23 7.23 7.21 7.21 7.21 7.21 7.19 7.19 7.12 7.11 7.10 7.09 7.09 7.08 7.07 6.99 6.96
5.92 5.89 5.66 5.66 5.64 5.64 5.63 5.63 5.61 5.61 5.55 5.54 5.54 5.54 5.53 5.53 5.53 5.52 5.52 5.51 5.51 5.50 5.50 5.50 4.80 4.79 4.77 3.54 3.53 3.52 2.69 2.67 2.66 2.65 2.63 2.63 2.62 2.61 2.59 2.57 2.55 2.41 2.39 2.37 2.36 2.35 2.33 2.27 2.26 2.25 2.24 2.23 2.22 2.21 2.21
TfO
O
O
H
3j
1H NMR (400 MHz, C6D6)
2.04 1.03 1.05 1.04 1.03 0.99 1.00 1.03 1.00 1.00 2.03 1.02 1.03
9.5 9.0 8.5 8.0 7.5 7.0 6.5 6.0 5.5 5.0 4.5 4.0 3.5 3.0 2.5 2.0 1.5 1.0 0.5 0.0 -0.5
f1 (ppm)

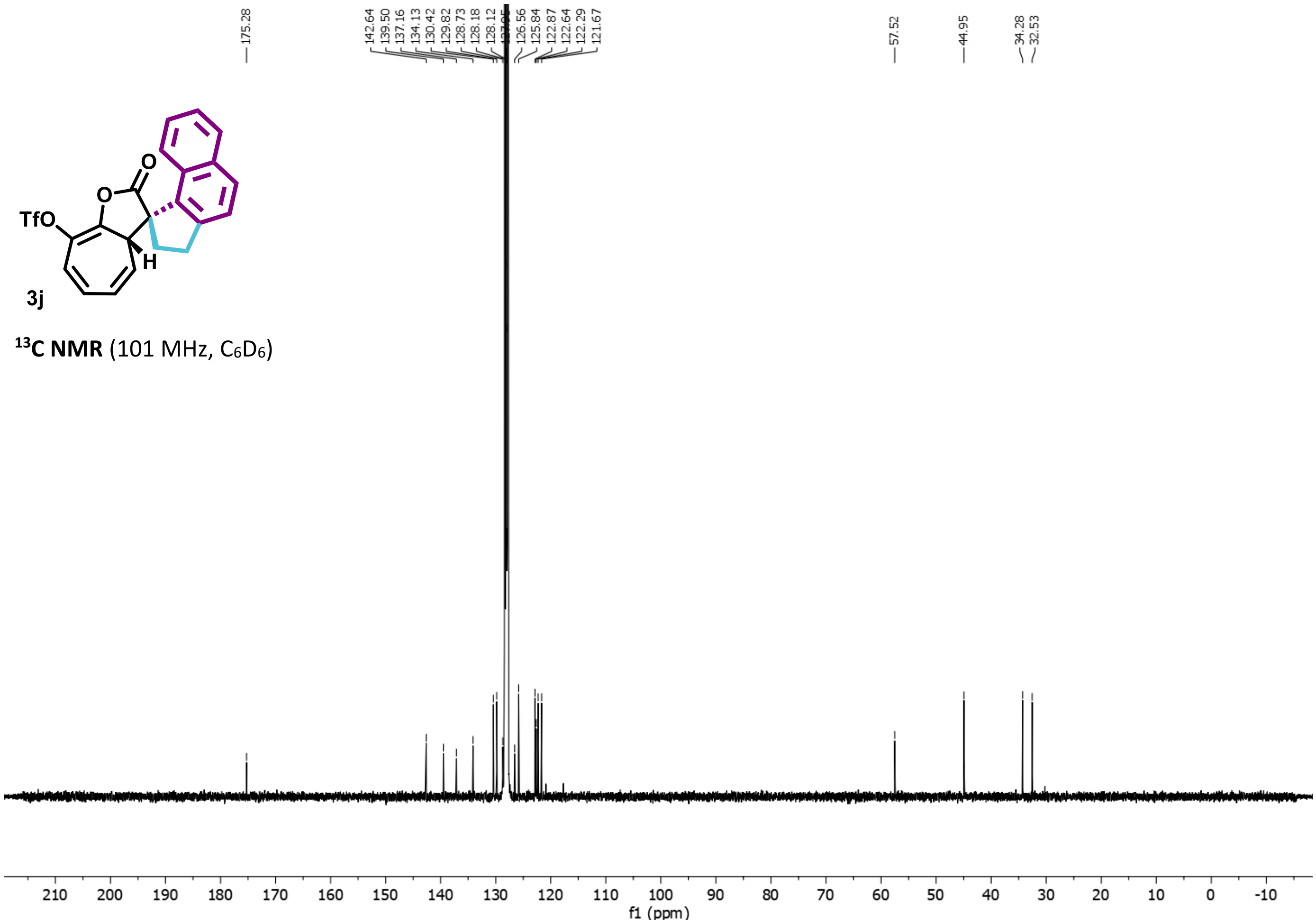
175.28
142.64 139.50 137.16 134.13 130.42 129.82 128.73 128.18 128.12 126.56 125.84 122.87 122.64 122.29 121.67
57.52
44.95
34.28 32.53
TfO
O
O
H
3j
13C NMR (101 MHz, C6D6)
210 200 190 180 170 160 150 140 130 120 110 100 90 80 70 60 50 40 30 20 10 0 -10
f1 (ppm)

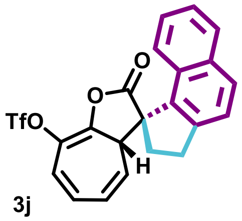

3j

^{19}F NMR (376 MHz, C_6D_6)

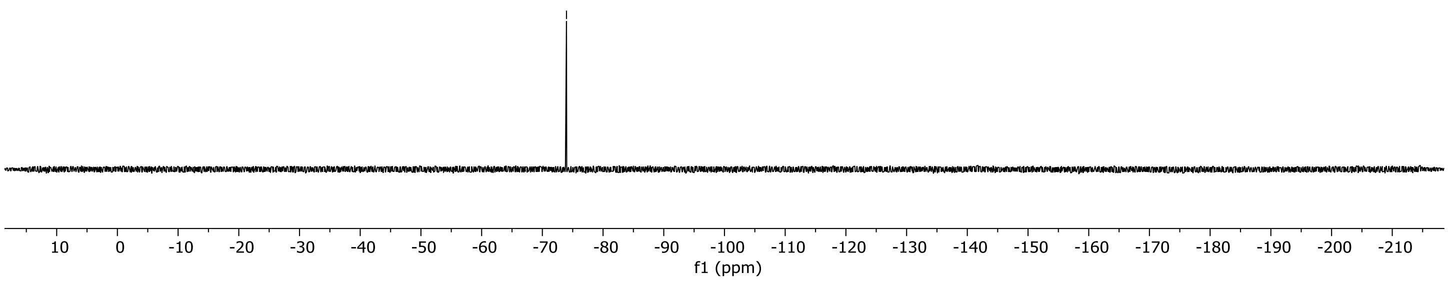

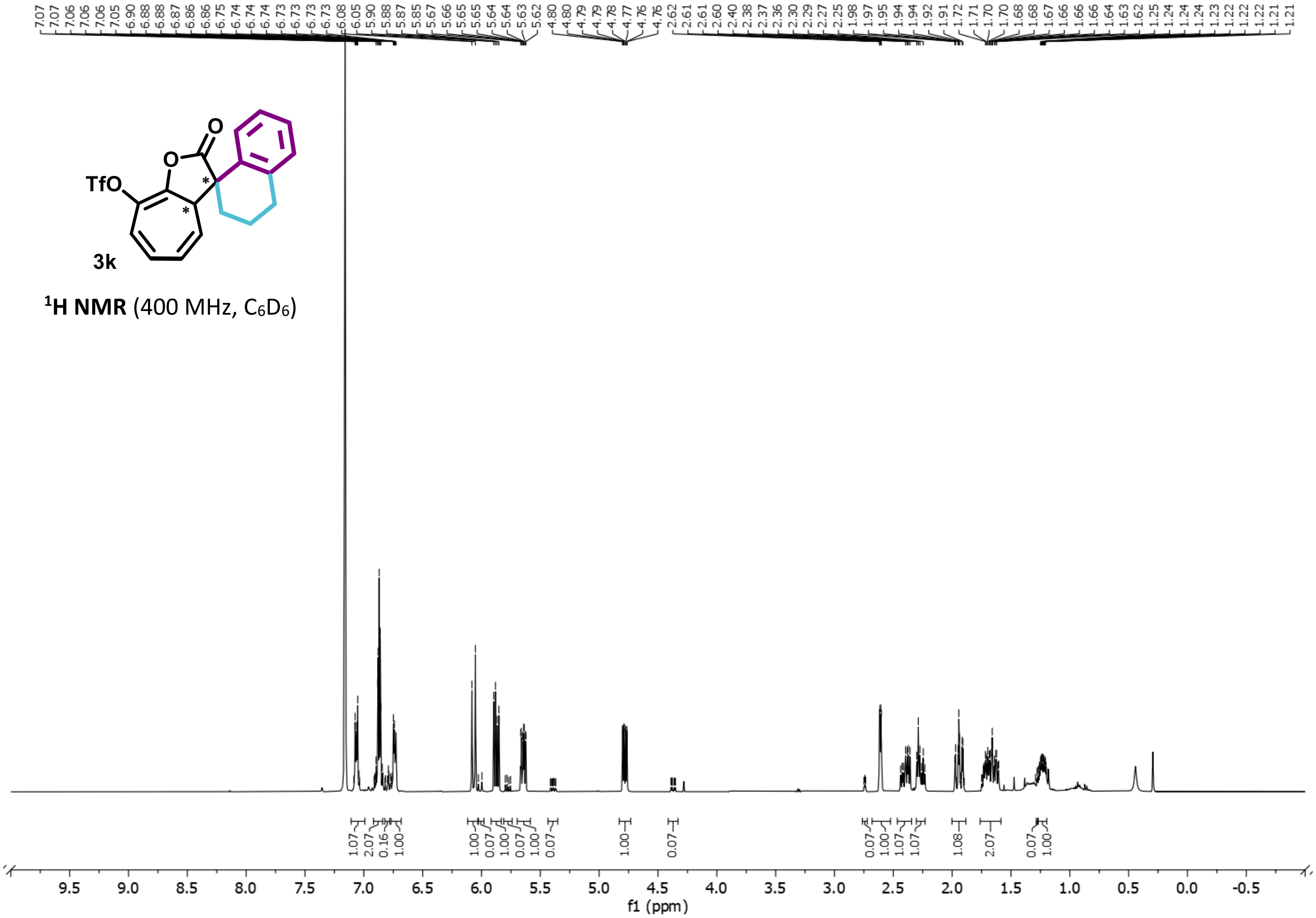
7.07
7.07
7.06
7.06
7.06
7.05
6.90
6.88
6.88
6.87
6.86
6.86
6.75
6.74
6.74
6.74
6.73
6.73
6.73
6.73
6.08
6.05
5.90
5.88
5.87
5.85
5.67
5.66
5.65
5.65
5.64
5.64
5.64
5.63
5.62
4.80
4.80
4.79
4.79
4.78
4.77
4.76
4.76
2.62
2.61
2.61
2.60
2.40
2.38
2.37
2.36
2.30
2.29
2.27
2.25
1.98
1.97
1.95
1.94
1.94
1.92
1.91
1.72
1.71
1.70
1.70
1.68
1.68
1.67
1.66
1.66
1.64
1.63
1.25
1.24
1.24
1.24
1.23
1.22
1.22
1.22
1.21
1.21
O
O
TfO
*
*
3k
1H NMR (400 MHz, C6D6)
1.07
2.07
0.16
1.00
1.00
0.07
1.00
0.07
1.00
0.07
1.00
0.07
1.00
0.07
1.00
1.07
1.07
1.08
2.07
0.07
1.00
f1 (ppm)

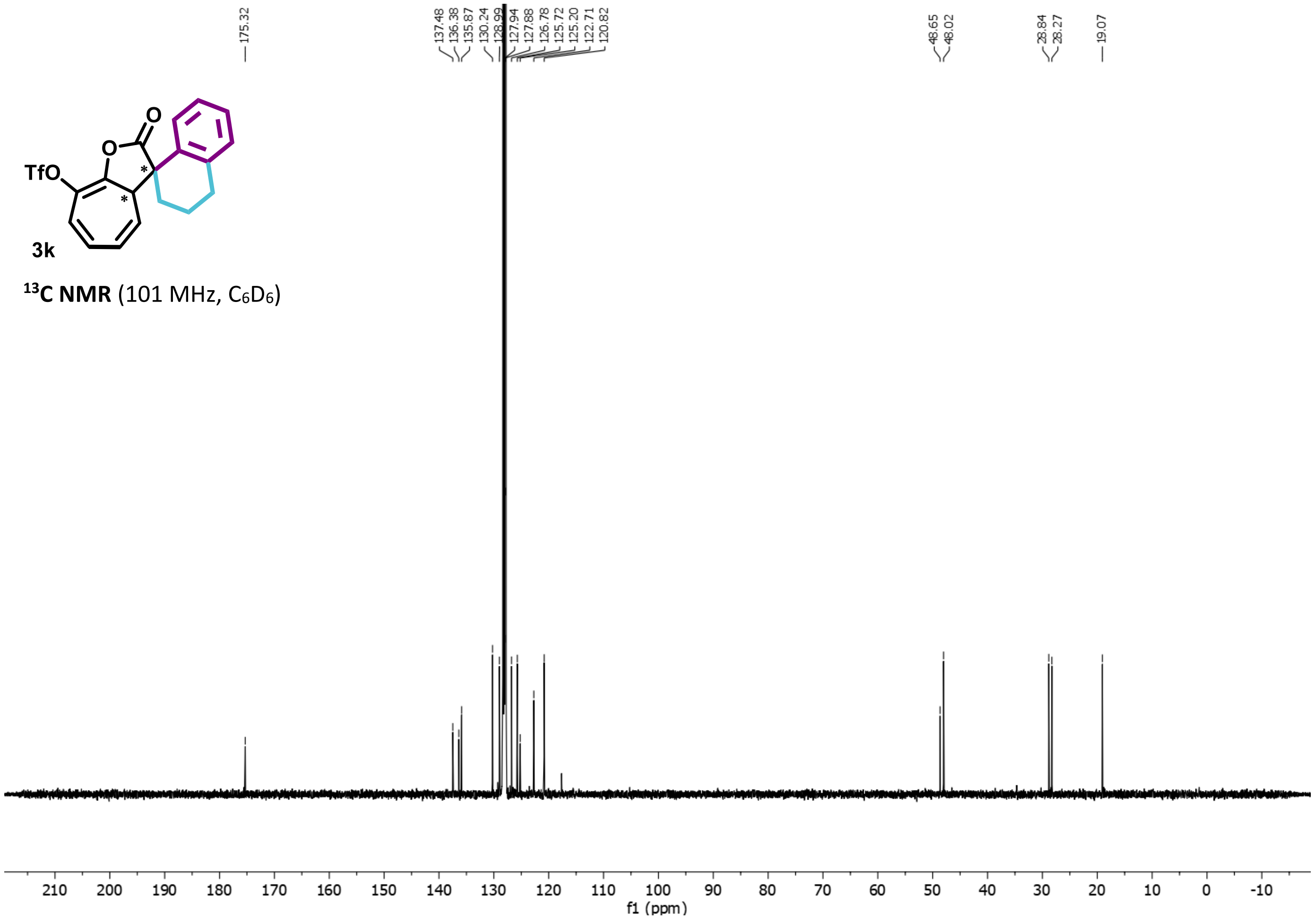
175.32
137.48
136.38
135.87
130.24
128.99
127.94
127.88
126.78
125.72
125.20
122.71
120.82
48.65
48.02
28.84
28.27
19.07
O
O
TfO
*
*
3k
13C NMR (101 MHz, C6D6)
f1 (ppm)

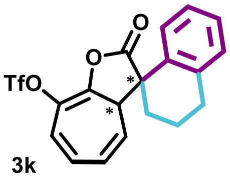

3k

^{19}F NMR (376 MHz, C_6D_6)

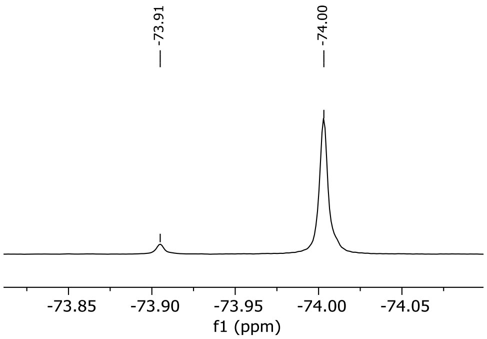

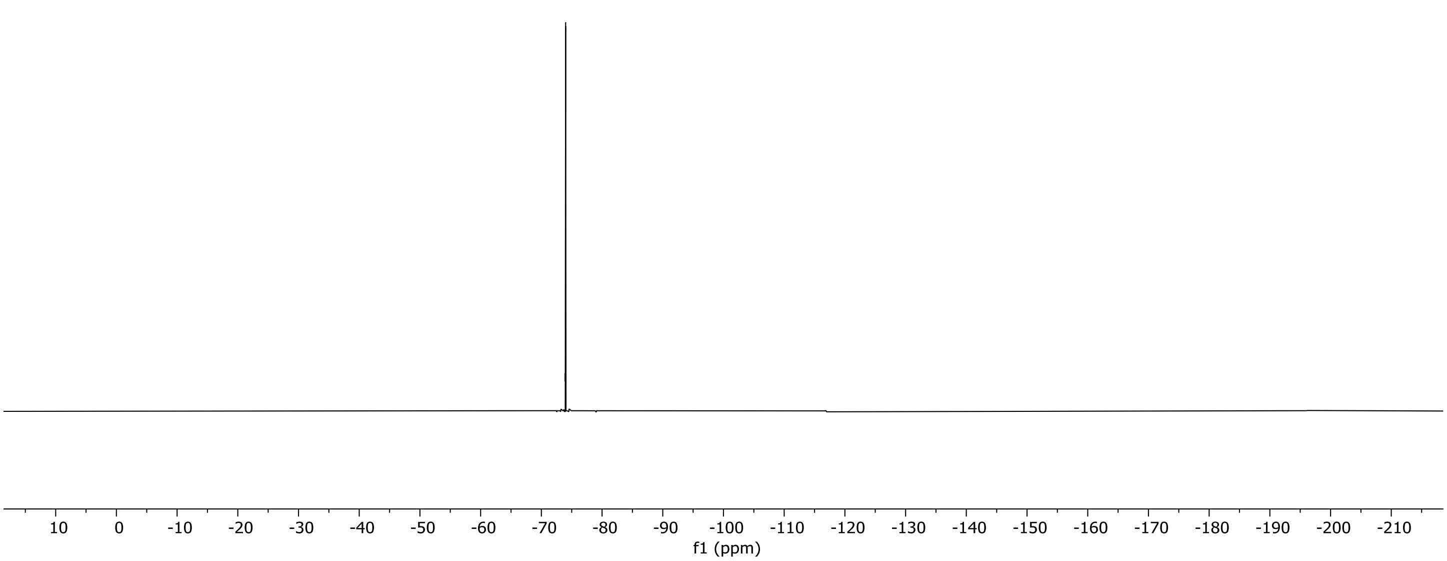

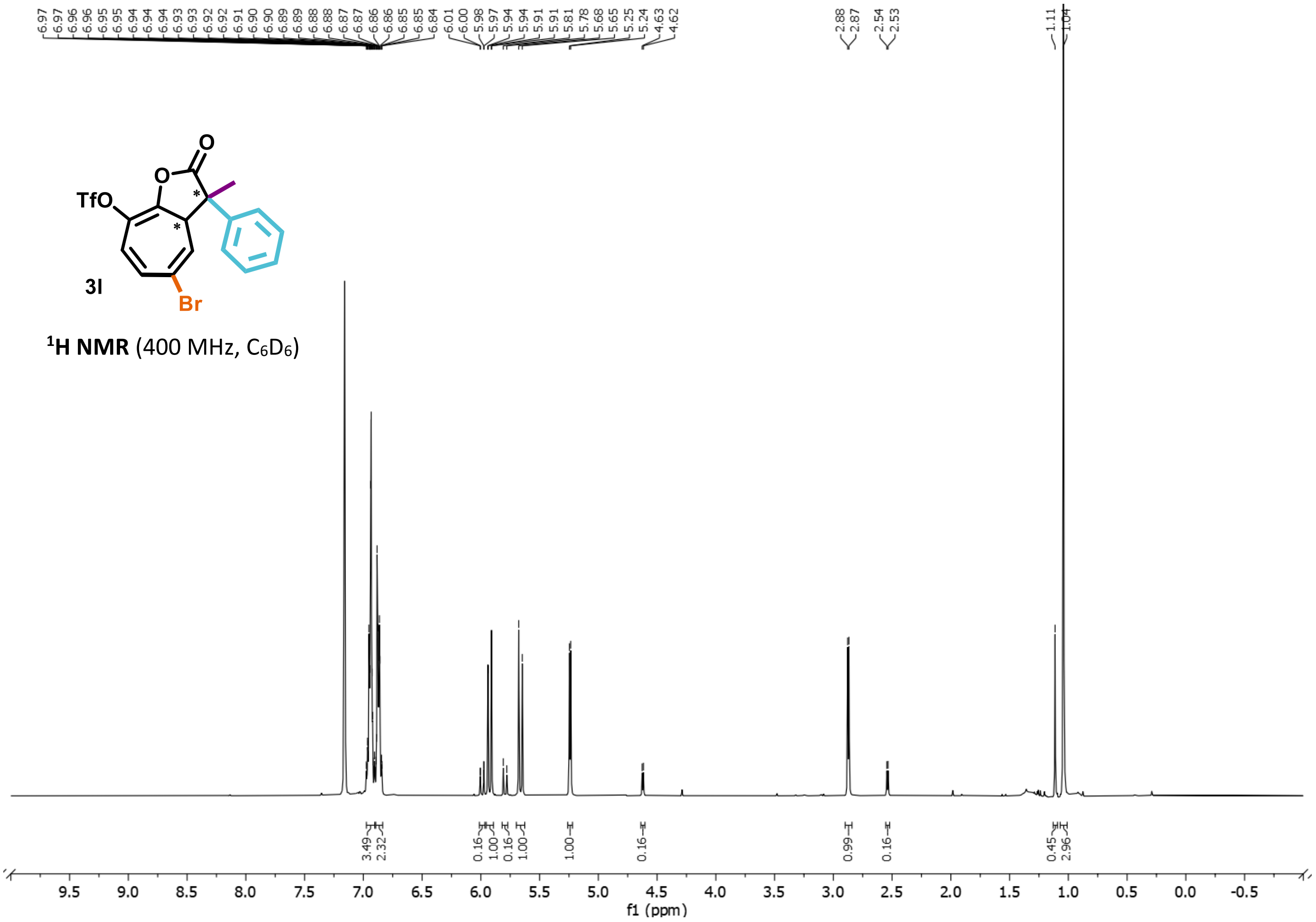

^{1}H **NMR** (400 MHz, C_6D_6)

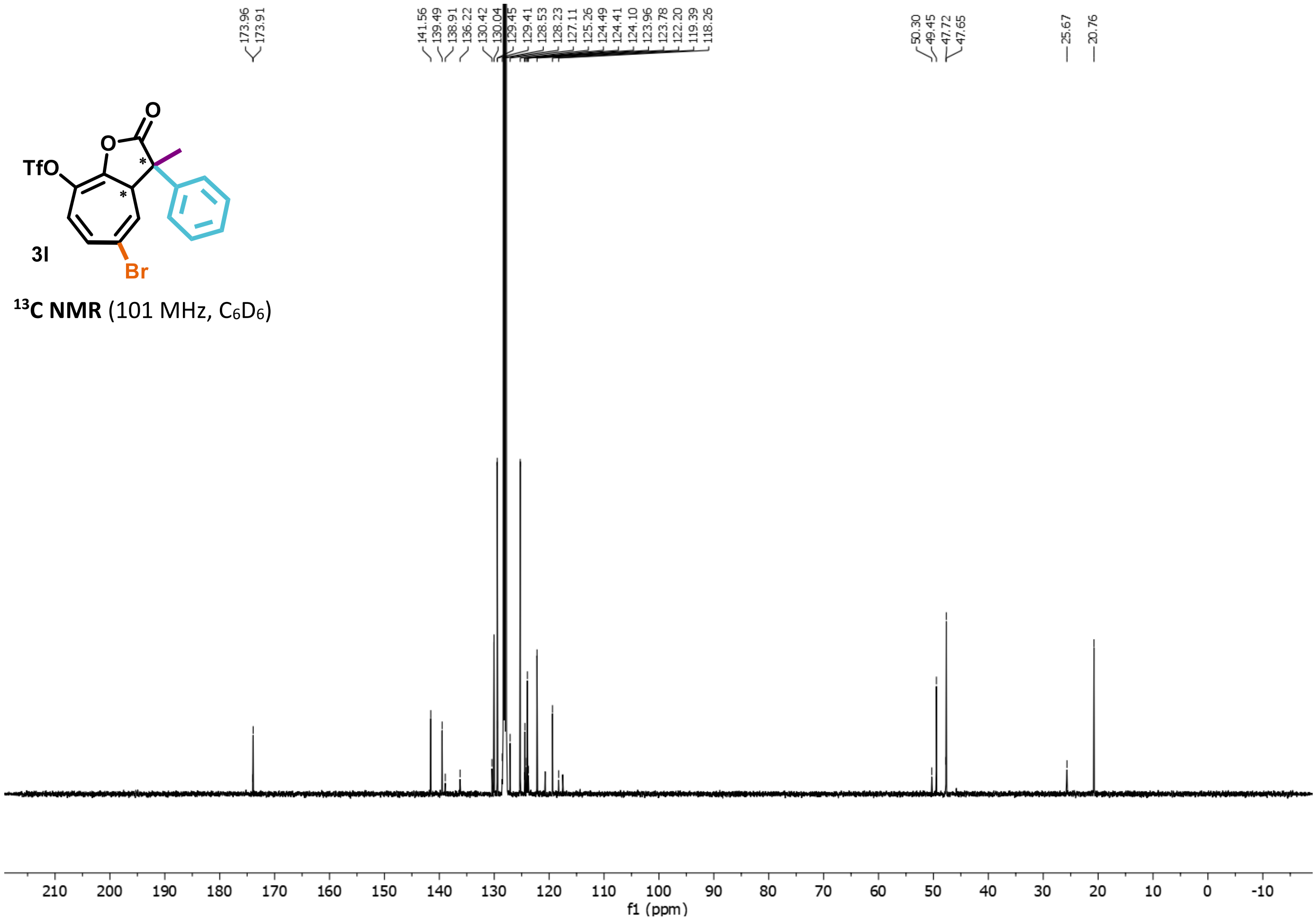

^{13}C **NMR** (101 MHz, C_6D_6)

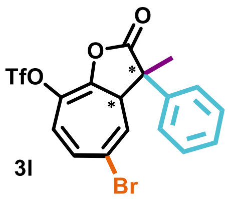

3l

^{19}**F NMR** (376 MHz, C_6D_6)

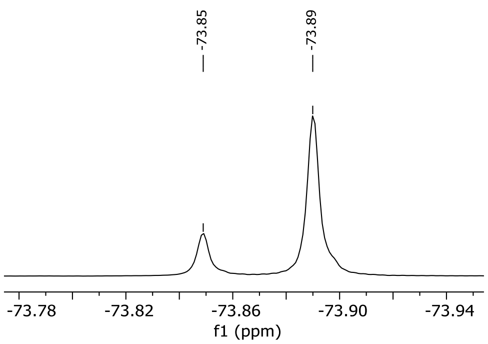

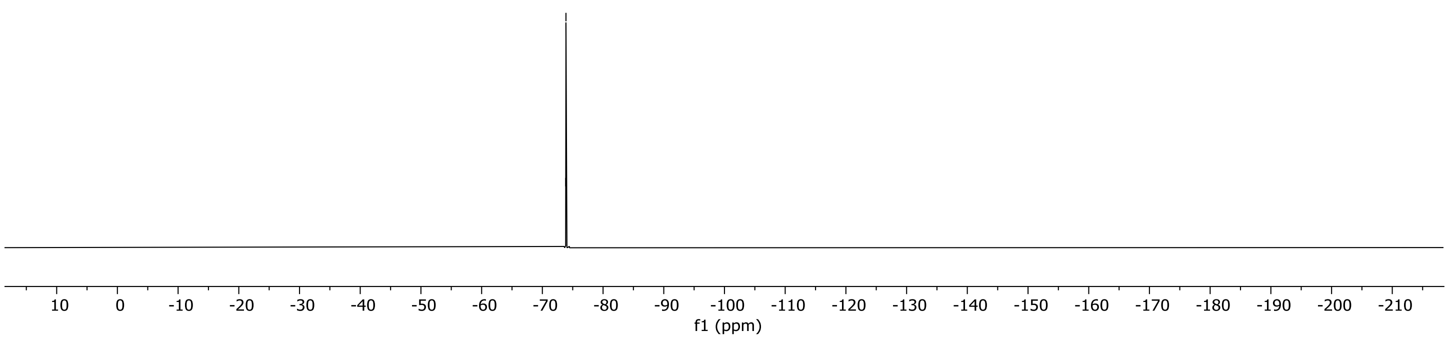

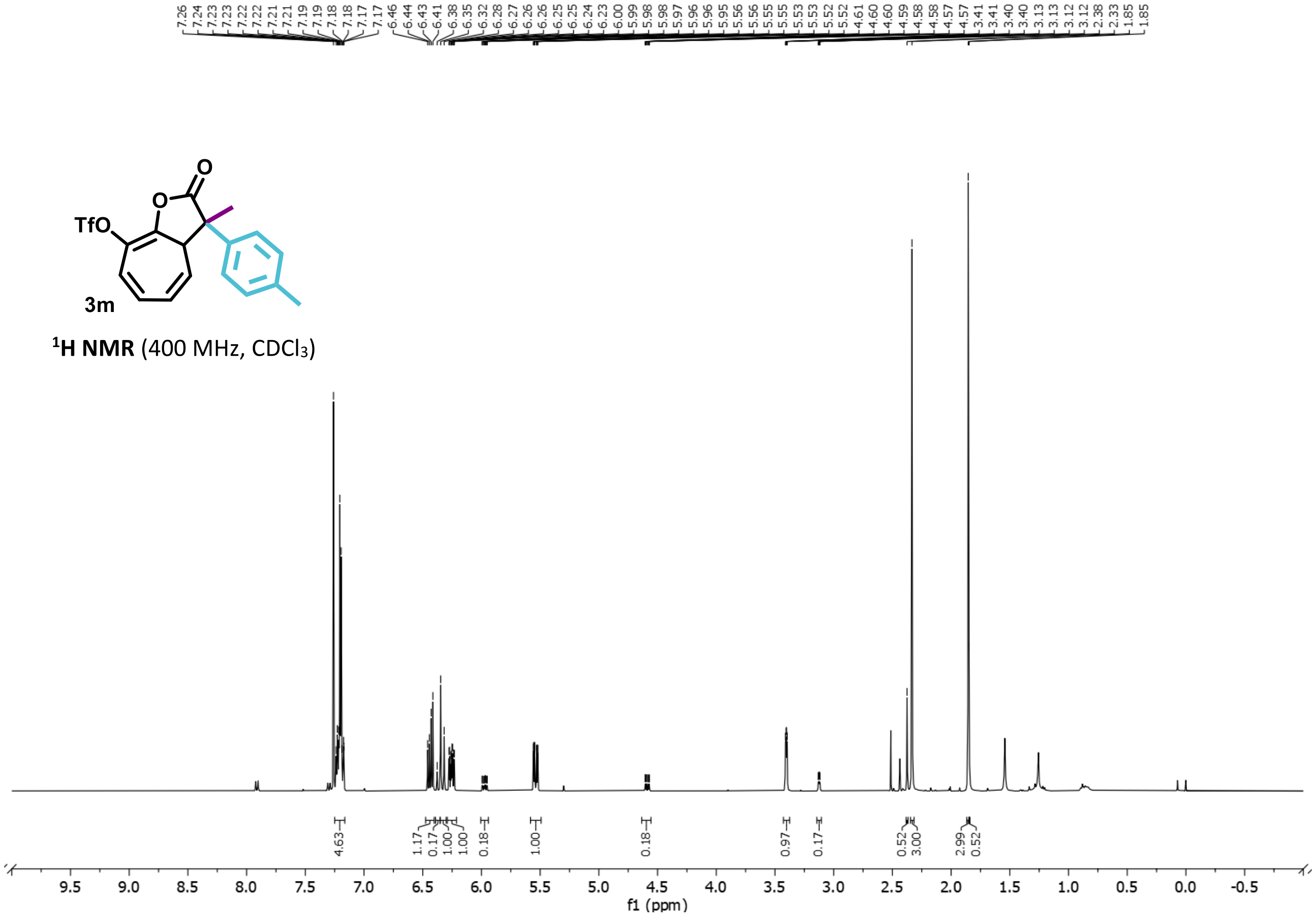
7.26 7.24 7.23 7.23 7.22 7.22 7.21 7.21 7.19 7.19 7.18 7.18 7.17 7.17 6.46 6.44 6.43 6.41 6.38 6.35 6.32 6.28 6.27 6.26 6.26 6.25 6.25 6.24 6.23 6.00 5.99 5.98 5.98 5.97 5.96 5.96 5.95 5.56 5.56 5.55 5.55 5.53 5.53 5.52 5.52 4.61 4.60 4.60 4.59 4.58 4.58 4.57 4.57 3.41 3.41 3.40 3.40 3.13 3.13 3.12 3.12 2.38 2.33 1.85 1.85
TfO
O
O
3m
^{1}H NMR (400 MHz, CDCl$_3$)
4.63 1.17 0.17 1.00 1.00 0.18 1.00 0.18 0.97 0.17 0.52 3.00 2.99 0.52
9.5 9.0 8.5 8.0 7.5 7.0 6.5 6.0 5.5 5.0 4.5 4.0 3.5 3.0 2.5 2.0 1.5 1.0 0.5 0.0 -0.5
f1 (ppm)

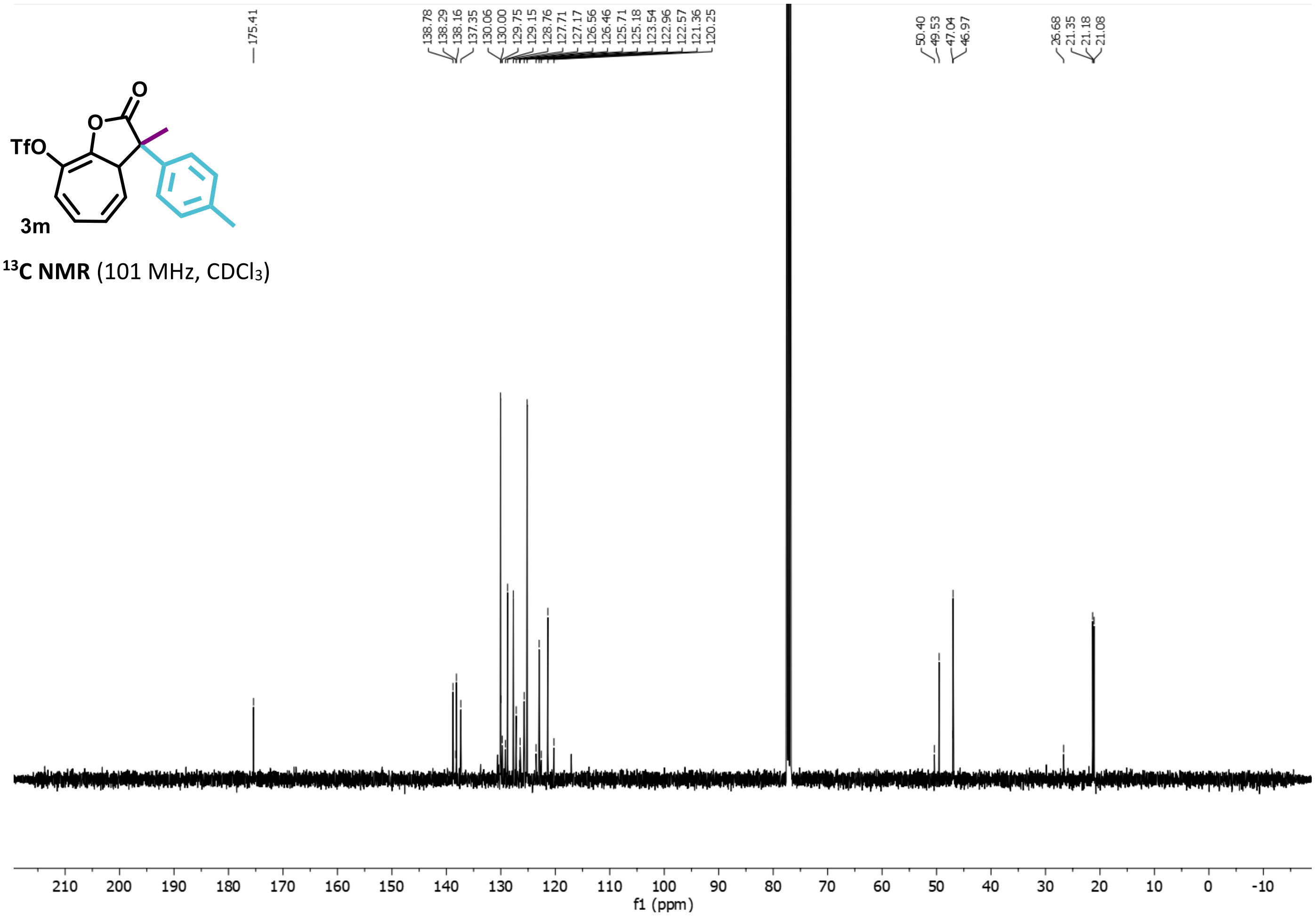
175.41
138.78 138.29 138.16 137.35 130.06 130.00 129.75 129.15 128.76 127.71 127.17 126.56 126.46 125.71 125.18 123.54 122.96 122.57 121.36 120.25
50.40 49.53 47.04 46.97
26.68 21.35 21.18 21.08
TfO
O
O
3m
^{13}C NMR (101 MHz, CDCl$_3$)
210 200 190 180 170 160 150 140 130 120 110 100 90 80 70 60 50 40 30 20 10 0 -10
f1 (ppm)

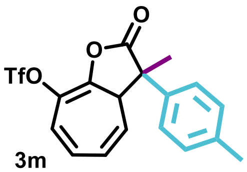

3m

^{19}F NMR (376 MHz, $CDCl_3$)

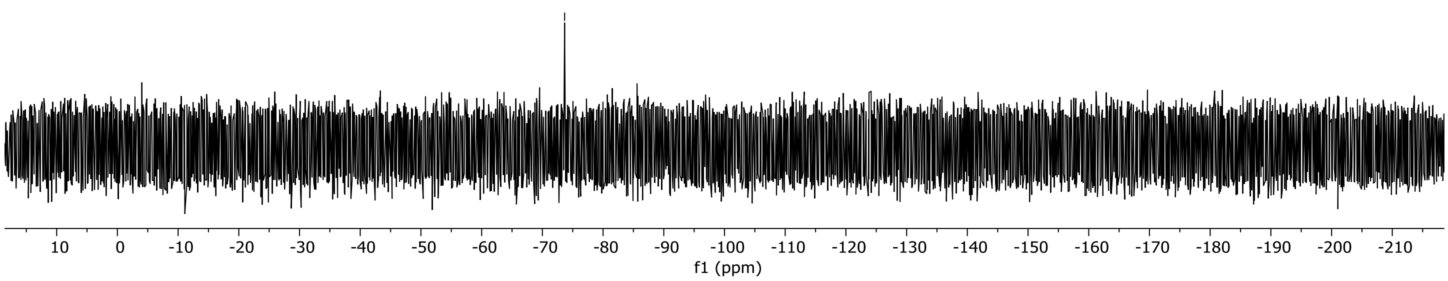

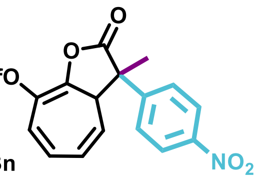

3n

^{1}**H NMR** (400 MHz, C_6D_6)

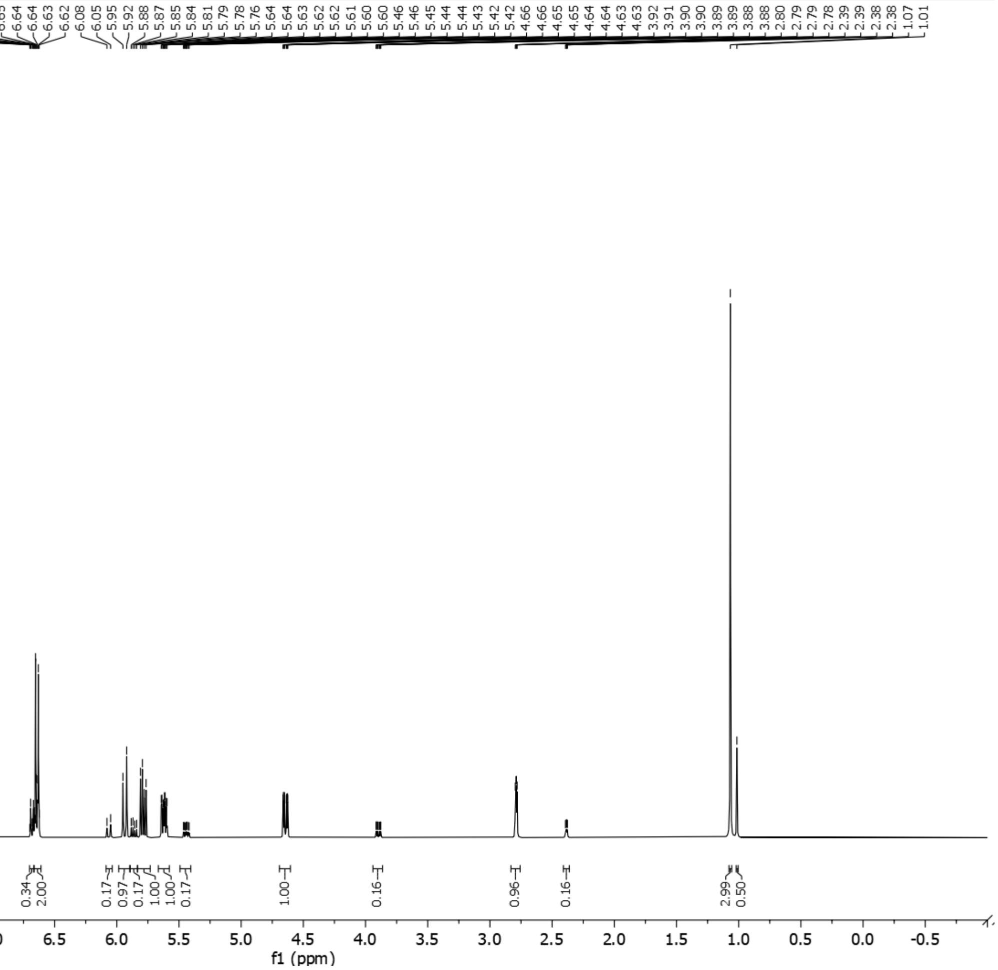

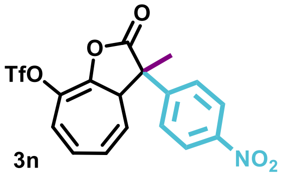

3n

^{13}**C NMR** (101 MHz, C_6D_6)

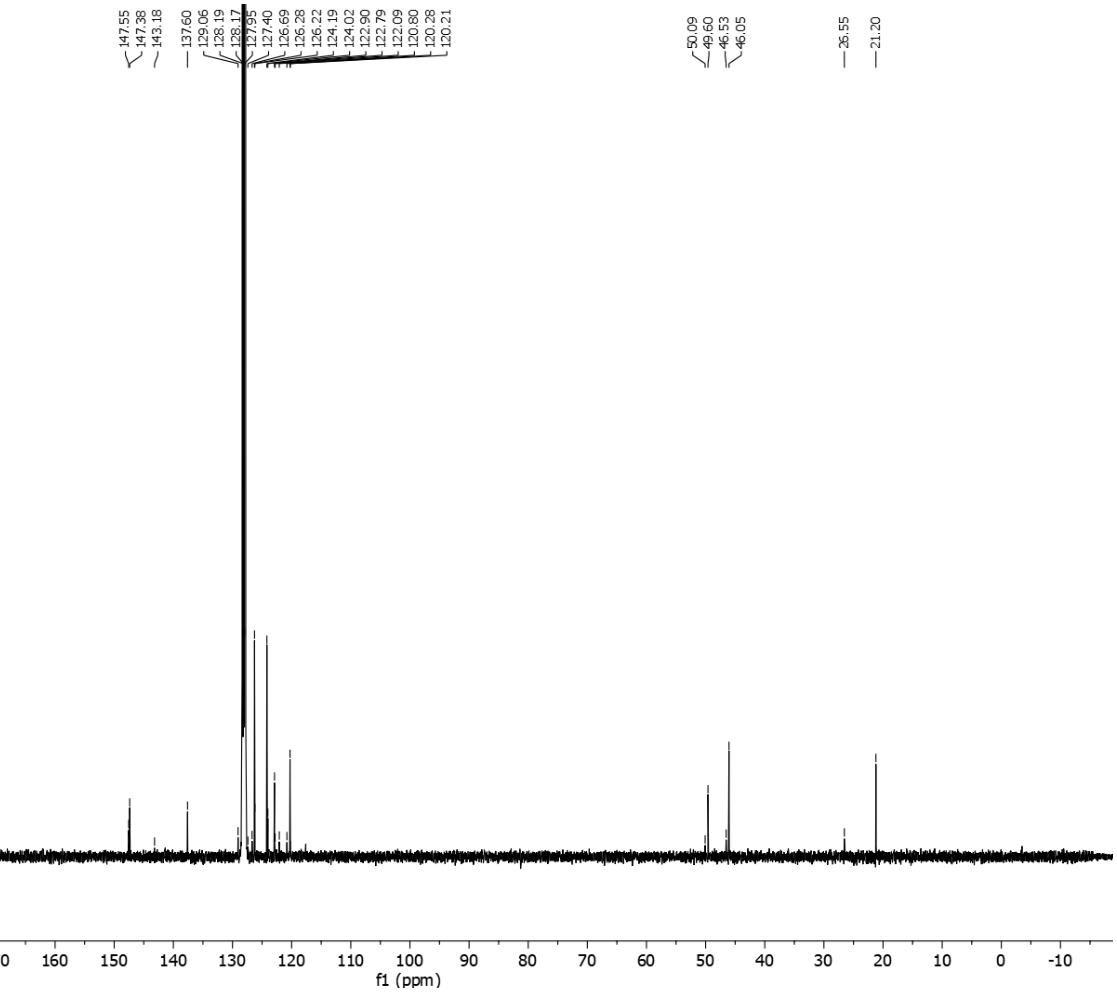

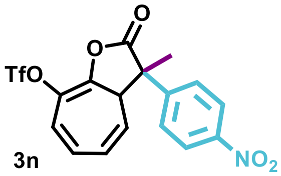

3n

^{19}F NMR (376 MHz, C_6D_6)

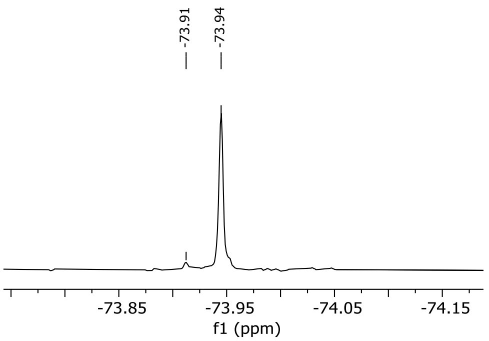

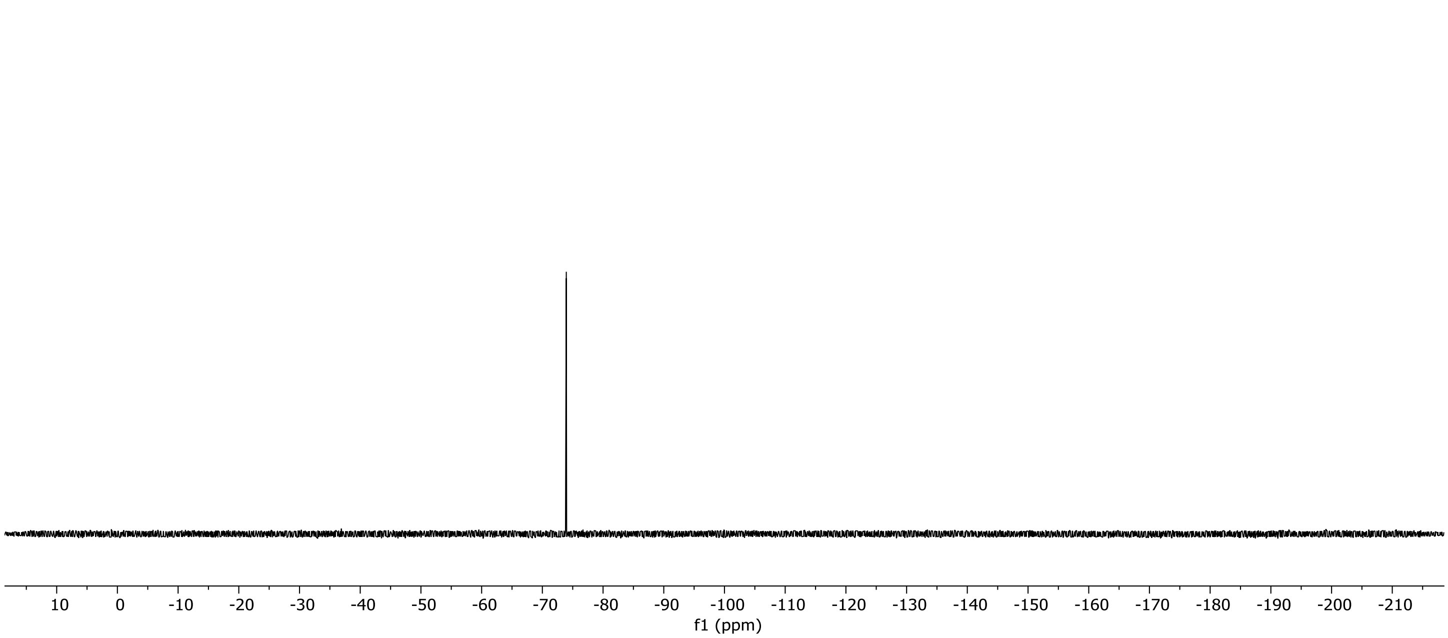

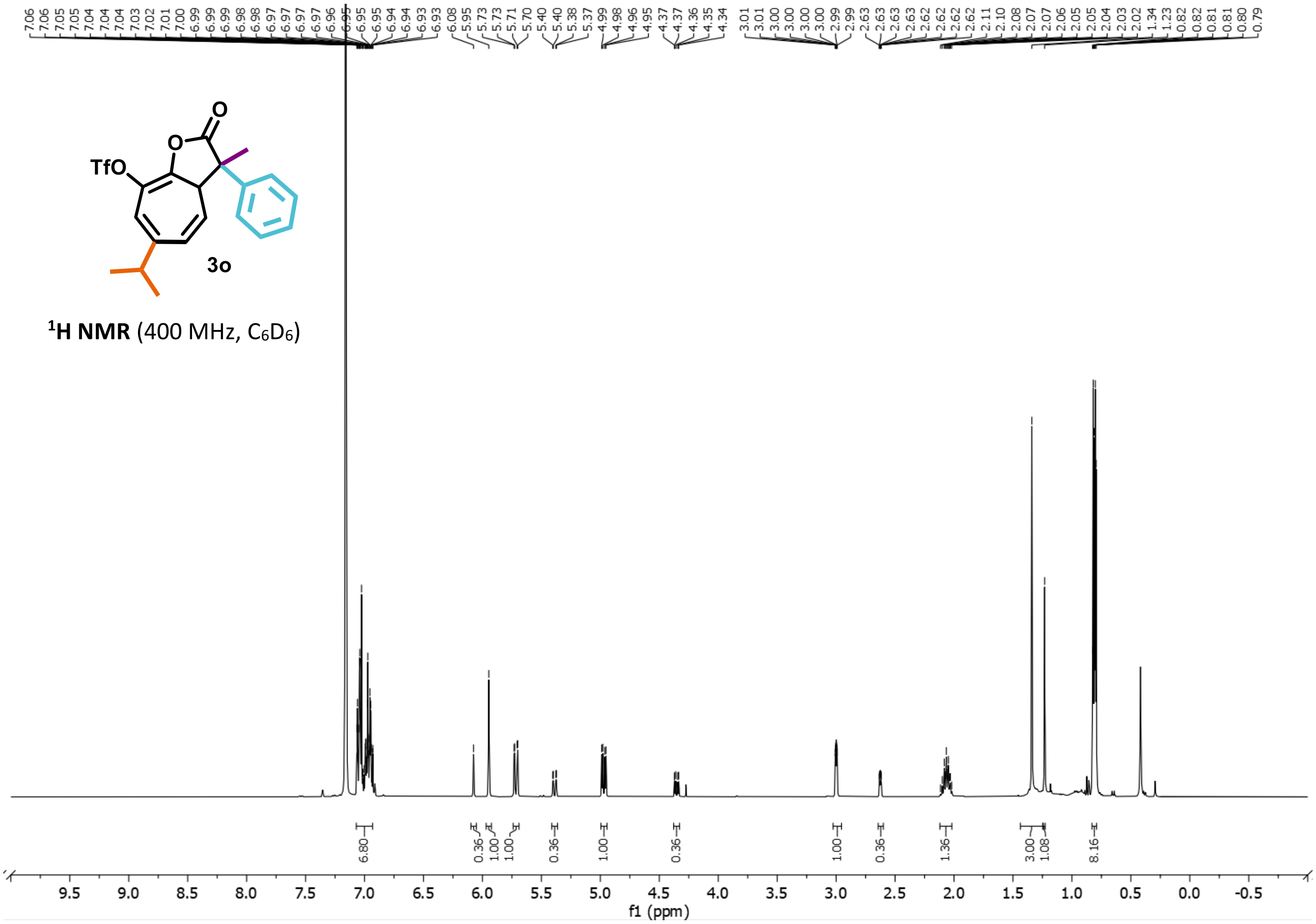

^{1}H NMR (400 MHz, C_6D_6)

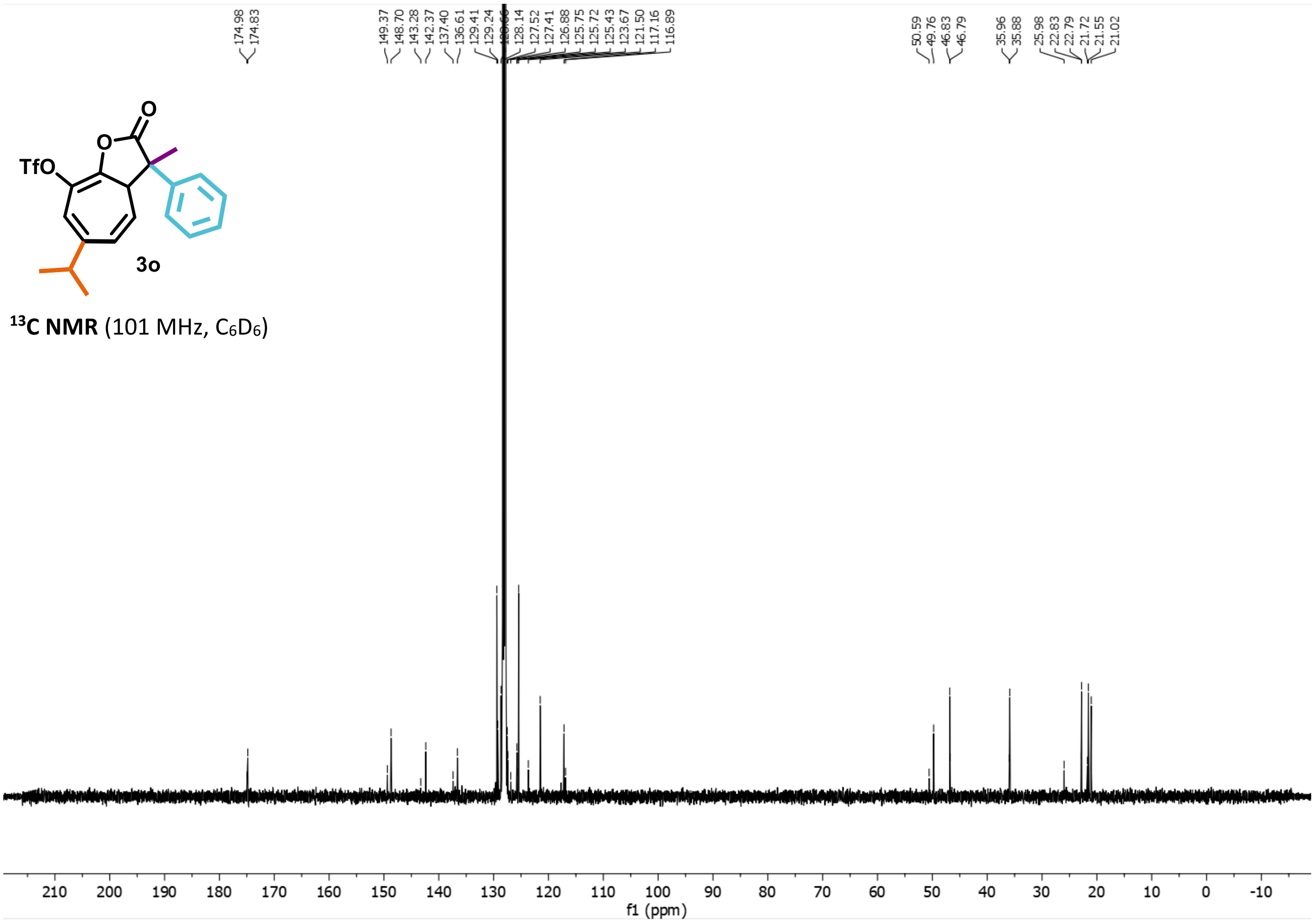

^{13}C NMR (101 MHz, C_6D_6)

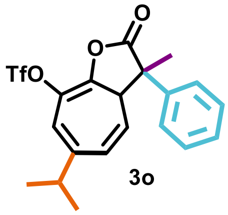

3o

^{13}**C NMR** (101 MHz, C_6D_6)

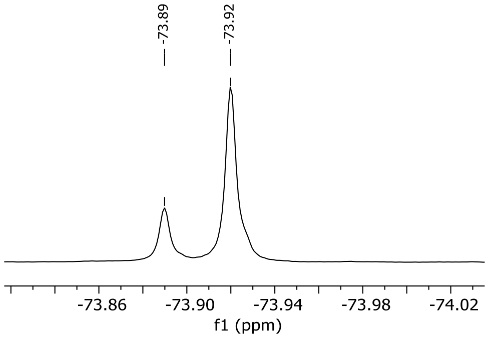

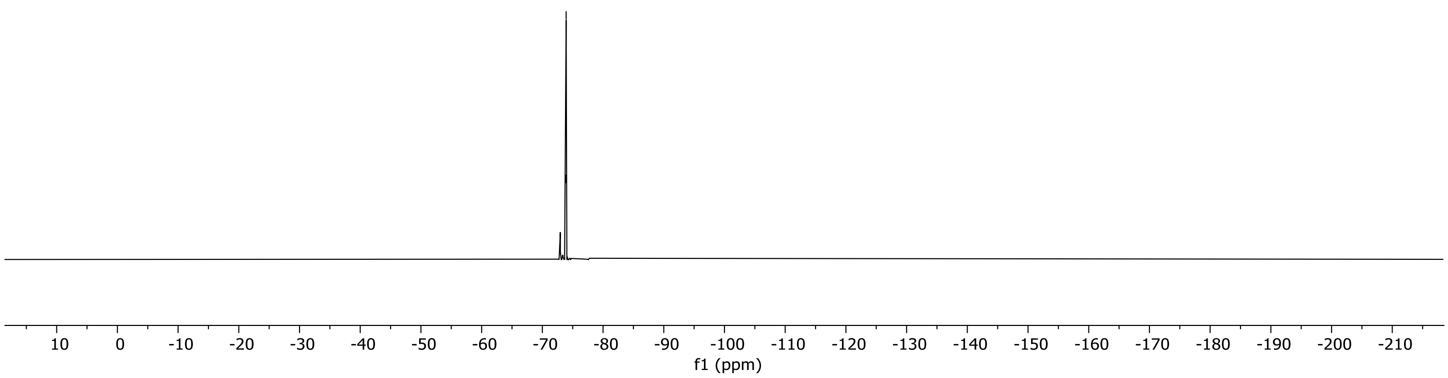

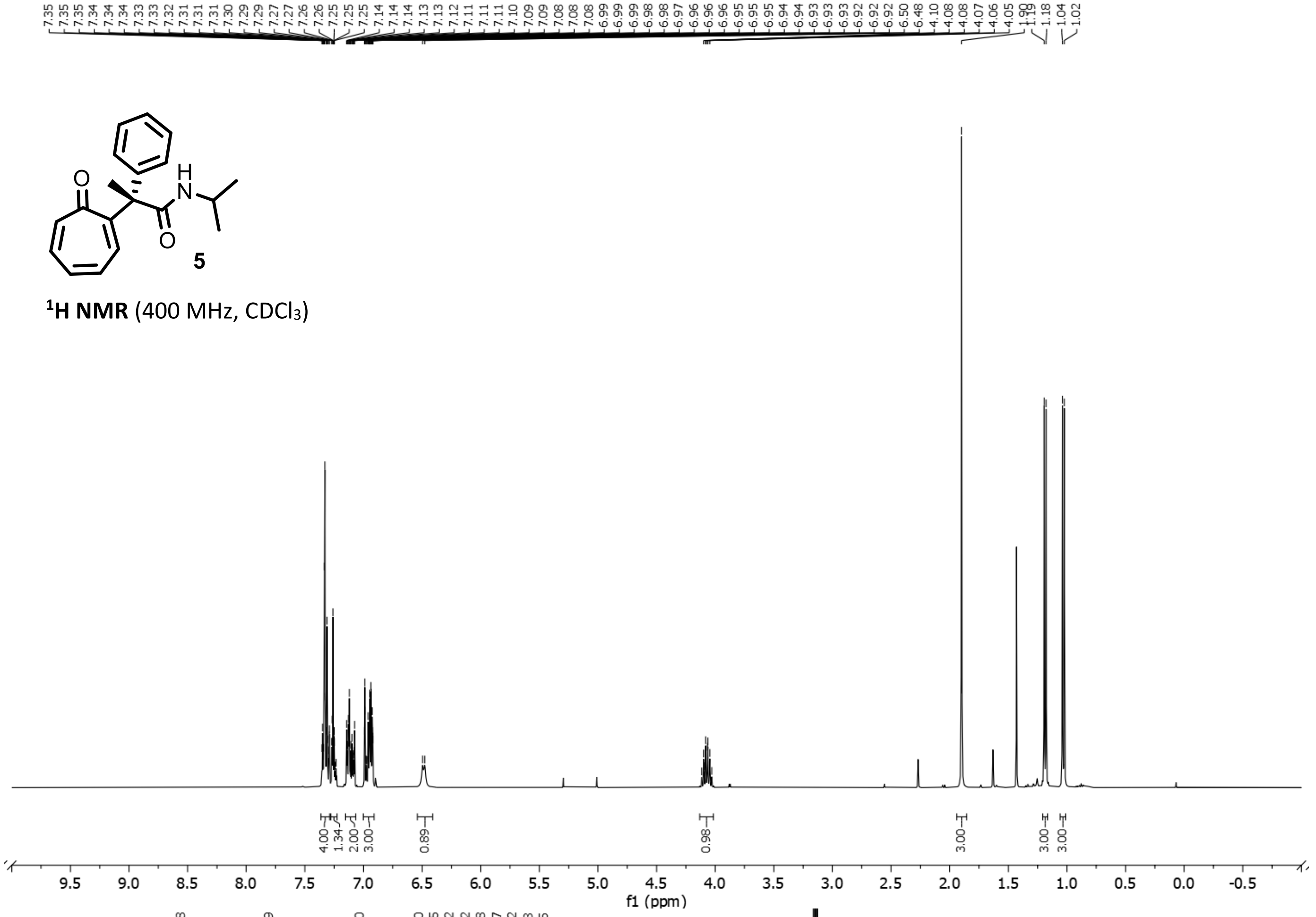
7.35 7.35 7.34 7.34 7.34 7.33 7.33 7.32 7.31 7.31 7.31 7.30 7.29 7.29 7.27 7.27 7.26 7.26 7.25 7.25 7.25 7.14 7.14 7.14 7.13 7.13 7.12 7.11 7.11 7.10 7.09 7.09 7.08 7.08 7.08 6.99 6.99 6.99 6.98 6.98 6.97 6.96 6.96 6.96 6.95 6.95 6.94 6.94 6.93 6.93 6.93 6.92 6.92 6.92 6.50 6.48 4.10 4.08 4.07 4.06 4.05 1.90 1.19 1.18 1.04 1.02
O
H
N
O
5
¹H NMR (400 MHz, CDCl₃)
4.00 1.34 2.00 3.00 0.89 0.98 3.00 3.00 3.00
9.5 9.0 8.5 8.0 7.5 7.0 6.5 6.0 5.5 5.0 4.5 4.0 3.5 3.0 2.5 2.0 1.5 1.0 0.5 0.0 -0.5
f1 (ppm)

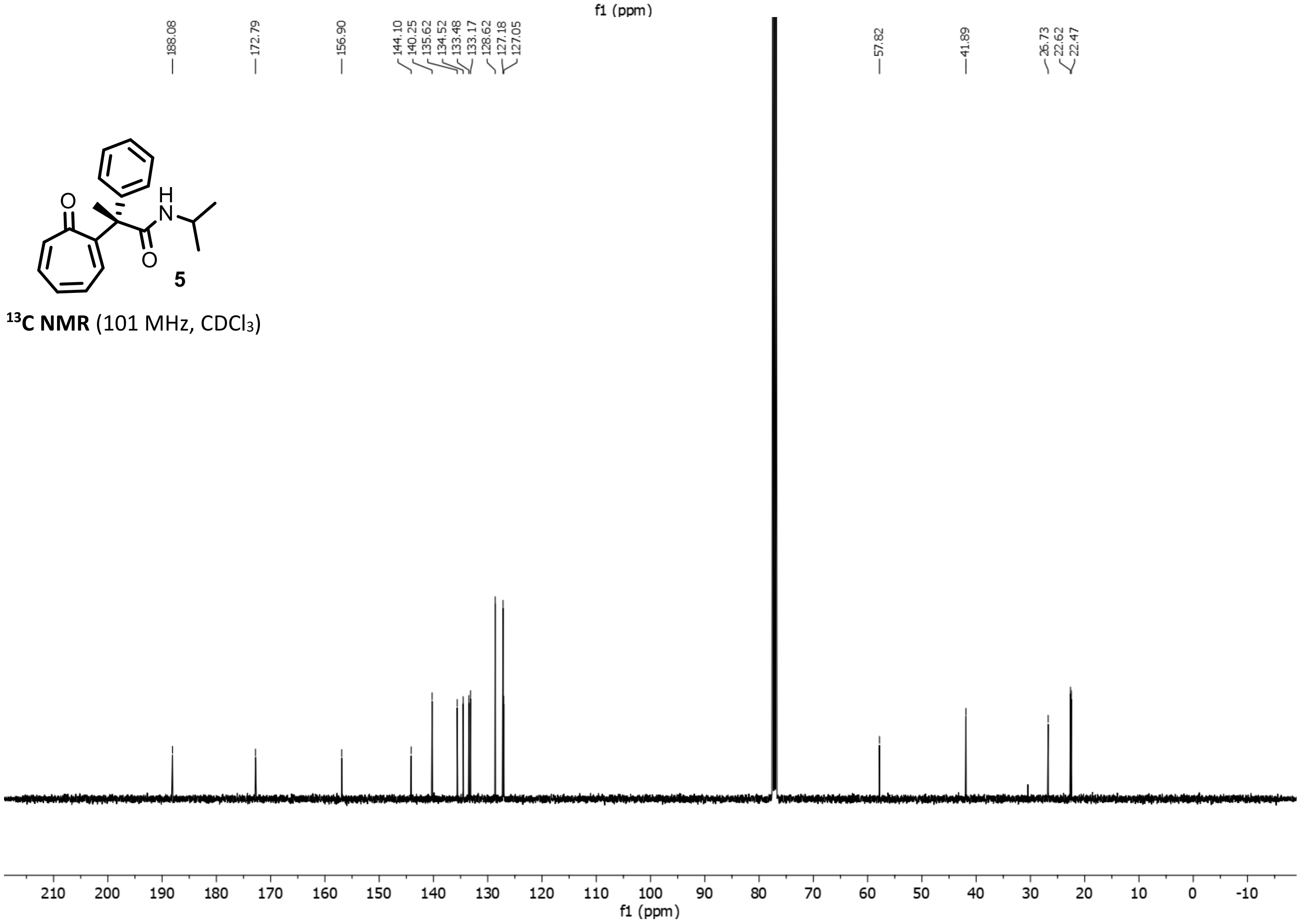
188.08
172.79
156.90
144.10 140.25 135.62 134.52 133.48 133.17 128.62 127.18 127.05
57.82
41.89
26.73 22.62 22.47
O
H
N
O
5
¹³C NMR (101 MHz, CDCl₃)
210 200 190 180 170 160 150 140 130 120 110 100 90 80 70 60 50 40 30 20 10 0 -10
f1 (ppm)

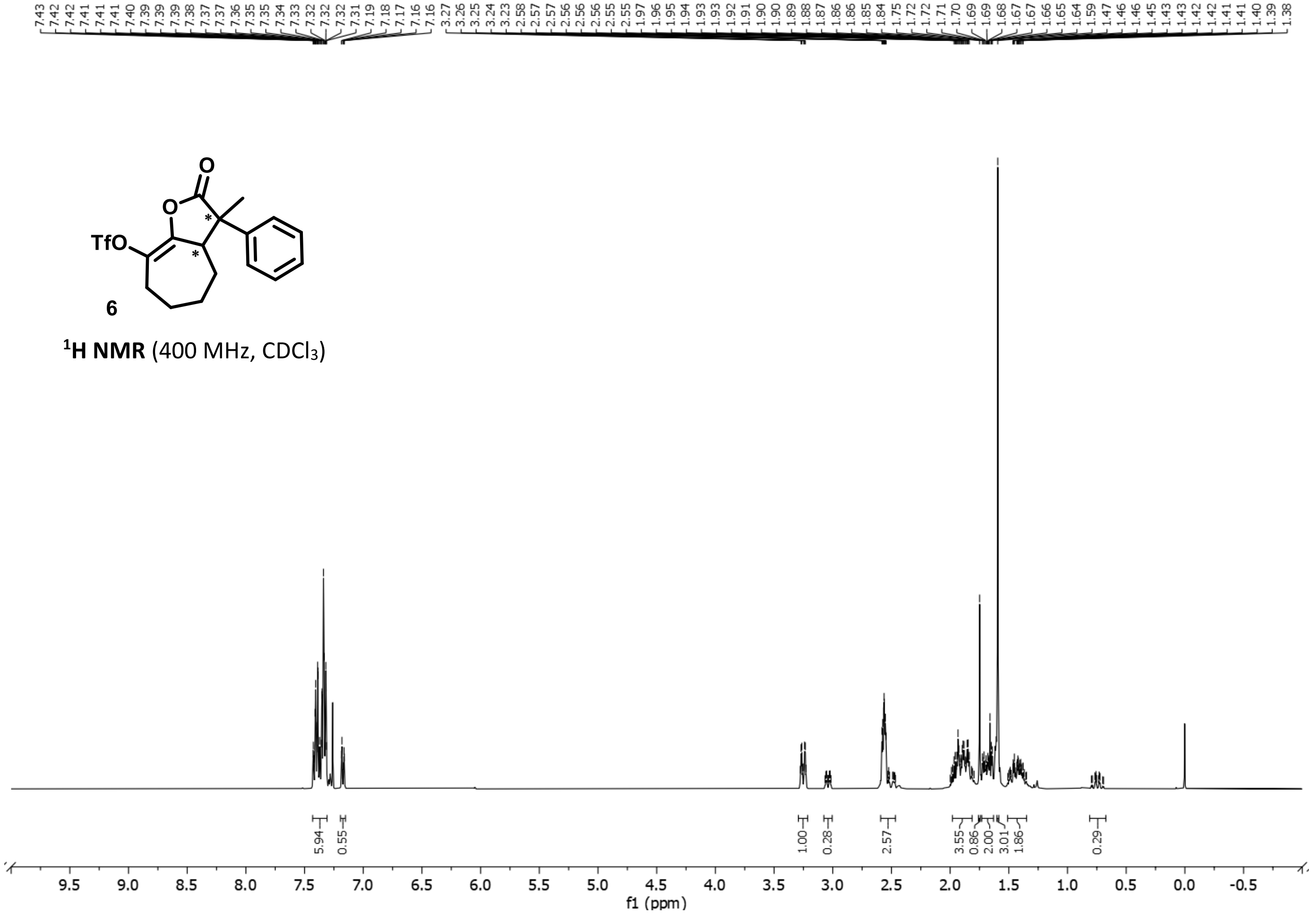
7.43 7.42 7.42 7.41 7.41 7.40 7.39 7.39 7.39 7.38 7.37 7.37 7.36 7.35 7.35 7.34 7.33 7.32 7.32 7.32 7.31 7.19 7.18 7.17 7.16 7.16 3.27 3.26 3.25 3.24 3.23 2.58 2.57 2.57 2.56 2.56 2.56 2.55 1.97 1.96 1.95 1.94 1.93 1.93 1.92 1.91 1.90 1.90 1.89 1.88 1.87 1.86 1.86 1.85 1.84 1.75 1.72 1.72 1.71 1.70 1.69 1.69 1.68 1.67 1.67 1.66 1.65 1.64 1.59 1.47 1.46 1.45 1.43 1.43 1.42 1.42 1.41 1.40 1.39 1.38
O
O
TfO
*
*
6
¹H NMR (400 MHz, CDCl₃)
5.94
0.55
1.00
0.28
2.57
3.55
0.86
2.00
3.01
1.86
0.29
9.5 9.0 8.5 8.0 7.5 7.0 6.5 6.0 5.5 5.0 4.5 4.0 3.5 3.0 2.5 2.0 1.5 1.0 0.5 0.0 -0.5
f1 (ppm)

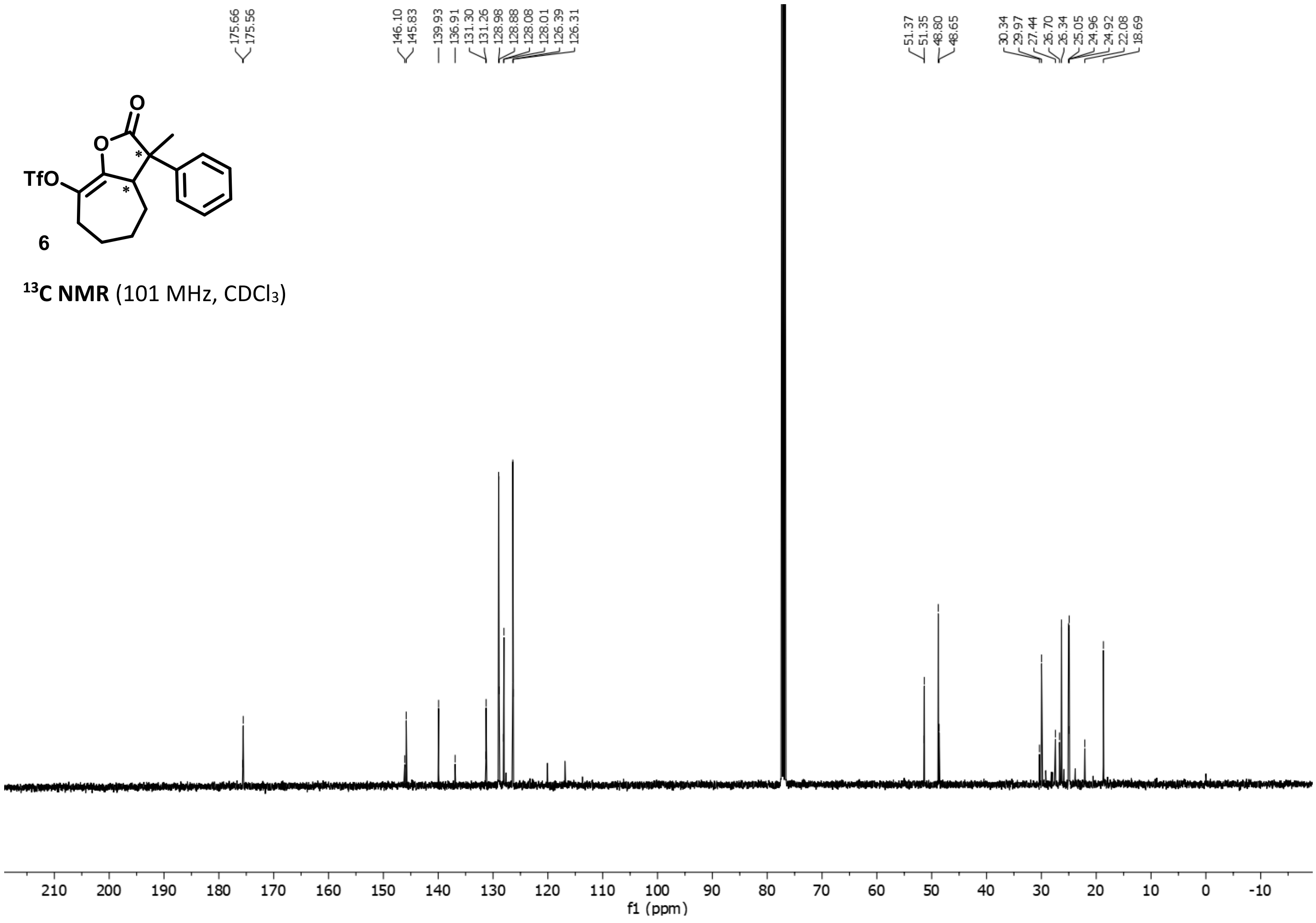
175.66 175.56
146.10 145.83
139.93
136.91
131.30 131.26
128.98 128.88 128.08 128.01
126.39 126.31
51.37 51.35 48.80 48.65
30.34 29.97 27.44 26.70 26.34 25.05 24.96 24.92 22.08 18.69
O
O
TfO
*
*
6
¹³C NMR (101 MHz, CDCl₃)
210 200 190 180 170 160 150 140 130 120 110 100 90 80 70 60 50 40 30 20 10 0 -10
f1 (ppm)

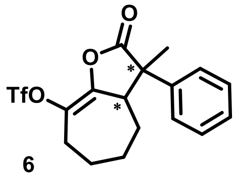

6

^{19}F NMR (376 MHz, $CDCl_3$)

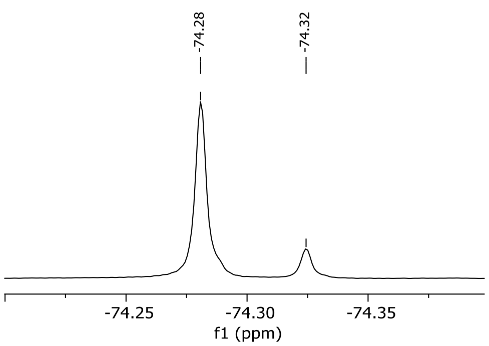

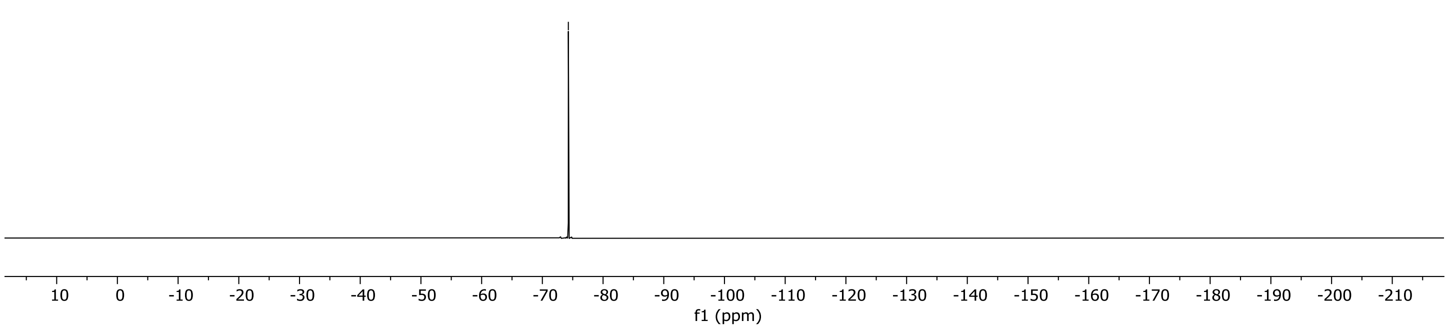

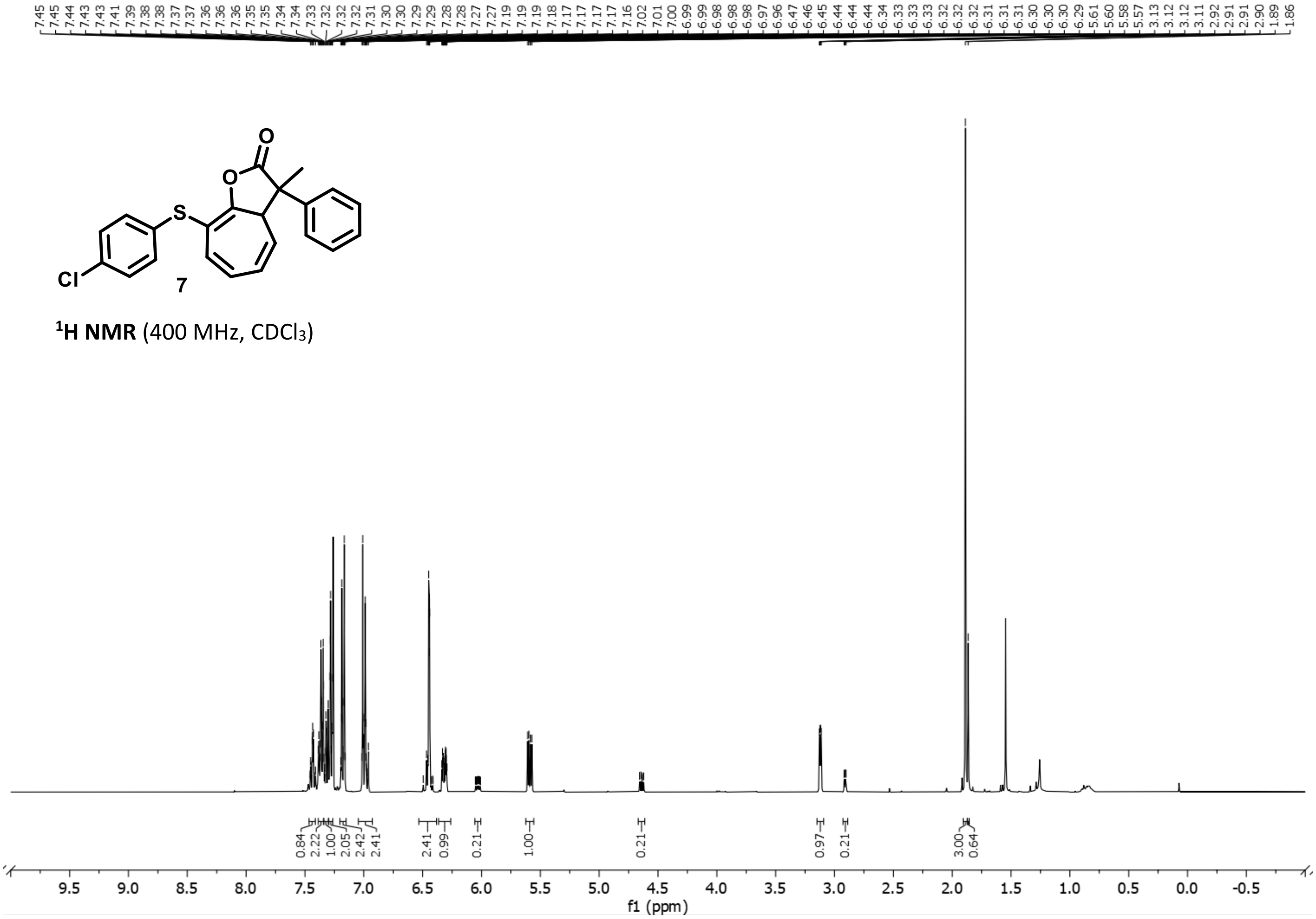

7
¹H NMR (400 MHz, CDCl₃)
f1 (ppm)

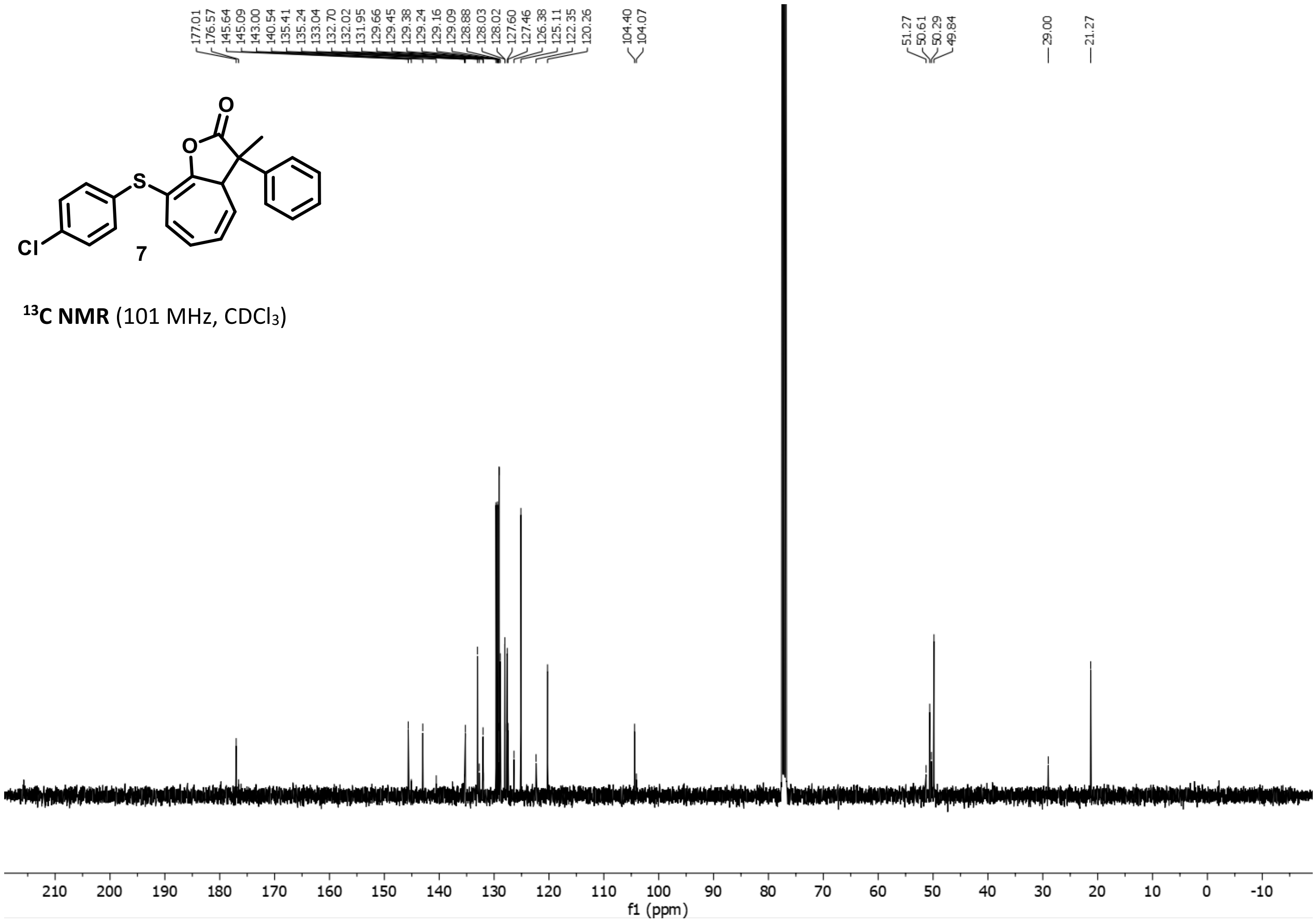

7
¹³C NMR (101 MHz, CDCl₃)
f1 (ppm)

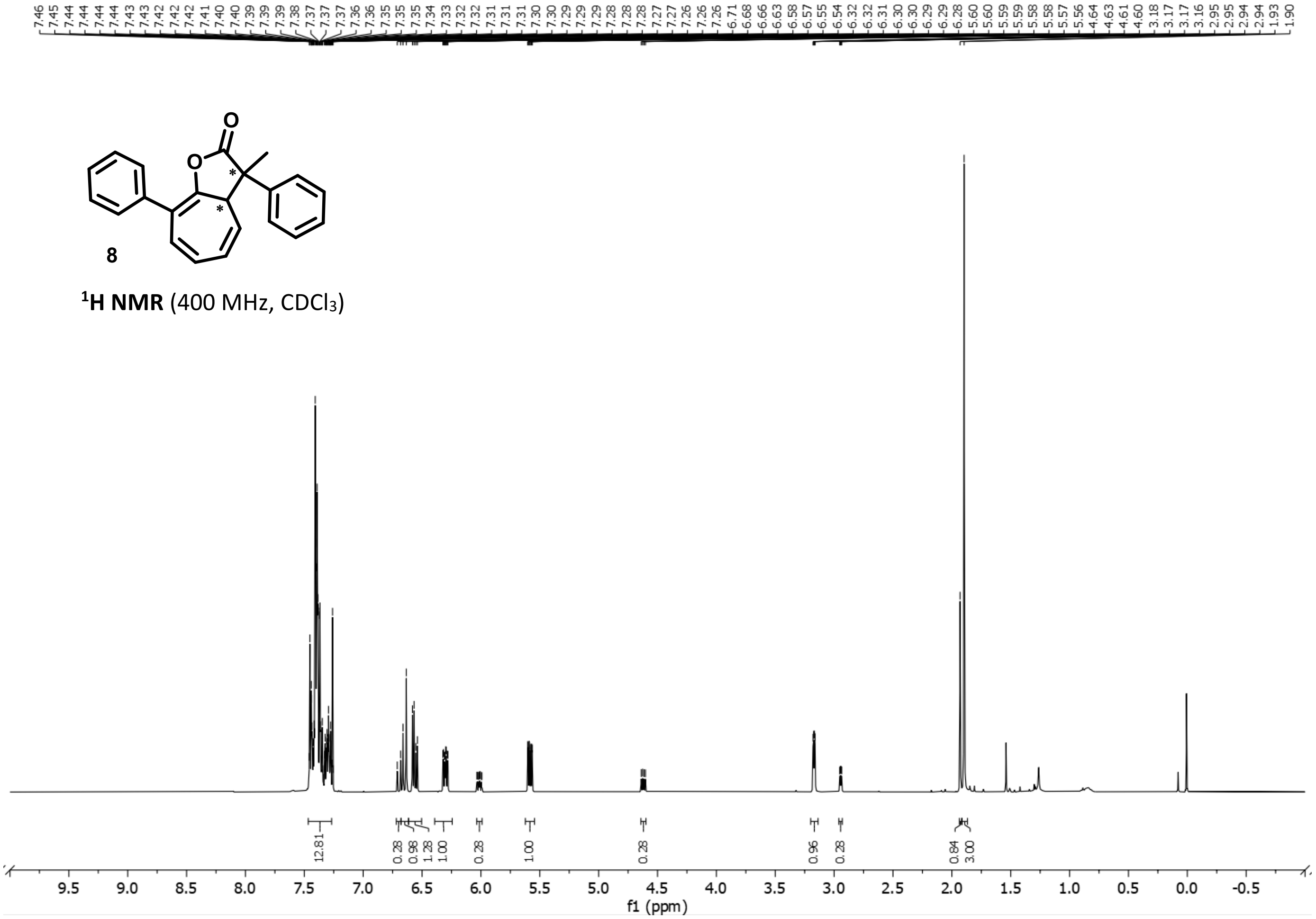
7.46 7.45 7.44 7.44 7.44 7.43 7.43 7.42 7.42 7.42 7.41 7.40 7.40 7.39 7.39 7.39 7.38 7.37 7.37 7.37 7.36 7.36 7.35 7.35 7.35 7.34 7.33 7.32 7.31 7.31 7.31 7.30 7.30 7.29 7.29 7.29 7.28 7.28 7.28 7.27 7.27 7.26 7.26 6.71 6.68 6.66 6.63 6.58 6.57 6.55 6.54 6.32 6.31 6.30 6.30 6.29 6.29 6.28 5.60 5.59 5.59 5.58 5.58 5.57 5.56 4.64 4.63 4.61 4.60 3.18 3.17 3.16 2.95 2.95 2.94 1.93 1.90
8
¹H NMR (400 MHz, CDCl₃)
12.81 0.28 0.98 1.28 1.00 0.28 1.00 0.28 0.96 0.28 0.84 3.00
9.5 9.0 8.5 8.0 7.5 7.0 6.5 6.0 5.5 5.0 4.5 4.0 3.5 3.0 2.5 2.0 1.5 1.0 0.5 0.0 -0.5
f1 (ppm)

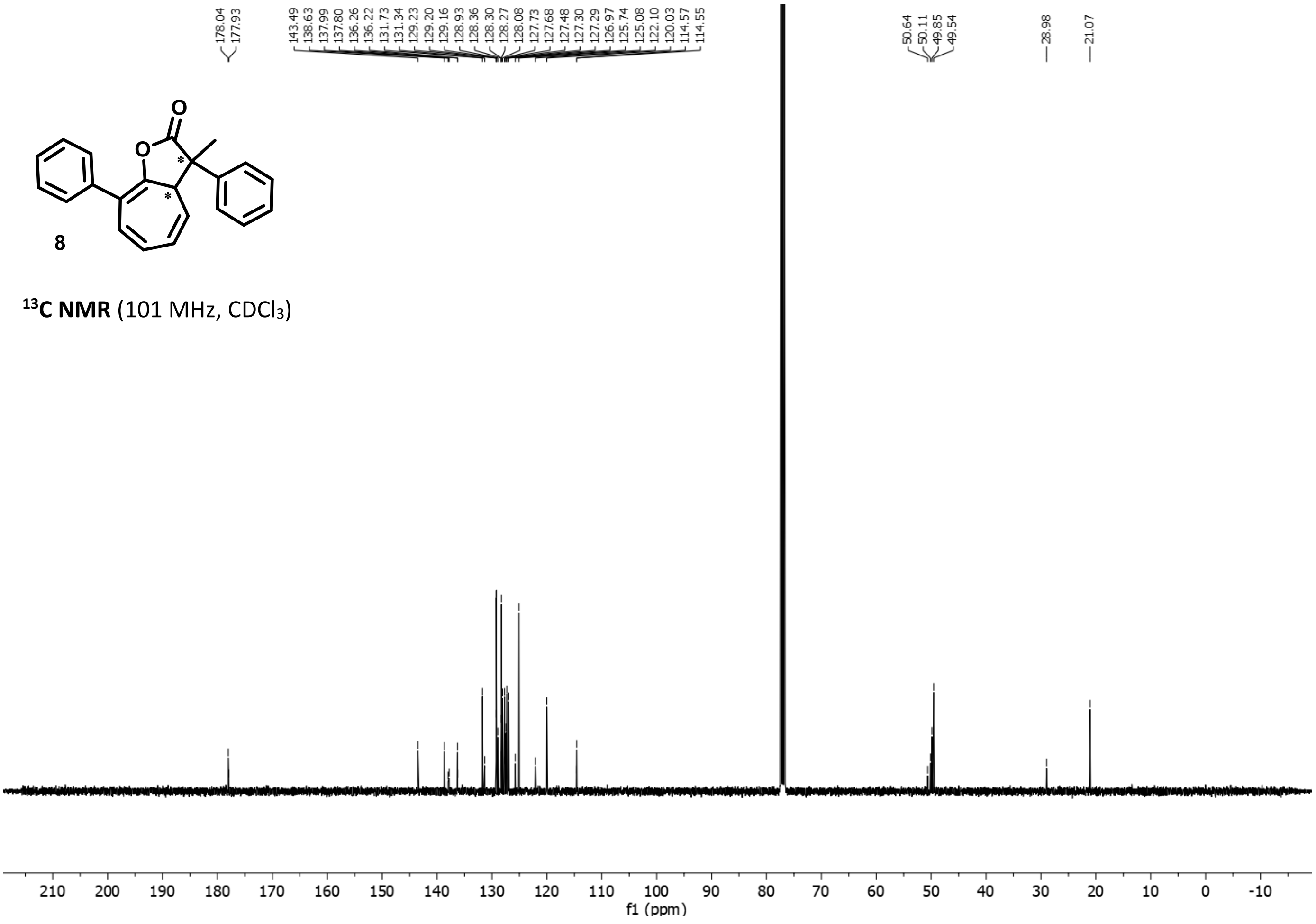
178.04 177.93
143.49 138.63 137.99 137.80 136.26 136.22 131.73 131.34 129.23 129.20 129.16 128.93 128.36 128.30 128.27 128.08 127.73 127.68 127.48 127.30 127.29 126.97 125.74 125.08 122.10 120.03 114.57 114.55
50.64 50.11 49.85 49.54
28.98
21.07
8
¹³C NMR (101 MHz, CDCl₃)
210 200 190 180 170 160 150 140 130 120 110 100 90 80 70 60 50 40 30 20 10 0 -10
f1 (ppm)

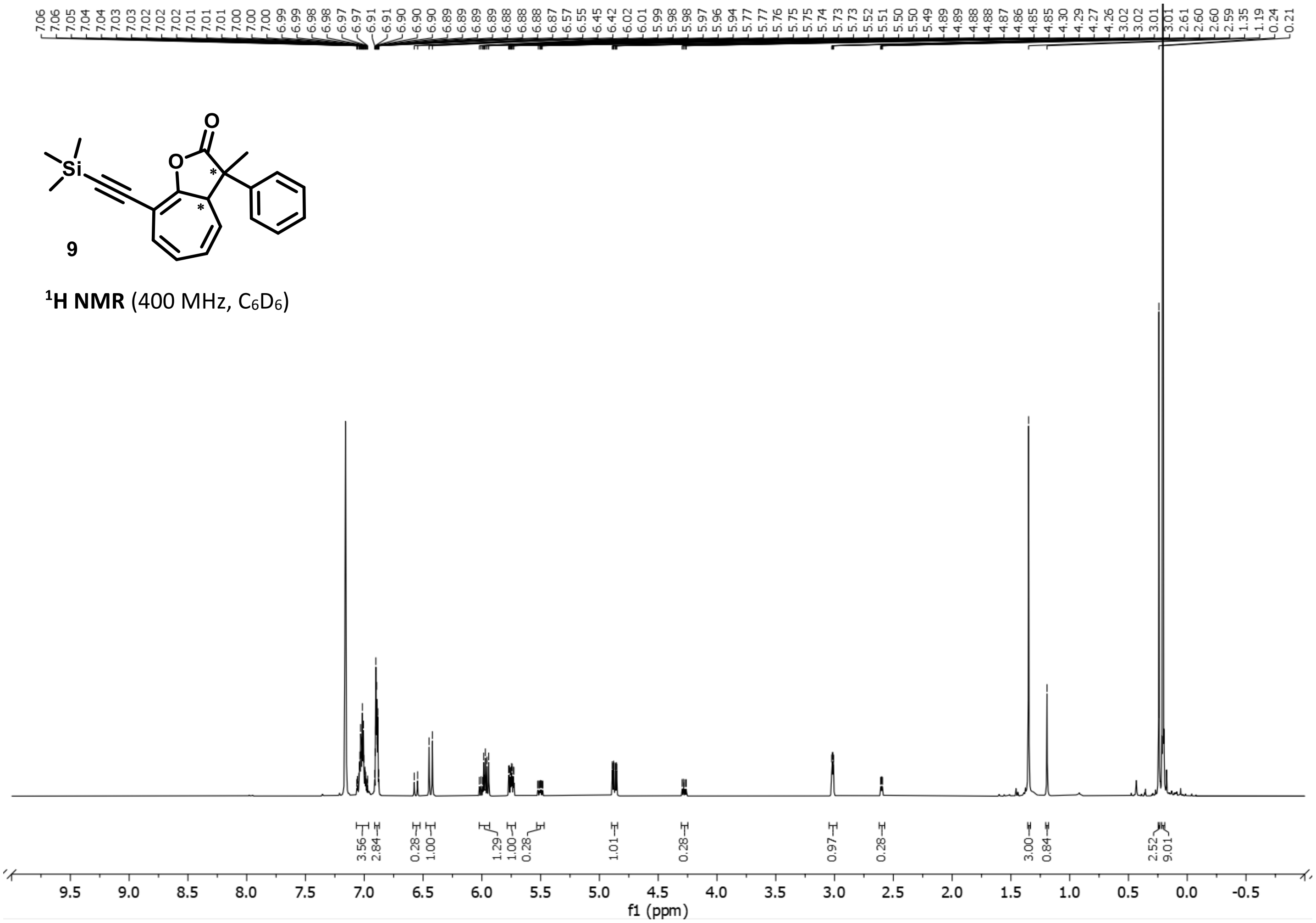
7.06 7.06 7.05 7.04 7.04 7.03 7.03 7.02 7.02 7.02 7.01 7.01 7.01 7.00 7.00 6.99 6.99 6.98 6.98 6.97 6.97 6.91 6.91 6.90 6.90 6.90 6.89 6.89 6.89 6.89 6.88 6.88 6.88 6.87 6.57 6.55 6.45 6.42 6.02 6.01 5.99 5.98 5.98 5.97 5.96 5.94 5.77 5.77 5.76 5.75 5.75 5.74 5.73 5.52 5.51 5.50 5.50 5.49 4.89 4.89 4.88 4.88 4.87 4.86 4.85 4.30 4.29 4.27 4.26 3.02 3.02 3.01 3.01 2.61 2.60 2.59 1.35 1.19 0.24 0.21
O
O
Si
*
*
9
¹H NMR (400 MHz, C6D6)
3.56 2.84 0.28 1.00 1.29 1.00 0.28 1.01 0.28 0.97 0.28 3.00 0.84 2.52 9.01
9.5 9.0 8.5 8.0 7.5 7.0 6.5 6.0 5.5 5.0 4.5 4.0 3.5 3.0 2.5 2.0 1.5 1.0 0.5 0.0 -0.5
f1 (ppm)

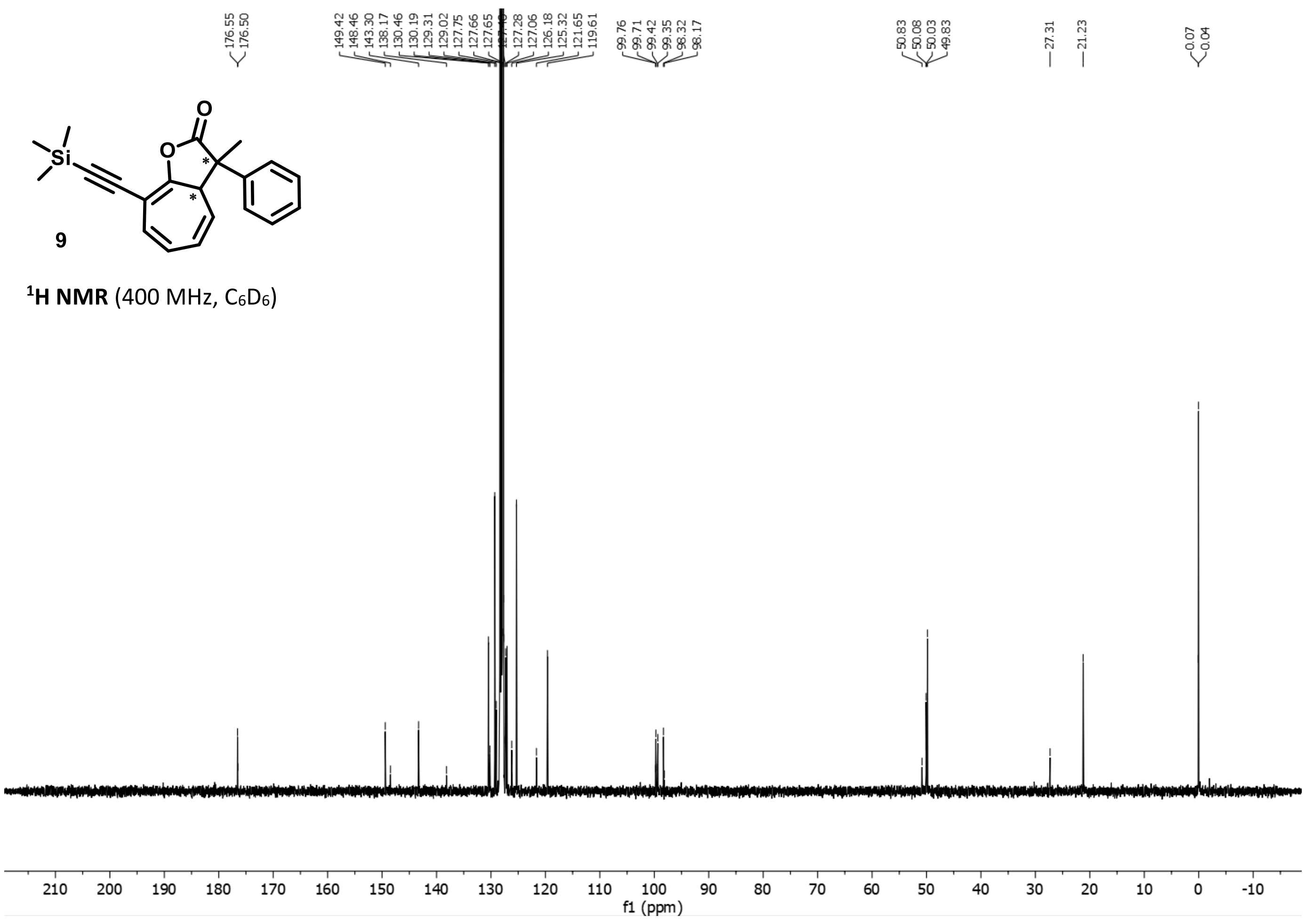
176.55 176.50
149.42 148.46 143.30 138.17 130.46 130.19 129.31 129.02 127.75 127.66 127.65 127.28 127.06 126.18 125.32 121.65 119.61
99.76 99.71 99.42 99.35 98.32 98.17
50.83 50.08 50.03 49.83
27.31
21.23
0.07 0.04
O
O
Si
*
*
9
¹H NMR (400 MHz, C6D6)
210 200 190 180 170 160 150 140 130 120 110 100 90 80 70 60 50 40 30 20 10 0 -10
f1 (ppm)

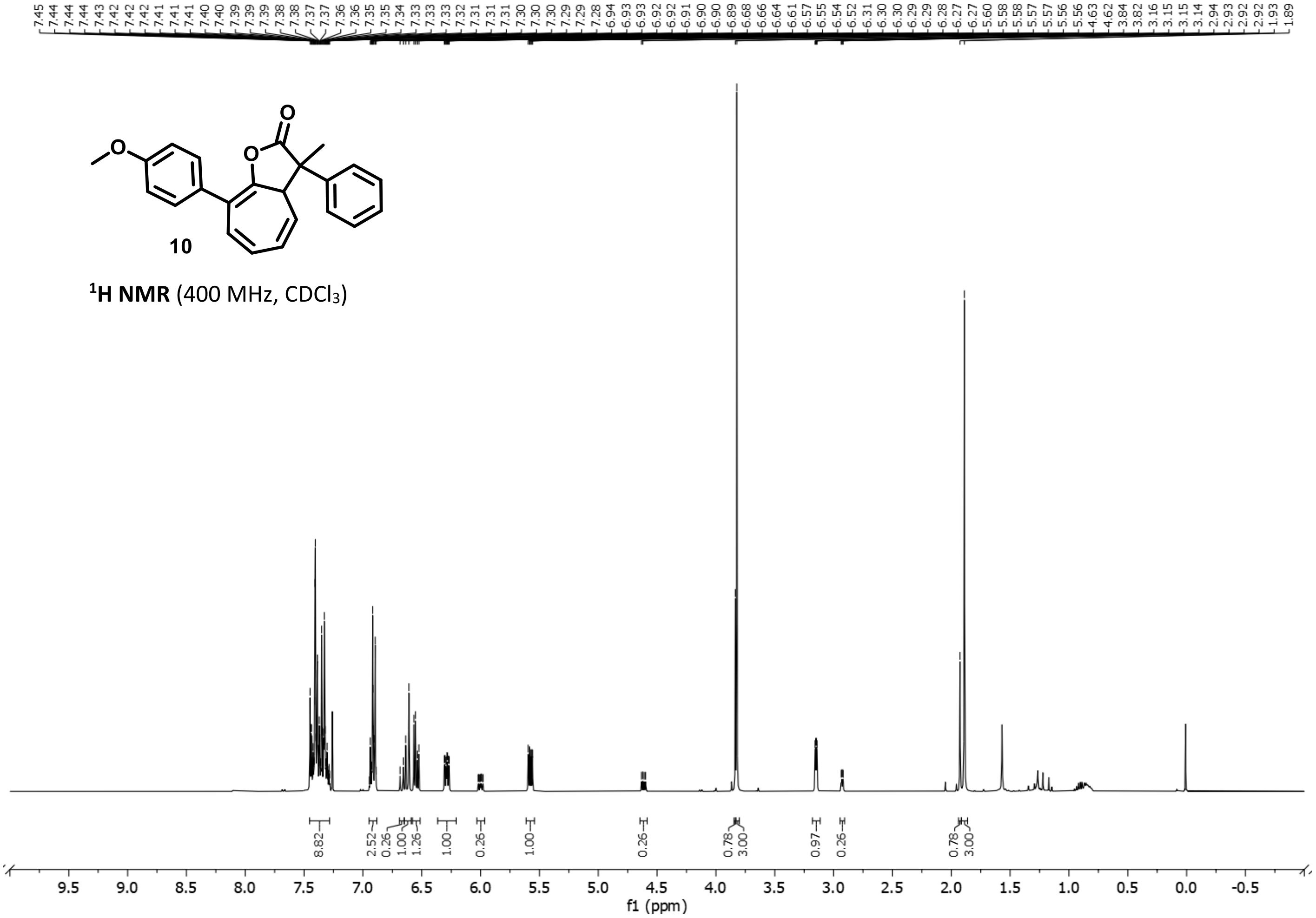
10
1H NMR (400 MHz, CDCl3)
f1 (ppm)

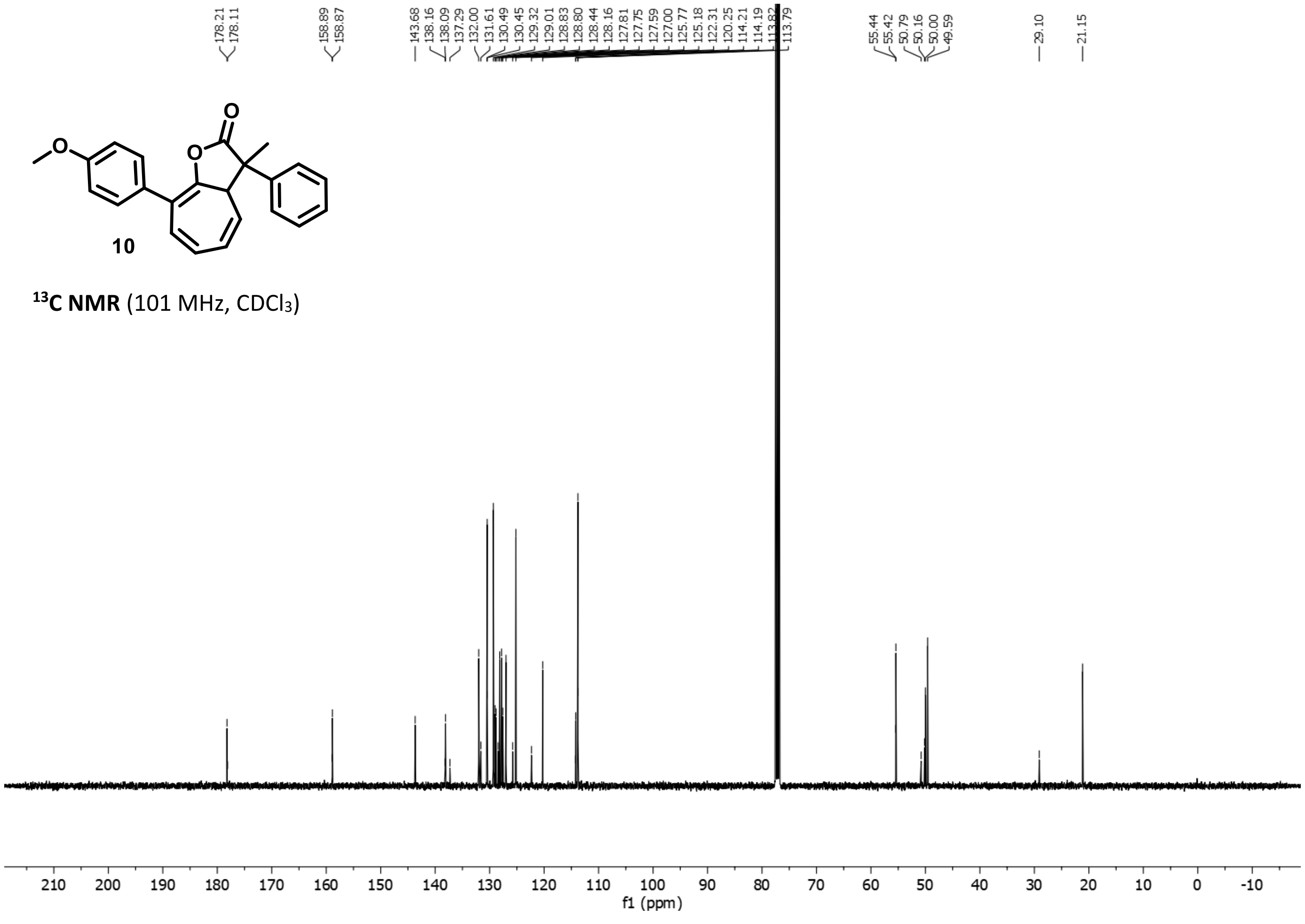
10
13C NMR (101 MHz, CDCl3)
f1 (ppm)

9. HPLC

HPLC chromatograms of racemic and asymmetric products 4

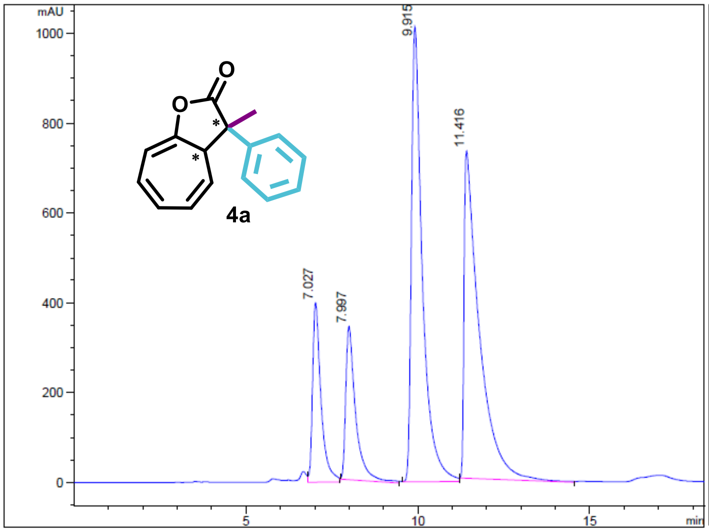

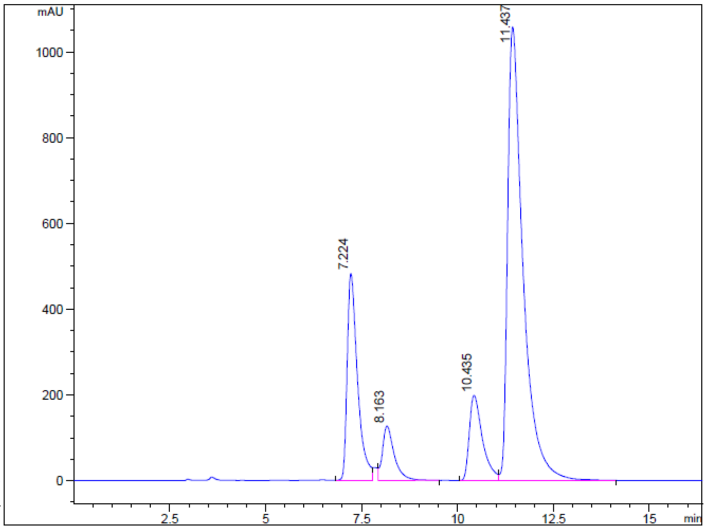

Signal 1: MWD1 E, Sig=280,16 Ref=360,100

Area %	Peak #	RT [min]	Type	Width [min]	Area	Area %	Name
11.608	1	7.027	VV	0.251	6806.872	11.608	
11.655	2	7.997	MM	0.334	6834.705	11.655	
38.520	3	9.915	VV	0.322	22588.799	38.520	
38.217	4	11.416	MM	0.512	22411.369	38.217	

Signal 1: MWD1 E, Sig=280,16 Ref=360,100

Area %	Peak #	RT [min]	Type	Width [min]	Area	Area %	Name
19.664	1	7.224	VV	0.282	9091.802	19.664	
5.974	2	8.163	VB	0.320	2762.029	5.974	
10.266	3	10.435	BV	0.357	4746.503	10.266	
64.096	4	11.437	VB	0.411	29635.500	64.096	

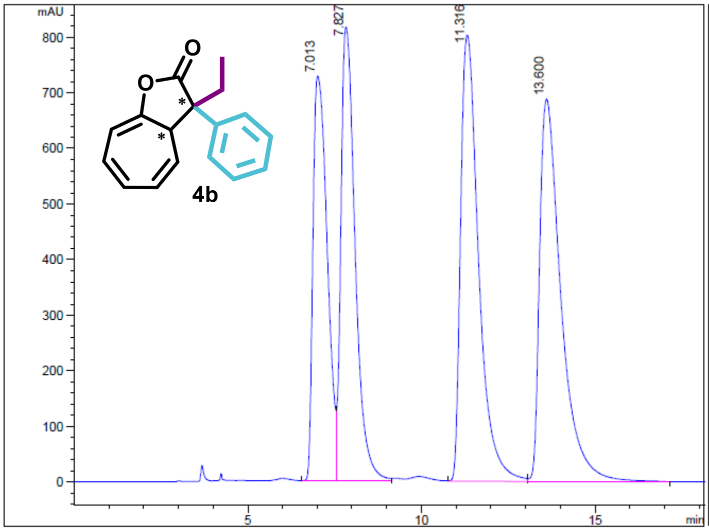

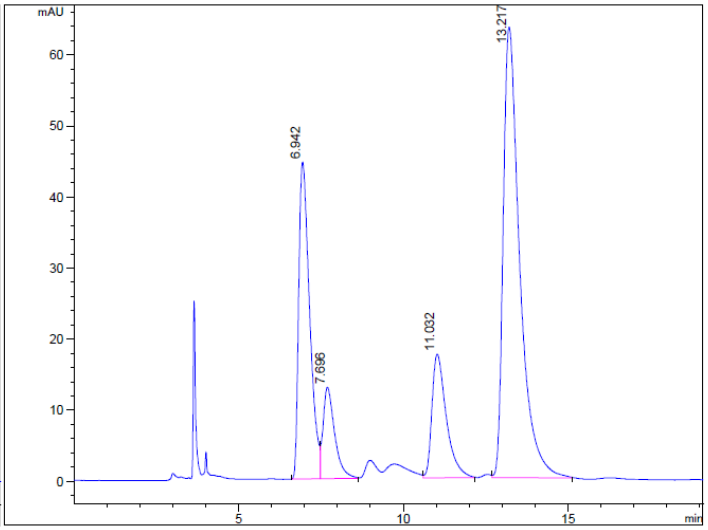

Signal 1: MWD1 E, Sig=280,16 Ref=360,100

Area %	Peak #	RT [min]	Type	Width [min]	Area	Area %	Name
20.234	1	7.013	VV	0.451	20683.666	20.234	
22.855	2	7.827	VV	0.442	23363.225	22.855	
28.256	3	11.316	VV	0.554	28883.480	28.256	
28.655	4	13.600	VB	0.643	29291.314	28.655	

Signal 1: MWD1 E, Sig=280,16 Ref=360,100

Area %	Peak #	RT [min]	Type	Width [min]	Area	Area %	Name
25.172	1	6.942	BV	0.351	1027.687	25.172	
7.819	2	7.696	VB	0.365	319.241	7.819	
12.928	3	11.032	VB	0.454	527.803	12.928	
54.080	4	13.217	VB	0.520	2207.913	54.080	

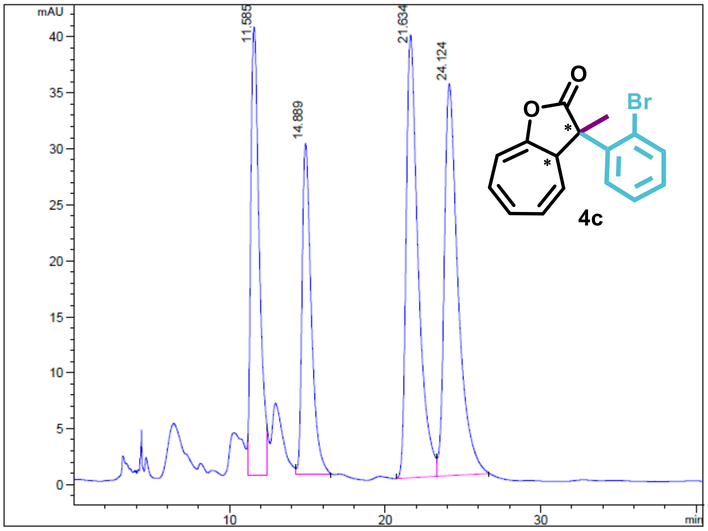

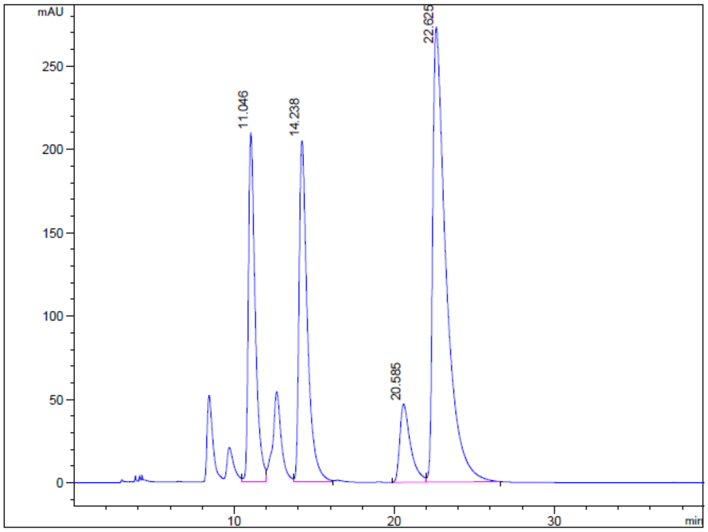

Signal 1: MWD1 E, Sig=280,16 Ref=360,100

Area %	Peak #	RT [min]	Type	Width [min]	Area	Area %	Name
21.568	1	11.585	MF	0.632	1518.597	21.568	
17.359	2	14.889	FM	0.689	1222.265	17.359	
30.384	3	21.634	BB	0.790	2139.346	30.384	
30.688	4	24.124	BB	0.884	2160.745	30.688	

Signal 1: MWD1 E, Sig=280,16 Ref=360,100

Area %	Peak #	RT [min]	Type	Width [min]	Area	Area %	Name
19.650	1	11.046	VV	0.447	6233.185	19.650	
23.338	2	14.238	VB	0.538	7403.038	23.338	
6.914	3	20.585	BV	0.686	2193.234	6.914	
50.098	4	22.625	VB	0.835	15891.396	50.098	

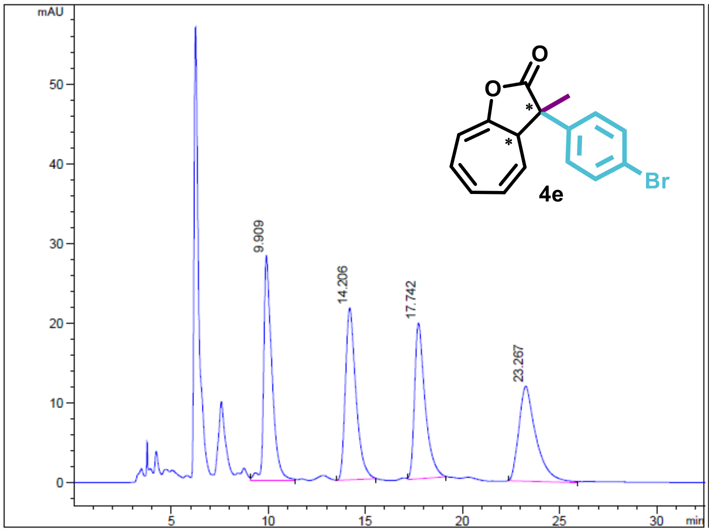

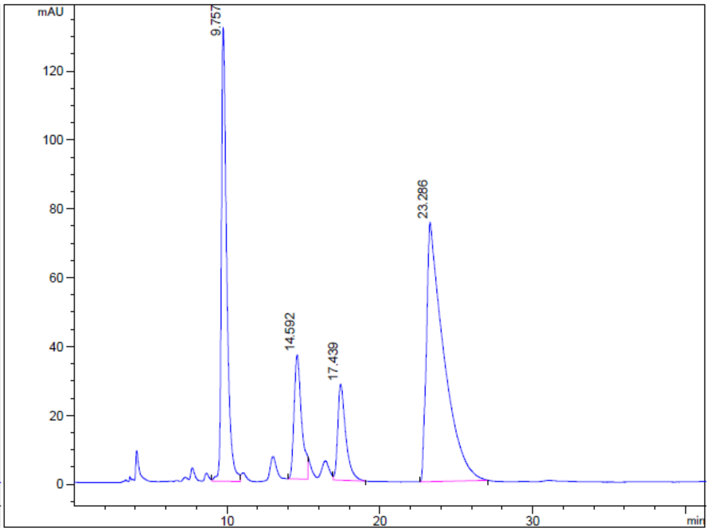

Signal 1: MWD1 B, Sig=254,16 Ref=360,100

Area %	Peak #	RT [min]	Type	Width [min]	Area	Area %	Name
26.136	1	9.909	MM	0.479	807.231	26.136	
26.288	2	14.206	VB	0.553	811.947	26.288	
23.788	3	17.742	BB	0.547	734.705	23.788	
23.788	4	23.267	MM	1.026	734.733	23.788	

Signal 1: MWD1 B, Sig=254,16 Ref=360,100

Area %	Peak #	RT [min]	Type	Width [min]	Area	Area %	Name
28.254	1	9.757	VV	0.361	3220.184	28.254	
10.745	2	14.592	MF	0.566	1224.626	10.745	
8.905	3	17.439	VB	0.536	1014.877	8.905	
52.097	4	23.286	BB	1.010	5937.578	52.097	

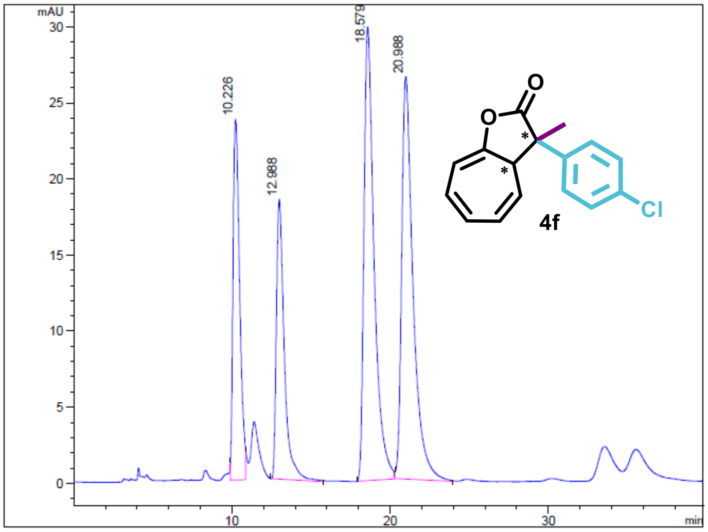

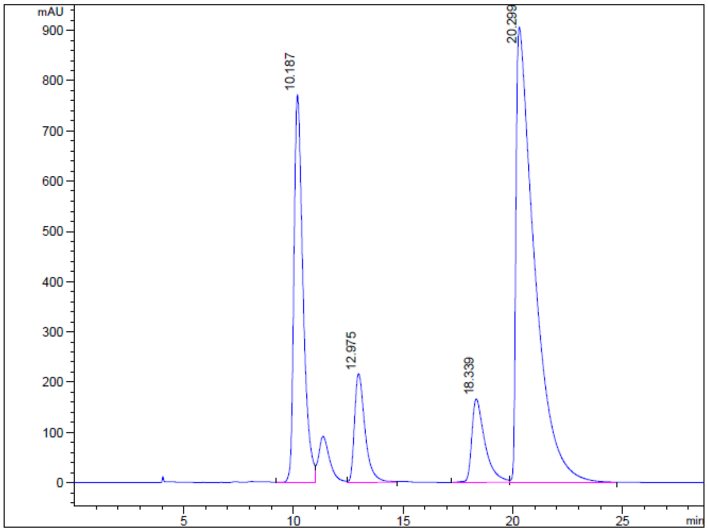

Signal 1: MWD1 E, Sig=280,16 Ref=360,100

Area %	Peak #	RT [min]	Type	Width [min]	Area	Area %	Name
17.269	1	10.226	MF	0.485	689.724	17.269	
16.047	2	12.988	MM	0.581	640.919	16.047	
33.418	3	18.579	BB	0.664	1334.741	33.418	
33.267	4	20.988	MM	0.837	1328.697	33.267	

Signal 1: MWD1 E, Sig=280,16 Ref=360,100

Area %	Peak #	RT [min]	Type	Width [min]	Area	Area %	Name
24.902	1	10.187	VV	0.442	22322.045	24.902	
8.098	2	12.975	VB	0.507	7259.488	8.098	
7.778	3	18.339	BV	0.612	6972.331	7.778	
59.222	4	20.299	VB	0.820	53087.215	59.222	

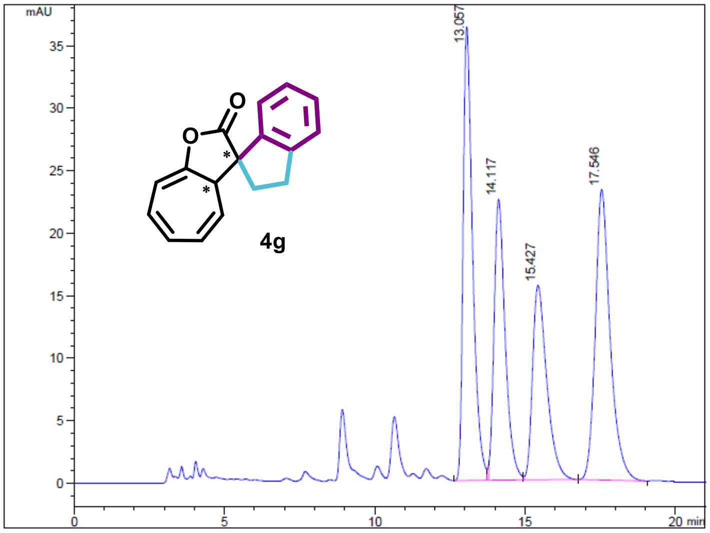

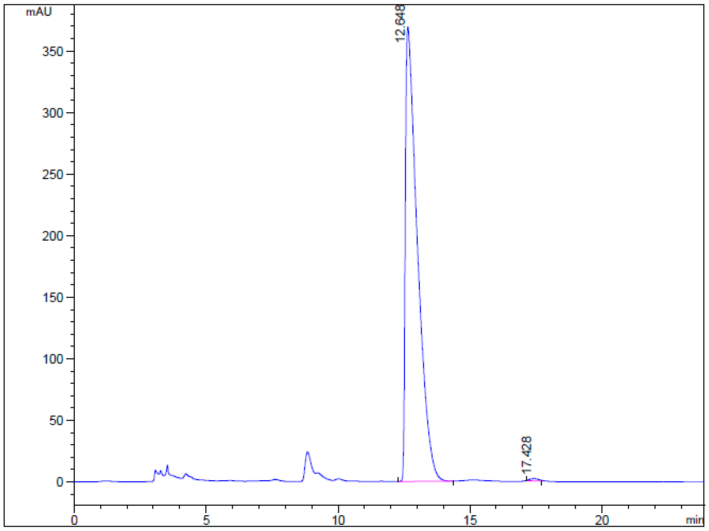

Signal 1: VWD1 A, Wavelength=280 nm

Area %	Peak #	RT [min]	Type	Width [min]	Area	Area %	Name
29.596	1	13.057	BV	0.330	794.878	29.596	
21.486	2	14.117	FM	0.428	577.054	21.486	
19.119	3	15.427	VB	0.494	513.489	19.119	
29.800	4	17.546	BB	0.505	800.351	29.800	

Signal 1: VWD1 A, Wavelength=280 nm

Area %	Peak #	RT [min]	Type	Width [min]	Area	Area %	Name
99.633	1	12.648	BB	0.464	11745.881	99.633	
0.367	2	17.428	MM	0.387	43.244	0.367	

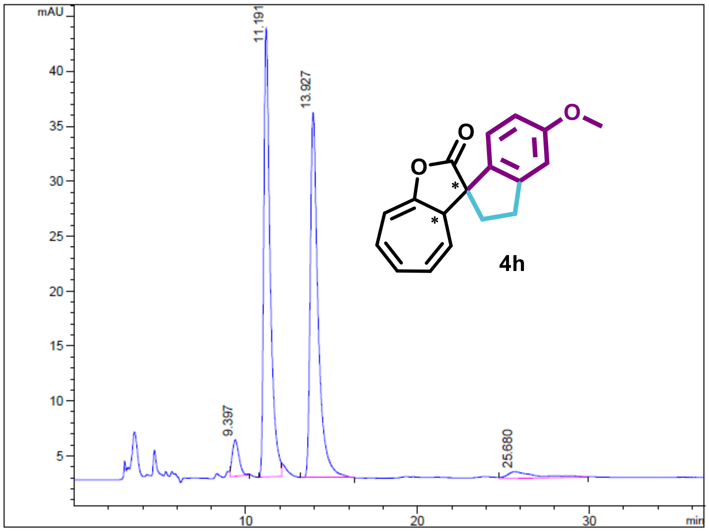

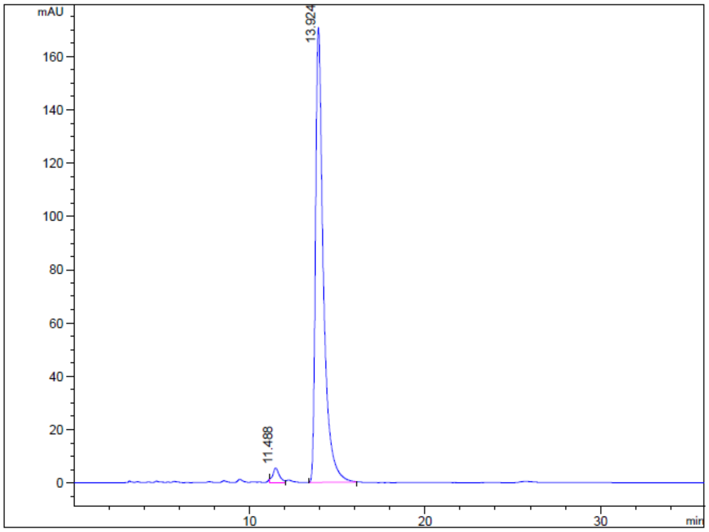

Signal 1: MWD1 B, Sig=254,16 Ref=360,100

Area %	Peak #	RT [min]	Type	Width [min]	Area	Area %	Name
3.967	1	9.397	FM	0.452	89.994	3.967	
46.406	2	11.191	MF	0.429	1052.824	46.406	
46.369	3	13.927	MM	0.529	1051.993	46.369	
3.258	4	25.680	MM	2.021	73.921	3.258	

Signal 1: MWD1 B, Sig=254,16 Ref=360,100

Area %	Peak #	RT [min]	Type	Width [min]	Area	Area %	Name
2.562	1	11.488	FM	0.432	140.833	2.562	
97.438	2	13.924	BB	0.460	5355.657	97.438	

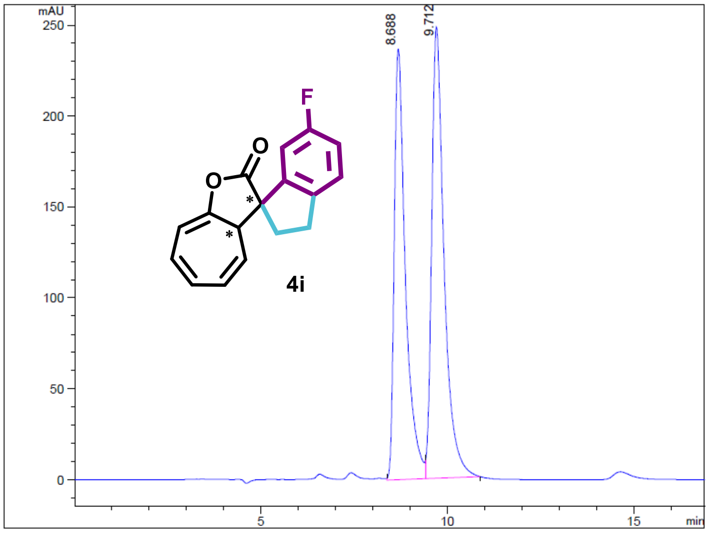

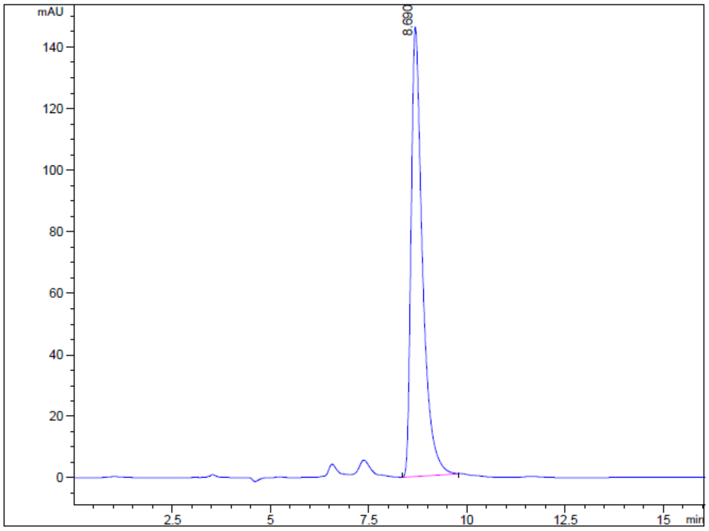

Signal 1: MWD1 E, Sig=280,16 Ref=360,100

Area %	Peak #	RT [min]	Type	Width [min]	Area	Area %	Name
46.566	1	8.688	MF	0.351	4994.691	46.566	
53.434	2	9.712	FM	0.385	5731.296	53.434	

Signal 1: MWD1 E, Sig=280,16 Ref=360,100

Area %	Peak #	RT [min]	Type	Width [min]	Area	Area %	Name
100.000	1	8.690	BB	0.301	2943.413	100.000	

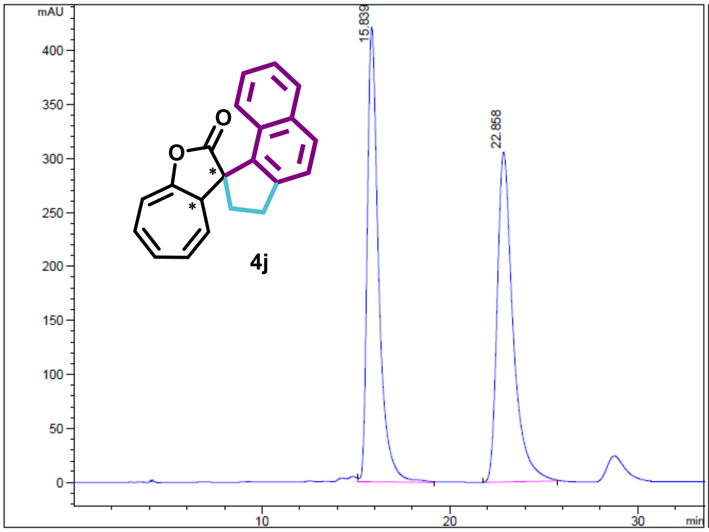

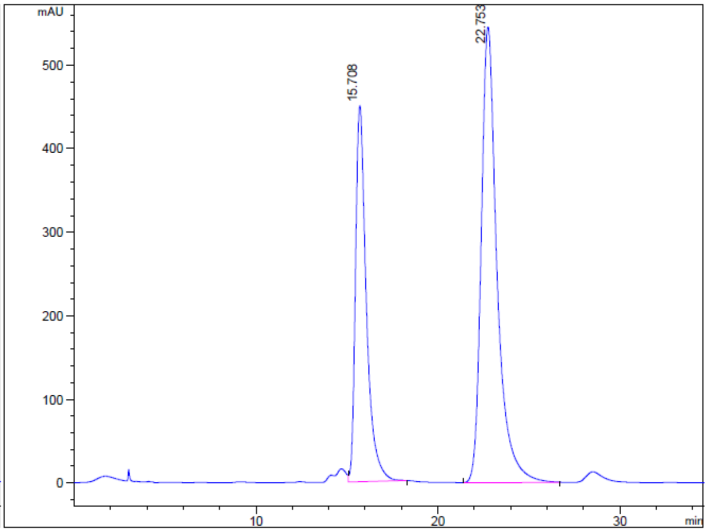

Signal 1: MWD1 F, Sig=280,16 Ref=360,100

Area %	Peak #	RT [min]	Type	Width [min]	Area	Area %	Name
49.789	1	15.839	FM	0.701	17715.160	49.789	
50.211	2	22.858	MM	0.975	17865.121	50.211	

Signal 1: MWD1 F, Sig=280,16 Ref=360,100

Area %	Peak #	RT [min]	Type	Width [min]	Area	Area %	Name
36.166	1	15.708	VB	0.624	18682.779	36.166	
63.834	2	22.753	BB	0.900	32975.496	63.834	

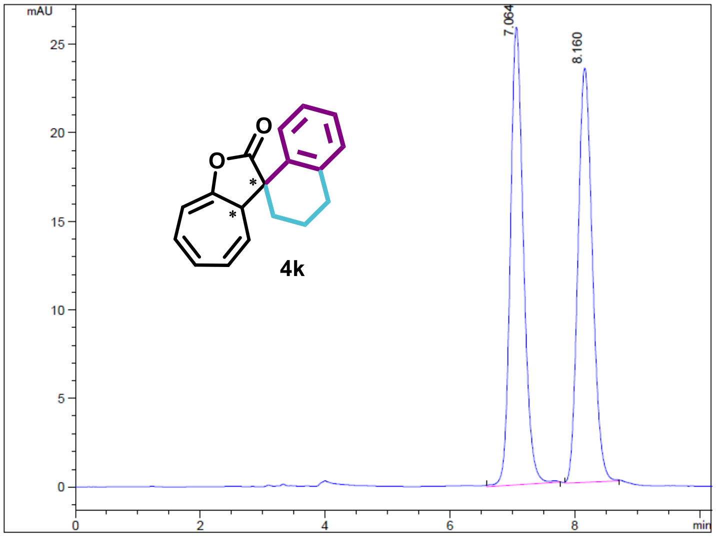

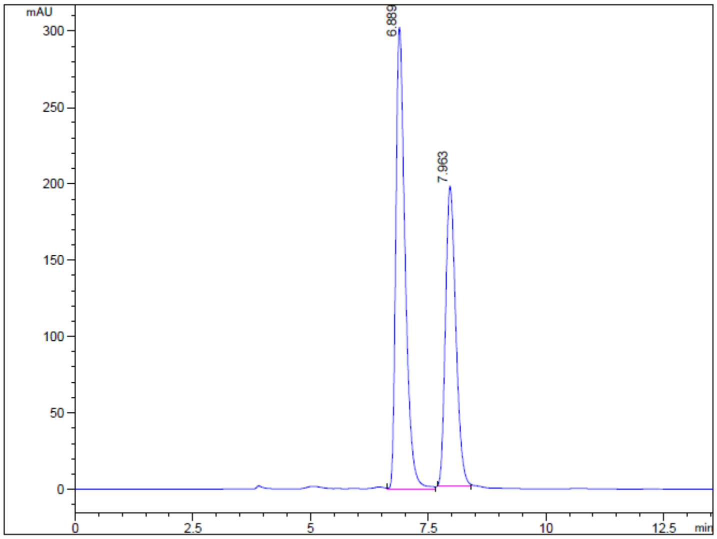

Signal 1: VWD1 A, Wavelength=280 nm

Area %	Peak #	RT [min]	Type	Width [min]	Area	Area %	Name
49.943	1	7.064	MM	0.243	377.238	49.943	
50.057	2	8.160	MM	0.269	378.104	50.057	

Signal 1: VWD1 A, Wavelength=280 nm

Area %	Peak #	RT [min]	Type	Width [min]	Area	Area %	Name
58.073	1	6.889	VV	0.207	4143.604	58.073	
41.927	2	7.963	MM	0.254	2991.573	41.927	

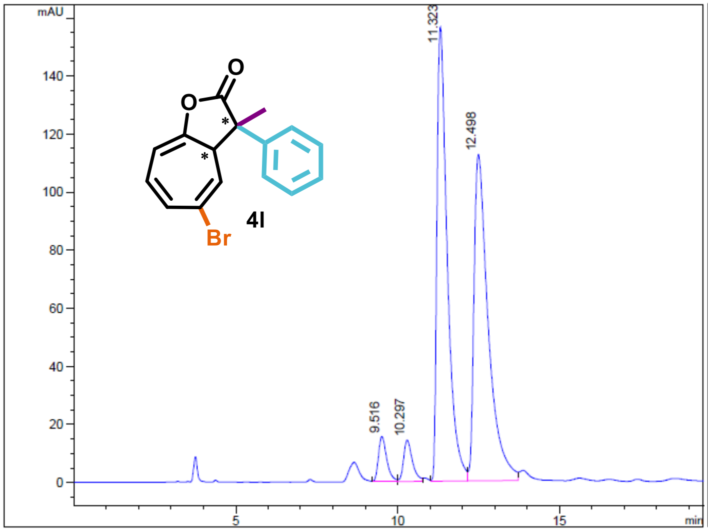

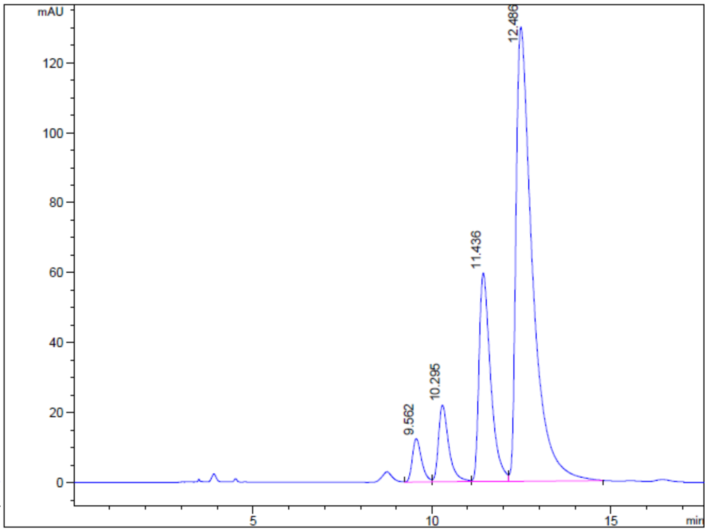

Signal 1: MWD1 F, Sig=280,16 Ref=360,100

Area %	Peak #	RT [min]	Type	Width [min]	Area	Area %	Name
3.742	1	9.516	VV	0.272	280.357	3.742	
3.617	2	10.297	VV	0.288	271.004	3.617	
46.470	3	11.323	VV	0.334	3481.656	46.470	
46.171	4	12.498	VV	0.455	3459.265	46.171	

Signal 1: MWD1 F, Sig=280,16 Ref=360,100

Area %	Peak #	RT [min]	Type	Width [min]	Area	Area %	Name
3.611	1	9.562	VV	0.273	222.738	3.611	
7.099	2	10.295	VV	0.301	437.865	7.099	
21.751	3	11.436	VV	0.345	1341.560	21.751	
67.539	4	12.486	VB	0.470	4165.685	67.539	

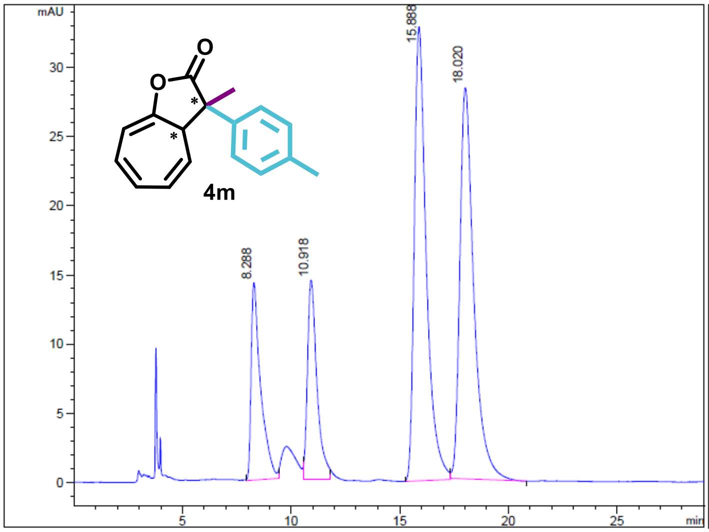

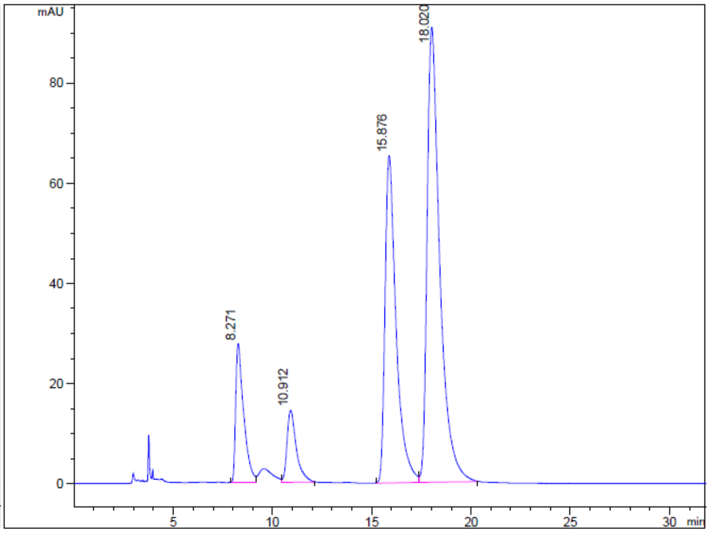

Signal 1: MWD1 E, Sig=280,16 Ref=360,100

Area %	Peak #	RT [min]	Type	Width [min]	Area	Area %	Name
12.802	1	8.288	MM	0.509	435.950	12.802	
12.819	2	10.918	MF	0.504	436.512	12.819	
37.177	3	15.888	BB	0.578	1265.978	37.177	
37.203	4	18.020	MM	0.746	1266.870	37.203	

Signal 1: MWD1 E, Sig=280,16 Ref=360,100

Area %	Peak #	RT [min]	Type	Width [min]	Area	Area %	Name
9.952	1	8.271	BV	0.402	780.047	9.952	
6.021	2	10.912	VB	0.492	471.894	6.021	
32.487	3	15.876	BV	0.577	2546.341	32.487	
51.540	4	18.020	VB	0.661	4039.706	51.540	

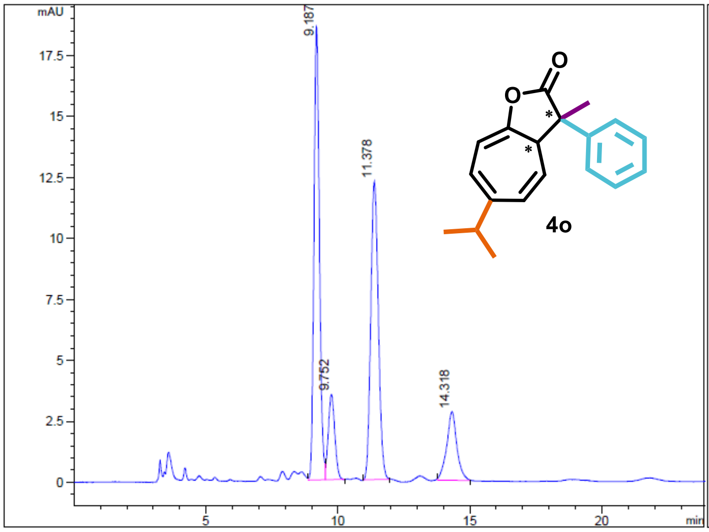

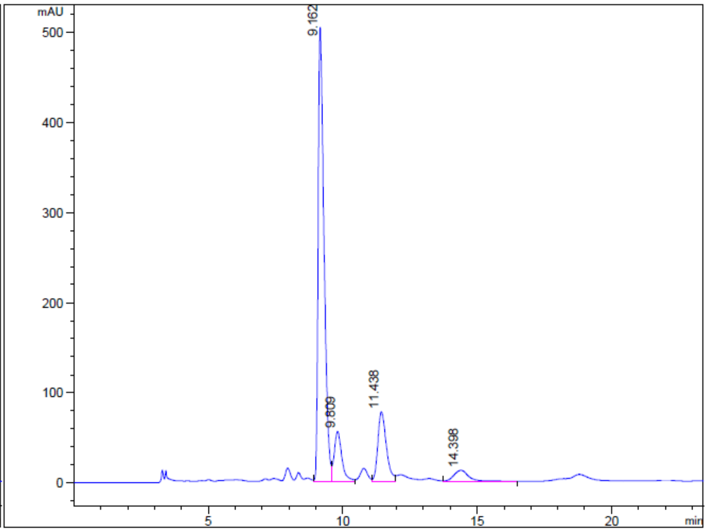

Signal 1: MWD1 E, Sig=280,16 Ref=360,100

Area %	Peak #	RT [min]	Type	Width [min]	Area	Area %	Name
41.348	1	9.187	VV	0.222	267.969	41.348	
9.275	2	9.752	VB	0.267	60.111	9.275	
37.692	3	11.378	BB	0.313	244.271	37.692	
11.685	4	14.318	BB	0.383	75.728	11.685	

Signal 1: MWD1 E, Sig=280,16 Ref=360,100

Area %	Peak #	RT [min]	Type	Width [min]	Area	Area %	Name
70.116	1	9.162	VV	0.229	7641.596	70.116	
10.037	2	9.809	VV	0.298	1093.912	10.037	
15.558	3	11.438	VV	0.337	1695.630	15.558	
4.288	4	14.398	VB	0.589	467.329	4.288	

HPLC chromatograms of derivatization products

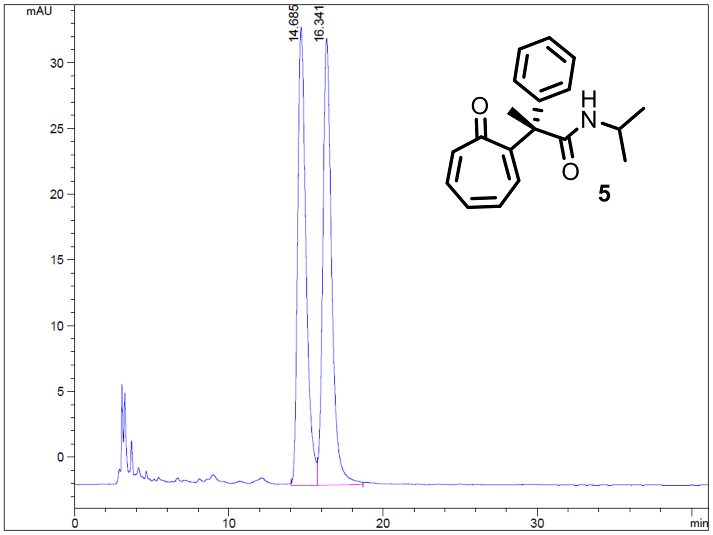

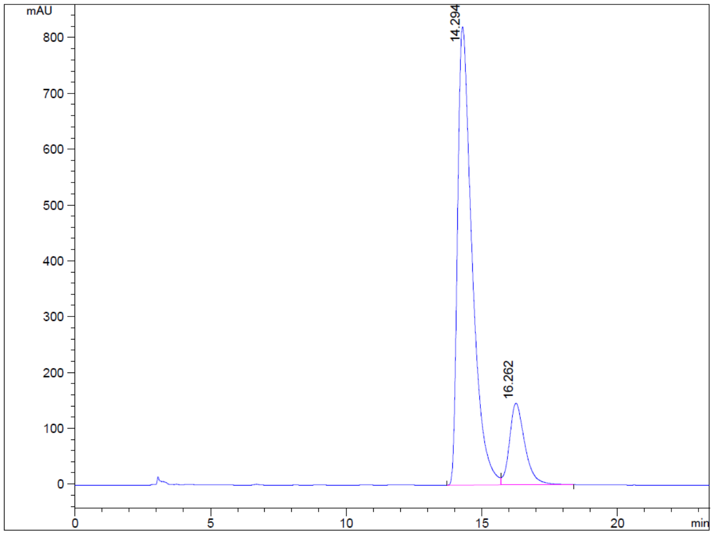

Signal 1: VWD1 A, Wavelength=230 nm

Area %	Peak #	RT [min]	Type	Width [min]	Area	Area %	Name
49.271	1	14.685	FM	0.633	1324.592	49.271	
50.729	2	16.341	MF	0.669	1363.807	50.729	

Signal 1: VWD1 A, Wavelength=230 nm

Area %	Peak #	RT [min]	Type	Width [min]	Area	Area %	Name
84.104	1	14.294	BV	0.558	30642.912	84.104	
15.896	2	16.262	VB	0.595	5791.637	15.896	

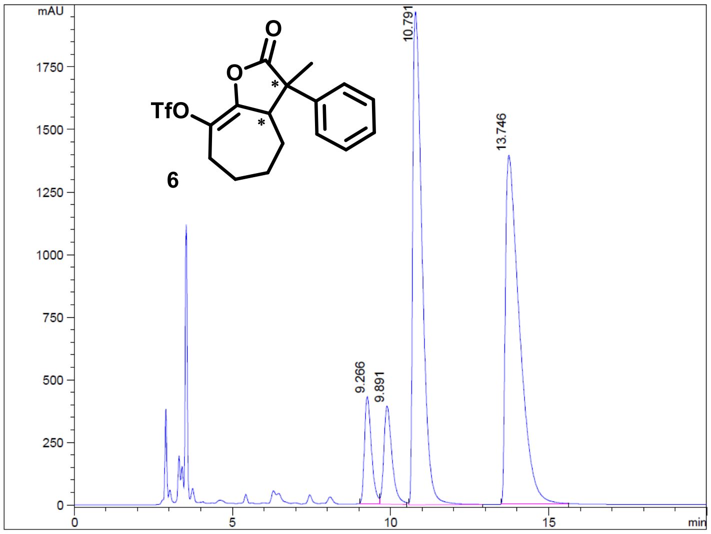

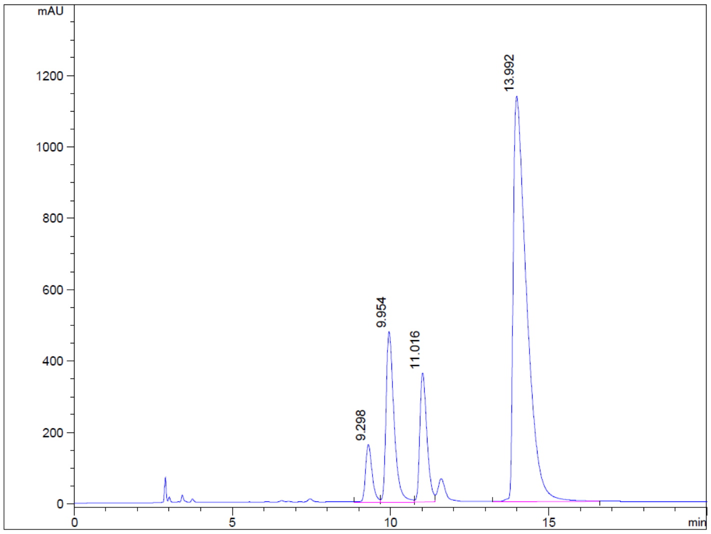

Signal 1: MWD1 C, Sig=210,8 Ref=360,100

Area %	Peak #	RT [min]	Type	Width [min]	Area	Area %	Name
6.822	1	9.266	MF	0.257	6634.871	6.822	
7.176	2	9.891	MF	0.296	6979.349	7.176	
41.733	3	10.791	MM	0.343	40591.070	41.733	
44.269	4	13.746	MM	0.516	43057.766	44.269	

Signal 1: MWD1 C, Sig=210,8 Ref=360,100

Area %	Peak #	RT [min]	Type	Width [min]	Area	Area %	Name
4.695	1	9.298	BV	0.221	2340.781	4.695	
16.743	2	9.954	VV	0.266	8347.448	16.743	
11.338	3	11.016	VV	0.241	5652.835	11.338	
67.224	4	13.992	BV	0.434	33515.293	67.224	

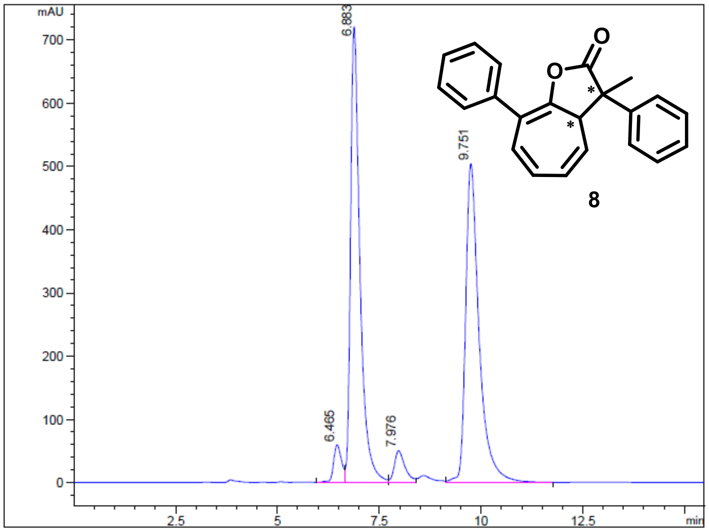

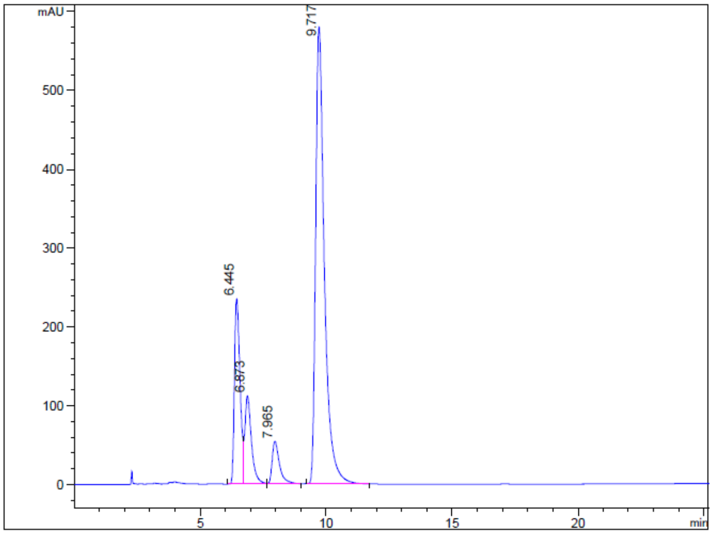

Signal 1: MWD1 B, Sig=254,16 Ref=360,100

Area %	Peak #	RT [min]	Type	Width [min]	Area	Area %	Name
3.257	1	6.465	VV	0.209	814.125	3.257	
46.465	2	6.883	VV	0.240	11614.247	46.465	
3.684	3	7.976	VV	0.273	920.783	3.684	
46.594	4	9.751	VB	0.342	11646.612	46.594	

Signal 1: MWD1 B, Sig=254,16 Ref=360,100

Area %	Peak #	RT [min]	Type	Width [min]	Area	Area %	Name
17.584	1	6.445	BV	0.238	3665.901	17.584	
9.836	2	6.873	VV	0.268	2050.511	9.836	
5.222	3	7.965	VB	0.301	1088.689	5.222	
67.358	4	9.717	BB	0.363	14042.298	67.358	

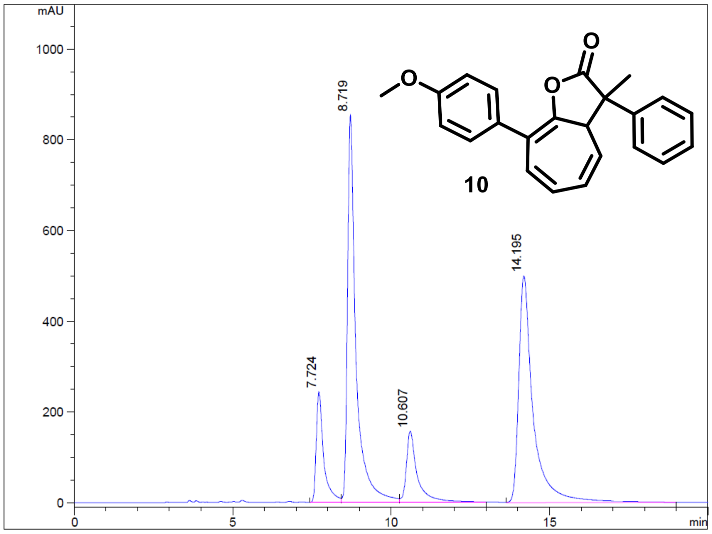

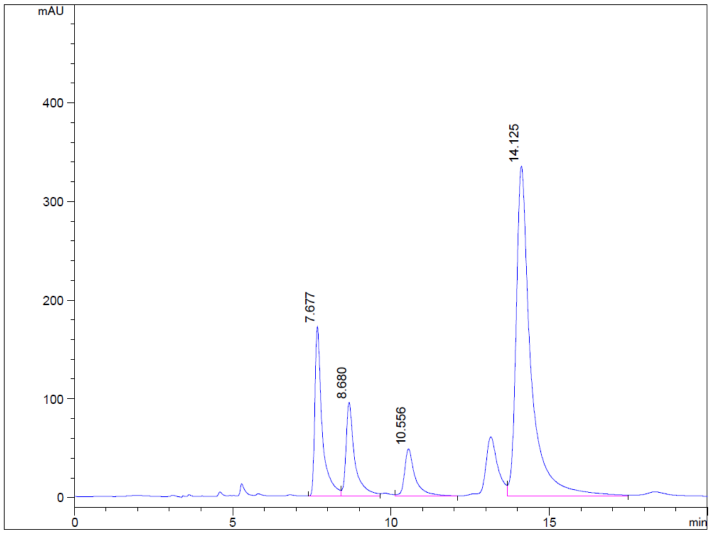

Signal 1: MWD1 B, Sig=254,16 Ref=360,100

Area %	Peak #	RT [min]	Type	Width [min]	Area	Area %	Name
10.035	1	7.724	VV	0.241	3999.595	10.035	
40.232	2	8.719	VV	0.269	16035.244	40.232	
9.661	3	10.607	VB	0.348	3850.403	9.661	
40.072	4	14.195	MM	0.532	15971.558	40.072	

Signal 1: MWD1 B, Sig=254,16 Ref=360,100

Area %	Peak #	RT [min]	Type	Width [min]	Area	Area %	Name
16.754	1	7.677	VV	0.231	2757.494	16.754	
11.113	2	8.680	VV	0.275	1828.977	11.113	
6.740	3	10.556	VB	0.335	1109.325	6.740	
65.392	4	14.125	VB	0.462	10762.455	65.392	

10. SCXRD analysis

Preparation of crystals suitable for single crystal X-ray analysis

Single crystals of **8** were obtained by slow evaporation placing 10 mg of compound **8** in 1 mL of dichloromethane. Colorless crystals were obtained after several days.

Single crystals of **4k** were obtained by slow evaporation by adding three drops of hexane to 3 mg of compound **4k** and then adding ethyl acetate until dissolved. Colorless crystals were obtained after several days. Compound **4j** was obtained under the same conditions as compound **4k**.

Single-Crystal X-ray Diffraction analysis (SCXRD)

Single crystal X-ray Diffraction data were acquired on a Rigaku XtaLAB Synergy-S diffractometer, equipped with a PhotonJet-S CuKα (λ = 1.54184 Å) radiation source, and HyPix-Arc 100° detector. Data of compounds **8** and **4j** were collected at room temperature, while compound **4k** in low temperature (160 K) with an open-flow nitrogen attachment from Oxford Cryosystem. CrysAlisPro program package[8] was used for data collection, data reduction, and absorption correction. Using Olex2[9] as the graphical interface, all crystal structures were solved with the SHELX structure solution program using Intrinsic Phasing methods (SHELXT).[10] The structures were refined with the SHELXL[11] refinement package using the least squares minimization on F^2. All non-hydrogen atoms were refined anisotropically, and hydrogen atom positions were calculated geometrically and placed in idealized positions. Absolute structure of compound **8** was determined by anomalous dispersion method, taking in account the Flack parameter.[12] Crystallographic data were deposited in the Cambridge Crystallographic Data Centre (CCDC, File Nos. 2543238, 2543239, 2543240) data and can be downloaded free of charge from CCDC database via www.ccdc.cam.ac.uk/structures.

The crystallographic data of compounds are summarized in **Table S5**. Compound **8** crystallizes in the $P2_12_12_1$ chiral space group, with one molecule comprised in the asymmetric unit. The crystal structure confirmed the *R*,*S* spatial arrangement of the chiral centers in this compound, determined via the anomalous dispersion method (**Figures S5** and **S6**).[12]

Both **4k** and **4j** compound packing in $P2_1/c$ space group and comprising one molecule in the asymmetric unit (**Figures S7-S10**). Since the crystal structure of these compounds exhibits a center of inversion symmetry, it is not possible to determine the absolute structure via SCXRD analysis.

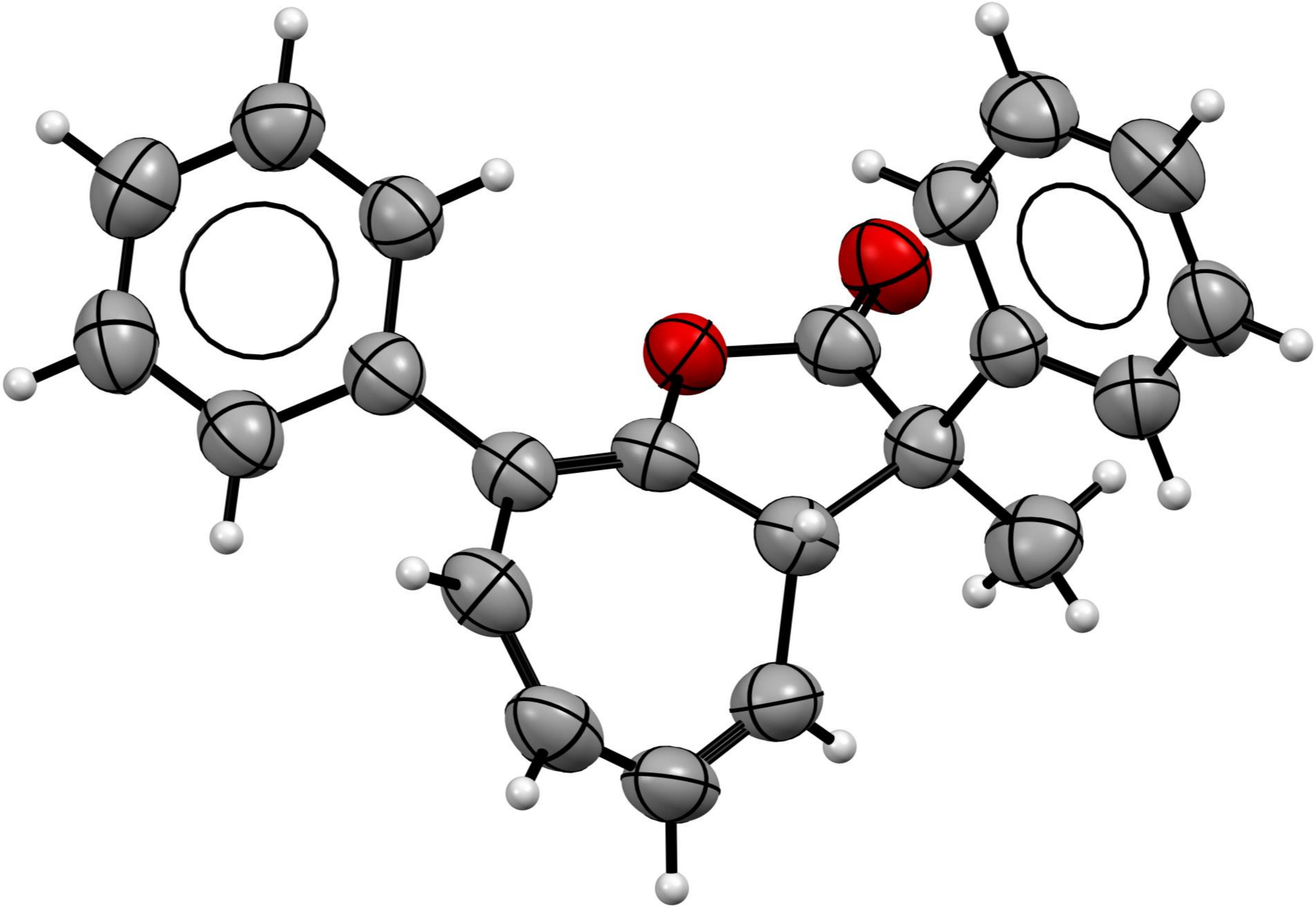

Figure S5. ORTEP projection of **8**. Nonhydrogen atoms are represented as thermal ellipsoids drawn at the 50% probability level.

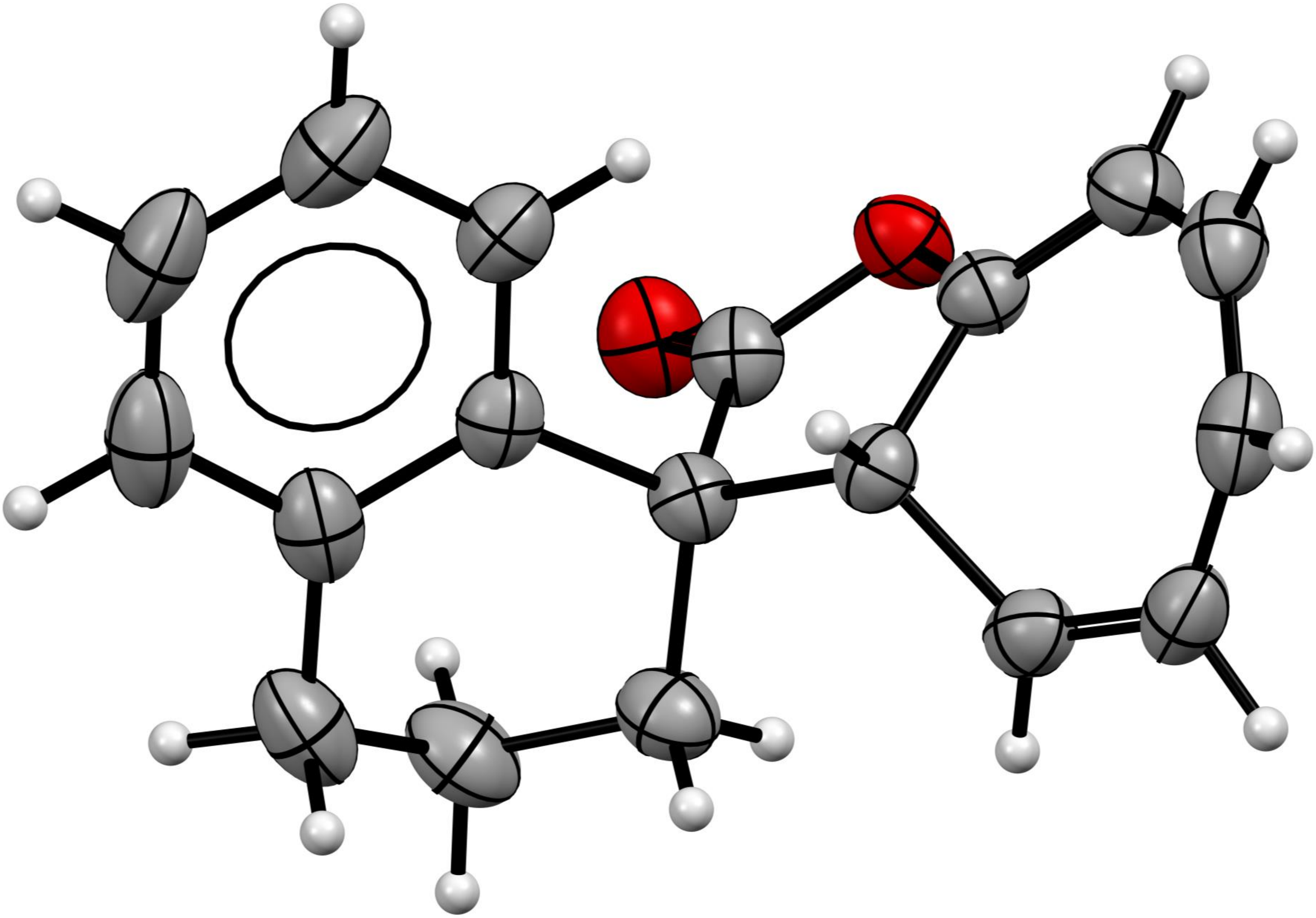

Figure S6. ORTEP projection of **4k**. Nonhydrogen atoms are represented as thermal ellipsoids drawn at the 50% probability level.

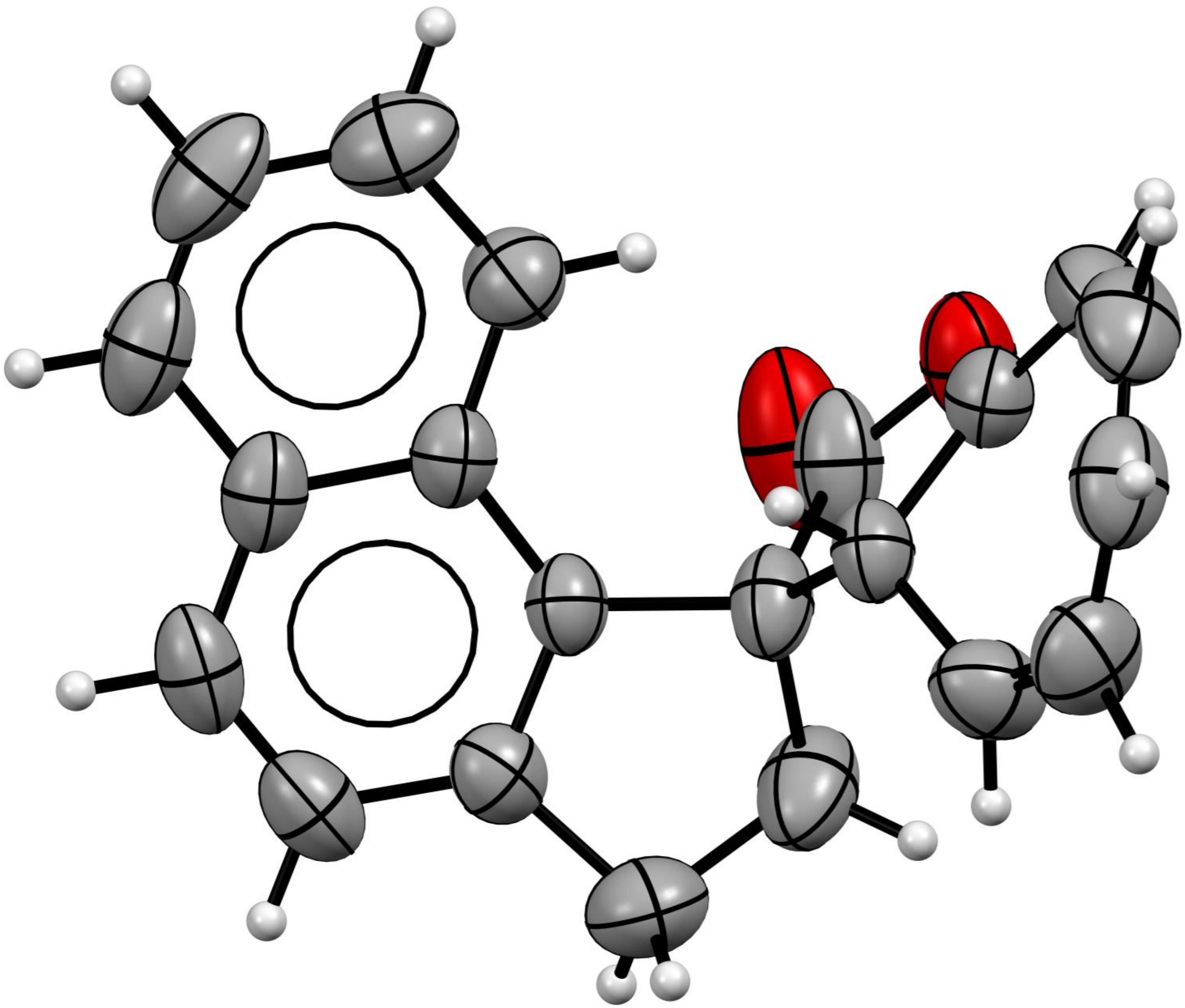

Figure S7. ORTEP projection of **4j**. Nonhydrogen atoms are represented as thermal ellipsoids drawn at the 50% probability level.

Table S5. Crystallographic data and structure refinement parameters for compounds.

Parameter	Compound		
	**8**	**4j**	**4k**
Chemical formula	$C_{22}H_{18}O_2$	$C_{21}H_{16}O_2$	$C_{18}H_{16}O_2$
Formula weight	314.36	300.34	264.31
Crystal color/shape	Colorless / block	Colorless / block	Colorless / plate
Crystal size (mm^3)	0.083×0.048×0.023	0.100×0.066×0.043	0.118×0.065×0.009
Temperature (K)	294.8(2)	294.58(10)	160.00(10)
Radiation source	CuKa (l=1.54184)	CuKa (l=1.54184)	CuKa (l=1.54184)
Crystal system	orthorhombic	Monoclinic	Monoclinic
Space group	$P2_12_12_1$	$P2_1$/c	$P2_1$/c
Z/Z'	4/1	4/1	4/1
a (Å)	7.7724(3)	9.4032(2)	11.3477(2)
b (Å)	12.2225(4)	12.2318(3)	8.8491(2)
c (Å)	17.5121(5)	13.7422(2)	13.5024(3)
α (°)	90	90	90
β (°)	90	106.665(2)	94.451(2)
γ (°)	90	90	90
Unit-cell volume ($Å^3$)	1663.62(10)	1514.21(6)	1351.78(5)
ρ_{calc}($g.cm^{-3}$)	1.255	1.317	1.299
μ (mm^{-1})	0.625	0.662	0.662
F(000)	664.0	632.0	560.0
2θ range for data collection (°)	8.822 to 137.36	9.818 to 136.496	7.814 to 149.244
Index ranges	-9 ≤ h ≤ 9, -14 ≤ k ≤ 14, -20 ≤ l ≤ 21	-11 ≤ h ≤ 11, -13 ≤ k ≤ 14, -16 ≤ l ≤ 14	-14 ≤ h ≤ 11, -11 ≤ k ≤ 11, -16 ≤ l ≤ 16
Reflections collected	18319	15520	27314
Data/restraints/parameters	3057/0/218	2768/0/208	2743/0/182
Largest diff. peak/hole (e $Å^{-3}$)	0.16/-0.22	0.70/-0.30	0.17/-0.15
Completeness (%)	100	100	99.9
R_{int}	0.0525	0.0227	0.0296
R_1[a] / wR_2[b]	0.0434 / 0.1204	0.0635 / 0.1962	0.0406 / 0.1099
Goodness-of-fit on F^2	1.059	1.089	1.060

[a] R_1 = □||F_o| − |F_c||/□|F_o|; [b] wR_2 = [□[$w(F_o^2 - F_c^2)^2$]/□[$w(F_o^2)^2$]]$^{1/2}$;
Flack parameter of **8**: 0.04(15); Hooft Parameter of **8**: 0.06(13)

11. Specifications and datasheets for the UV-LED lamps

Setup A

CREE PRODUCT FAMILY DATA SHEET

)-DS41 REV 18

Cree® XLamp® XT-E LEDs

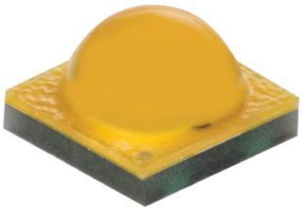

XT-E White

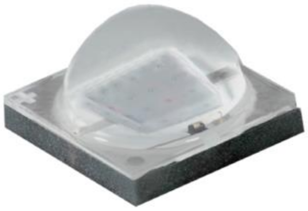

XT-E Royal Blue

PRODUCT DESCRIPTION

Optimized for directional, high-lumen applications, from indoor and outdoor to portable and lamp retrofits, the XLamp® XT-E LED delivers high performance and high reliability in the industry-standard XP/XT footprint. The XT-E LED offers the benefits of the XT/XP platform — compact and proven 3.45 mm x 3.45 mm package and established ecosystem — enabling lighting manufacturers to simplify the design process and shorten time to market.

The XT-E LED is available in royal blue and white. The XT-E White LED offers a high-efficacy option. In this document, the term White denotes the white XT-E LED without regard to its efficacy. The terms Standard and High Efficacy are used when necessary to differentiate the performace of the High Efficacy XT-E LED from the XT-E LED without the high-efficacy option.

FEATURES

- Maximum Vf for High Efficacy XT-E White: 2.85 V
- Available in 70-, 80- and 90-CRI minimum white
- Binned at 85 °C
- Available in 2200 K CCT
- Thermal resistance: White 5 °C/W, Royal Blue 3.5 °C/W
- Wide viewing angle: White 115°, Royal Blue 130°
- Maximum drive current: White 1.5 A, Royal Blue 1.5 A
- Electrically neutral thermal path
- Vf binning supported for XT-E White and Royal Blue
- XT-E Royal Blue sorted into 2.5-nm-wavelength bins
- Unlimited floor life at ≤ 30 °C/85% RH
- Reflow solderable - JEDEC J-STD-020C compatible
- RoHS and REACh compliant
- UL® recognized component (E349212)

NOTE: For remote phosphor applications, a separate license to certain Cree patents is required.

Copyright © 2011-2020 Cree, Inc. All rights reserved. The information in this document is subject to change without notice. Cree®, the Cree logo, EasyWhite® and XLamp® are registered trademarks of Cree, Inc. UL® and the UR logo are registered trademarks of UL LLC.

Cree, Inc.
4600 Silicon Drive
Durham, NC 27703
USA Tel: +1.919.313.5300

RELATIVE SPECTRAL POWER DISTRIBUTION - WHITE

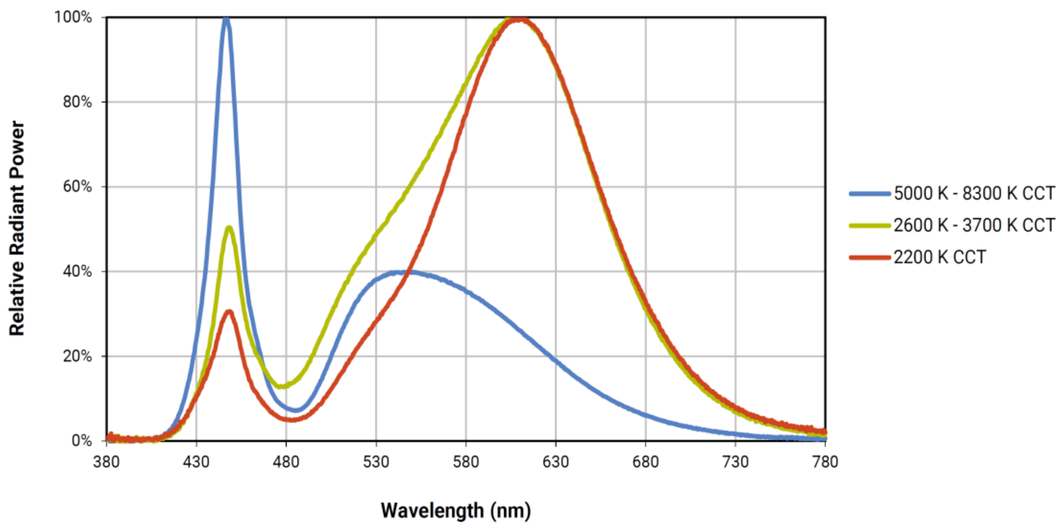

RELATIVE SPECTRAL POWER DISTRIBUTION - ROYAL BLUE

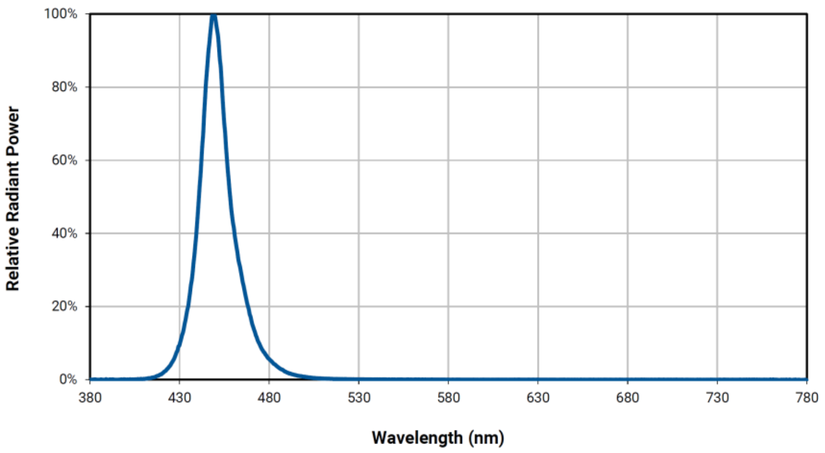

Copyright © 2011-2020 Cree, Inc. All rights reserved. The information in this document is subject to change without notice. Cree®, the Cree logo, EasyWhite® and XLamp® are registered trademarks of Cree, Inc. UL® and the UR logo are registered trademarks of UL LLC.

Setup B

ABI 23W Tuna Blue LED Bulb Coral Reef Optimized Spectrum 11-Band PAR38

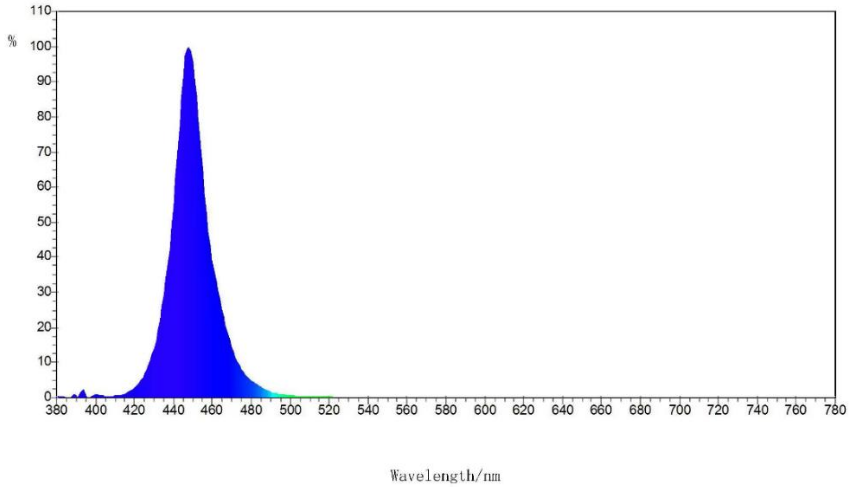

Dominant Wavelength: 453.2 nm

Optical Power: 970.96 mW (at 700.0 mA, 3.27 V)

12. References

[1] López, S.C., Bertuzzi, G., Bandini, M. Site-Selective Gold-Catalyzed Alkylation of α-Aminotropones with Allenes. *Org. Lett.* **2024**, *26*, 9251–9256. https://doi.org/10.1021/acs.orglett.4c03372.

[2] Jørgensen, A. K. Exploring Heterotropones and Examining Their Propensity to Undergo [4+2] Cycloadditions. *Org. Lett.* **2024**, *26*, 1539–1543. https://doi.org/10.1021/acs.orglett.3c04080.

[3] Jørgensen, A. K. Mechanistic Investigation of the Pseudo-Halogen Effect in Enantioselective Aminocatalyzed [6+4] and [10+6] Cycloadditions: Enabling Unique Favorskii-Like Rearrangements. *J. Am. Chem. Soc.* **2024**, *146*, 34231–34251. https://doi.org/10.1021/jacs.4c15353.

[4] Lewis, E. S., James D. T., Kim M. H. Azulene-Derived Fluorescent Probe for Bioimaging: Detection of Reactive Oxygen and Nitrogen Species by Two-Photon Microscopy. *J. Am. Chem. Soc.* **2019**, *141*, 19389–19396. https://doi.org/10.1021/jacs.9b09813.

[5] Bandini, M. Electrochemical Site-Selective Alkylation of Tropones via Formal C(sp^3)−C(sp^2) Coupling Reaction. *Adv. Synth. Catal*. **2024**, *366*, 1965 – 1971. https://doi.org/10.1002/adsc.202400050.

[6] Jørgensen, A. K. An Enantioselective Nucleophilic Aromatic tele-Substitution. *J. Am. Chem. Soc.* **2025**, *147*, 37854–37863. https://doi.org/10.1021/jacs.5c14328.

[7] Zhou, Q.-L., Zhu, S.-F. Enantioselective N-H Insertion Reaction of α-Aryl α-Diazoketones: An Efficient Route to Chiral α-Aminoketones. *Angew. Chem. Int. Ed.* **2014**, *53*, 3913 –3916. https://doi.org/10.1002/anie.201400236.

[8] CrysAlisPro Software System, Rigaku Corporation, Oxford, UK. 2025.

[9] Dolomanov, O. V., Bourhis, L. J., Gildea, R. J., Howard, J. A. K., Puschmann, H. OLEX2: A Complete Structure Solution, Refinement and Analysis Program. *J. Appl. Crystallogr.* **2009**, *42*, 339–341. https://doi.org/10.1107/S0021889808042726.

[10] Sheldrick, G. M. SHELXT - Integrated Space-Group and Crystal-Structure Determination. *Acta Crystallogr. Sect. A Found. Crystallogr.* **2015**, *71*, 3–8. https://doi.org/10.1107/S2053273314026370.

[11] Sheldrick, G. M. Crystal Structure Refinement with SHELXL. *Acta Crystallogr. Sect. C Struct. Chem.* **2015**, *71* (Md), 3–8. https://doi.org/10.1107/S2053229614024218.

[12] Flack, H. D., Bernardinelli, G. Absolute Structure and Absolute Configuration. **1999**, No. A55, 908–915. https://doi.org/10.1107/S0108767399004262.; Flack, H. D., Bernardinelli, G. Reporting and Evaluating Absolute-Structure and Absolute-Configuration Determinations. **2000**, *33*, 1143–1148. https://doi.org/10.1107/S0021889800007184.; Linden, A. Best Practice and Pitfalls in Absolute Structure Determination. *Tetrahedron: Asymmetry* **2017**, *28*, 1314–1320. https://doi.org/10.1016/j.tetasy.2017.07.010.
